# Chimeric oligosaccharide conjugate induces opsonic antibodies against *Streptococcus pneumoniae* serotypes 19A and 19F†

**DOI:** 10.1039/d0sc02230f

**Published:** 2020-06-26

**Authors:** Someswara Rao Sanapala, Bruna M. S. Seco, Ju Yuel Baek, Shahid I. Awan, Claney L. Pereira, Peter H. Seeberger

**Affiliations:** Department of Biomolecular Systems, Max Planck Institute of Colloids and Interfaces Am Mūhlenberg 1 D-14424 Potsdam Germany peter.seeberger@mpikg.mpg.de; Department of Chemistry and Biochemistry, Freie Universität Berlin Arnimallee 22 D-14195 Berlin Germany

## Abstract

*Streptococcus pneumoniae* 19A (ST19A) and 19F (ST19F) are among the prevalent serotypes causing pneumococcal disease worldwide even after introduction of a 13-valent pneumococcal conjugate vaccine (PCV13). Synthetic glycoconjugate vaccines have defined chemical structures rather than isolated polysaccharide mixtures utilized in marketed vaccines. Ideally, a minimal number of synthetic antigens would cover as many bacterial serotypes to lower cost of goods and minimize the response to carrier proteins. To demonstrate that a chimeric oligosaccharide antigen can induce a protective immune response against multiple serotypes, we synthesized a chimeric antigen (ST19AF) that is comprised of a repeating unit of ST19A and ST19F capsular polysaccharide each. Synthetic glycan epitopes representing only ST19A, and ST19F were prepared for comparison. Semisynthetic glycoconjugates containing chimeric antigen ST19AF induced high antibody titers able to recognize native CPS from ST19A and ST19F in rabbits. The antibodies were able to kill both strains of *pneumococci*. Chimeric antigens are an attractive means to induce an immune response against multiple bacterial serotypes.

## Abstract 2

Chimeric antigens are an attractive means to induce an immune response against multiple bacterial serotypes. The chimeric semisynthetic glycoconjugate ST19AF induced antibodies with opsonic activity able to kill ST19A and ST19F bacteria in rabbits.

## Graphical abstract

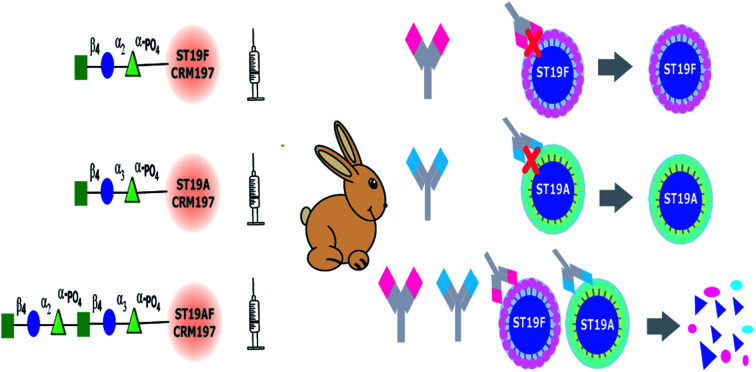

## Introduction

Invasive pneumococcal diseases (IPD) caused by *Streptococcus pneumoniae* (Sp), are a major cause of morbidity and mortality in toddlers and older adults worldwide.^2,3^ Despite the introduction of a 13-valent pneumococcal conjugate vaccine (PCV13) in 2010, ST19A remains a major pathogen worldwide^4–8^ that is associated with 14% of all IPD cases.^9^ Since the efficacy of PCV 13 against ST19A is being debated^4,10,11^ the development of new vaccine approaches covering ST19A and ST19F is desirable.

Currently glycoconjugate vaccines contain polysaccharides isolated from bacterial cell cultures, that are conjugated to proteins to create heterogeneous vaccine compositions.^12^ The ST19 CPS is among the most labile polysaccharides used for vaccine production as the phosphate diester groups can be cleaved during purification and conjugation resulting in severely suppressed immunogenicity.^13,14^ Phosphate diesters in the polysaccharide of *Salmonella typhimurium* and *Clostridium difficile* PSII are crucial for inducing a strong immune response to the native polysaccharide.^15,16^ Synthetic oligosaccharide conjugates containing well-defined antigens, have proven very effective in preventing bacterial infections caused by encapsulated bacteria,^17–27^ and the conjugation process does not impair antigens having phosphate diester groups. The saccharide to protein ratio (glycan loading) is an important parameter for glycoconjugate vaccine development. The novel strategy described here, helps to reduce the amount of protein used and may simplify the formulation of multicomponent vaccines against bacterial pathogens.

**Fig. 1:**
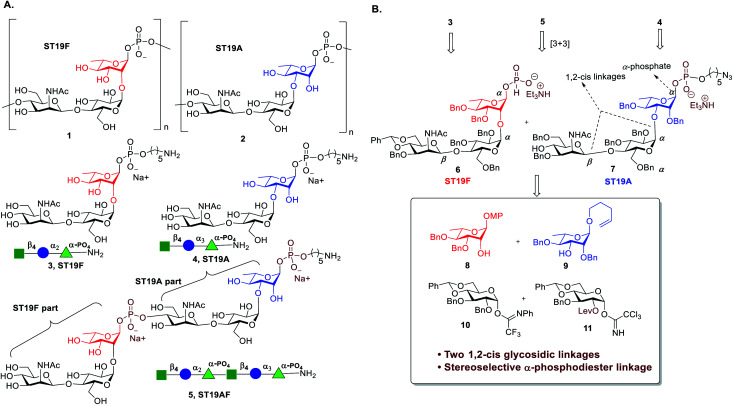
(A) Structures of the *S. pneumoniae* CPS repeating units of serotypes ST19F (1), ST19A (2) and synthetic oligosaccharide antigens **3**, and **4** that resemble these repeating units as well as chimeric oligosaccharide **5**; (B) Retrosynthetic analysis of **3**, **4**, and **5**.

The ST19 CPS are homopolymers that are comprised of a common β-d-ManpNAc-(1 → 4)-α-d-Glcp disaccharide linked to C2 (ST19F **1**) or C3 of L-Rha (ST19A **2**) respectively (Fig. 1A).^28,29^ Several oligosaccharide antigens related to ST19A^30−32^ and ST19F^33−36^ have been synthesized but neither the anomeric phosphate was stereoselectively constructed nor any biological studies were conducted. The synthesis and immunological evaluation of both antigens is important to establish a better fundamental understanding of the exact epitopes responsible for a protective immune response.

Almost 100 *S. pneumoniae* serotypes can cause IPD.^37^ Marketed glycoconjugate vaccines contain up to 13 different CPS as new formulations with up to 20 serotypes are under development.^38^ As more serotypes are included, antigens that can induce a protective immune response against more than one serotype would be particularly valuable. Multivalent vaccines based on glyco-nanoparticles,^39^ or created by Ugi-multicomponent reactions as well as bivalent unimolecular vaccines have been explored.^40^ Chimeric antigens that combine the repeating unit sequences of two different serotypes in one oligosaccharide that is then conjugated to a carrier protein have not been reported. We combine ST19F (**3**) and ST19A (**4**) *via* a phosphodiester linkage to form the chimeric oligosaccharide antigen ST19AF (**5**) (Fig. 1A). A terminal C5-amino linker enables further site-specific conjugation to a carrier protein, to induce protective antibodies against both serotypes using the chimeric antigen (Fig. 1A).

## Results and discussion

**Scheme 1:**
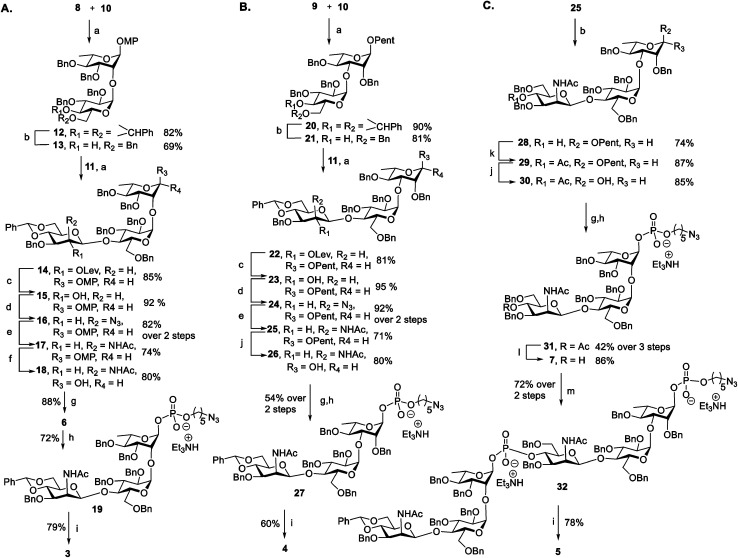
Synthesis of antigens ST19F **3** (A), ST19A **4** (B) and chimeric ST19AF **5** (C) TMSOTf, CH_2_Cl_2_, MS 4 Å, −20 °C, 2 h; (b) Et_3_SiH, TFA, TFAA, DCM, MS 4 Å, 0 °C – rt; (c) NH_2_–NH_2_, AcOH, CH_2_Cl_2_–MeOH (3 : 1), rt, 1 h; (d) (1) 1,1-sulfonyldiimidazole, NaH, DMF, −40 °C, 4 h; (2) TBAN_3_, toluene, 100 °C, 3 h; (e) Zn–Cu Couple, THF : Ac_2_O : AcOH (3 : 3 : 1), rt, 15 h; (f) CAN, CH_3_CN–toluene–H_2_O, rt, 10 h; (g) 2-chloro-1,3,2-benzodioxaphos-yphorin-4-one, H_3_PO_3_, Py, rt, 2 h then 50 °C, 36 h; (h) (1) HO(CH_2_)_5_N_3_, PivCl, pyridine, rt, 1.5 h; (2) I_2_, Py : H_2_O (18 : 2), −40 °C, 30 min then TEAB; (i) (1) H_2_, Pd/C, EtOAc : MeOH : H_2_O, 3 d; (2) Dowex 50W X4, Na^+^; (j) NBS, CH_3_CN, 0 °C – rt, 2 h; (k) Ac_2_O, Py, 0 °C, 1 h; (l) 0.5 M NaOMe, MeOH, rt, 3 h; (m) **6**, PivCl, pyridine, rt, 2 h.

Antigen construction of **3**, **4**, **5** requires the stereoselective installation of two 1,2-*cis* glycosidic linkages and the stereoselective formation of an α-phosphodiester linkage to the trisaccharide (Fig. 1B). Trisaccharides **6** or **7** can be synthesized *via* a linear synthetic approach from the reducing to the non-reducing end using the building blocks **8**,^41^**9**,^42^**10**,^43^ and **11**.^44^ For the assembly of trisaccharide building block **6**, glucosyl imidate **10** was coupled to rhamnosyl acceptor **8** to obtain α-linked disaccharide **12** (^1^*J*_C–H_ = 170.5 Hz) in 82% yield (Scheme 1A). Regioselective reductive ring opening of the benzylidene acetal in **12** using triethylsilane and trifluoroacetic acid delivered acceptor **13** in 69% yield. Disaccharide acceptor **13** was glycosylated with glucosyl imidate **11** to afford trisaccharide **14** in 85% yield (doublet, *J* = 8.0 Hz, ^1^*J*_C–H_ = 167.9 Hz). Selective levulinoyl ester (Lev) cleavage (92%) using hydrazine acetate followed by inversion of the 2-hydroxyl group was achieved by conversion of the corresponding sulfonyl diimidazole and subsequent nucleophilic displacement with tetrabutylammonium azide to obtain **16** (82% over two steps). The inversion of stereochemistry was confirmed by examination of the ^1^H–^1^H and ^13^C–^1^H coupling constants^45,46^ to H-1 at *δ* 4.43 (doublet, *J* = 1.4 Hz) and ^1^*J*_C–H_ = 163.2 Hz. Conversion of the azide to the corresponding acetamido group was obtained by treatment with freshly activated Zn–Cu couple to give **17** in 74% yield. Removal of the *p*-methoxyphenyl group using ceric ammonium nitrite (CAN) gave hemiacetal **18**. The installation of a stereoselective H-phosphonate at the reducing end of phosphate diester **19** was achieved with phosphorous acid and an excess of sterically bulky 2-chloro-5,5-dimethyl-2-oxo-1,3,2-dioxaphosphorinane^41^ in pyridine at 50 °C to obtain thermodynamically more stable α-H-phosphonate **6** (^31^P NMR: *δ* 0.91, ^1^*J*_P,H_ = 641.8, ^3^*J*_P,H-1_ = 7.5 Hz & ^1^H NMR: *δ* 5.70, ^1^*J*_C,H_ = 176 Hz). The selectivity may result from conversion of the more reactive β-glycosyl H-phosphonate to the thermodynamically more stable α-phosphonate by S_N_2 displacement with excess phosphorous acid.^47^ The protected phosphodiester-linked trisaccharide **19** was prepared by coupling H-phosphonate **6** with 5-azido pentanol using pivaloyl chloride in pyridine as a condensing agent followed by oxidation with iodine in water–pyridine. Hydrogenation of **19** provided deprotected trisaccharide sodium salt **3** after passing through Dowex 50W X4 Na^+^ resin as a single diastereomer.

The synthesis of ST19A trisaccharide **4** followed a similar synthetic approach (Scheme 1B). Orthogonal glycosylation between glucosyl imidate **10** and *n*-pentenyl acceptor **9** gave α-linked disaccharide **20** (^1^*J*_C,H_ = 172.0 Hz), which was subjected to reductive benzylidene acetal cleavage to obtain **21**. Disaccharide **21** containing a 4-hydroxyl group was coupled with glycosyl imidate **11** to give trisaccharide **22** (*δ* 4.42, doublet, *J* = 8.0 Hz, ^1^*J*_C–H_ = 165.2 Hz). Cleavage of the Lev group followed by stereoselective inversion of the C2-hydroxyl group in **23** yielded the corresponding azide **24** (*δ* 4.29, singlet, ^1^*J*_C,H_ = 162.2 Hz). Reduction of the azide to the corresponding acetamido derivative using Zn–Cu couple afforded **25**. Hydrolysis of pentenyl glycoside delivered hemiacetal **26**, which was converted into the azidopentyl linked phosphonate derivative **27***via* the H-phosphonate method. Hydrogenation using Pd/C in EtOAc : MeOH : H_2_O (3 : 2 : 1) provided deprotected trisaccharide as triethylammonium salt that was converted into corresponding sodium salt using Dowex 50W X4 Na^+^ resin to obtain white solid **4** as a single diastereomer (*δ*_P_ −1.92, ^1^*J*_C,H_ = 174.2 Hz).

With trisaccharides **25** and **6** in hand, the synthesis of the chimeric antigen **5** commenced (Scheme 1C). Reductive cleavage of the benzylidene acetal in trisaccharide **25** was achieved using triethylsilane and TFA to give **28**, that was acetylated at the 4-OH to furnish **29**. Hydrolysis of the pentenyl glycoside followed by H-phosphonate installation gave solely α-glycosyl phosphate **31** (*δ*_P_ −2.64, ^3^*J*_P,H-1_ = 8.1 Hz, ^1^*J*_C,H_ = 176.0 Hz). Acetate ester cleavage furnished acceptor **7** that was condensed with ST19F H-phosphonate **6** to afford hybrid ST19AF **32**. Finally, hexasaccharide **32** was deprotected by hydrogenolysis to provide the disodium salt of antigen **5** following exchange with Dowex 50W X4 Na^+^ resin. The antigens **3**, **4**, and **5** were obtained as white solids following purification by reverse phase C18-column HPLC and lyophilization.

**Fig. 2:**
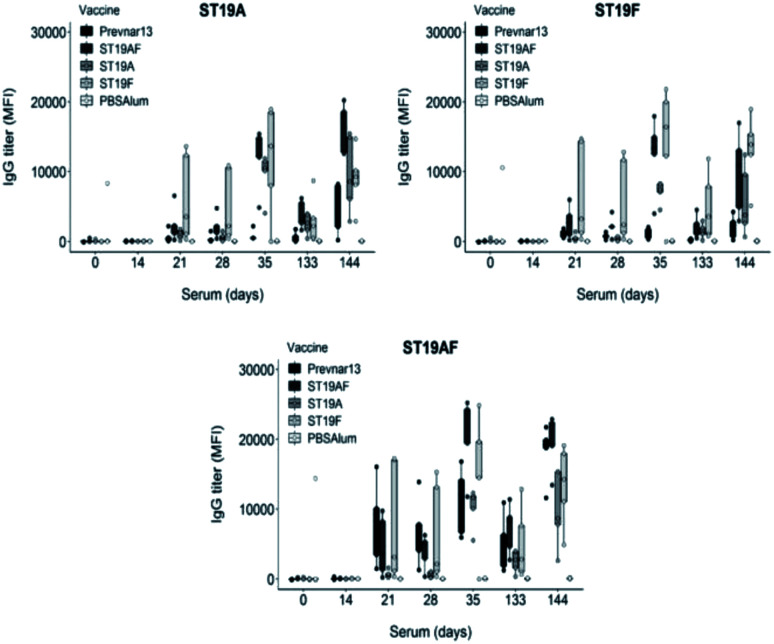
Semisynthetic glycoconjugates ST19A, ST19F or ST19AF induce antibody production and a long-term memory response. Zika rabbits (*n* = 5 per group) were immunized four times (days 0, 14, 28, and 133) intramuscularly with semisynthetic glycoconjugate vaccine ST19A, ST19F or ST19AF in aluminum hydroxide as well as positive control Prevnar13® and negative control PBS + aluminum hydroxide. Polysaccharide-specific antibody titers were analyzed by glycan microarray. The 1 : 100 sera dilution was used in the analysis. MFI = mean fluorescence intensity.

The synthetic antigens **3–5** were conjugated to CRM197 with an average of seven, five and five oligosaccharides attached per CRM197 molecule respectively as determined by MALDI analysis. Rabbits were immunized with conjugates containing the chimeric ST19AF, or the ST19A or ST19F antigens formulated with aluminum hydroxide. The conjugates induced strong anti-glycan antibody titers after delivery of three vaccine doses at two week intervals. The long term immune response was confirmed following a boost three and a half months after the last immunization (day 133). High antibody titers were faster attained showing that memory B cells were activated and able to generate antibodies against the vaccine antigen (day 144) (Fig. 2). Rabbits immunized with Prevnar13®, a marketed vaccine containing both native CPS serotypes, produced higher antibody titers against the hybrid ST19AF when compared to ST19A and ST19F antigens (Fig. 2). Increased binding to the chimeric oligosaccharide may be a result of an ionic interaction with the phosphate diester connecting the two repeating units. Ionic protein–glycan interactions are known for cell recognition^48,49^ and antibody–glycan interactions.^15^

**Fig. 3:**
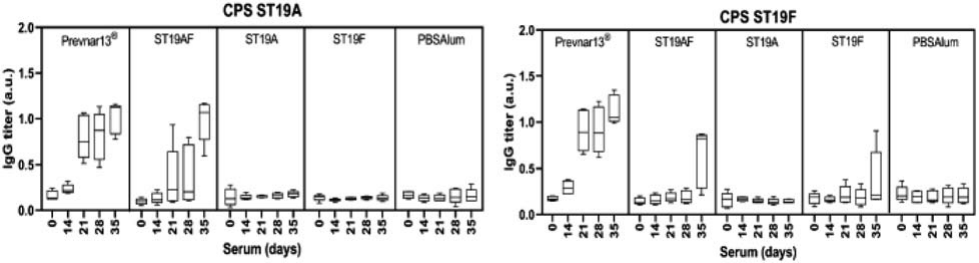
Antibodies produced in response to semisynthetic glycoconjugate ST19AF recognize native CPS19A and CPS19F. Zika rabbits (*n* = 5 per group) were immunized three times (days 0, 14, 28) intramuscularly with semisynthetic ST19A, ST19F or ST19AF glycoconjugate formulated with aluminum hydroxide as well as positive control Prevnar13® and negative control PBS + aluminum hydroxide. Polysaccharide-specific antibody titers were analyzed by ELISA. The 1 : 100 sera dilution was used in the analysis. a.u. = absorbance units.

Rabbit antibodies raised in response to glycoconjugate vaccination were tested against native CPS of ST19A (CPS19A) and ST19F (CPS19F) to determine the cross-reactivity of antibodies produced against synthetic or native antigens. Chimeric ST19AF generated antibodies that recognize both CPS. Anti-CPS19A antibody titer was highest after three doses (day 35). ST19A antigen at the reducing-end of the chimeric structure may favor a better B-cell presentation due to the proximity to the carrier protein presented *via* MHC class II (Fig. 3).^50^ ST19F conjugate induced low antibody titers against CPS19F while ST19A did not generate antibodies recognizing native CPS19A although antibodies against the synthetic antigen were detected (Fig. 2). The absence of the phosphate diester in the shorter antigens might impair the generation of antibodies that can recognize native CPS since Prevnar13®, that contains the native repeating units (RU) in varying length, is able to induce a high immune response. Antibodies raised against ST19A and ST19F did not cross react showing the importance of l-rhamnose linkage^48,49^ in generating specific antibodies against native CPS of different bacteria (Fig. 3).

**Fig. 4:**
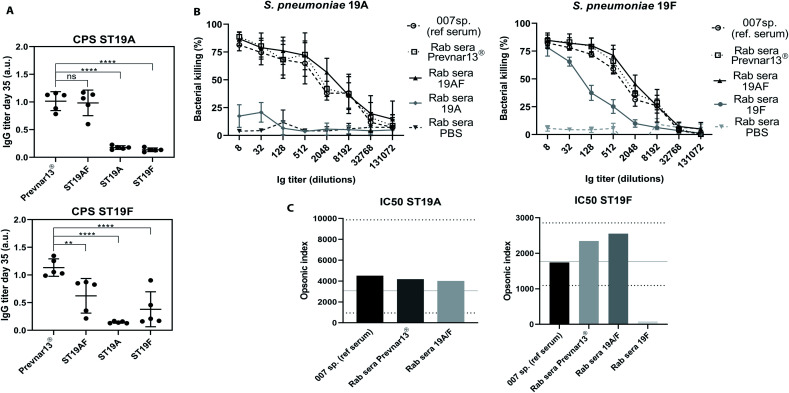
ST19AF antigen induced antibodies with opsonophagocytic activity. (A) IgG titer of rabbits (*n* = 5 per group) immunized with Prevnar13® and semisynthetic glycoconjugates ST19AF, ST19A and ST19F after three doses was used in OPKA assay. Antibody titers were compared to the commercially available vaccine Prevnar13® and statistical differences were determined using ANOVA. (B) *In vitro* OPKA of pooled rabbit sera raised against semisynthetic glycoconjugates ST19A, ST19F and ST19AF. The results were compared to Prevnar13® and to the reference serum 007sp. Rabbits immunized with PBS and aluminum hydroxide was used as a negative control. Data presented as mean ± SD of two independent experiments in duplicate. (C) Opsonic index refers to the sera dilution where 50% of bacteria are killed and was determined by four parameters logistic regression. The OI of ST19AF synthetic glycoconjugate was in accordance to the 007sp. reference serum, where the mean (black line) and 95% CI (dotted lines) are represented.^1^ a.u. – absorbance unit. ****p < 0.0001, **p < 0.001.

Antibody-mediated bacterial killing *in vitro*, was determined by an opsonophagocytic killing assay (OPKA) with sera of rabbits immunized with three doses of ST19A, ST19F, ST19AF, Prevnar13®, or PBS and aluminium hydroxide (Fig. 4A). The anti-pneumococcal reference serum (007sp) served as standard reference.^51^ ST19AF glycoconjugate elicits opsonic antibodies able to kill both bacterial serotypes. Anti-ST19F antibodies had a very weak opsonic activity while antibodies induced by the ST19A conjugate were not bactericidal (Fig. 4B), reflecting the weak or absent recognition of native capsular polysaccharide (Fig. 3). The ST19AF serum dilution for 50% bacterial killing is within the range of opsonic activity of the standard sera 007sp. (Fig. 4C). While the antibody titer produced in response to ST19AF was lower than that for Prevnar13® against CPS19F, the opsonic activity of both vaccines were similar. Thus, the quality of antibodies generated with the ST9AF vaccine overcame the reduced antibody titer. Using just one antigen to protect against two serotypes is helpful for reducing the amount of carrier protein in vaccine formulations containing different serotypes.

## Conclusions

We synthesized a chimeric oligosaccharide antigen containing the repeating units of ST19A and ST19F capsular polysaccharides as well as synthetic glycan epitopes resembling ST19A and ST19F. The chimeric antigen glycoconjugate induced an excellent immune response in rabbits. The antibodies produced in response to the chimeric antigen killed ST19A and ST19F bacteria, while the conjugates containing the other glycan epitopes failed to do so. With an increasing number of serotypes to be included in vaccines due to serotype replacement, ideally, each antigen can induce an immune response against more than one serotype, reducing the chemical steps needed for synthetic vaccine production. These findings will expedite glycoconjugate vaccine development as co-formulation studies containing chimeric antigens will help to reduce the amount of carrier protein in semi-synthetic glycoconjugate vaccines.

## Materials and methods

Oligosaccharide antigens were synthesized using standard protocols and conjugated to CRM197. Synthetic antigens were printed on NHS-activated microarray slides. Animal experiment (project 49062) was performed in strict accordance with the NIH/OLAW Animal Welfare Assurance, identification number F16-00178 (A5755-01) and was authorized by LALLF MV (Landesamt für Landwirtschaft, Lebensmittelsicherheit und Fischerei Mecklenburg-Vorpommern, Germany) in accordance to TierSchG 7221.3-2-002/19. The immune response was analyzed by glycan microarrays and ELISA. The functional attribute of the immune response was monitored by OPKA using HL-60 cells. Bacteria serotypes ST19A and ST19F were isolates from Charité Infektiologie und Pneumologie department, Universitätsmedizin Berlin. Detailed materials and methods can be found in ESI Appendix.†

## Conflicts of interest

None of the authors declare a conflict of interest.

## Supplementary Material

SC-011-D0SC02230F-s001
