# Supplementary material for: Chimeric oligosaccharide conjugate induces opsonic antibodies against Streptococcus pneumoniae serotypes 19A and 19F
Source: Chem Sci. 2020 Jun 26;11(28):7401–7. doi: 10.1039/d0sc02230f (PMC8159444; doi:10.1039/d0sc02230f)
Supplement: SC-011-D0SC02230F-s001 [file SC-011-D0SC02230F-s001.pdf]

Electronic Supplementary Material (ESI) for Chemical Science.
This journal is © The Royal Society of Chemistry 2020

Supplementary Information for

Chimeric Oligosaccharide Conjugate Induces Opsonic Antibodies against *Streptococcus pneumoniae* Serotypes 19A and 19F

Someswara Rao Sanapala,[†,1,2] Bruna M. S. Seco,[†,‡,1] Ju Yuel Baek,[†] Shahid I. Awan,[†] Claney L. Pereira,[‡,2] and Peter H. Seeberger*[,†,‡,3]

[†] Department of Biomolecular Systems, Max Planck Institute of Colloids and Interfaces, Am Mühlenberg 1, D-14424 Potsdam, Germany

‡ Department of Chemistry and Biochemistry, Freie Universität Berlin, Arnimallee 22, D-14195 Berlin, Germany

AUTHOR INFORMATION

*Corresponding author:

Prof. Dr. Peter H. Seeberger

Author Contributions:

[1]S.R.S. and B.M.S.S. contributed equally to this work.

[2]Present address: Vaxxilon Deutschland GmbH, Magnusstraße 11,12489 Berlin, Germany.

[3]To whom correspondence may be addressed. Email: peter.seeberger@mpikg.mpg.de.

This PDF file includes:

Supplementary text

Figures S1 to S3

Spectra

SI References

Table of Contents

1. General information………………………………………………………………… S3

2. Synthetic procedures ………………...…………………………………………...... S3

3. Conjugation of synthetic antigens 3, 4, 5 to CRM197…………………..……. S33

4. Vaccine formulation………………………………………………………………… S35

5. Rabbit immunizations ……………………………………………….……....... S35

6. Enzyme-Linked Immunosorbent Assay (ELISA)....................................... S36

7. Glycan microarrays………………………………………………………………… S36

8. In vitro opsonophagocytic killing assay…………………………………….. S40

9. Spectra…………………………………………………………………………….. S41

References………………………………………………………………………… S105

Experimental Section

1. General Information

Commercial grade solvents were used unless stated otherwise. Anhydrous solvents were obtained from a Waters Dry Solvent System. Solvents for chromatography were distilled prior to use. Sensitive reactions were carried out in heat-dried glassware and under an argon atmosphere. Analytical thin layer chromatography (TLC) was performed on Kieselgel 60F254 glass plates pre-coated with a 0.25 mm thickness of silica gel. Spots were visualized by Hanessian's stain [5% (w/v) ammonium molybdate, 1% (w/v) cerium(II) sulfate and 10% (v/v) sulfuric acid in water]. Silica column chromatography was performed on Fluka Kieselgel 60 (230-400 mesh).

^{1}H, ^{13}C, ^{31}P and two-dimensional NMR spectra were measured with a Varian 400 MHz, Bruker 400 MHz, Varian 600 MHz and Bruker Avance 700 MHz spectrometer at 296 K. Chemical shifts (δ) are reported in parts per million (ppm) relative to the respective residual solvent peaks (CDCl3: *δ* 7.26 in 1H and 77.20 in 13C NMR; D_2O: *δ* 4.79 in 1H NMR).The following abbreviations are used to indicate peak multiplicities: s singlet; d doublet; dd doublet of doublets; t triplet; dt doublet of triplets; q quartet; m multiplet. Coupling constants (J) are reported in Hertz (Hz). Non-edited, multiplicity edited and without broadband decoupling HSQC spectrum and the coupled HSQC spectrum were run with the software supplied with the spectrometers to identify the proton bearing carbons, to distinguish CH_3,CH_2, and CH groups and one bond ^{1}H-^{13}C coupling constants respectively. In HSQC spectrum, methine/methyl resonances have positive intensity and are plotted in red; methylene resonances have negative intensity and are plotted in blue. High resolution mass spectra (HRMS) were obtained using 6210 ESI-TOF mass spectrometer (Agilent) and MALDI-TOF autoflexTM (Bruker) instruments. IR spectra were recorded on a Perkin-Elmer 1600 FTIR spectrometer. Optical rotations were measured by using a Perkin-Elmer 241 and Unipol L1000 polarimter, with concentrations expressed in g per 100 mL.

2. Synthetic Procedures

4-Methoxyphenyl-3,4-di-*O*-benzyl-*α*-L-rhamnopyranoside (8)

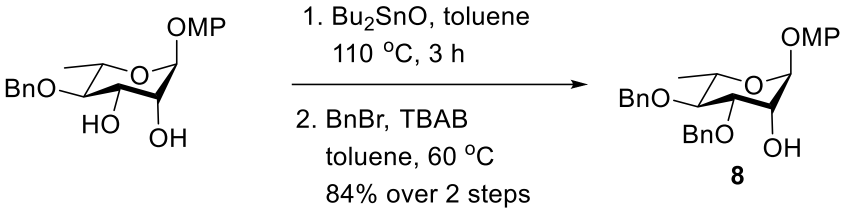

Dibutyltin oxide (0.93 g, 3.75 mmol) was added to a suspension of pent-4-enyl 4-O-benzyl-α-L-rhamnopyranoside (0.9 g, 2.49 mmol) in toluene (20 mL) and the reaction mixture was heated at 110 °C for 3 h. Removal of the solvent from the reaction mixture under reduced pressure gave stannylene acetal that was kept under high vacuum for 2 h. The crude product was dissolved in toluene (15 mL), and was added tetrabutylammonium bromide (1.21 g, 4.99 mmol) and benzyl bromide (0.45 mL, 3.74 mmol) at room temperature under nitrogen atmosphere. The reaction mixture was heated at 60 °C for 6 h. The solids were filtered off from the reaction mixture and the residue was partitioned between ethyl acetate and water. The organic layers were washed with water, brine, dried over Na_2SO_4 and filtered. The filtrate was concentrated *in vacuo* and purified by column chromatography on silica gel (20–50% ethyl acetate / pet ether) to obtain **8** as oil (0.95 g, 84% over 2 steps). Analytical data were essentially the same as reported previously.[1] ^{1}H NMR (400 MHz, $CDCl_3$) δ 7.46 – 7.29 (m, 10H, Ar), 7.02 – 6.92 (m, 2H, Ar), 6.88 – 6.76 (m, 2H, Ar), 5.45 (d, J = 1.8 Hz, 1H, H-1), 4.91 (d, J = 10.9 Hz, 1H, CH*H*Ph), 4.77 (s, 2H, C*H2*Ph), 4.66 (d, J = 10.9 Hz, 1H, C*H*HPh), 4.22 (dd, J = 3.9, 1.9 Hz, 1H, H-2), 4.04 (dd, J = 9.1, 3.4 Hz, 1H, H-3), 3.87 (m, 1H, H-5), 3.77 (s, 3H, OCH3), 3.52 (t, J = 9.4 Hz, 1H, H-4), 2.62 (d, J = 1.7 Hz, 1H, OH), 1.28 (d, J = 6.2 Hz, 3H, CH3); ^{13}C NMR (101 MHz, $CDCl_3$) δ 154.84, 150.13, 138.27, 137.84, 128.63, 128.46, 128.08, 128.01, 127.91, 127.82, 117.55, 114.58, 97.75, 79.89, 79.85, 76.73, 75.52, 72.23, 68.52, 67.96, 55.65, 17.93, 13.55.

Pent-4-enyl 2,4-di-O-benzyl-α-L-rhamnopyranoside (9)

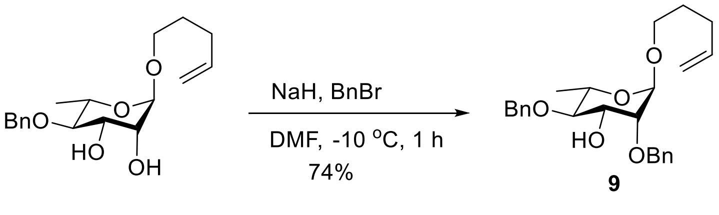

NaH (120 mg, 4.99 mmol) was added to a solution of pent-4-enyl 4-O-benzyl-α-L-rhamnopyranoside (0.9 g, 2.49 mmol) in DMF (15 mL) at 0 °C. The resulting mixture was stirred for 30 min followed by the addition of BnBr (0.29 mL, 2.49 mmol). Further it was stirred overnight at room temperature. After the completion of the reaction it was diluted with ice-water and extracted with EtOAc. The combined organic layer was

washed with ice-water and with brine. The resulting solution was dried over Na_2SO_4, concentrated and purified by silica gel column chromatography (Hexane/EtOAc 12:1) to afford **9** as pale yellow oil (0.83 g, 74%). Analytical data were essentially the same as reported previously.[2] ^{1}H NMR (400 MHz, Chloroform-*d*) δ 7.44 – 7.29 (m, 11H, Ar), 5.81 (ddt, *J* = 16.9, 10.1, 6.6 Hz, 1H, olefine), 5.09 – 4.96 (m, 2H, olefine), 4.92 (d, *J* = 11.0 Hz, 1H, PhCH*H*), 4.83 – 4.73 (m, 2H, PhC*H*H, H-1), 4.68 (d, *J* = 11.1 Hz, 1H, PhCH*H*), 4.61 (d, *J* = 11.8 Hz, 1H, PhCH*H*), 3.97 (td, *J* = 9.3, 3.8 Hz, 1H, H-3), 3.74 (dd, *J* = 3.8, 1.6 Hz, 1H, H-2), 3.68 (dq, *J* = 9.5, 6.5 Hz, 2H, H-5, OC*H*H), 3.44 – 3.25 (m, 2H, CH_2 of pentenyl), 2.33 (d, *J* = 9.3 Hz, 1H, OH), 2.10 (m, , 2H), 1.70 – 1.63 (m, 2H, CH_2 of pentenyl), 1.35 (d, *J* = 6.2 Hz, 3H, CH_3); ^{13}C NMR (101 MHz, $CDCl_3$) *δ* 138.62, 138.14, 137.85, 128.72, 128.56, 128.19, 128.13, 127.87, 115.05, 96.94, 82.47, 78.86, 77.48, 75.28, 73.16, 71.82, 67.22, 66.93, 30.41, 28.73, 18.15.

2,3-Di-*O*-benzyl-4,6-O-benzyledine-*α*-D-glucopyranosyl--1-N-phenyltrifluoroacetamidate (10)

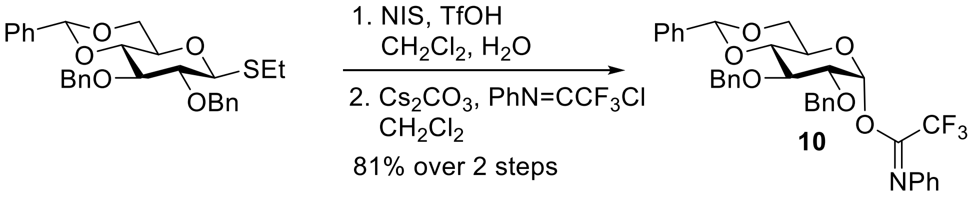

To a solution of thioglycoside (2.3 g, 4.67 mmol) in DCM (50 mL) and water (5 mL) was added NIS (1.05 g, 4.67 mmol) followed by TfOH (21 *μ*L, 0.23 mmol) at 0 ^{o}C. After 1 h, the reaction was quenched with aq. $NaHCO_3$ and extracted with DCM. The organic layers were washed with aq. $NaHCO_3$, brine and dried over Na_2SO_4. The solvents were evaporated *in vacuo* to give crude hemiacetal, which was kept under high vacuum for 2 h and taken for next step.

To a solution of Hemiacetal in DCM (30 mL) was added cesium carbonate (3.04 g, 9.34 mmol) and 2,2,2-trifluoro-N-phenylacetimidoyl chloride (1.36 mL, 8.40 mmol) at 0 ^{o}C. After 1 h, the solids were filtered off and the filtrate was concentrated. The crude residue was purified by column chromatography using 5-10% EtOAc in hexane (1% Et_3N) as eluents to give **10** as sticky solid. (only *α* product, 1.5 g, 81%). Analytical data were essentially the same as reported previously.[3] ^{1}H NMR (400 MHz, $CDCl_3$) *δ* 7.63 – 7.50 (m, 2H, Ar), 7.50 – 7.27 (m, 16H, Ar), 7.15 (t, *J* = 7.4 Hz, 1H, Ar), 6.80 (d, *J* =

7.7 Hz, 2H, Ar), 6.63 – 6.36 (m, 1H, H-1), 5.64 (s, 1H, PhCH), 5.01 (d, *J* = 11.2 Hz, 1H, PhCH*H*), 4.95 – 4.85 (m, 2H), 4.80 (d, *J* = 12.0 Hz, 1H, PhCH*H*), 4.40 (dd, *J* = 10.4, 4.9 Hz, 1H), 4.16 (t, *J* = 9.3 Hz, 1H, H-3), 4.12 – 3.98 (m, 1H), 3.77 (q, *J* = 9.7 Hz, 3H); ^{13}C NMR (101 MHz, $CDCl_3$) δ 143.66, 138.61, 137.93, 137.25, 129.23, 128.93, 128.66, 128.52, 128.46, 128.28, 128.16, 128.10, 127.88, 127.82, 126.18, 124.46, 119.55, 101.52, 81.54, 78.67, 78.34, 77.52, 75.58, 73.99, 68.83, 65.23.

3-*O*-Benzyl-4,6-O-benzyledine-2-O-levulinyl-*α*-D-glucopyranosyl-1-trichloroacetamidate (11)

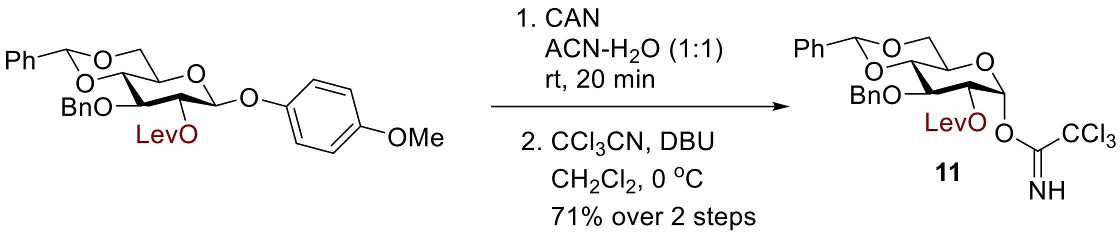

CAN (4.37 g, 7.97 mmol, 2 equiv.) was added to a solution of *p*-methoxy phenyl glycoside (2.24 g, 3.9 mmol) in acetonitrile-water (1:1; 120 mL), and the mixture was stirred for 20 min at rt. The volatile solvent was removed *in vacuo*. The residue was dissolved in CH_2Cl_2, washed with water, dried with Na_2SO_4 and concentrated. The residue was purified by column chromatography (2:1; hexane: ethyl acetate) to give hemiacetal as an *α* isomer. Hemiacetal (1.02 g, 2.23 mmol), trichloroacetonitrile (0.89 mL, 8.90 mmol) were suspended in anhydrous dichloromatane (20 mL) and left stirring at 0 °C for a period of 10 min, at which point DBU (0.067 mL, 0.45 mmol) was added. After 1 h, t.l.c. indicated the formation of a product with complete consumption of the starting material. The volatiles were removed under reduced pressure. The resulting residue was purified by flash column chromatography (hexane : ethyl acetate = 4:1 to 2:1 in 1% Et_3N) to afford *α*-isomer as product to obtain **11** (994 mg, 1.57 mmol, 71%). Analytical data were essentially the same as reported previously.[4] 1 H NMR (400 MHz, $CDCl_3$): *δ* 8.63 (s, 1H, NH), 7.55 – 7.47 (m, 2H, Ar), 7.43 – 7.37 (m, 3H, Ar), 7.36 – 7.28 (m, 5H, Ar), 6.49 (d, J = 3.7 Hz, 1H, H-1), 5.62 (s, 1H, PhCH), 5.10 (dd, J = 9.7, 3.8 Hz, 1H, H-2), 4.93 (d, J = 11.8 Hz, 1H, PhCH*H*), 4.76 (d, J = 11.8 Hz, 1H, PhC*H*H), 4.36 (dd, J = 10.3, 4.9 Hz, 1H, H-3), 4.15 (t, J = 9.5 Hz, 1H, H-4), 4.06 (m, 1H, H-5), 3.81 (m, 2H, H-6), 2.69 (td, J = 6.7, 3.1 Hz, 2H, CH_2 of Lev), 2.51 (td, J = 7.2, 6.6, 2.1 Hz, 2H, CH_2 of Lev), 2.16 (s, 3H, CH_3 of Lev). 13 C NMR (100 MHz, $CDCl_3$): *δ* 206.0, 171.0, 161.1, 138.0, 129.1, 128.4, 128.3, 128.0, 127.8, 126.1, 101.4, 96.1, 80.9, 78.2, 74.0, 71.0, 68.5, 66.9, 37.6, 29.9, 27.7.

4-Methoxyphenyl-(2,3-di-O-benzyl-4,6-O-benzylidene-α-D-glucopyranosyl)-(1→2)-3,4-di-O-benzyl-α-L-rhamnopyranoside (12)

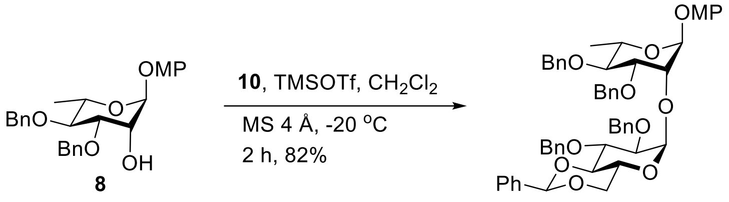

A solution of **8** (0.9 g, 1.99 mmol), imidate **10** (2.01 g, 3.39 mmol)) and 4 Å molecular sieves (2.5 g) in anhydrous CH_2Cl_2 (40 mL) was cooled to -20 °C, then TMSOTf (36.1 *μ*L, 0.19 mmol) was added dropwise. The reaction mixture was gradually brought to room temperature over 2 h. After complete consumption of starting material, Et_3N (2 mL) was added and evaporated the reaction mixture to dryness. The crude product was purified by silica gel column chromatography (Hexane/EtOAc 6:1) to give **12** as a sticky liquid (1.45 g, 82%). $[\alpha]_D^{25}$ = +12.82 (c = 1.68, $CHCl_3$); IR ν_{max} (film) 3460, 3034, 2934, 1724, 1603, 1507, 1455, 1365, 1216, 1091, 1029, 918, 826, 751, 698 cm^{-1}; ^1H NMR (400 MHz, $CDCl_3$) δ 7.49 – 7.43 (m, 2H, Ar), 7.39 – 7.30 (m, 9H, Ar), 7.29 – 7.27 (m, 2H, Ar), 7.25 – 7.18 (m, 6H, Ar), 6.89 – 6.84 (m, 2H, Ar), 6.78 – 6.73 (m, 2H, Ar), 5.51 (s, 1H, PhCH), 5.36 (d, *J* = 2.0 Hz, 1H, H-1), 4.95 – 4.86 (m, 3H, $PhCH_2$), 4.84 – 4.77 (m, 3H, $PhCH_2$), 4.75 – 4.58 (m, 4H, $PhCH_2$, H-1´(merged)), 4.21 (td, *J* = 10.0, 4.9 Hz, 1H, H-5), 4.13 – 4.06 (m, 2H), 3.99 (m, 2H), 3.83 – 3.77 (m, 1H), 3.74 (s, 3H), 3.64 – 3.53 (m, 4H), 1.26 (d, *J* = 6.3 Hz, 3H, CH_3); ^{13}C NMR (101 MHz, $CDCl_3$) δ 154.99, 150.56, 139.06, 138.67, 138.59, 138.49, 137.76, 129.02, 128.59, 128.53, 128.46, 128.35, 128.30, 128.15, 128.08, 127.90, 127.82, 127.79, 127.74, 127.69, 126.28, 117.74, 114.74, 101.36, 98.52, 96.81, 82.62, 80.19, 79.66, 78.83, 78.47, 75.59, 75.26, 73.34, 72.47, 69.21, 69.02, 63.04, 55.85, 18.25; HRMS (ESI): Calcd for $C_{54}H_{56}O_{11}$ $[M+Na]^+$ 903.3720, found: 903.3710.

4-Methoxyphenyl-(2,3,6-tri-O-benzyl-α-D-glucopyranosyl)-(1→2)-3,4-di-*O*-benzyl-*α*-L-rhamnopyranoside (13)

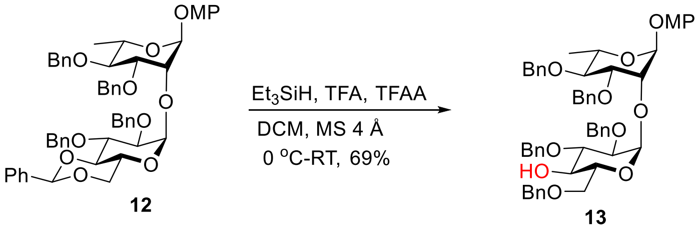

To a mixture of **12** (1.3 g, 1.48 mmol), MS 4 Å (2.0 g) in anhydrous CH_2Cl_2 (25 mL) were added triflouroacetic anhydride (107.0 *μ*L, 0.74 mmol) and Et_3SiH (1.42 mL, 8.85

mmol) at 0 °C, slowly. After stirring the reaction mixture for 10 min, TFA (0.57 mL, 7.38 mmol) was added dropwise at 0 °C. The reaction mixture was gradually brought to room temperature over 2 h. The reaction was quenched with acetone (2 mL) and the solids were filtered off. The mixture was poured into saturated aqueous $NaHCO_3$ and extracted with DCM (3x20 mL). The combined organic extracts were washed with water, brine (50 mL), dried over Na_2SO_4, filtered and concentrated. The crude was purified by flash chromatography using hexane and ethyl acetate as eluent (3:1) to give the desired product **13** as a pale yellow oil (0.9 g, 69%). $[\alpha]_D^{25}$ = +3.8 (c = 1.11, $CHCl_3$); IR ν_{max} (film) 3475, 3065, 3032, 2925, 1729, 1604, 1507, 1455, 1364, 1289, 1216, 1099, 1061, 1038, 1029, 987, 913, 827, 735, 698 cm^{-1}; ^{1}H NMR (400 MHz, $CDCl_3$) δ 7.32 – 7.11 (m, 24H, Ar), 6.82 (d, J = 9.1 Hz, 2H, Ar), 6.69 (d, J = 9.1 Hz, 2H, Ar), 5.29 (d, J = 1.8 Hz, 1H, H-1), 4.92 – 4.86 (m, 2H, PhC*H*H, H-1´), 4.82 (d, J = 10.9 Hz, 1H, PhCH*H*), 4.74 – 4.63 (m, 3H, $PhCH_2$), 4.61 (d, J = 3.2 Hz, 1H, $PhCH_2$), 4.54 (dd, J = 11.3, 5.7 Hz, 2H, $PhCH_2$), 4.36 (d, J = 12.2 Hz, 1H, $PhCH_2$), 4.28 (d, J = 12.1 Hz, 1H, $PhCH_2$), 4.14 – 4.09 (m, 1H, H-2), 4.03 (dt, J = 9.7, 3.5 Hz, 1H), 3.96 (dd, J = 9.1, 3.1 Hz, 1H), 3.84 – 3.72 (m, 2H), 3.64 (s, 3H, OCH_3), 3.61 – 3.51 (m, 2H), 3.48 (dd, J = 9.5, 3.5 Hz, 1H), 3.37 (dd, J = 10.4, 3.9 Hz, 1H), 3.29 (dd, J = 10.5, 3.3 Hz, 1H, H-2´), 1.22 (d, J = 6.2 Hz, 3H, CH_3). ^{13}C NMR (101 MHz, $CDCl_3$) *δ* 154.90, 150.50, 138.94, 138.55, 138.49, 138.40, 138.05, 128.52, 128.46, 128.42, 128.41, 128.37, 128.07, 128.06, 127.86, 127.76, 127.71, 127.69, 127.65, 127.48, 127.45, 117.74, 114.62, 96.82, 96.62, 80.89, 80.21, 79.80, 78.68, 75.20, 75.13, 75.10, 74.36, 73.57, 72.60, 72.03, 70.83, 70.47, 69.26, 68.89, 55.67, 18.19; HRMS (ESI): Calcd for $C_{54}H_{58}O_{11}$ $[M+Na]^+$ 905.3876, found: 905.3880.

4-Methoxyphenyl (3-O-benzyl-4,6-O-benzylidene-2-levulinoyl-β-D-glucopyranosyl)-(1→4)-(2,3,6-tri-O-benzyl-α-D-glucopyranosyl)-(1→2)-3,4-di-O-benzyl-α-L-rhamnopyranoside (14)

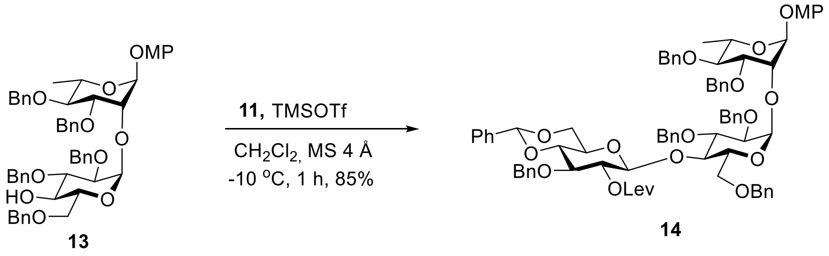

To a solution of **13** (0.32 g, 0.54 mmol), imidate **11** (0.64 g, 1.09 mmol)) and 4 Å molecular sieves (1.0 g) in anhydrous CH_2Cl_2 (15 mL) was cooled to -10 °C, then TMSOTf (19.7 *µ*L, 0.11 mmol) was added dropwise. After 1 h, the reaction was

quenched Et_3N (1 mL) and then the solids were filtered off. The filtrate was evaporated *in vacuo* to dryness. The crude product was purified by silica gel (100-200) column chromatography (Hexane/EtOAc 5:1) to give **14** as a foam (0.61 g, 85%). $[\alpha]_D^{25}$ = -17.54 (c = 0.55, $CHCl_3$); IR ν_{max} (film) 3032, 2926, 1751, 1720, 1507, 1455, 1365, 1313, 1261, 1215, 1178, 1096, 1028, 913, 826, 802, 749, 697, 665 cm^{-1}; ^{1}H NMR (400 MHz, $CDCl_3$) δ 7.48 (m, 3H, Ar), 7.44 – 7.28 (m, 31H, Ar), 7.23 (m, 5H, Ar), 6.91 – 6.84 (m, 2H, Ar), 6.81 – 6.75 (m, 2H, Ar), 5.47 (s, 1H, PhCH), 5.40 (d, *J* = 2.0 Hz, 1H, H-1), 5.01 – 4.85 (m, 5H, $PhCH_2$, H-1´, H-2´´), 4.80 (m, 5H, $PhCH_2$), 4.71 – 4.55 (m, 6H, $PhCH_2$), 4.34 (d, *J* = 8.0 Hz, 1H, H-1´´), 4.21 – 4.10 (m, 3H), 4.03 (ddd, *J* = 7.9, 6.0, 2.5 Hz, 2H), 3.95 – 3.85 (m, 3H), 3.77 (s, 5H), 3.66 – 3.57 (m, 3H), 3.56 – 3.48 (m, 1H), 3.46 – 3.34 (m, 2H), 3.22 (dd, *J* = 10.8, 1.8 Hz, 1H), 3.09 (td, *J* = 9.7, 4.9 Hz, 1H), 2.57 – 2.42 (m, 1H, C*H*H of Lev), 2.42 – 2.29 (m, 2H, CH_2 of Lev), 2.23 – 2.14 (m, 1H, CH*H* of Lev), 1.92 (s, 3H, CH3 of Lev), 1.31 (d, *J* = 6.2 Hz, 3H, CH_3); ^{13}C NMR (101 MHz, $CDCl_3$) δ 205.96, 171.34, 154.88, 150.44, 139.56, 138.89, 138.78, 138.58, 138.50, 137.84, 137.44, 129.21, 128.76, 128.69, 128.62, 128.58, 128.49, 128.46, 128.44, 128.30, 128.27, 128.18, 127.99, 127.92, 127.86, 127.78, 127.74, 127.70, 127.60, 127.41, 126.20, 117.64, 114.67, 101.30, 100.69, 98.63, 96.94, 81.79, 80.16, 79.65, 79.29, 79.03, 78.66, 76.25, 75.38, 75.27, 74.15, 73.80, 73.72, 73.26, 71.88, 70.70, 68.94, 68.80, 67.28, 66.00, 55.83, 37.71, 29.79, 27.84, 18.19; HRMS (ESI): Calcd for $C_{79}H_{84}O_{18}$ $[M+Na]^+$ 1343.5555, found: 1343.5573.

4-Methoxyphenyl-(3-O-benzyl-4,6-*O*-benzylidene-*β*-D-glucopyranosyl)-(1→4)-(2,3,6-tri-*O*-benzyl-*α*-D-glucopyranosyl)-(1→2)-3,4-di-O-benzyl-*α*-L-rhamnopyranoside (15)

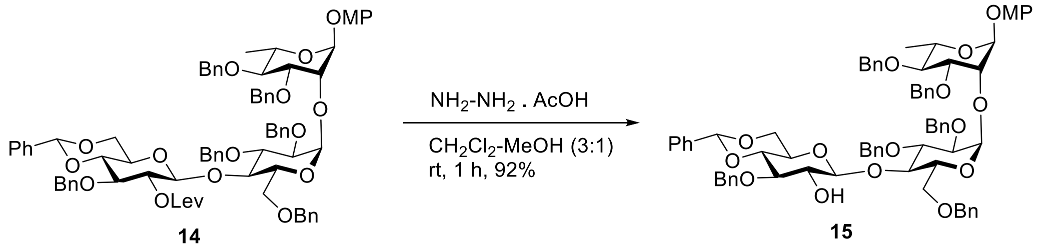

14 **15**

Hydrazine acetate (75.3 mg, 0.81 mmol) dissolved in MeOH (6 mL), was added to a solution of **14** (0.6 g, 0.45 mmol) in DCM (18 mL). The reaction mixture was stirred for 1 h and afterwards quenched by the addition of acetone. Then it was evaporated to dryness and purified by silica gel column chromatography (Hexane/EtOAc 4:1) to obtain **15** as foam (0.51 g, 92%). $\alpha]_D^{25}$ = -9.11 (c = 0.33, $CHCl_3$); IR ν_{max} (film) 3030, 2922, 1509, 1451, 1365, 1315, 1260, 1215, 1174, 1095, 1021, 913, 826, 803, 747,

697, 663 cm^{-1}; ^{1}H NMR (400 MHz, $CDCl_3$) δ 7.48 – 7.44 (m, 2H, Ar), 7.41 – 7.27 (m, 29H, Ar), 7.25 – 7.22 (m, 3H, Ar), 6.94 – 6.87 (m, 2H, Ar), 6.84 – 6.76 (m, 2H, Ar), 5.47 (s, 1H, PhCH), 5.36 (d, *J* = 1.9 Hz, 1H, H-1), 4.99 – 4.86 (m, 5H, $PhCH_2$, H-1´), 4.82 – 4.65 (m, 5H, $PhCH_2$), 4.62 – 4.49 (m, 3H, $PhCH_2$, H-1´´(merged)), 4.32 (d, *J* = 11.9 Hz, 1H, PhCH*H*), 4.20 – 4.11 (m, 2H), 4.04 (dd, *J* = 9.3, 3.2 Hz, 1H), 4.01 – 3.92 (m, 3H), 3.84 (dq, *J* = 9.6, 6.1 Hz, 1H), 3.78 (s, 3H), 3.70 – 3.60 (m, 2H), 3.60 – 3.49 (m, 3H), 3.47 – 3.39 (m, 2H), 3.25 (dd, *J* = 11.4, 1.9 Hz, 1H), 3.06 (m, 2H), 1.33 (d, *J* = 6.2 Hz, 3H, CH_3); ^{13}C NMR (101 MHz, $CDCl_3$) δ 154.81, 150.33, 139.24, 138.61, 138.55, 138.38, 137.46, 137.35, 128.97, 128.45, 128.43, 128.38, 128.25, 128.22, 128.03, 127.96, 127.86, 127.73, 127.70, 127.67, 127.54, 127.33, 127.26, 127.05, 126.06, 117.60, 114.54, 103.80, 101.14, 97.27, 96.53, 77.36, 76.72, 73.60, 73.02, 72.11, 69.99, 68.79, 68.68, 68.03, 66.27, 55.67, 18.05; HRMS (ESI): Calcd for $C_{74}H_{78}O_{16}$ $[M+Na]^+$ 1245.5187, found: 1245.5187.

4-Methoxyphenyl-(2-azido-3-*O*-benzyl-4,6-*O*-benzylidene-*β*-D-mannopyranosyl)-(1→4)-(2,3,6-tri-O-benzyl-*α*-D-glucopyranosyl)-(1→2)-3,4-di-*O*-benzyl-L-rhamnopyranoside (16)

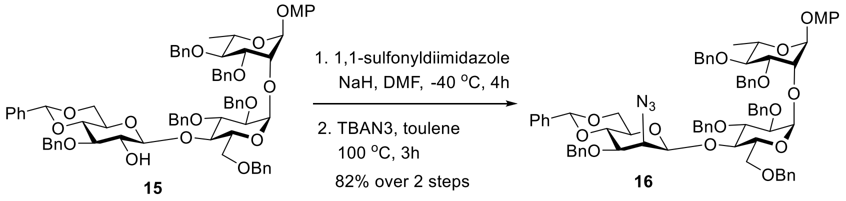

To a solution of **15** (0.28 g, 0.23 mmol) in DMF (17 mL) was added NaH (60%, 33.0 mg, 1.37 mmol) at 0 °C and then the reaction was brought to rt over 1 h. The reaction was cooled to -40 °C, to this 1,1′-sulfonyldiimidazole (68.1 mg, 0.34 mmol) was added. The reaction was stirried at the same temperature for 4 h before quenching with methanol (2 mL). The reaction mixture was partitioned between diethyl ether (3 x 10 mL) and water (10 mL). The combined organic layers were washed with water, brine (50 mL), dried over Na_2SO_4, filtered and concentrated. The crude product was directly taken for the next step without purification.

The sulfonyl derivative was dissolved in anhydrous toluene (7 mL) and then tetrabutylammonium azide (143.7 mg, 0.58 mmol) was added. The reaction was heated at 110 °C for 3 h. The reaction mixture was diluted with water and extracted with ethyl acetate (2 x 15 mL). The combined organic extracts were washed with water, brine (10 mL), dried over Na_2SO_4, filtered and concentrated. The crude was purified by

flash chromatography using hexane and ethyl acetate as eluent (4:1) to give the desired product **16** (234 mg, 82% over 2 steps). $[\alpha]_D^{25}$ = -70.48 (c = 0.10, $CHCl_3$); IR ν_{max} (film) 3033, 2921, 2191, 1515, 1457, 1362, 1313, 1262, 1210, 1172, 1097, 1022, 916, 823, 801, 747, 695, 662 cm^{-1}; 1H NMR (400 MHz, $CDCl_3$) δ 7.38 (Ar, 2H, Ar), 7.35 – 7.27 (m, 12H, Ar), 7.25 – 7.09 (m, 19H, Ar), 6.87 – 6.81 (m, 2H, Ar), 6.73 (d, J = 9.1 Hz, 2H, Ar), 5.42 (s, 1H, PhCH), 5.27 (d, J = 1.9 Hz, 1H, H-1), 4.90 (m, 2H, $PhCH_2$, H-1´), 4.83 – 4.77 (m, 2H, $PhCH_2$), 4.72 – 4.64 (m, 5H, $PhCH_2$), 4.62 (m, 1H, PhCH*H*), 4.57 (m, 1H, PhCH*H*), 4.54 (m, 1H, PhCH*H*), 4.50 (m, 1H, PhCH*H*), 4.46 (s, 1H, PhCH*H*), 4.36 (d, J = 1.4 Hz, 1H, H-1´´(manno)), 4.12 – 4.01 (m, 4H), 4.00 – 3.94 (m, 2H), 3.94 – 3.87 (m, 2H), 3.86 – 3.74 (m, 4H), 3.70 (s, 3H), 3.60 – 3.53 (m, 2H), 3.52 – 3.42 (m, 3H), 3.27 (dd, J = 9.6, 3.7 Hz, 1H), 3.14 (dd, J = 11.2, 2.4 Hz, 1H, H-6´), 3.06 (dd, J = 11.2, 2.0 Hz, 1H, H-6), 2.88 (td, J = 9.7, 4.9 Hz, 1H, H-5), 1.26 (d, J = 6.2 Hz, 3H, CH_3)., ^{13}C NMR (101 MHz, $CDCl_3$) δ 155.08, 150.57, 139.45, 138.85, 138.67, 138.64, 138.23, 137.76, 137.59, 129.13, 128.71, 128.63, 128.56, 128.51, 128.43, 128.39, 128.34, 128.29, 128.18, 128.09, 127.96, 127.85, 127.78, 127.56, 127.51, 127.25, 126.23, 117.85, 114.78, 101.67, 100.54, 97.55, 96.90, 80.42, 80.16, 79.66, 79.11, 78.67, 77.52, 76.66, 75.55, 75.29, 75.22, 73.68, 73.18, 72.79, 72.40, 69.89, 69.10, 68.59, 68.10, 67.31, 63.72, 55.85, 18.20.HRMS (ESI): Calcd for $C_{74}H_{77}N_3O_{15}$ $[M+K]^+$ 1286.4986, found: 1286.5102.

4-Methoxyphenyl-(2-acetamido-3-*O*-benzyl-4,6-*O*-benzylidene-*β*-D-mannopyranosyl)-(1→4)-(2,3,6-tri-O-benzyl-*α*-D-glucopyranosyl)-(1→2)-3,4-di-*O*-benzyl-L-rhamnopyranoside (17)

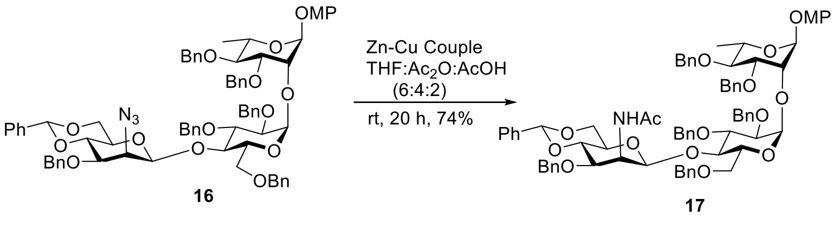

To a solution of **16** (0.21 g, 165.2 *μ*mol) in THF : Ac_2O : AcOH (6.7 mL, 6 : 4 : 2) was added freshly activated Zn-Cu couple (0.72 g) at room temperature. The reaction was stirred at rt for 20 h. The reaction mixture was diluted with EtOAc and the solids were filtered off. The filtrate was concentrated *in vacuo* and the resulting residue was purified by flash chromatography using hexane and ethyl acetate as eluent (3:2) to give the desired product **17** (156 mg, 74%). $[\alpha]_D^{25}$ = -16.70 (c = 0.71, $CHCl_3$); IR ν_{max} (film) 3032,

2926, 2856, 1680, 1507, 1455, 1367, 1216, 1180, 1101, 1039, 1029, 913, 827, 804, 735, 697 cm^{-1}; ^{1}H NMR (400 MHz, $CDCl_3$) 7.52 – 7.47 (m, 2H, Ar), 7.44 – 7.27 (m, 28H, Ar), 7.25 – 7.20 (m, 8H, Ar), 6.94 – 6.88 (m, 2H, Ar), 6.83 – 6.77 (m, 2H, Ar), 5.50 (m, 2H, PhCH, NH), 5.37 (d, *J* = 1.9 Hz, 1H, H-1), 5.00 (dd, *J* = 11.4 Hz, 2H, $PhCH_2$), 4.92 (d, *J* = 3.7 Hz, 1H, H-1'), 4.82 (d, *J* = 12.2 Hz, 1H, PhCH*H*), 4.78 – 4.68 (m, 5H, $PhCH_2$), 4.63 – 4.54 (m, 3H, $PhCH_2$), 4.47 (m, 1H, PhCH*H*), 4.43 (s, 1H, H-1''), 4.23 (d, *J* = 12.0 Hz, 1H, H-6'), 4.15 (dd, *J* = 3.3, 2.0 Hz, 1H), 4.14 – 3.96 (m, 5H), 3.91 – 3.81 (m, 2H), 3.78 (s, 3H, OCH_3), 3.64 (m, 2H), 3.60 – 3.48 (m, 2H), 3.32 (m, 2H), 3.18 (dd, *J* = 11.0, 1H, H-6''), 3.05 (td, *J* = 9.7, 4.8 Hz, 1H), 1.74 (s, 3H, $NHCOCH_3$), 1.35 (d, *J* = 6.2 Hz, 3H, CH_3); ^{13}C NMR (101 MHz, $CDCl_3$) δ 170.66, 154.97, 150.48, 139.72, 138.71, 138.55, 138.49, 138.29, 137.71, 137.47, 129.20, 128.75, 128.67, 128.61, 128.57, 128.53, 128.49, 128.45, 128.41, 128.34, 128.27, 128.22, 127.98, 127.88, 127.84, 127.79, 127.77, 127.68, 127.52, 126.47, 126.25, 117.76, 114.74, 114.70, 101.82, 99.69, 97.78, 96.86, 80.66, 80.28, 79.79, 78.89, 78.64, 73.57, 73.14, 72.28, 71.23, 70.02, 68.95, 68.84, 67.92, 67.27, 55.83, 50.64, 32.12, 29.89, 29.56, 23.38, 22.89, 18.24, 14.34; HRMS (ESI): Calcd for $C_{76}H_{81}NO_{16}$ $[M+Na]^+$ 1286.5447, found: 1286.5422.

2-Acetamido-3-*O*-benzyl-4,6-*O*-benzylidene-β-D-mannopyranosyl-(1→4)-(2,3,6-tri-*O*-benzyl-α-D-glucopyranosyl)-(1→2)-3,4-di-*O*-benzyl-L-rhamnopyranose (18)

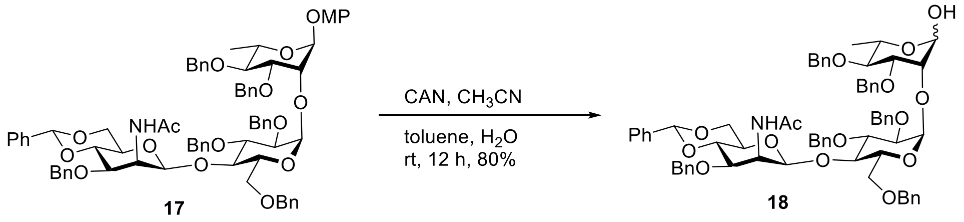

CAN (0.22 g, 0.40 mmol) dissolved in water (11 ml) was added to a solution of **17** (102 mg, 0.081 mmol) in a mixture of acetonitrile (15 mL) and toluene (11 mL) at 0 °C. The reaction mixture was stirred at rt for 12 h. After completion of the reaction, the reaction was quenched with aq. $NaHCO_3$ and extracted with EtOAc (3x 15 mL). The organic layer was washed with brine, dried over $MgSO_4$ and evaporated to dryness. The resulting crude product was purified by silica gel column chromatography (Hexane/EtOAc 2:1) to afford **18** as glassy solid as anomeric mixture (α : β =1:3, 74 mg, 80%). $[\alpha]_D^{25}$ = +5.08 (c = 1.07, $CHCl_3$); IR ν_{max} (film) 3409, 3065, 3032, 2925, 2858, 1960, 1661, 1498, 1455, 1367, 1330, 1312, 1293, 1262, 1213, 1178, 1079, 1044, 1027,

909, 802, 750, 736, 697, 666 cm^{-1}; ^{1}H NMR (700 MHz, $CDCl_3$) δ 7.42 (m, 3H, Ar), 7.39 – 7.27 (m, 21H, Ar), 7.24 – 7.08 (m, 22H, Ar), 5.41 (s, 2H, PhCH), 5.34 (d, J = 9.6 Hz, 1H, NH), 4.95 (d, J = 10.8 Hz, 1H, PhCH*H*), 4.88 (m, 2H, $PhCH_2$), 4.82 (d, J = 11.8 Hz, 1H), 4.74 (m, 1H), 4.72 – 4.66 (m, 4H), 4.61 (m, 5H), 4.53 (m, 2H), 4.49 – 4.39 (m, 3H), 4.38 (t, J = 11.8 Hz, 1H), 4.32 (s, 1H), 4.07 (dd, J = 10.4, 4.9 Hz, 1H), 4.03 – 3.75 (m, 8H), 3.59 – 3.43 (m, 5H), 3.42 – 3.35 (m, 2H), 3.33 – 3.29 (m, 2H), 3.28 – 3.20 (m, 1H), 3.17 (dd, J = 9.9, 4.3 Hz, 1H), 2.97 (td, J = 9.7, 4.8 Hz, 1H), 2.93 (d, J = 11.0 Hz, 1H), 1.70 (s, 3H), 1.69 (s, 3H), 1.35 (d, J = 4.5 Hz, 3H), 1.28 (d, J = 6.2 Hz, 1H); ^{13}C NMR (176 MHz, $CDCl_3$) δ 170.50, 170.43, 139.32, 138.56, 138.41, 138.37, 138.30, 138.16, 138.12, 137.64, 137.42, 137.36, 137.32, 137.00, 129.05, 128.72, 128.67, 128.64, 128.58, 128.52, 128.48, 128.43, 128.41, 128.38, 128.35, 128.30, 128.28, 128.24, 128.23, 128.07, 127.97, 127.84, 127.75, 127.72, 127.70, 127.66, 127.59, 127.54, 127.46, 127.36, 126.37, 126.22, 126.10, 102.52, 101.71, 101.65, 99.65, 99.31, 93.96, 93.87, 92.40, 81.51, 81.49, 81.28, 81.15, 80.41, 80.14, 79.78, 79.51, 78.71, 78.54, 78.48, 78.41, 75.88, 75.79, 75.52, 75.33, 74.89, 74.46, 73.42, 73.39, 72.64, 72.15, 71.64, 71.44, 71.15, 71.03, 70.44, 68.64, 68.48, 67.42, 67.16, 50.49, 50.41, 31.95, 29.72, 29.38, 23.25, 23.21, 22.71, 18.14, 18.09, 14.15; HRMS (ESI): Calcd for $C_{69}H_{75}NO_{15}$ [M+Na]$^+$ 1180.5034, found: 1180.5028.

(2-Acetamido-3-*O*-benzyl-4,6-*O*-benzylidene-β-D-mannopyranosyl-(1→4)-(2,3,6-tri-*O*-benzyl-α-D-glucopyranosyl)-(1→2)-3,4-di-*O*-benzyl-L-rhamnopyranosyl hydrogen phosphonate triethylammonium salt (6)

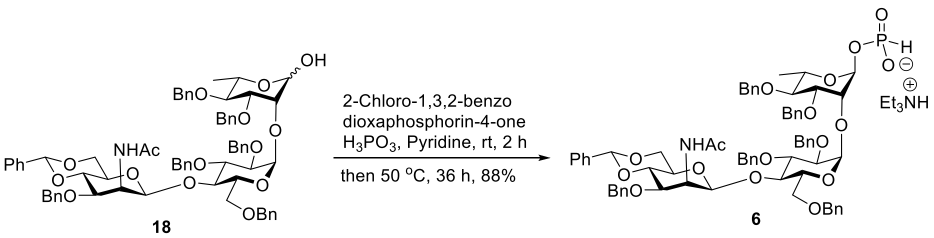

Compound **18** (20 mg, 17.26 μmol) and phosphorous acid (4.2 mg, 51.79 μmol) were mixed and co-evaporated with pyridine (3 x 2.0 mL). The dried reaction mixture was kept under high vacuum overnight. To a solution premixed compound **18** and H_3PO_3 in anhydrous pyridine (1.5 mL) was added 2-chloro-5,5-dimethyl-2-oxo-1,3,2-dioxaphosphorinane (5.3 mg g, 25.89 μmol) at 0 °C was then after 10 min, the ice bath was removed and the reaction mixture was stirred for 2 h at rt. The reaction mixture was heated to 50 °C, and stirring was continued for 36 h. Then, 1 M aq.

triethylammonium hydrogen carbonate (2 mL) was added to destroy excess of reagent, and the mixture was diluted with dichloromethane, washed with 0.5 M aq. triethylammonium hydrogen carbonate (TEAB), dried (Na_2SO_4), filtered and concentrated. The residue was purified by column chromatography (ethyl acetate–hexane (1:1 to 1:0) then dichloromethane–methanol (20:1) together with 1% trimethylamine as eluents. The product fractions were evaporated then was dissolved in dichloromethane and washed with 0.25 M aq. triethylammonium hydrogen carbonate. The organic layer was separated, concentrated *in vacuo* to give H-phosphonate **6** as foam (20 mg, 88%). ^{1}H NMR (700 MHz, $CDCl_3$) δ 12.12 (s, 1H, NH), 7.41 (d, J = 8.2 Hz, 3H, Ar), 7.35 – 7.27 (m, 15H, Ar), 7.23 – 7.08 (m, 20H, Ar), 5.60 (d, $^3J_{P,H}$ = 7.5 Hz, 1H, H-1), 5.41 (m, 2H, NH, PhCH), 4.96 (m, 1H, H-1'), 4.90 (dd, J = 11.4 Hz, 2H, $PhCH_2$), 4.70 (d, J = 11.8 Hz, 1H, $PhCH_2$), 4.61 (m, 6H, $PhCH_2$), 4.50 (m, 4H, $PhCH_2$, H-1''), 4.45 – 4.36 (m, 2H, $PhCH_2$), 4.17 (d, J = 12.1 Hz, 1H, PhCH*H*), 4.08 (s, 1H), 4.03 – 3.97 (m, 2H), 3.92 (m, 4H), 3.77 (t, J = 9.3 Hz, 1H), 3.55 (t, J = 9.9 Hz, 3H), 3.51 – 3.42 (m, 3H), 3.23 (m, 1H), 3.19 (m, 1H), 3.09 (d, J = 11.0 Hz, 1H), 2.94 (p, J = 9.5, 8.1 Hz, 9H, $N\underline{(CH_2)_3}CH_3)_3$), 1.66 (s, 3H), 1.26 (d, J = 6.3 Hz, 3H, CH_3), 1.21 – 1.18 (m, 14H, $N(CH_2)_3\underline{(CH_3)_3}$); ^{13}C NMR (101 MHz, $CDCl_3$) δ 170.59, 139.76, 138.76, 138.45, 138.26, 137.99, 137.76, 137.44, 135.11, 130.77, 129.15, 128.64, 128.54, 128.46, 128.41, 128.34, 128.29, 128.14, 127.98, 127.75, 127.68, 127.63, 127.42, 127.27, 126.39, 126.20, 125.41, 118.88, 117.21, 101.72, 99.62, 96.55, 93.27, 80.50, 80.05, 79.32, 78.57, 75.98, 75.35, 75.16, 74.73, 73.45, 72.33, 72.15, 71.17, 69.68, 69.54, 68.77, 67.92, 67.17, 50.58, 45.56, 29.82, 23.29, 21.59, 18.10, 8.61; ^{31}P NMR (162 MHz, $CDCl_3$): δ_P 0.91, $^1J_{H\text{-}P}$ = 641.81 Hz & $^3J_{H1\text{-}P}$ = 8.2 Hz; HRMS (ESI): Calcd for $C_{75}H_{91}N_2O_{17}P$ $[M\text{-}Et_3N\text{+}H]^+$ 1222.4923, found: $[M\text{-}Et_3NH\text{+}H]^+$ 1222.4873.

5-Azidopentyl-1-*O*-(2-acetamido-3-*O*-benzyl-4,6-*O*-benzylidene-β-D-mannopyranosyl-(1→4)-(2,3,6-tri-*O*-benzyl-α-D-glucopyranosyl)-(1→2)-3,4-di-*O*-benzyl-L-rhamnopyranosyl phosphate) triethylammonium salt (19)

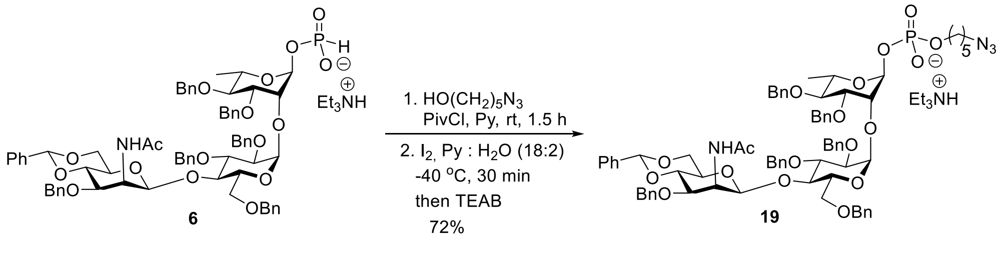

H-phosphonate **6** (5 mg, 4.09 μmol) and C5 azido linker (2.1 mg, 20.45 μmol) were mixed and co-evaporated with pyridine (3 x 1.0 mL). The dried reaction mixture was kept under high vacuum for 30 min. The reaction mixture was dissolved in anhydrous pyridine (1.0 mL) and then pivaloyl chloride (1.5 μL, 12.27 μmol) was added. The reaction was stirred at rt for 2 h. Then, the reaction was cooled to -40 ^{o}C, a freshly prepared solution of Iodine (3.2 mg, 13.22 μmol) in pyridine-water (19 : 1, 0.1 mL) was added. After 30 min the mixture was diluted with dichloromethane, washed successively with 10% aq. sodium thiosulfate, 0.5 M aq. triethylammonium bicarbonate (TEAB), dried over Na_2SO_4, filtered and concentrated. The residue was purified by column chromatography (ethyl acetate–hexane (1:1 to 1:0) then dichloromethane–methanol (25:1) together with 1% trimethylamine as eluents. The product fractions were evaporated then was dissolved in dichloromethane and washed with 0.25 M aq. triethylammonium bicarbonate. The organic layer was separated, concentrated and co-concentrated from dichloromethane (5 × 10 mL) to afford **19** as foam (4 mg, 72% over two steps). ^{1}H NMR (600 MHz, $CDCl_3$) δ 12.02 (s, 2H, NH), 7.41 (d, *J* = 7.3 Hz, 2H, Ar), 7.28 (m, 15H, Ar), 7.22 – 7.12 (m, 10H, Ar), 7.10 (d, *J* = 7.5 Hz, 1H, Ar), 7.02 (dd, *J* = 7.3, 3.8 Hz, 1H, Ar), 5.54 (d,$^{3}J_{P.H-1}$ = 8.1 Hz, 1H, H-1), 5.42 (d, *J* = 9.2 Hz, 1H, NH), 5.40 (s, 1H, PhCH), 4.99 (d, *J* = 4.1 Hz, 1H, H-1’), 4.90 (m, 2H), 4.70 (d, *J* = 11.7 Hz, 1H, PhCH*H*), 4.68 – 4.58 (m, 4H, $PhCH_2$), 4.50 (m, 3H, $PhCH_2$), 4.41 (m, 2H, $PhCH_2$, H-1’’), 4.17 (d, *J* = 12.0 Hz, 1H), 4.10 (s, 1H), 4.06 – 3.97 (m, 2H), 3.91 (q, *J* = 9.5, 8.8 Hz, 3H), 3.85 – 3.74 (m, 3H), 3.56 (m, 3H), 3.51 – 3.41 (m, 2H), 3.28 – 3.18 (m, 2H), 3.17 – 3.05 (m, 4H), 3.01 – 2.90 (m, 7H, $N(\underline{CH_2})_3(CH_3)_3$), 1.66 (s, 3H, $NHCOCH_3$), 1.54 (q, *J* = 9.3, 8.2 Hz, 2H), 1.49 (t, *J* = 7.5 Hz, 2H), 1.26 (d, *J* = 6.5 Hz, 3H, CH_3), 1.23 (t, *J* = 7.3 Hz, 11H, $N(CH_2)_3(\underline{CH_3})_3$); ^{13}C NMR (151 MHz, $CDCl_3$) δ 170.57, 139.80, 138.83, 138.60, 138.34, 137.89, 137.54, 129.21, 129.14, 128.70, 128.61, 128.51, 128.48, 128.42, 128.36, 128.29, 128.15, 128.08, 127.82, 127.73, 127.63, 127.50, 127.43, 127.35, 126.51, 126.26, 125.47, 101.79, 99.81, 96.66, 94.37, 80.53, 80.09, 79.42, 78.70, 78.64, 75.62, 75.24, 74.76, 73.52, 72.32, 72.21, 71.31, 70.75, 69.81, 69.50, 68.84, 68.13, 67.28, 65.88, 51.51, 50.70, 45.81, 45.53, 32.11, 30.35, 30.22, 29.88, 29.54, 28.73, 27.89, 23.36, 23.20, 22.88, 21.68, 18.26, 14.30, 8.72; ^{31}P NMR (162 MHz, $CDCl_3$) δ_P -2.44, $^{3}J_{H1\text{-}P}$ = 8.1 Hz; HRMS (ESI): Calcd for $C_{74}H_{85}N_4O_{18}P$ $[M\text{-}Et_3N+H]^+$ 1349.5669, found: 1349.5752.

5-Aminopentyl-1-*O*-(2-acetamido-2-deoxy-*β*-D-mannopyranosyl-(1→4)-(α-D-glucopyranosyl)-(1→2)-L-rhamnopyranosyl phosphate) sodium salt (3)

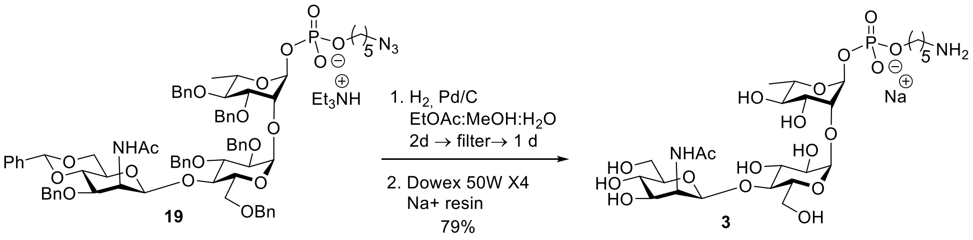

To a degased solution of compound **19** (5.0 mg, 3.58 *μ*mol) in ethyl acetate-methanol-water (3:2:1; 3 mL) was added Pd/C (7 mg; 10%) and hydrogenolyzed using hydrogen for 2 days, then the solids were filtered off and lyophilized the filtrate (^{1}H NMR showed little peaks in aromatic region). Again Pd/C (7 mg; 10%) was added to the mixture in ethyl acetate-methanol-water (3:2:1; 3 mL) and hydrogenolyzed for another 24 h. The mixture was filtered using a PTFE hydrophobic filter, and the residue was purified by gel filtration on a C18 column, using water–acetonitrile (95 : 5) as eluent. The fractions were eluted with water through a column filled with Dowex 50W-X4 resin (Na+) and all the fractions were mixed together and Lyophilized to obtain **3** as a white powder (2.03 mg, 79%). ^{1}H NMR (600 MHz, D_2O) *δ* 5.44 (d, J = 7.4 Hz, 1H, H-1 (α-Rha), 5.04 (d, $^3J_{P,H-1}$ = 3.8 Hz, 1H, H-1' (α-Glc)), 4.91 (d, J = 1.6 Hz, 1H, H-1'' (β-Man)), 4.57 (dd, J = 4.4, 1.7 Hz, 1H, H-2''), 4.15 – 4.08 (m, 1H), 4.00 (t, J = 2.6 Hz, 1H), 3.98 – 3.88 (m, 6H), 3.87 – 3.80 (m, 3H), 3.80 – 3.74 (m, 3H), 3.74 – 3.70 (m, 3H), 3.68 – 3.64 (m, 1H), 3.63 – 3.57 (m, 2H), 3.54 (td, J = 9.8, 3.5 Hz, 2H), 3.47 (m, 1H), 3.05 (m, 2H), 2.09 (s, 3H, $NHCOCH_3$), 1.72 (m, 4H), 1.52 – 1.46 (m, 2H), 1.36 – 1.31 (m, 4H); ^{13}C NMR (176 MHz, D_2O) δ 175.40, 99.28, 97.72, 93.45, 78.56, 77.00, 76.48, 71.98, 71.66, 71.17, 71.05, 70.28, 70.04, 69.64, 69.55, 69.00, 66.57, 66.01, 60.33, 59.69, 53.28, 39.36, 29.10, 26.31, 22.02, 21.99, 16.67; ^{31}P NMR (243 MHz, D_2O) *δ* -1.88; HRMS (ESI): Calcd for $C_{25}H_{47}O_{18}N2P$ $[M+H]^+$ 695.2634, found: 695.2630.

Pent-4-enyl-(2,3-di-O-benzyl-4,6-O-benzylidene-α-D-glucopyranosyl)-(1→3)-2,4-di-O-benzyl-α-L-rhamnopyranoside (20)

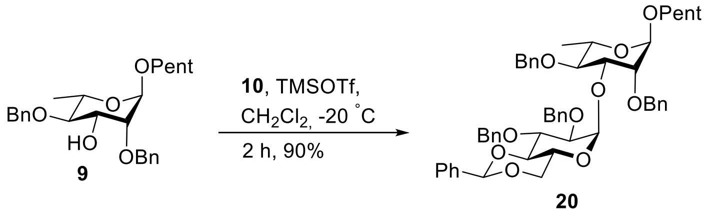

A solution of **9** (0.6 g, 1.45 mmol) and imidate **10** (1.72 g, 2.91 mmol)) and vacuum dried 4 Å molecular sieves (2.0 g) in anhydrous CH_2Cl_2 (40 mL) was cooled to - 20 °C, then TMSOTf (26.3 μL, 0.15 mmol) was added dropwise. The reaction mixture was gradually brought to room temperature over 2 h. After complete consumption of starting material, Et_3N (2 mL) was added and evaporated to dryness. The crude product was purified by silica gel (100-200) column chromatography (Hexane/EtOAc 6:1) to give **20** as a sticky liquid (1.1 g, 90%). $[\alpha]_D^{25}$ = +6.41 (c = 3.13, $CHCl_3$); IR ν_{max} (film) 3061, 3032, 2922, 1723, 1643, 1606, 1582, 1496, 1452, 1364, 1313, 1275, 1207, 1177, 1091, 1052, 1026, 912, 842, 804, 735, 712, 697 cm^{-1}; ^1H NMR (400 MHz, $CDCl_3$) δ 7.57 (d, *J* = 8.0 Hz, 2H, Ar), 7.46 (dd, *J* = 6.7, 3.0 Hz, 2H, Ar), 7.44 – 7.34 (m, 10H, Ar), 7.33 – 7.27 (m, 10H, Ar), 7.22 – 7.14 (m, 3H, Ar), 5.79 (ddt, *J* = 16.9, 10.2, 6.6 Hz, 1H, C*H*=CH_2), 5.56 (s, 1H, PhCH), 5.13 (d, *J* = 3.6 Hz, 1H, H-1 (α-Rha)), 5.07 – 4.90 (m, 5H, $PhCH_2$), 4.89 – 4.73 (m, 5H, $PhCH_2$), 4.69 (d, *J* = 2.0 Hz, 1H, H-1'), 4.59 (m, 2H, $PhCH_2$), 4.23 – 4.12 (m, 3H), 4.08 (dd, *J* = 8.8, 2.9 Hz, 1H), 3.89 (t, *J* = 2.5 Hz, 1H), 3.66 (m, 6H), 3.33 (dt, *J* = 9.8, 6.5 Hz, 1H), 2.07 (q, *J* = 7.1 Hz, 2H, pentenyl), 1.68 – 1.62 (m, 2H, pentenyl), 1.34 (d, *J* = 5.8 Hz, 3H, CH_3); ^{13}C NMR (101 MHz, $CDCl_3$) δ 138.71, 138.68, 138.26, 138.19, 138.17, 138.10, 137.74, 129.55, 128.97, 128.58, 128.52, 128.49, 128.45, 128.42, 128.39, 128.34, 128.29, 128.27, 128.23, 127.92, 127.86, 127.75, 127.66, 126.54, 126.33, 120.62, 115.06, 101.38, 98.27, 96.95, 82.66, 80.38, 79.17, 78.64, 77.52, 77.47, 75.75, 75.25, 73.90, 73.40, 69.15, 68.35, 66.96, 63.14, 30.43, 28.76, 18.12; HRMS (ESI): Calcd for $C_{52}H_{58}O_{10}$ $[M+Na]^+$ 865.3927, found: 865.3927.

Pent-4-enyl-(2,3,6-tri-O-benzyl-α-D-glucopyranosyl)-(1→3)-2,4-di-O-benzyl-α-L-rhamnopyranoside (21)

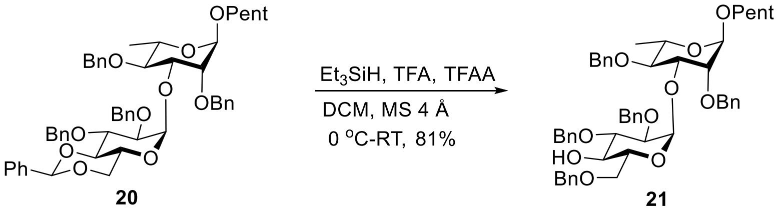

To a mixture of **20** (0.85 g, 1.01 mmol), MS 4 Å (1.7 g) in anhydrous CH_2Cl_2 (18 mL) were added triflouroacetic anhydride (71.2 μL, 0.50 mmol) and Et_3SiH (0.96 mL, 6.04 mmol) at 0 °C, slowly. After stirring the reaction mixture for 10 min, TFA (0.39 mL, 5.04 mmol) was added dropwise at 0 °C. The reaction mixture was gradually brought to

room temperature over 2 h. The reaction was quenched with acetone (2 mL) and the solids were filtered off. The mixture was poured into aq. $NaHCO_3$ and extracted with DCM (3x20 mL). The combined organic extracts were washed with water, brine (50 mL), dried over Na_2SO_4, filtered and concentrated. The crude was purified by flash chromatography using hexane and ethyl acetate as eluent (3:1) to give the desired product **21** as pale yellow oil (0.69 g, 81%). $[\alpha]_D^{25}$ = +23.27 (c = 1.71, $CHCl_3$); IR ν_{max} (film) 3449, 3065, 3033, 2925, 1723, 1641, 1603, 1586, 1497, 1454, 1362, 1316, 1271, 1209, 1177, 1093, 1054, 1026, 913, 841, 805, 737, 711, 697 cm^{-1}; ^1H NMR (600 MHz, $CDCl_3$) δ 7.26 (m, 4H, Ar), 7.24 – 7.14 (m, 20H, Ar), 5.69 (ddt, J = 16.9, 10.3, 6.6 Hz, 1H, CH=CH_2), 5.11 (d, J = 3.3 Hz, 1H, H-1 (α-Rha)), 4.95 – 4.85 (m, 3H, $PhCH_2$), 4.81 (d, J = 10.8 Hz, 1H, PhCHH), 4.74 (d, J = 12.0 Hz, 1H, PhCHH), 4.71 – 4.59 (m, 4H, $PhCH_2$, H-1' (α-Glc)), 4.53 (dd, J = 11.4, 2H, $PhCH_2$), 4.43 (d, J = 12.1 Hz, 1H, $PhCH_2$), 4.32 (d, J = 12.2 Hz, 1H, $PhCH_2$), 4.03 (dd, J = 9.0, 2.9 Hz, 1H, H-2'), 3.91 (dt, J = 9.8, 3.8 Hz, 1H), 3.86 – 3.78 (m, 2H), 3.65 – 3.50 (m, 6H), 3.49 – 3.40 (m, 2H), 3.24 (dt, J = 9.8, 6.5 Hz, 1H), 1.97 (q, J = 7.3 Hz, 2H, pentenyl), 1.52 (p, J = 7.0 Hz, 2H, pentenyl), 1.24 (d, J = 6.1 Hz, 3H, CH_3); ^{13}C NMR (151 MHz, $CDCl_3$) δ 138.92, 138.74, 138.44, 138.25, 138.23, 138.22, 128.69, 128.53, 128.44, 128.41, 128.39, 128.20, 128.13, 127.93, 127.87, 127.84, 127.79, 127.70, 127.68, 127.65, 115.04, 98.28, 95.29, 81.54, 80.32, 79.53, 76.48, 75.79, 75.39, 75.33, 73.58, 73.39, 73.26, 71.29, 70.35, 69.51, 68.40, 67.03, 30.45, 28.81, 18.22; HRMS (ESI): Calcd for $C_{52}H_{60}O_{10}$ $[M+Na]^+$ 883.3823, found: 883.3835.

Pent-4-enyl-(3-*O*-benzyl-4,6-*O*-benzylidene-2-levulinoyl-*β*-D-glucopyranosyl)-(1→4)-(2,3,6-tri-*O*-benzyl-α-D-glucopyranosyl)-(1→3)-2,4-di-*O*-benzyl-α-L-rhamnopyranoside (22)

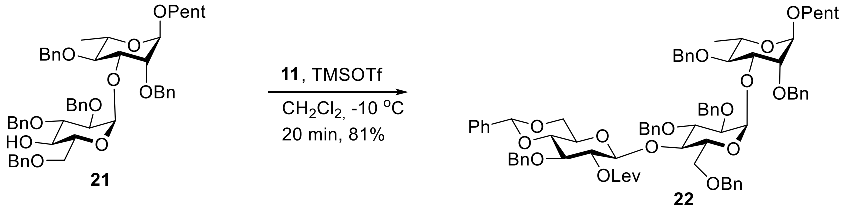

To a solution of **21** (0.34 g, 0.40 mmol) and imidate **11** (0.38 g, 0.64 mmol) and 4 Å molecular sieves (2.0 g) in anhydrous CH_2Cl_2 (40 mL) was cooled to - 10 °C, then TMSOTf (7.09 μL, 0.04 mmol) was added dropwise. After 20 min, the reaction was quenched Et_3N (1 mL) and then the solids were filtered off. The filtrate was evaporated *in vacuo* to dryness. The crude product was purified by silica gel (100-200) column

chromatography (Hexane/EtOAc 4:1) to give **22** as a sticky solid (0.42 g, 81%). $[\alpha]_D^{25}$ = +7.71 (c = 2.27, $CHCl_3$); IR ν_{max} (film) 3065, 3032, 2962, 2926, 2872, 1751, 1720, 1605, 1497, 1455, 1365, 1313, 1261, 1208, 1177, 1087, 1027, 912, 802, 748, 696, 667 cm^{-1}; ^1H NMR (400 MHz, $CDCl_3$) δ 7.50 (m, Ar, 2H), 7.45 – 7.38 (m, 6H, Ar), 7.38 – 7.29 (m, 21H, Ar), 7.27 – 7.22 (m, 8H, Ar), 5.80 (ddt, *J* = 16.9, 10.1, 6.6 Hz, 1H, C*H*=CH_2), 5.48 (s, 1H, PhCH), 5.17 (d, *J* = 3.5 Hz, 1H, H-1 (α-Rha)), 5.05 – 4.90 (m, 4H, $PhCH_2$, H-2''), 4.85 (dd, *J* = 11.5, 5.8 Hz, 2H, $PhCH_2$), 4.81 – 4.61 (m, 9H, $PhCH_2$), 4.57 (d, *J* = 11.1 Hz, 1H, PhCH*H*), 4.42 (d, *J* = 8.0 Hz, 1H, H-1''), 4.27 (d, *J* = 12.1 Hz, 1H, PhC*H*H), 4.17 (dd, *J* = 10.5, 5.0 Hz, 1H), 4.10 – 4.01 (m, 2H), 4.01 – 3.87 (m, 3H), 3.80 (dd, *J* = 11.0, 2.2 Hz, 1H), 3.71 – 3.55 (m, 6H), 3.53 – 3.38 (m, 3H), 3.33 (dt, *J* = 9.7, 6.4 Hz, 1H), 3.13 (td, *J* = 9.7, 5.0 Hz, 1H), 2.60 (q, *J* = 6.6 Hz, 2H), 2.35 (td, *J* = 6.7, 5.0 Hz, 2H), 2.07 (s, 4H, Lev), 1.67 – 1.56 (m, 7H), 1.24 (d, *J* = 5.5 Hz, 3H, CH_3). ^{13}C NMR (101 MHz, $CDCl_3$) δ 206.09, 171.17, 139.39, 138.66, 138.61, 138.49, 138.42, 138.20, 137.89, 137.40, 129.17, 128.70, 128.69, 128.58, 128.56, 128.53, 128.43, 128.41, 128.39, 128.27, 128.20, 128.19, 128.12, 127.93, 127.90, 127.85, 127.79, 127.74, 127.72, 127.68, 127.62, 127.35, 126.16, 115.02, 101.26, 100.74, 98.31, 97.19, 81.75, 80.25, 80.02, 79.18, 78.67, 77.48, 76.84, 76.46, 75.27, 74.11, 73.75, 73.68, 73.42, 73.26, 70.83, 68.76, 68.17, 67.61, 66.91, 65.95, 37.87, 30.43, 29.89, 28.80, 27.87, 18.10; HRMS (ESI): Calcd for $C_{77}H_{86}O_{17}$ $[M+Na]^+$ 1305.5762, found: 1305.5757.

Pent-4-enyl-(3-*O*-benzyl-4,6-*O*-benzylidene-β-D-glucopyranosyl)-(1→4)-(2,3,6-tri-*O*-benzyl-*α*-D-glucopyranosyl)-(1→3)-2,4-di-*O*-benzyl-*α*-L-rhamnopyranoside (23)

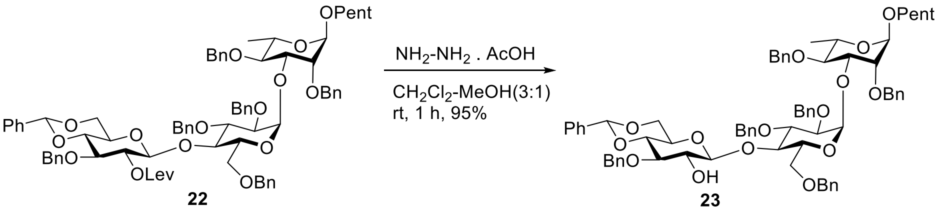

Hydrazine acetate (43 mg, 0.47 mmol) dissolved in MeOH (3 mL), was added to a solution of **22** (0.4 g, 0.31 mmol) in DCM (12 mL). The reaction mixture was stirred at rt for 1h and afterwards quenched by the addition of acetone. Then it was evaporated to dryness and purified by silica gel column chromatography (Hexane/EtOAc 5:1) to afford **23** (0.35 g, 95%). $[\alpha]_D^{25}$ = 16.83 (c = 1.21, $CHCl_3$); IR ν_{max} (film) 3428, 3065, 3033, 2926, 1723, 1641, 1604, 1497, 1454, 1365, 1315, 1263, 1209, 1094, 1069, 1027, 913, 806, 737, 697 cm^{-1}; ^1H NMR (400 MHz, $CDCl_3$) δ 7.39 (m, 2H, Ar), 7.35 – 7.08

(m, 28H, Ar), 5.71 (ddt, *J* = 17.0, 10.2, 6.7 Hz, 1H, C*H*=CH_2), 5.38 (s, 1H, PhCH), 5.06 (d, *J* = 3.5 Hz, 1H, H-1 (α-Rha)), 4.97 – 4.46 (m, 14H, $PhCH_2$, H-1''), 4.28 – 4.21 (m, 2H, PhCH*H*, H-1''), 4.01 (dd, *J* = 9.1, 2.9 Hz, 1H), 3.98 – 3.87 (m, 4H), 3.77 (t, *J* = 2.5 Hz, 1H), 3.70 (d, *J* = 11.2 Hz, 1H), 3.67 – 3.50 (m, 4H), 3.49 – 3.25 (m, 5H), 3.22 (m, 2H), 2.97 (m, 1H), 2.14 (d, *J* = 3.2 Hz, 1H, OH), 2.05 – 1.94 (m, 2H, pentenyl), 1.58 – 1.48 (m, 2H, pentenyl), 1.27 (d, *J* = 6.0 Hz, 3H, CH_3); ^{13}C NMR (101 MHz, $CDCl_3$) *δ* 139.29, 138.83, 138.70, 138.61, 138.23, 138.18, 137.77, 137.50, 129.08, 128.56, 128.49, 128.47, 128.44, 128.36, 128.34, 128.27, 128.19, 128.03, 127.96, 127.86, 127.82, 127.81, 127.78, 127.75, 127.61, 127.43, 127.40, 126.18, 115.05, 103.47, 101.25, 98.27, 94.87, 81.32, 80.78, 80.33, 80.04, 78.97, 77.48, 76.19, 75.30, 75.18, 74.59, 73.74, 73.52, 73.39, 70.03, 68.82, 68.34, 68.10, 66.93, 66.20, 30.42, 28.74, 18.09; HRMS (ESI): Calcd for $C_{77}H_{80}O_{15}$ $[M+Na]^+$ 1207.5394, found: 1207.5386.

Pent-4-enyl-(2-azido-3-*O*-benzyl-4,6-*O*-benzylidene-*β*-D-mannopyranosyl)-(1→4)-(2,3,6-tri-O-benzyl-*α*-D-glucopyranosyl)-(1→3)-2,4-di-*O*-benzyl-L-rhamnopyranoside (24)

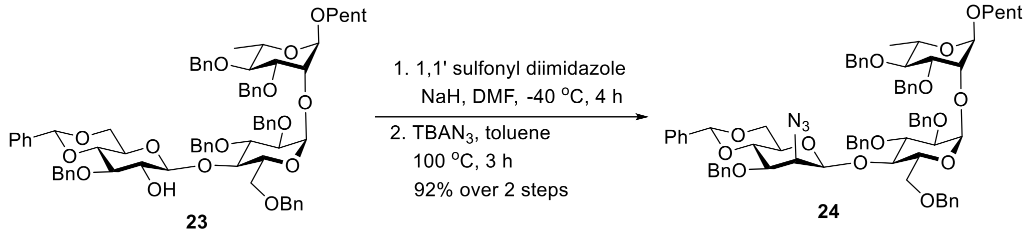

To a solution of **23** (0.1 g, 84.3 *μ*mol) in DMF (5 mL) was added NaH (60%) (12.2 mg, 0.51 mmol) at 0 °C and then the reaction was brought to rt over 1 h. The reaction was cooled to -40 °C, to this 1,1′-sulfonyldiimidazole (25.3 mg, 0.13 mmol). The reaction was stirring at the same temperature for 4 h before quenching with methanol (1 mL). The reaction mixture was partitioned between diethyl ether (3 x 10 mL) and water (10 mL). The combined organic layers were washed with water, brine (50 mL), dried over Na_2SO_4, filtered and concentrated. The crude product was directly taken for the next step without purification.

The sulfonyl derivative was dissolved in anhydrous toluene (1 mL) and then tetrabutylammonium azide (54.6 mg, 0.19 mmol) was added. The reaction was heated at 110 °C for 3 h. The reaction mixture was diluted with water and extracted with ethyl acetate (2 x 15 mL). The combined organic extracts were washed with water, brine (10 mL), dried over Na_2SO_4, filtered and concentrated. The crude was purified by flash

chromatography using hexane and ethyl acetate as eluent (4:1) to give the desired product **24** (94 mg, 92% over 2 steps). $[\alpha]_D^{25}$ = -4.72 (c = 0.70, $CHCl_3$); IR ν_{max} (film) 3033, 2927, 2108, 1498, 1455, 1363, 1262, 1211, 1097, 1052, 1028, 914, 805, 737, 698 cm^{-1}; ^1H NMR (600 MHz, $CDCl_3$) δ 7.47 (d, J = 7.2 Hz, 2H, Ar), 7.42 – 7.28 (m, 14H, Ar), 7.28 – 7.18 (m, 15H, Ar), 7.15 (t, J = 7.4 Hz, 1H, Ar), 5.80 (ddt, J = 16.9, 10.3, 6.6 Hz, 1H, C*H*=CH2), 5.48 (s, 1H, PhCH), 5.10 (d, J = 3.6 Hz, 1H, H-1 (α-Rha)), 5.06 – 4.95 (m, 3H, $PhCH_2$), 4.90 (d, J = 11.5 Hz, 1H, PhCH*H*), 4.86 – 4.61 (m, 9H, $PhCH_2$, H-1''), 4.55 (d, J = 12.1 Hz, 1H, PhCH*H*), 4.29 (s, 1H, H-1'' (β-Man)), 4.16 (d, J = 12.1 Hz, 1H, H-6), 4.10 – 3.92 (m, 5H), 3.89 – 3.78 (m, 2H), 3.71 (dt, J = 12.4, 6.1 Hz, 1H), 3.67 – 3.56 (m, 3H), 3.53 – 3.43 (m, 2H), 3.38 (d, J = 11.1 Hz, 1H), 3.32 (dt, J = 9.8, 6.5 Hz, 1H), 3.25 (m, 2H), 2.92 (td, J = 9.7, 4.9 Hz, 1H), 2.08 (q, J = 7.2 Hz, 2H), 1.63 (p, J = 6.9 Hz, 2H), 1.31 (d, J = 6.2 Hz, 3H, CH_3); ^{13}C NMR (101 MHz, $CDCl_3$) δ 139.22, 138.67, 138.61, 138.22, 138.09, 137.42, 137.36, 129.01, 128.58, 128.52, 128.46, 128.33, 128.25, 128.18, 128.13, 128.08, 128.01, 127.86, 127.74, 127.69, 127.66, 127.54, 127.41, 127.37, 126.07, 114.95, 101.49, 99.65, 98.28, 95.74, 80.53, 79.71, 78.83, 78.49, 76.75, 76.18, 75.87, 75.18, 74.67, 73.80, 73.61, 73.50, 72.46, 69.55, 68.42, 67.97, 67.02, 66.82, 63.11, 30.34, 28.65, 17.99; HRMS (ESI): Calcd for $C_{73}H_{81}N3O_{14}$ $[M+Na]^+$ 1232.5454, found: 1232.5549.

Pent-4-enyl-(2-acetamido-3-*O*-benzyl-4,6-*O*-benzylidene-*β*-D-mannopyranosyl)-(1→4)-(2,3,6-tri-O-benzyl-*α*-D-glucopyranosyl)-(1→3)-2,4-di-*O*-benzyl-L-rhamnopyranoside (25)

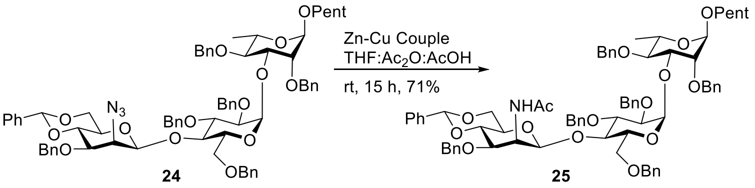

To a solution of **24** (0.2 g, 165.2 μmol) in THF : Ac_2O : AcOH (6.7 mL, 6 : 4 : 2) was added freshly activated Zn-Cu couple (0.4 g) at room temperature. The reaction was stirred at rt for 15 h. The reaction mixture was diluted with EtOAc and the solids were filtered off. The filtrate was concentrated *in vacuo* and the resulting residue was purified by flash chromatography using hexane and ethyl acetate as eluent (3:2) to give the desired product **25** (144 mg, 71%). $[\alpha]_D^{25}$ = -3.28 (c = 1.93, $CHCl_3$); IR ν_{max} (film) 3365, 3065, 3033, 2926, 2857, 1727, 1603, 1497, 1454, 1366, 1315, 1270, 1228, 1176, 1094,

1068, 1027, 913, 843, 749, 710, 697 cm^{-1}; ^{1}H NMR (400 MHz, $CDCl_3$) δ 7.48 – 7.39 (m, 2H, Ar), 7.39 – 7.06 (m, 29H, Ar), 6.99 (m, 2H, Ar), 5.71 (ddt, J = 16.9, 10.2, 6.6 Hz, 1H, CH=CH_2), 5.37 (m, 2H, NH, PhCH), 5.04 (d, J = 3.6 Hz, 1H, H-1), 4.95 – 4.81 (m, 4H, $PhCH_2$), 4.80 – 4.69 (m, 3H, $PhCH_2$), 4.69 – 4.39 (m, 9H, $PhCH_2$, H-1'), 4.30 (s, 1H, H-1''), 4.12 (d, J = 12.0 Hz, 1H, H-6), 4.06 (dd, J = 10.5, 5.0 Hz, 1H), 4.00 – 3.91 (m, 2H), 3.91 – 3.81 (m, 2H), 3.77 (t, J = 2.5 Hz, 1H), 3.64 (dt, J = 9.3, 6.0 Hz, 1H), 3.59 – 3.49 (m, 4H), 3.44 (t, J = 9.6 Hz, 1H), 3.29 – 3.16 (m, 4H), 2.97 (td, J = 9.7, 4.8 Hz, 1H), 2.05 – 1.96 (m, 2H, pentenyl), 1.64 (s, 3H, $NHCOCH_3$), 1.56 (dt, J = 8.0, 6.7 Hz, 2H, pentenyl), 1.23 (d, J = 6.1 Hz, 3H, CH_3); ^{13}C NMR (101 MHz, $CDCl_3$) δ 170.40, 139.42, 138.74, 138.38, 138.22, 138.14, 138.11, 137.52, 137.42, 129.09, 128.64, 128.51, 128.46, 128.39, 128.36, 128.30, 128.28, 128.23, 128.15, 128.06, 127.77, 127.72, 127.70, 127.51, 127.47, 126.57, 126.16, 115.02, 101.69, 99.45, 98.24, 96.46, 80.95, 79.90, 79.29, 78.65, 77.94, 76.36, 75.78, 75.70, 75.05, 75.00, 73.60, 73.51, 73.38, 71.24, 70.12, 68.78, 68.35, 67.88, 67.01, 66.89, 50.47, 30.39, 28.72, 23.21, 18.14; HRMS (ESI): Calcd for $C_{74}H_{83}O_{15}$ $[M+Na]^+$ 1248.5660, found: 1248.5677.

2-Acetamido-3-*O*-benzyl-4,6-*O*-benzylidene-β-D-mannopyranosyl-(1→4)-(2,3,6-tri-*O*-benzyl-α-D-glucopyranosyl)-(1→3)-2,4-di-*O*-benzyl-L-rhamnopyranose (26)

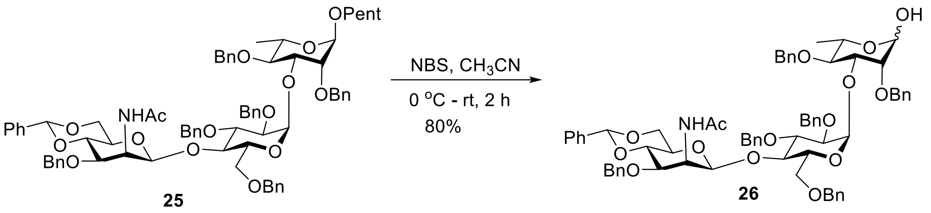

To a solution of **25** (60 mg, 49.0 μmol) in anhydrous acetonitrile (5 mL) was added NBS (11.32 mg, 64.0 μmol) at rt. After 2 h stirring at rt, the reaction was diluted with EtOAc and quenched by addition aq. $NaHCO_3$. The combined organic extracts were washed with water, brine (10 mL), dried over Na_2SO_4, filtered and concentrated. The crude was purified by flash chromatography using hexane and ethyl acetate as eluent (7:3) to give the hemiacetal **26** as white semi solid (α : β =1.5 : 1; 46 mg, 80%). $[\alpha]_D^{25}$ = +11.68 (c = 1.0, $CHCl_3$); IR ν_{max} (film) 3409, 3062, 3030, 2921, 2852, 1963, 1664, 1495, 1452, 1362, 1332, 1310, 1291, 1264, 1211, 1174, 1075, 1042, 1025, 907, 804, 754, 733, 697, 666 cm^{-1}; ^{1}H NMR (400 MHz, $CDCl_3$) δ 7.45 – 7.39 (m, 3H), 7.32 (dd, J = 8.1, 3.4 Hz, 10H), 7.29 – 7.20 (m, 8H), 7.17 (d, J = 3.9 Hz, 4H), 7.15 – 7.06 (m, 7H), 7.00 (h, J = 4.4 Hz, 1H), 5.39 (s, 2H), 5.35 (d, J = 9.4 Hz, 1H), 5.20 (s, 1H), 5.14 – 5.03

(m, 2H), 5.00 (d, J = 11.5 Hz, 1H), 4.84 (d, J = 11.7 Hz, 2H), 4.79 – 4.61 (m, 5H), 4.61 – 4.53 (m, 4H), 4.53 – 4.40 (m, 3H), 4.30 (d, J = 8.7 Hz, 1H), 4.14 (dd, J = 12.0, 8.3 Hz, 2H), 4.10 – 4.01 (m, 2H), 3.99 – 3.71 (m, 7H), 3.62 – 3.48 (m, 3H), 3.44 (dd, J = 12.6, 6.6 Hz, 2H), 3.38 – 3.30 (m, 1H), 3.22 (d, J = 10.8 Hz, 3H), 2.97 (tdd, J = 10.0, 4.9, 2.0 Hz, 1H), 2.86 (s, 1H), 1.65 (s, 1H), 1.64 (s, 2H), 1.25 (d, J = 6.2 Hz, 2H), 1.22 (d, J = 6.3 Hz, 3H); ^{13}C NMR (101 MHz, $CDCl_3$) δ 170.51, 139.47, 139.25, 138.77, 138.43, 138.36, 138.29, 138.23, 138.17, 138.02, 137.76, 137.62, 137.48, 137.43, 129.19, 129.16, 128.76, 128.71, 128.67, 128.64, 128.57, 128.54, 128.53, 128.47, 128.43, 128.39, 128.38, 128.36, 128.28, 128.27, 128.24, 128.21, 128.18, 128.13, 128.10, 128.03, 128.00, 127.83, 127.79, 127.77, 127.63, 127.60, 127.54, 126.65, 126.62, 126.23, 126.22, 101.79, 101.77, 99.58, 99.50, 97.06, 96.39, 93.23, 93.13, 81.00, 80.94, 79.90, 79.68, 79.38, 79.15, 78.72, 78.68, 77.52, 76.60, 76.50, 75.86, 75.84, 75.77, 75.13, 75.09, 74.94, 74.71, 74.64, 74.36, 73.72, 73.67, 73.61, 73.50, 71.53, 71.32, 70.36, 70.21, 68.85, 68.79, 68.63, 67.97, 67.87, 67.17, 67.09, 53.60, 50.54, 32.08, 29.86, 29.82, 29.52, 23.29, 23.26, 22.86, 18.56, 18.31, 14.30; HRMS (ESI): Calcd for $C_{69}H_{75}O_{15}$ $[M+Na]^+$ 1180.5034, found: 1180.5028.

5-Azidopentyl-1-*O*-(2-acetamido-3-*O*-benzyl-4,6-*O*-benzylidene-β-D-mannopyranosyl-(1→4)-(2,3,6-tri-*O*-benzyl-α-D-glucopyranosyl)-(1→3)-2,4-di-*O*-benzyl-L-rhamnopyranosyl phosphate) triethylammonium salt (27)

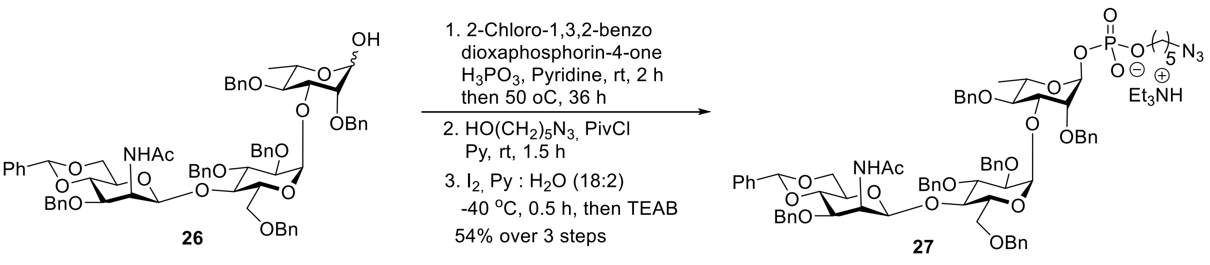

Compound **26** (8 mg, 6.91 μmol) and phosphorous acid (1.7 mg, 20.72 μmol) were mixed and co-evaporated with pyridine (3 x 1.0 mL). The dried reaction mixture was kept under high vacuum for overnight. To a solution premixed compound **26** and H_3PO_3 in anhydrous pyridine (1.0 mL) was added 2-chloro-5,5-dimethyl-2-oxo-1,3,2-dioxaphosphorinane (2.1 mg g, 10.36 μmol) at 0 ^{o}C. The ice bath was removed after 10 min, and the reaction mixture was stirred for 2 h at rt. Then the reaction mixture was heated to 50 ^{o}C, and stirring was continued for 36 h. Then 1 M aq. triethylammonium bicarbonate (2 mL) was added to destroy excess of reagent, and the mixture was

diluted with dichloromethane, washed with 0.5 M aq. triethylammonium bicarbonate (TEAB), dried over Na_2SO_4, filtered and concentrated. The residue was purified by column chromatography (ethyl acetate–hexane (1:1 to 1:0) then dichloromethane–methanol (20:1) together with 1% trimethylamine as eluents. The product fractions were evaporated then was dissolved in dichloromethane and washed with 0.25 M aq. triethylammonium bicarbonate (TEAB). The organic layer was separated, concentrated *in vacuo* to give the H-phosphonate as foam (7 mg).

H-phosphonate (7 mg, 5.29 *μ*mol) and C5 azido linker (2.05 mg, 15.87 *μ*mol) were mixed and co-evaporated with pyridine (3 x 1.0 mL). The dried reaction mixture was kept under high vacuum for 30 min. The reaction mixture was dissolved in anhydrous pyridine (1.0 mL) and then pivaloyl chloride (1.62 *μ*L, 13.22 *μ*mol) was added. The reaction was stirred at rt for 2 h. Then the reaction was cooled to -40 ^oC, a freshly prepared solution of iodine (3.2 mg, 13.22 *μ*mol) in pyridine-water (19 : 1, 0.1 mL) was added. After 15 min the mixture was diluted with diluted with dichloromethane, washed successively with 10% aq. sodium thiosulfate, 0.5 M aq. triethylammonium hydrogen carbonate (TEAB), dried (Na_2SO_4), filtered and concentrated. The residue was purified by column chromatography (ethyl acetate–hexane (1:1 to 1:0) then dichloromethane–methanol (25:1) together with 1% trimethylamine as eluents. The product fractions were evaporated then was dissolved in dichloromethane and washed with 0.25 M aq. triethylammonium hydrogen carbonate. The organic layer was separated, concentrated and co-concentrated from dichloromethane (5 × 10 mL) to afford **27** as foam (5 mg, 54% over 3 steps). ^{1}H NMR (400 MHz, $CDCl_3$) *δ* 11.72 (s, 8H, NH (excess TEAB salt)), 7.54 – 7.48 (m, 3H, Ar), 7.39 (m, 22H, Ar), 7.31 (m, 8H, Ar), 7.25 (m, 8H, Ar), 7.22 – 7.17 (m, 13H, Ar), 5.63 – 5.54 (br d, 1H, H-1), 5.48 (s, 1H, PhCH), 5.44 (d, *J* = 9.0 Hz, 1H, NH), 5.17 (m, 3H, $PhCH_2$), 5.13 – 5.06 (s, 1H, H-1’), 4.93 (d, *J* = 11.7 Hz, 1H, PhCH*H*), 4.88 – 4.76 (m, 4H, $PhCH_2$), 4.74 – 4.58 (m, 8H, $PhCH_2$), 4.50 (m, 6H, $PhCH_2$), 4.33 (s, 1H, H-1’’ (β-Man)), 4.22 – 4.11 (m, 4H), 3.97 (m, 7H), 3.81 (d, *J* = 18.7 Hz, 3H), 3.72 – 3.55 (m, 6H, $N\underline{(CH_2)_3}(CH_3)_3$)), 3.55 – 3.46 (m, 2H), 3.29 – 3.22 (m, 4H), 3.12 (m, 6H), 1.69 (s, 3H, $NHCOCH_3$), 1.51-1.59 (m, 5H), 1.40 (t, *J* = 7.3 Hz, 9H, $N(CH_2)_3\underline{(CH_3)_3}$); ^{13}C NMR (151 MHz, $CDCl_3$) δ 170.42, 139.56, 138.95, 138.59, 138.36, 138.22, 138.04, 137.70, 137.55, 129.14, 128.68, 128.52, 128.45, 128.41, 128.36, 128.21, 128.05, 127.94, 127.81, 127.73, 127.64, 127.36, 126.65, 126.25, 101.77, 99.38, 95.61, 94.66, 81.02, 79.36, 79.24, 78.76, 75.61, 75.05, 74.85, 73.67,

73.45, 73.34, 71.34, 69.96, 69.48, 68.88, 68.06, 67.28, 67.11, 65.85, 50.57, 46.05, 30.63, 29.85, 27.42, 23.26, 18.30, 8.79; ^{31}P NMR (162 MHz, $CDCl_3$): δ_P -2.49. HRMS (ESI): Calcd for $C_{74}H_{85}N_4O_{18}P$ [M-Et3N+H]$^+$ 1349.5669, found: 1349.5693.

5-Aminopentyl-1-*O*-(2-Acetamido-2-deoxy-*β*-D-mannopyranosyl-(1→4)-(α-D-glucopyranosyl)-(1→3)-L-rhamnopyranosyl phosphate) sodium salt (4)

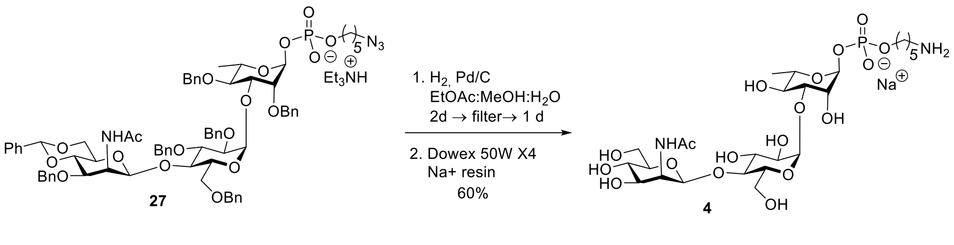

To a degased solution of compound **27** (5.0 mg, 3.58 *μ*mol) in ethyl acetate-methanol-water (3:2:1; 3 mL) was added Pd/C (7 mg; 10%) and hydrogenolyzed using hydrogen for 2 days, then the solids were filtered off and lyophilized the filtrate (^{1}H NMR showed little peaks in aromatic region). Again Pd/C (7 mg; 10%) was added to the mixture in ethyl acetate-methanol-water (3:2:1; 3 mL) and hydrogenolyzed for another 24 h. The mixture was filtered using a PTFE hydrophobic filter, and the residue was purified by gel filtration on a C18 column, using water–acetonitrile (95 : 5) as eluent. The obtained fractions were eluted with water through a column filled with Dowex 50W-X4 resin (Na+) and all the fractions were mixed together and Lyophilized to obtain **4** as a white powder (1.55 mg, 60%). ^{1}H NMR (600 MHz, D_2O) δ 5.66 (d, $^3J_{P,H-1}$ = 7.8 Hz, 1H, H-1 (α-Rha)), 5.36 (d, J = 3.9 Hz, 1H, H-1’ (α-Glc)), 5.17 (s, 1H, H-1’’ (β-Man)), 4.43 (s, 1H, H-2’’), 4.30 (d, J = 10.1 Hz, 1H), 4.23 – 4.15 (m, 6H), 4.10 (td, J = 10.3, 9.8, 3.9 Hz, 3H), 4.05 (d, J = 3.2 Hz, 2H), 4.03 (d, J = 3.9 Hz, 1H), 3.99 (d, J = 9.2 Hz, 2H), 3.94 (d, J = 4.4 Hz, 1H), 3.92 – 3.87 (m, 2H), 3.86 – 3.78 (m, 4H), 3.73 (s, 1H), 3.31 (t, J = 7.5 Hz, 2H), 2.54 (t, J = 7.3 Hz, 1H), 2.44 (t, J = 7.9 Hz, 1H), 2.35 (s, 3H), 2.02 – 1.94 (m, 4H), 1.78 – 1.72 (m, 3H), 1.59 (d, J = 6.2 Hz, 3H, CH_3). ^{13}C NMR (176 MHz, D_2O) δ 175.43, 99.28, 95.58, 95.53, 78.45, 76.47, 75.38, 72.03, 71.93, 71.46, 71.14, 70.15, 70.10, 69.74, 67.43, 66.60, 66.00, 62.46, 60.35, 59.62, 53.31, 39.36, 35.03, 29.09, 26.28, 22.00, 21.95, 16.86; ^{31}P NMR (243 MHz, D_2O): δ_P -1.92; HRMS (ESI): Calcd for $C_{25}H_{47}O_{18}N2P$ [M+H]$^+$ 695.2634, found: 695.2629.

Pent-4-enyl-(2-acetamido-3,6-di-*O*-benzyl -*β*-D-mannopyranosyl)-(1→4)-(2,3,6-tri-O-benzyl-*α*-D-glucopyranosyl)-(1→3)-2,4-di-*O*-benzyl-L-rhamnopyranoside (28)

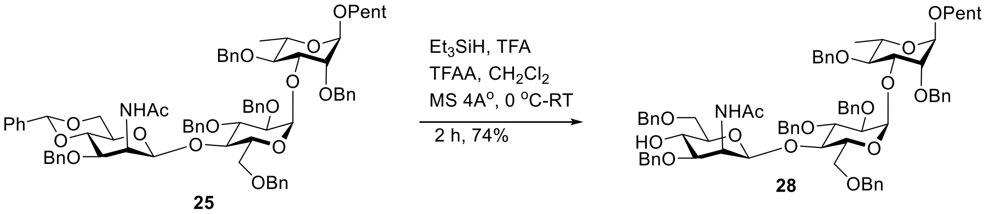

To a mixture of **25** (50.0 mg, 40.76 *μ*mol), MS 4 Å (0.1 g) in anhydrous CH_2Cl_2 (2 mL) were added triflouro aceticanhydride (3.0 *μ*L, 20.38 *μ*mol) and Et_3SiH (40 *μ*L, 24.45 *μ*mol) slowly at 0 °C. After stirring the reaction mixture for 10 min, TFA (15.7 *μ*L, 20.38 *μ*mol) was added dropwise at 0 °C. The reaction mixture was gradually brought to room temperature over 2 h. The reaction was quenched with acetone (1 mL) and solids were filtred off. The mixture was poured into aq. $NaHCO_3$ and extracted with DCM (3x10 mL). The combined organic extracts were washed with water, brine (50 mL), dried over Na_2SO_4, filtered and concentrated. The crude was purified by flash chromatography using hexane and ethyl acetate as eluent (3:1) to give the desired product **28** as foam (37 mg, 74%). $[\alpha]_D^{25}$ = -56.63 (c = 0.45, $CHCl_3$); IR ν_{max} (film) 3361, 3060, 3026, 2922, 2853, 1726, 1602, 1495, 1452, 1368, 1312, 1270, 1226, 1096, 1066, 1027, 913, 843, 710, 697 cm^{-1}; ^1H NMR (700 MHz, $CDCl_3$) *δ* 7.42 – 7.35 (m, 7H, Ar), 7.34 (m, 5H, Ar), 7.32 – 7.27 (m, 11H, Ar), 7.25 – 7.23 (m, 3H, Ar), 7.19 (m, 10H, Ar), 5.79 (ddt, *J* = 17.0, 10.0, 6.5 Hz, 1H, C*H*=CH_2), 5.51 (d, *J* = 9.5 Hz, 1H, NH), 5.13 (d, *J* = 3.6 Hz, 1H, H-1), 5.04 – 4.95 (m, 2H, CH=*CH_2*), 4.93 – 4.84 (m, 3H, $PhCH_2$), 4.80 (dd, *J* = 11.7 Hz, 2H, $PhCH_2$), 4.70 – 4.46 (m, 11H, $PhCH_2$, H-1', H-1''), 4.31 (d, *J* = 12.1 Hz, 1H, PhCH*H*), 4.25 (d, *J* = 11.0 Hz, 1H, PhCH*H*), 4.03 (m, 2H), 3.97 (m, 2H), 3.84 (t, *J* = 2.5 Hz, 1H), 3.70 (dq, *J* = 12.1, 6.3 Hz, 1H), 3.66 – 3.51 (m, 7H), 3.41 – 3.29 (m, 3H), 3.11 (dt, *J* = 8.9, 3.9 Hz, 1H), 3.05 (dd, *J* = 9.4, 3.9 Hz, 1H), 2.07 (q, *J* = 7.2 Hz, 2H), 1.67 (s, 3H, $NHCOCH_3$), 1.64 – 1.60 (m, 2H), 1.32 (d, *J* = 6.2 Hz, 3H, CH_3); ^{13}C NMR (176 MHz, $CDCl_3$) δ 170.33, 139.50, 138.68, 138.30, 138.11, 138.06, 137.82, 137.73, 128.52, 128.49, 128.41, 128.38, 128.29, 128.24, 128.21, 128.13, 128.06, 128.00, 127.93, 127.81, 127.67, 127.60, 127.43, 127.16, 126.59, 114.91, 99.39, 98.21, 96.21, 81.07, 80.13, 79.96, 79.29, 76.15, 75.80, 74.98, 74.93, 73.54, 73.41, 73.33, 73.28, 71.02, 70.04, 69.34, 68.28, 68.20, 67.50, 66.83, 49.31, 30.31, 29.71, 28.67, 23.09, 18.07; HRMS (ESI): Calcd for $C_{74}H_{85}NO_{15}$ $[M+Na]^+$ 1250.5811, found: 1250.5823.

Pent-4-enyl-(2-acetamido-4-*O*-acetyl-3,6-di-*O*-benzyl -*β*-D-mannopyranosyl)-(1→4)-(2,3,6-tri-O-benzyl-*α*-D-glucopyranosyl)-(1→3)-2,4-di-*O*-benzyl-L-rhamnopyranoside (29)

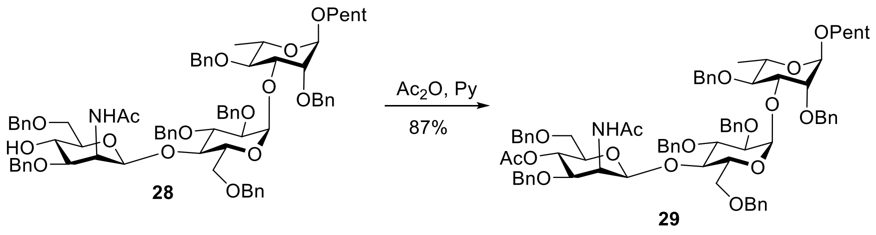

Acetic anhydride (8 *μ*L, 85.47 *μ*mol) was added to a solution of **28** (35 mg, 28.49 *μ*mol) in pyridine (1 mL) at 0 °C. After stirring at rt for 3h, the reaction was quenched with a few drops of methanol and concentrated in rotor under reduced pressure. The residue was purified by silica gel column chromatography using 30-40% ethyl acetate in hexane as eluents to obtain **29** (31 mg, 87%). $[\alpha]_D^{25}$ = +16.30 (c = 0.72, $CHCl_3$); ^{1}H NMR (600 MHz, $CDCl_3$) *δ* 7.35 – 7.22 (m, 8H, Ar), 7.22 – 7.13 (m, 16H, Ar), 7.11 (m, 5H, Ar), 7.06 (m, 2H, Ar), 5.71 (ddt, *J* = 16.9, 10.2, 6.6 Hz, 1H, C*H*=CH2), 5.52 (d, *J* = 9.6 Hz, 1H, NH), 5.04 (d, *J* = 3.6 Hz, 1H, H-1), 4.96 – 4.86 (m, 3H, H-4'', $PhCH_2$), 4.84 – 4.76 (m, 3H, $PhCH_2$), 4.73 (d, *J* = 12.0 Hz, 1H, PhCH*H*), 4.66 – 4.45 (m, 8H, $PhCH_2$, H-1'), 4.41 – 4.30 (m, 3H, $PhCH_2$, H-1''), 4.21 (dd, *J* = 16.3, 12.2 Hz, 2H), 4.02 – 3.85 (m, 4H), 3.76 (t, *J* = 2.5 Hz, 1H), 3.67 – 3.59 (m, 1H), 3.58 – 3.50 (m, 2H), 3.48 (dd, *J* = 9.4, 3.5 Hz, 1H), 3.41 – 3.35 (m, 1H), 3.30 – 3.21 (m, 4H), 3.11 – 3.01 (m, 2H), 2.03 – 1.96 (m, 2H), 1.91 – 1.85 (m, 3H), 1.61 (s, 3H, $NHCOCH_3$), 1.55 (p, *J* = 6.8 Hz, 3H), 1.24 (d, *J* = 6.2 Hz, 3H, CH_3); ^{13}C NMR (151 MHz, $CDCl_3$) δ 170.65, 169.86, 139.55, 138.84, 138.47, 138.27, 138.21, 138.17, 137.95, 128.67, 128.63, 128.61, 128.57, 128.47, 128.46, 128.44, 128.41, 128.36, 128.33, 128.31, 128.28, 128.16, 128.14, 128.00, 127.97, 127.85, 127.83, 127.81, 127.79, 127.77, 127.74, 127.58, 127.38, 126.88, 115.06, 99.21, 98.39, 96.49, 81.11, 80.09, 79.43, 77.88, 77.41, 76.38, 75.98, 75.22, 75.16, 74.01, 73.76, 73.62, 73.57, 73.46, 70.56, 70.20, 69.17, 68.46, 68.32, 68.28, 66.98, 49.55, 32.09, 31.61, 30.46, 30.37, 29.86, 29.82, 29.53, 28.83, 23.36, 22.86, 21.06, 18.22, 14.29; HRMS (ESI): Calcd for $C_{76}H_{87}NO_{16}$ $[M+Na]^+$ 1292.5917, found: 1292.5980.

2-Acetamido-4-*O*-acetyl-3,6-di-*O*-benzyl -*β*-D-mannopyranosyl-(1→4)-(2,3,6-tri-*O*-benzyl-α-D-glucopyranosyl)-(1→2)-3,4-di-*O*-benzyl-L-rhamnopyranose (30)

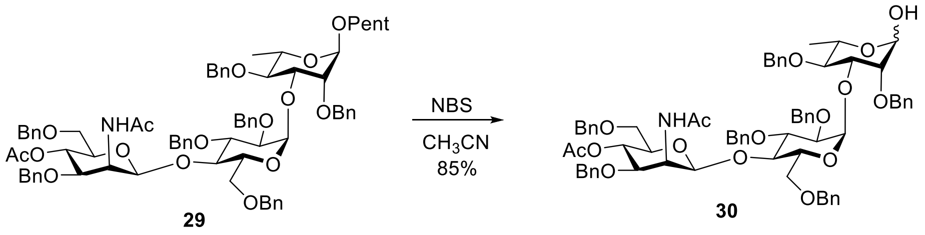

To a solution of **29** (35 mg, 27.54 *µ*mol) in acetonitrile (3 mL) was added NBS (6.37 mg, 35.8 *µ*mol) at rt. After 2 h stirring at rt, the reaction was diluted with EtOAc and quenched by addition aq. $NaHCO_3$. The combined organic extracts were washed with water, brine (10 mL), dried over Na_2SO_4, filtered and concentrated. The crude was purified by flash chromatography using hexane and ethyl acetate as eluent (7:3) to give the hemiacetal **30** as white semi solid (β : α = 1 : 2; 28 mg, 85%). ^{1}H NMR (600 MHz, $CDCl_3$) *δ* 7.35 – 7.29 (m, 6H, Ar), 7.26 – 7.10 (m, 42H, Ar), 7.06 (m, 4H, Ar), 5.61 (dd, *J* = 9.7 Hz, 1H, NH), 5.07 (m, 2H, H-1), 5.00 (m, 1H), 4.90 (t, *J* = 9.7 Hz, 1H), 4.87 – 4.70 (m, 7H), 4.68 – 4.47 (m, 14H), 4.42 (d, *J* = 11.4 Hz, 2H), 4.40 – 4.30 (m, 6H), 4.22 (td, *J* = 13.2, 11.5, 4.6 Hz, 5H), 4.05 (dd, *J* = 9.1, 2.8 Hz, 2H), 3.90 (m, 7H), 3.82 – 3.79 (m, 2H), 3.78 – 3.71 (m, 2H), 3.57 (q, *J* = 9.4, 7.9 Hz, 2H), 3.52 – 3.45 (m, 3H), 3.35 (m, 3H), 3.30 – 3.23 (m, 5H), 3.11 – 3.03 (m, 3H), 1.89 (s, 1H), 1.88 (s, 3H), 1.63 (s, 2H), 1.63 (s, 3H), 1.23 (d, *J* = 6.3 Hz, 5H). ^{13}C NMR (101 MHz, $CDCl_3$) *δ* 177.56, 170.74, 169.82, 139.39, 139.17, 138.60, 138.24, 138.20, 138.09, 138.06, 138.03, 137.95, 137.92, 137.87, 137.78, 137.65, 137.62, 128.54, 128.50, 128.47, 128.44, 128.38, 128.35, 128.34, 128.31, 128.27, 128.25, 128.23, 128.15, 128.07, 128.02, 127.95, 127.87, 127.83, 127.76, 127.72, 127.70, 127.68, 127.65, 127.62, 127.48, 127.33, 127.24, 126.74, 99.15, 99.10, 96.73, 96.21, 93.36, 93.06, 80.94, 80.88, 79.75, 79.17, 79.01, 76.98, 76.94, 76.75, 76.48, 76.21, 75.83, 75.81, 75.08, 75.06, 74.89, 74.74, 74.31, 73.86, 73.81, 73.58, 73.52, 73.45, 73.40, 71.64, 70.37, 70.13, 70.00, 68.88, 68.82, 68.57, 68.09, 68.05, 67.99, 67.96, 49.36, 49.30, 45.29, 31.96, 31.47, 30.20, 29.73, 29.69, 29.65, 29.55, 29.41, 29.40, 27.66, 23.20, 22.74, 21.52, 20.95, 18.29, 18.11, 14.18; HRMS (ESI): Calcd for $C_{71}H_{79}NO_{16}$ $[M+Na]^+$ 1224.5291, found: 1224.5259.

5-Azidopentyl-1-*O*-(2-acetamido-4-*O*-acetyl-3,6-di-*O*-benzyl-*β*-D-mannopyranosyl -(1→4)-(2,3,6-tri-*O*-benzyl-α-D-glucopyranosyl)-(1→3)-2,4-di-*O*-benzyl-L-rhamnopyranosyl phosphate) triethylammonium salt (31)

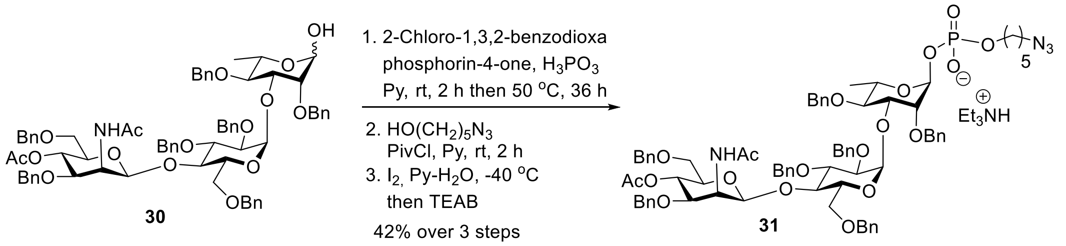

Compound **30** (25 mg, 20.79 *µ*mol) and phosphorous acid (5.11 mg, 62.37 *µ*mol) were mixed and co-evaporated with pyridine (3 x 1.0 mL). The dried reaction mixture was kept under high vacuum overnight. To a solution of premixed compound **30** and H_3PO_3 in anhydrous pyridine (2.0 mL) was added 2-chloro-5,5-dimethyl-2-oxo-1,3,2-dioxaphosphorinane (6.32 mg, 31.18 *µ*mol) at 0 °C. The ice bath was removed after 10 min and the reaction mixture was stirred for 2 h at rt. Then the reaction mixture was heated to 50 °C, and stirring was continued for 36 h. Then 1 M aq. triethylammonium bicarbonate (2 mL) was added to destroy excess reagent, and the mixture was diluted with dichloromethane, washed with 0.5 M aq. triethylammonium bicarbonate (TEAB), dried (Na_2SO_4), filtered and concentrated. The residue was purified by column chromatography (ethyl acetate–hexane (1:1 to 1:0) then dichloromethane–methanol (20:1) together with 1% trimethylamine as eluents. The product fractions were evaporated then was dissolved in dichloromethane and washed with 0.25 M aq. Triethylammonium hydrogen carbonate. The organic layer was separated, concentrated *in vacuo* to give H-phosphonate as foam (22 mg).

H-phosphonate (22 mg, 16.08 *µ*mol) and C5 azido linker (6.23 mg, 48.26 *µ*mol) were mixed and co-evaporated with pyridine (3 x 1.0 mL). The dried reaction mixture was kept under high vacuum for 30 min. The reaction mixture was dissolved in anhydrous pyridine (1.0 mL) and then pivaloyl chloride (4.95 *µ*L, 40.2 *µ*mol) was added. The reaction was stirred at rt for 2 h. Then the reaction was cooled to -40 °C, a freshly prepared solution of iodine (9.47 mg, 40.2 *µ*mol) in Py–H_2O 19 : 1 (0.1 mL) was added. After 15 min the mixture was diluted with dichloromethane, washed successively with 10% aq. sodium thiosulfate, 0.5 M aq. triethylammonium bicarbonate (TEAB), dried (Na_2SO_4), filtered and concentrated. The residue was purified by column chromatography (ethyl acetate–hexane (1:1 to 1:0) then dichloromethane–methanol (25:1) together with 1% trimethylamine as eluents. The product fractions were evaporated then dissolved in dichloromethane and washed with 0.25 M aq. triethylammonium hydrogen carbonate. The organic layer was separated,

concentrated and co-concentrated from dichloromethane (5 × 10 mL) to afford **31** as foam (13 mg, 42% over 3 steps). ^{1}H NMR (400 MHz, $CDCl_3$) *δ* 11.98 (s, 1H, NH), 7.33 – 7.28 (m, 7H, Ar), 7.17 (m, 26H, Ar), 7.10 (m, 6H, Ar), 5.52 (d, $^{3}J_{P,H-1}$ = 6.1 Hz, 1H, H-1), 5.47 (d, *J* = 9.6 Hz, 1H, NH), 4.99 (d, *J* = 3.6 Hz, 1H, H-1'), 4.89 (t, *J* = 9.7 Hz, 1H, H-4''), 4.83 – 4.71 (m, 5H, $PhCH_2$), 4.68 – 4.49 (m, 9H, $PhCH_2$), 4.45 (dd, *J* = 9.9, 4.2 Hz, 1H), 4.41 – 4.31 (m, 3H, $PhCH_2$, H-1''), 4.30 – 4.12 (m, 5H), 4.08 (dd, *J* = 9.7, 2.8 Hz, 1H), 3.98 – 3.75 (m, 10H), 3.63 – 3.54 (m, 2H), 3.47 (dd, *J* = 9.1, 3.5 Hz, 2H), 3.38 (dd, *J* = 10.7, 3.4 Hz, 2H), 3.29 – 3.17 (m, 3H), 3.17 – 3.04 (m, 6H), 3.04 – 3.00 (m, 1H), 3.00 – 2.93 (m, 10H), 1.89 (s, 3H), 1.58 (s, 3H, $NHCOCH_3$), 1.53 – 1.46 (m, 7H), 1.34 (m, 6H), 1.22 (d, *J* = 7.3 Hz, 23H); ^{13}C NMR (101 MHz, $CDCl_3$) *δ* 170.65, 169.88, 139.53, 138.88, 138.50, 138.26, 138.21, 138.12, 137.88, 128.82, 128.63, 128.60, 128.55, 128.49, 128.46, 128.44, 128.40, 128.29, 128.22, 128.12, 128.06, 128.00, 127.97, 127.80, 127.78, 127.73, 127.69, 127.67, 127.37, 126.79, 125.48, 125.07, 124.24, 122.74, 119.09, 111.30, 98.86, 95.58, 94.76, 81.13, 79.33, 79.08, 75.50, 75.20, 74.95, 73.91, 73.71, 73.54, 73.48, 73.36, 70.47, 69.88, 69.52, 69.06, 68.23, 68.16, 65.74, 51.47, 49.47, 45.71, 45.41, 32.08, 30.30, 29.86, 29.82, 29.53, 28.69, 27.78, 23.31, 23.15, 22.86, 21.65, 21.08, 18.27, 14.31, 8.69; ^{31}P NMR (162 MHz, $CDCl_3$): δ_P -2.64. HRMS (ESI): Calcd for $C_{76}H_{89}N_4O_{19}P$ $[M-Et_3N+H]^+$ 1393.5931, found: 1393.5940.

5-Azidopentyl-1-*O*-(2-acetamido-3,6-di-*O*-benzyl-*β*-D-mannopyranosyl-(1→4)-(2,3,6-tri-*O*-benzyl-α-D-glucopyranosyl)-(1→3)-2,4-di-*O*-benzyl-L-rhamnopyranosyl phosphate) triethylammonium salt (7)

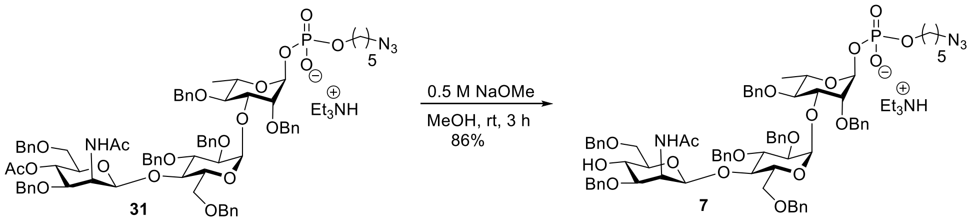

To a solution of **31** (12 mg, 86.11 *μ*mol) in a mixture of CH_2Cl_2 (0.5 mL) and MeOH (0.5 mL) was added a stock solution of 0.5 M NaOMe in methanol at rt and then the reaction mixture was stirred at rt for 3 h. Amberlite IR 120 H+ resin was added carefully maintaining the P^H and stirred for 4 min. The resin was filtered off and the filtrate was concentrated *in vacuo.* The crude was purified by flash chromatography using hexane and ethyl acetate as eluent (5:3) furnished **7** (10 mg, 86%). ^{1}H NMR (600 MHz, $CDCl_3$) *δ* 12.29 (s, 1H, NH), 7.33 – 7.25 (m, 7H, Ar), 7.24 – 6.99 (m, 22H, Ar), 5.51 (d, $^{3}J_{P,H-}$

$_1$= 7.7 Hz, 1H, H-1), 5.42 (d, *J* = 9.8 Hz, 1H, NH), 5.03 (s, 1H, H-1'), 4.81 (m, 2H, $PhCH_2$), 4.75 (m, 3H, $PhCH_2$), 4.54 (m, 5H), 4.42 (m, 3H), 4.35 (s, 1H), 4.20 (m, 2H), 4.10 (d, *J* = 9.8 Hz, 2H), 3.89 (m, 5H), 3.80 – 3.69 (m, 3H), 3.62 – 3.54 (m, 2H), 3.50 (m, 4H), 3.24 (d, *J* = 11.3 Hz, 2H), 3.14 (d, *J* = 32.6 Hz, 3H), 3.02 (d, *J* = 9.5 Hz, 1H), 2.94 (q, *J* = 7.7 Hz, 4H), 2.79 – 2.72 (m, 1H), 2.61 (t, *J* = 7.8 Hz, 1H), 2.14 (m, 2H), 2.00 – 1.89 (m, 2H), 1.54 (s, 3H), 1.48 (m, 4H), 1.22 – 1.17 (m, 9H); ^{13}C NMR (176 MHz, $CDCl_3$) δ 170.46, 139.66, 138.93, 138.55, 138.25, 138.21, 138.04, 137.98, 137.92, 129.20, 128.68, 128.66, 128.60, 128.56, 128.55, 128.50, 128.43, 128.39, 128.28, 128.23, 128.11, 128.08, 128.04, 127.96, 127.81, 127.76, 127.70, 127.68, 127.66, 127.37, 127.29, 126.72, 125.47, 99.29, 94.49, 81.21, 80.32, 79.42, 79.24, 75.68, 75.07, 73.68, 73.43, 73.37, 73.24, 71.17, 69.90, 69.49, 68.33, 67.67, 65.52, 57.84, 51.50, 49.46, 45.67, 45.51, 32.10, 30.37, 29.88, 29.84, 29.54, 29.50, 29.43, 28.73, 27.87, 23.20, 23.17, 22.87, 21.68, 21.63, 18.28, 14.30, 11.40, 11.24, 8.69, 8.16; ^{31}P NMR (162 MHz, $CDCl_3$): δ_P -2.25. HRMS (ESI): Calcd for $C_{74}H_{87}N_4O_{18}P$ [M-Et3N+Na]$^+$ 1373.5545, found: 1373.5643.

5-Azidopentyl-1-*O*-[2-acetamido-3,6-di-*O*-benzyl-*β*-D-mannopyranosyl-(1→4)-(2,3,6-tri-*O*-benzyl-α-D-glucopyranosyl)-(1→2)-3,4-di-*O*-benzyl-L-rhamnopyranosyl phosphate (2-acetamido-3-*O*-benzyl-4,6-*O*-benzylidene-β-D-mannopyranosyl-(1→4)-(2,3,6-tri-*O*-benzyl-α-D-glucopyranosyl)-(1→3)-2,4-di-*O*-benzyl-L-rhamnopyranosyl phosphate)] bis-triethylammonium salt (32)

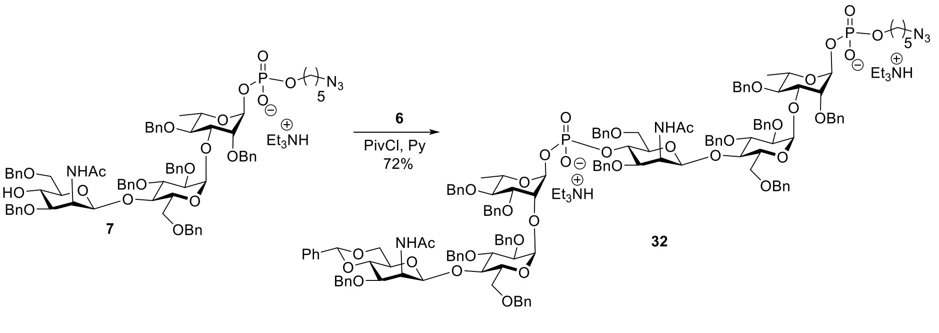

H-phosphonate **6** (7.05 mg, 5.78 *µ*mol) and acceptor **7** (6 mg, 4.44 *µ*mol) were mixed and co-evaporated with pyridine (3 x 1.0 mL). The dried reaction mixture was kept under high vacuum for 30 min. The reaction mixture was dissolved in anhydrous pyridine (1.0 mL) and then pivaloyl chloride (1.64 *µ*L, 13.32 *µ*mol) was added. The reaction was stirred at rt for 2 h. Then the reaction was cooled to -40 ^{o}C, a freshly prepared solution of iodine (2.7 mg, 11.2 *µ*mol) in Py–H_2O 19 : 1 (0.1 mL) was added. After 15 min the mixture was diluted with dichloromethane, washed successively with

10% aq. sodium thiosulfate, 0.5 M aq. triethylammonium bicarbonate (TEAB), dried (Na_2SO_4), filtered and concentrated. The residue was purified by column chromatography (ethyl acetate–hexane (1:1 to 1:0) then dichloromethane–methanol (25:1) together with 1% trimethylamine as eluents. The product fractions were evaporated then dissolved in dichloromethane and washed with 0.25 M aq. triethylammonium bicarbonate. The organic layer was separated, concentrated and co-concentrated from dichloromethane (5 × 10 mL) to afford bis-trirthylammonium salt **32** (8 mg, 72% over 2 steps). $[\alpha]_D^{25}$ = +153.51 (c = 0.14, $CHCl_3$); IR ν_{max} (film) 3408, 3062, 3033, 2926, 2855, 1753, 1371, 1224, 1070, 1042, 1024, 909, 804, 751, 734, 697 cm^{-1}; ^1H NMR (400 MHz, $CDCl_3$) δ ^1H NMR (400 MHz, $CDCl_3$) δ 11.80 (s, 6H), 7.45 – 7.39 (m, 4H), 7.35 – 7.22 (m, 31H), 7.19 – 7.03 (m, 36H), 5.66 – 5.59 (m, 1H), 5.54 – 5.45 (m, 6H), 5.41 (s, 1H), 5.06 (d, *J* = 3.8 Hz, 1H), 4.96 (d, *J* = 3.6 Hz, 1H), 4.95 – 4.87 (m, 2H), 4.80 – 4.50 (m, 15H), 4.48 – 4.32 (m, 10H), 4.22 (t, *J* = 2.4 Hz, 1H), 4.18 – 4.07 (m, 3H), 4.05 – 3.83 (m, 11H), 3.81 – 3.73 (m, 4H), 3.71 – 3.62 (m, 2H), 3.61 – 3.56 (m, 7H), 3.54 – 3.45 (m, 25H), 3.25 – 3.19 (m, 3H), 3.17 – 3.10 (m, 9H), 3.01 (q, *J* = 7.3 Hz, 61H), 1.94 (s, 4H), 1.91 (s, 3H), 1.51 (t, *J* = 8.4 Hz, 6H), 1.30 (t, *J* = 7.3 Hz, 92H); ^{13}C NMR (151 MHz, $CDCl_3$) δ 178.73, 170.56, 170.53, 140.03, 139.33, 138.65, 138.55, 138.41, 138.36, 138.03, 137.55, 129.10, 128.71, 128.68, 128.63, 128.57, 128.52, 128.48, 128.44, 128.39, 128.35, 128.33, 128.32, 128.27, 128.16, 128.12, 128.11, 128.05, 128.04, 128.00, 127.84, 127.80, 127.70, 127.62, 127.57, 127.53, 127.43, 127.39, 127.29, 127.26, 126.57, 126.51, 126.25, 101.76, 99.85, 99.79, 96.69, 96.66, 96.30, 96.16, 81.42, 80.23, 79.77, 79.45, 78.70, 77.41, 77.27, 77.20, 77.06, 76.99, 76.14, 76.09, 75.52, 75.42, 74.64, 73.52, 73.46, 72.81, 72.77, 72.37, 72.18, 72.16, 72.11, 71.30, 70.81, 69.12, 68.85, 68.50, 67.27, 65.43, 65.39, 64.14, 51.55, 51.43, 50.73, 45.54, 32.09, 32.07, 31.60, 30.48, 30.36, 30.31, 30.20, 29.93, 29.86, 29.82, 29.78, 29.68, 29.67, 29.52, 29.49, 29.39, 29.32, 28.77, 28.73, 28.66, 28.36, 27.39, 27.36, 23.38, 23.33, 23.29, 23.20, 22.86, 18.29, 18.21, 14.28, 8.77; ^{31}P NMR (243 MHz, $CDCl_3$) δ_P -3.61, -2.30. HRMS (ESI): Calcd for $C_{143}H_{161}N_5O_{35}P_2$ $[M\text{-}2Et_3N\text{+}H]^+$ 2571.0520, found: 2571.0596.

5-Aminopentyl-1-*O*-[(2-acetamido-2-deoxy-*β*-D-mannopyranosyl-(1→4)-(α-D-glucopyranosyl)-(1→2)-L-rhamnopyranosyl phosphate) (2-acetamido-2-deoxy-*β*-D-mannopyranosyl-(1→4)-(α-D-glucopyranosyl)-(1→3)-L-rhamnopyranosyl phosphate)] bis-sodium salt (5)

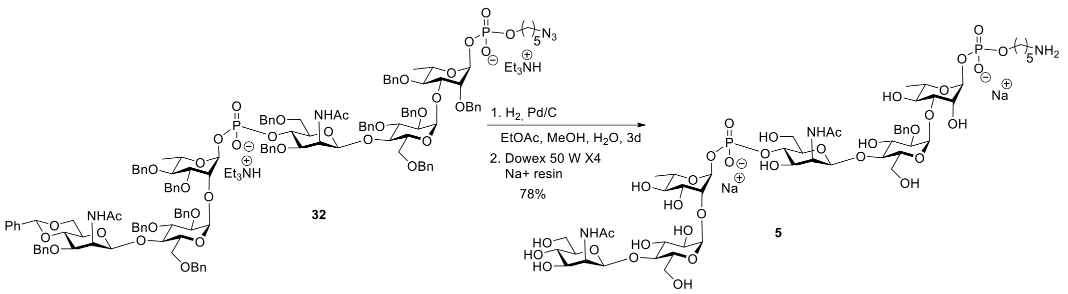

To a degased solution of compound **32** (5.0 mg, 18.39 μmol) in ethyl acetate-methanol-water (3:2:1; 2.2 mL) was added Pd/C (10 mg; 10%) and hydrogenolyzed using hydrogen balloon for three days. The mixture was filtered using a PTFE hydrophobic filter, and the residue was purified by gel filtration on a C18 column, using water–acetonitrile (95 : 5) as eluent. The obtained fractions were eluted with water through a column filled with Dowex 50W-X4 resin (Na+) and all the fractions were mixed together and lyophilized to obtain **5** as a white powder (1.93 mg, 78%). ^{1}H NMR (600 MHz, D_2O) δ 5.55 – 5.49 (d, $^{3}J_{P,H-1}$= 8.1 Hz, 1H, H-1 (α-Rha)), 5.39 (dd, $^{3}J_{P,H-1}$ = 7.8, $^{2}J_{H-1,H-2}$ = 2.1 Hz, 1H, H-1 (α-Rha)), 5.10 (d, J = 3.8 Hz, 1H, H-1' (α-Glc), 5.04 (d, J = 3.8 Hz, 1H, H-1' (α-Glc)), 4.93 (s, 1H, H-1'' (β-Man)), 4.90 (s, 1H, H-1'' (β-Man)), 4.61 (d, J = 4.1 Hz, 1H, H-2''), 4.57 (d, J = 4.5 Hz, 1H, H-2''), 4.17 (t, J = 2.7 Hz, 1H), 4.13 – 4.08 (m, 2H), 4.07 – 4.04 (m, 2H), 4.02 (dd, J = 9.4, 4.4 Hz, 1H), 3.97 – 3.88 (m, 10H), 3.86 – 3.82 (m, 3H), 3.79 – 3.74 (m, 6H), 3.68 – 3.52 (m, 8H), 3.47 (m, 2H), 3.04 (t, J = 7.5 Hz, 2H), 2.09 (2 x s, 6H, NHAc), 1.72 (m, 4H), 1.53 – 1.47 (m, 3H), 1.35 – 1.31 (m, 8H); ^{13}C NMR (151 MHz, D_2O) δ 175.42, 175.34, 99.22, 99.20, 97.62, 95.50, 95.49, 93.74, 78.50, 76.70, 76.42, 75.56, 75.30, 71.95, 71.62, 71.39, 71.13, 71.07, 71.01, 70.22, 70.06, 69.69, 69.59, 69.50, 69.37, 68.94, 67.31, 66.53, 65.98, 65.95, 60.29, 59.69, 59.59, 53.24, 53.12, 39.32, 29.08, 29.03, 26.24, 21.95, 16.82, 16.58; ^{31}P NMR (243 MHz, $CDCl_3$) δ_P -1.90, 2.53; HRMS (ESI): Calcd for $C_{45}H_{81}O_{35}N_3P_2$ $[M+H]^+$ 1286.4198, found: 1286.4193.

3. Conjugation of Synthetic Antigens 3, 4, 5 to CRM197

The saccharide ST19F **3** (1.76 mg, 1 eq.) was dissolved in 200 µL DMSO and 30 µL Et_3N was added in a vial. Disuccinimidyl adipate linker (8.35 mg, 100 eq.) was dissolved in 100 µL DMSO and the solution was slowly added to the saccharide solution. After 2.5 h stirring, the stirring bar was removed and the reaction mixture was frozen in liquid nitrogen. The solvent was completely evaporated *in vacuo* overnight

(lyophilizer). The residue was washed with chloroform (5x 1 mL) and DCM (3x 1 mL). The solid compounds were dissolved in DMSO and transferred to a 1.5 mL vial and lyophilized again.

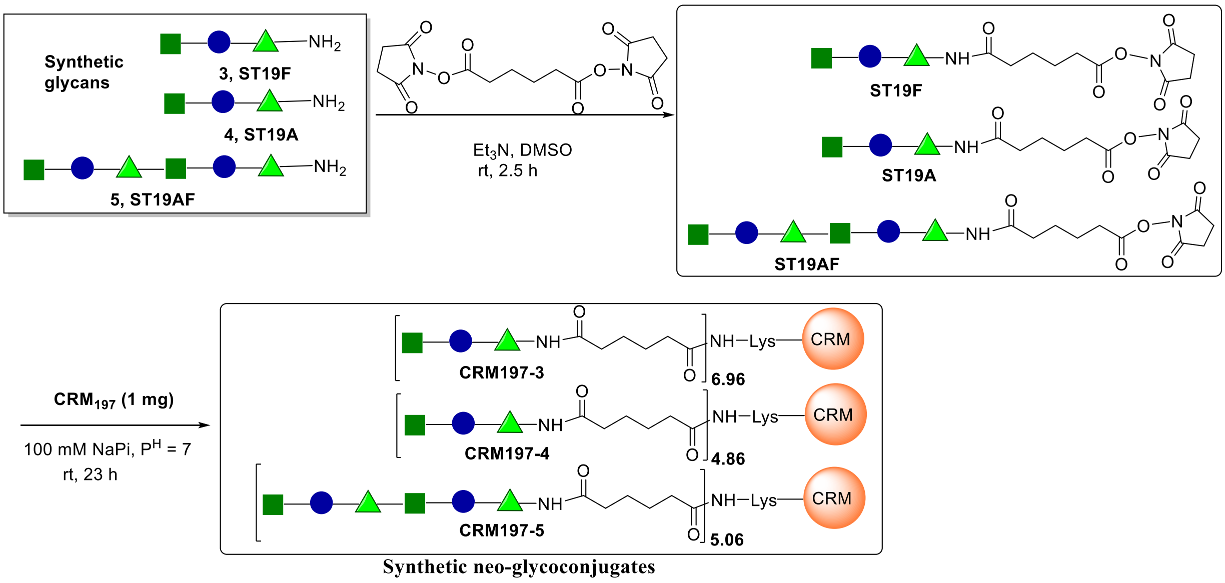

Fig. S1. Synthesis of neoglycoconjugates CRM197-**3**, CRM197-**4**, CRM197-**5**. Synthetic glycans **3**, **4**, **5** were covalently coupled with CRM197 using disuccinimidyl adipate ester as a coupling reagent.

Using an Amicon centrifugal 10k filter, CRM197 (1 mg) was washed with autoclaved water (500 µL, 2 x 300 µL) and 0.1 M phosphate buffer pH 7.0 (2 x 300 µL). After concentration, CRM197 in phosphate buffer pH 7.0 (total volume of 120 µL) was added to ST19F succinimidyl ester and stirred at room temperature for 23 h. After 23 h, the reaction mixture was washed with 10 mM sodium phosphate buffer pH 7.2, autocleaved water, and 0.1 M sodium phosphate buffer pH 7.

Similarly, synthetic glycans ST19F **4** (2.0 mg) and ST19AF **5** (1.9 mg) were conjugated to CRM197 using the synthetic procedure to afford the corresponding glycoconjugates. The average molecular size of the glycoconjugates was determined by matrix-assisted laser desorption/ionization (MALDI) analysis. Using CRM197 as standard and the average oligosaccharide attachment per CRM197 molecule (glycan loading) was calculated for ST19F, ST19A and ST19AF to be about 7, 5 and 5 respectively.

Charcterization of Glycoconjugates:

MALDI spectra of CRM197-**3** conjugate:

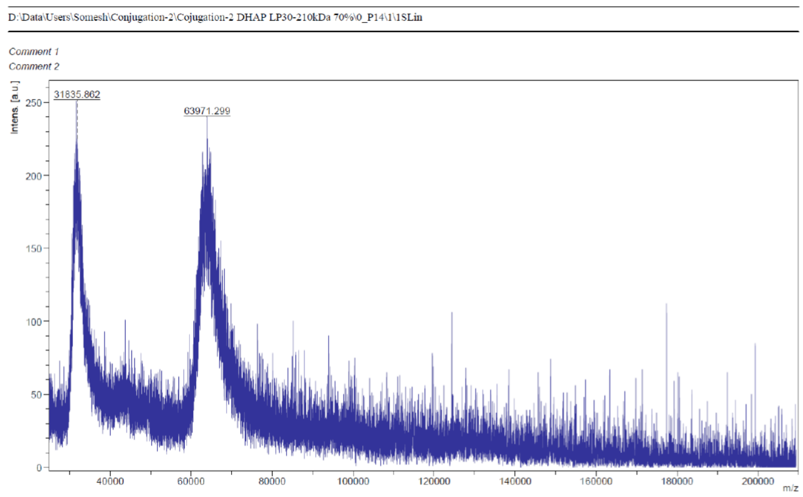

MALDI spectra of CRM197-**4** conjugate:

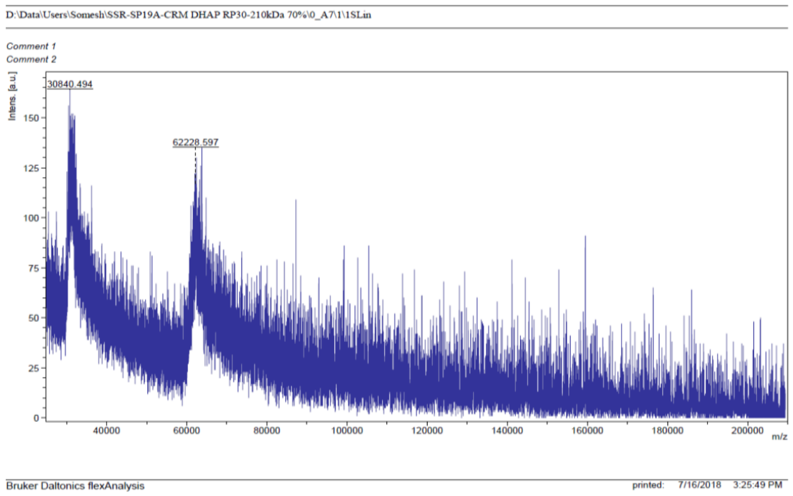

MALDI spectra of CRM197-**5** conjugate:

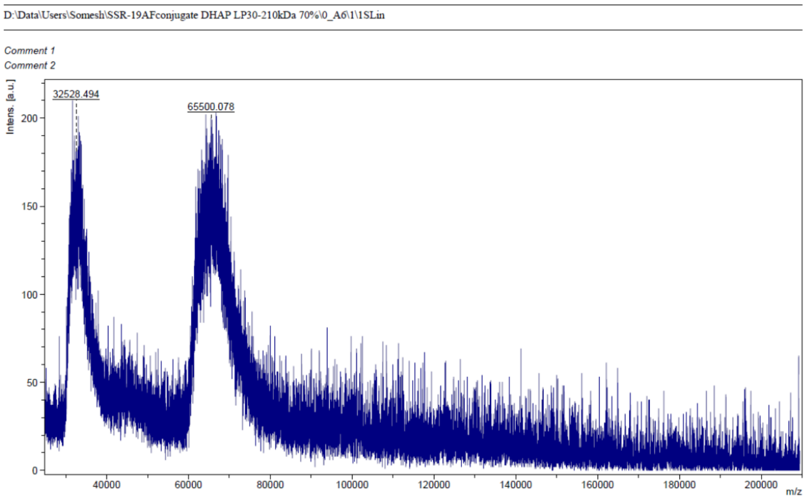

Fig. S2 MALDI-TOF analysis was used to determine the average molecular weight of the conjugate; CRM197 was used as a standard**.**

4. Vaccine Formulation

A total of 1µg of ST19A and ST19F and 2µg of ST19AF synthetic oligosaccharides already conjugated to CRM-197 were mixed with 125 µg of aluminum hydroxide ($Al(OH)_3$) (Alhydrogel, Brenntag, Denmark) and phosphate buffer (PBS, PAN-Biotech, Germany) was add up to 0.5 mL final volume. Vaccines were incubated overnight at 4°C with rotation in order to the antigen adsorb to the Alum matrix. The adsorption was confirmed by BSA assay (Micro BCA Protein Assay Kit, Thermo Scientific) based on the amount of protein in solution of the vaccines pre and post-incubation. As a negative vaccine control, only PBS formulated with 125µg of Alum was used while the marketed Prevnar 13® vaccine (Pfizer) was used as positive control.

5. Rabbit Immunizations

Five vaccine groups containing 10-12-week-old female Zika rabbits (n=5 per group) approved by the Landesamt für Landwirtschaft, Lebensmittelsicherheit und Fischerei Mecklenburg-Vorpommern, were immunized intramuscularly (i.m.) with 0.5 mL of the following vaccines: CRM-ST19A, CRM-ST19F, CRM-ST19AF, Prevnar13®, PBS-Alum. The immunizations were performed on day 0 (primary immunization) and boosted with the same vaccine formulation on days 14, 28, and 133. Blood was collected at days 0 (pre-immune), 14, 21, 35, 133, and 144 (terminal bleed) for hyper-immune sera. The antibody response was analyzed by ELISA and glycan microarray.

6. Enzyme-Linked Immunosorbent Assay (ELISA)

Blood collected from immunization time points were separated by centrifugation (1800 x g, 10 min) and the sera were used to assess the antibody titer against native capsular polysaccharide by ELISA. High binding 96-well polystyrene micro titer plates (Corning, USA) were coated overnight at 4°C with 50µL of the different CPSs (10µg/mL) in PBS (pH 7.4). The plates were washed three times with PBST (PBS + 0.1% Tween-20) and blocked with 2% BSA-PBS at RT for 1 h. After one wash with PBST, the plates were incubated with serial dilutions of rabbit serum in triplicate at RT for 1 h. The plates were washed with PBST and incubated with HRP conjugated goat anti-rabbit IgG antibodies (Sigma-Aldrich) diluted 1:10,000 in 1% BSA-PBS followed by incubation at RT for 1 h. The plate was washed thoroughly with PBST and developed using HRP

substrate 3,3’,5,5’-tetramethylbenzidine (BD Biosciences, United States). The reaction was stopped by quenching with 2% H_2SO_4 and the absorbance was recorded at 450 nm with an ELISA plate reader instrument (Infinite® 200 NanoQuant, Tecan, Switzerland). The statistical analysis was carried out in R studio software (Rstudio version 1.1.423) and GraphPad Prism 8.

7. Glycan Microarrays

Glass microarray slides (CodeLink, Surmodics™) with specific immobilized oligosaccharides were prepared as described previously[5], quenched, and stored at 4º C until use. Prior to sera incubation, the slides were blocked with 1% BSA-PBS, a FlexWell 64 grid was applied and the slides were incubated with serial dilutions of rabbit serum in duplicate, diluted in 1% BSA-PBS (w/v) in a humidifying chamber for 1 h at room temperature and washed three times with PBST. Further, the slides were incubated with a fluorescently labeled secondary antibody, goat anti- rabbit IgG Alexa Fluor® 635 (1:400 dilution), in a humidifying chamber for 1 h at room temperature, washed three times with PBST, rinsed with deionized water and dried by centrifugation (300 x g, 5 min). Slides were scanned with a GenePix 4300A microarray scanner (Molecular Devices, Sunnyvale, CA, USA). Image analysis was carried out with the GenePix Pro 7 software (Molecular Devices). The photomultiplier tube (PMT) voltage was adjusted such that scans were free of saturation signals. Only the 1:100 dilutions were used in the analysis due to best signal to noise ratio l. The statistical analysis was carried out in R studio software (Rstudio version 1.1.423) using the mean fluorescence intensity values subtracted from background noise.

Rabbit#1 Day 0 ST19AF	Rabbit#2 Day 28 ST19AF	Rabbit#3 Day 144 ST19AF	Rabbit#3 Day 21 Prevnar13
Rabbit#4 Day 14 ST19AF	Rabbit#5 Day 35 ST19AF	Rabbit#5 Day 0 Prevnar13	Rabbit#1 Day 35 Prevnar13
Rabbit#2 Day 0 ST19AF	Rabbit#3 Day 28 ST19AF	Rabbit#4 Day 144 ST19AF	Rabbit#4 Day 21 Prevnar13
Rabbit#5 Day 14 ST19AF	Rabbit#1 Day 133 ST19AF	Rabbit#1 Day 14 Pevnar13	Rabbit#2 Day 35 Prevnar13
Rabbit#3 Day 0 ST19AF	Rabbit#4 Day 28 ST19AF	Rabbit#5 Day 144 ST19AF	Rabbit#5 Day 21 Prevnar13
Rabbit#1 Day 21 ST19AF	Rabbit#2 Day 133 ST19AF	Rabbit#2 Day 14 Prevnar13	Rabbit#3 Day 35 Prevnar13
Rabbit#5 Day 0 ST19AF	Rabbit#5 Day 28 ST19AF	1% BSA in PBS	Rabbit#1 Day 28 Prevnar13
Rabbit#3 Day 21 ST19AF	Rabbit#3 Day 133 ST19AF	Rabbit#3 Day 14 Prevnar13	Rabbit#4 Day 35 Prevnar13
Rabbit#1 Day 14 ST19AF	Rabbit#1 Day 35 ST19AF	Rabbit#1 Day 0 Prevnar13	Rabbit#2 Day 28 Prevnar13
Rabbit#4 Day 21 ST19AF	Rabbit#4 Day 133 ST19AF	Rabbit#4 Day 14 Prevnar13	Rabbit#5 Day 35 Prevnar13
Rabbit#1 Day 14 ST19AF	Rabbit#2 Day 35 ST19AF	Rabbit#2 Day 0 Prevnar13	Rabbit#3 Day 28 Prevnar13
Rabbit#4 Day 21 ST19AF	Rabbit#5 Day 133 ST19AF	Rabbit#5 Day 14 Prevnar13	Rabbit#1 Day 133 Prevnar13
Rabbit#2 Day 14 ST19AF	Rabbit#3 Day 35 ST19AF	Rabbit#3 Day 0 Prevnar13	Rabbit#4 Day 28 Prevnar13
Rabbit#5 Day 21 ST19AF	Rabbit#1 Day 144 ST19AF	Rabbit#1 Day 21 Prevnar13	Rabbit#2 Day 133 Prevnar13
Rabbit#3 Day 14 ST19AF	Rabbit#4 Day 35 ST19AF	Rabbit#4 Day 0 Prevnar13	Rabbit#5 Day 28 Prevnar13
Rabbit#1 Day 28 ST19AF	Rabbit#2 Day 144 ST19AF	Rabbit#2 Day 21 Prevnar13	Rabbit#3 Day 133 Prevnar13

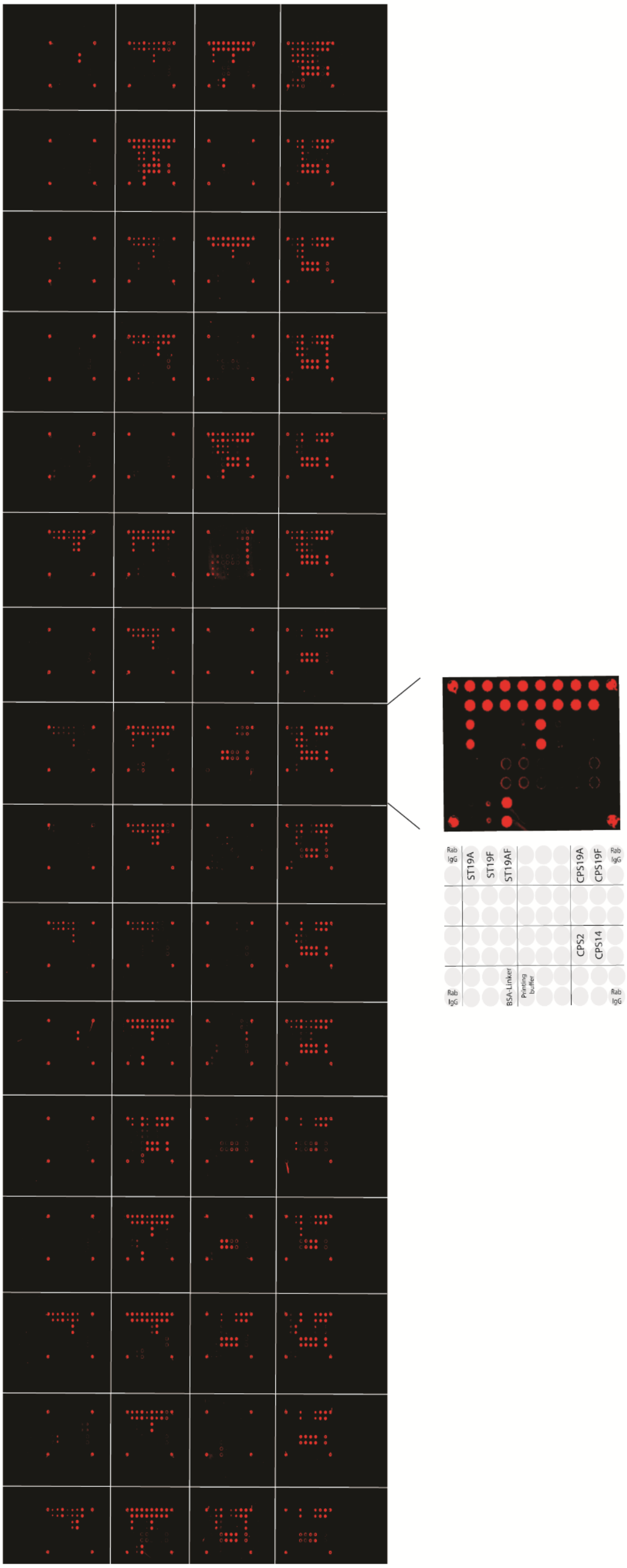

Rabbit#4<br>Day 133<br>Prevnar13	Rabbit#4<br>Day 14<br>ST19A	Rabbit#5<br>Day 35<br>ST19A	Rabbit#5<br>Day 0<br>ST19F
Rabbit#1<br>Day 0<br>ST19A	Rabbit#2<br>Day 28<br>ST19A	Rabbit#3<br>Day 144<br>ST19A	Rabbit#3<br>Day 21<br>ST19F
Rabbit#5<br>Day 133<br>Prevnar13	Rabbit#5<br>Day 14<br>ST19A	Rabbit#1<br>Day 133<br>ST19A	Rabbit#1<br>Day 14<br>ST19F
Rabbit#2<br>Day 0<br>ST19A	Rabbit#3<br>Day 28<br>ST19A	Rabbit#4<br>Day 144<br>ST19A	Rabbit#4<br>Day<br>ST19F
Rabbit#1<br>Day 144<br>Prevnar13	Rabbit#1<br>Day 21<br>ST19A	Rabbit#2<br>Day 133<br>ST19A	Rabbit#2<br>Day 14<br>ST19F
Rabbit#3<br>Day 0<br>ST19A	Rabbit#4<br>Day 28<br>ST19A	Rabbit#5<br>Day 144<br>ST19A	Rabbit#5<br>Day 21<br>ST19F
Rabbit#2<br>Day 144<br>Prevnar13	Rabbit#2<br>Day 21<br>ST19A	Rabbit#3<br>Day 133<br>ST19A	Rabbit#3<br>Day 14<br>ST19F
Rabbit#4<br>Day 0<br>ST19A	Rabbit#5<br>Day 28<br>ST19A	1% BSA<br>in<br>PBS	Rabbit#1<br>Day 28<br>ST19F
Rabbit#3<br>Day 144<br>Prevnar13	Rabbit#3<br>Day 21<br>ST19A	Rabbit#4<br>Day 133<br>ST19A	Rabbit#4<br>Day 14<br>ST19F
Rabbit#5<br>Day 0<br>ST19A	Rabbit#1<br>Day 35<br>ST19A	Rabbit#1<br>Day 0<br>ST19F	Rabbit#2<br>Day 28<br>ST19F
Rabbit#4<br>Day 144<br>Prevnar13	Rabbit#4<br>Day 21<br>ST19A	Rabbit#5<br>Day 133<br>ST19A	Rabbit#5<br>Day 14<br>ST19F
Rabbit#1<br>Day 14<br>ST19A	Rabbit#2<br>Day 35<br>ST19A	Rabbit#2<br>Day 0<br>ST19F	Rabbit#3<br>Day 28<br>ST19F
Rabbit#5<br>Day 144<br>Prevnar13	Rabbit#5<br>Day 21<br>ST19A	Rabbit#1<br>Day 144<br>ST19A	Rabbit#1<br>Day 21<br>ST19F
Rabbit#2<br>Day 14<br>ST19A	Rabbit#3<br>Day 35<br>ST19A	Rabbit#3<br>Day 0<br>ST19F	Rabbit#4<br>Day 28<br>ST19F
1% BSA<br>in<br>PBS	Rabbit#1<br>Day 28<br>ST19A	Rabbit#2<br>Day 144<br>ST19A	Rabbit#2<br>Day 21<br>ST19F
Rabbit#3<br>Day 14<br>ST19A	Rabbit#4<br>Day 35<br>ST19A	Rabbit#4<br>Day 0<br>ST19F	Rabbit#5<br>Day 28<br>ST19F

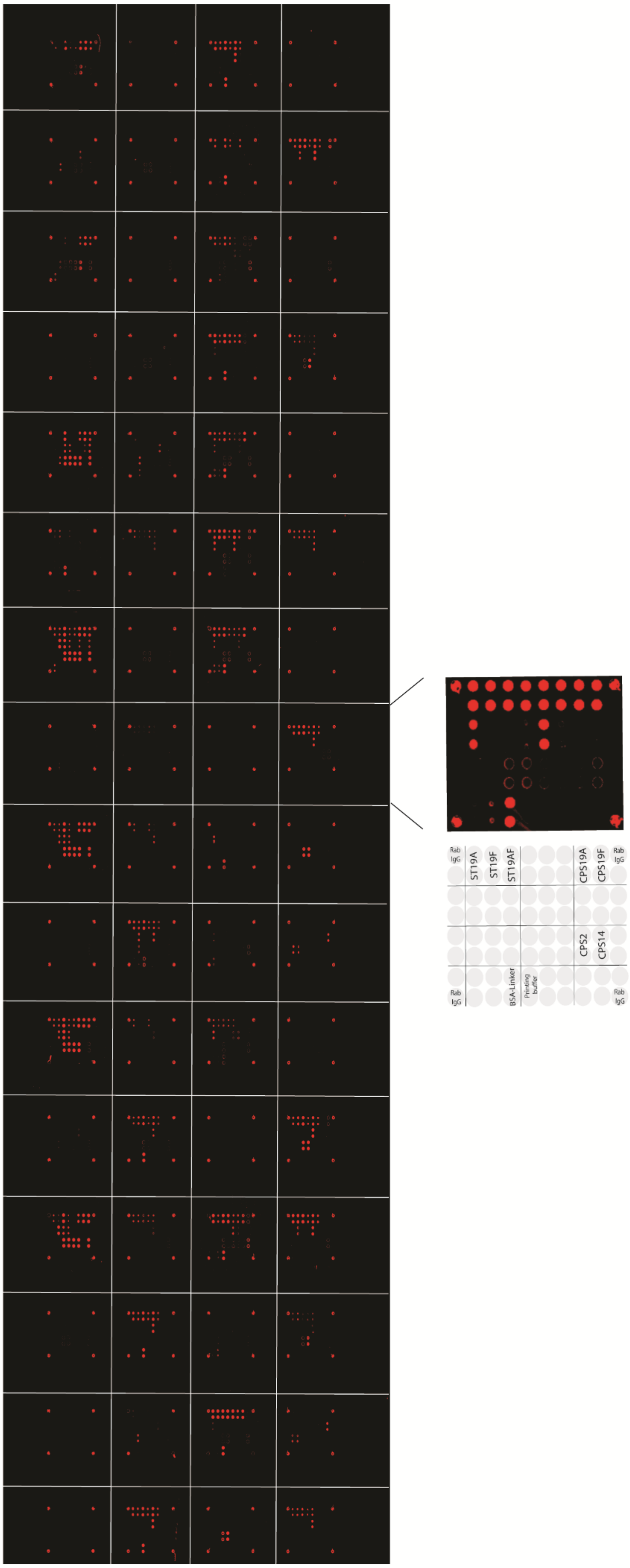

Rabbit#1 Day 35 ST19F	Rabbit#1 Day 0 PBS	Rabbit#2 Day 28 PBS	Rabbit#5 Day 144 PBS
Rabbit#4 Day 133 ST19F	Rabbit#4 Day 14 PBS	Rabbit#5 Day 35 PBS	BLANK 2ary anti-Rab IgG
Rabbit#2 Day 35 ST19F	Rabbit#2 Day 0 PBS	Rabbit#3 Day 28 PBS	sp 007 CTRL 1:10
Rabbit#5 Day 133 ST19F	Rabbit#5 Day 14 PBS	Rabbit#1 Day 133 PBS	BLANK 2ary anti-Rab IgG
Rabbit#3 Day 35 ST19F	Rabbit#3 Day 0 PBS	Rabbit#4 Day 28 PBS	Rabbit#5 Day 144 PBS
Rabbit#1 Day 144 ST19F	Rabbit#1 Day 21 PBS	Rabbit#2 Day 133 PBS	BLANK 2ary anti-Rab IgG
Rabbit#4 Day 35 ST19F	Rabbit#4 Day 0 PBS	Rabbit#5 Day 28 PBS	1% BSA in PBS
Rabbit#2 Day 144 ST19F	Rabbit#2 Day 21 PBS	Rabbit#3 Day 133 PBS	Rabbit#4 Day 144 PBS
Rabbit#5 Day 35 ST19F	Rabbit#5 Day 0 PBS	Rabbit#1 Day 35 PBS	sp 007 CTRL 1:50
Rabbit#3 Day 144 ST19F	Rabbit#3 Day 21 PBS	Rabbit#4 Day 133 PBS	BLANK
Rabbit#1 Day 133 ST19F	Rabbit#1 Day 14 PBS	Rabbit#2 Day 35 PBS	BLANK 2ary anti-Rab IgG
Rabbit#4 Day 144 ST19F	Rabbit#4 Day 21 PBS	Rabbit#5 Day 133 PBS	BLANK
Rabbit#2 Day 133 ST19F	Rabbit#2 Day 14 PBS	Rabbit#3 Day 35 PBS	BLANK 2ary anti-Rab IgG
Rabbit#5 Day 144 ST19F	Rabbit#5 Day 21 PBS	Rabbit#1 Day 144 PBS	BLANK
Rabbit#3 Day 133 ST19F	Rabbit#3 Day 14 PBS	Rabbit#4 Day 35 PBS	BLANK 2ary anti-Rab IgG
Rabbit#2 Day 144 PBS	Rabbit#1 Day 28 PBS	sp 007 CTRL 1:100	BLANK

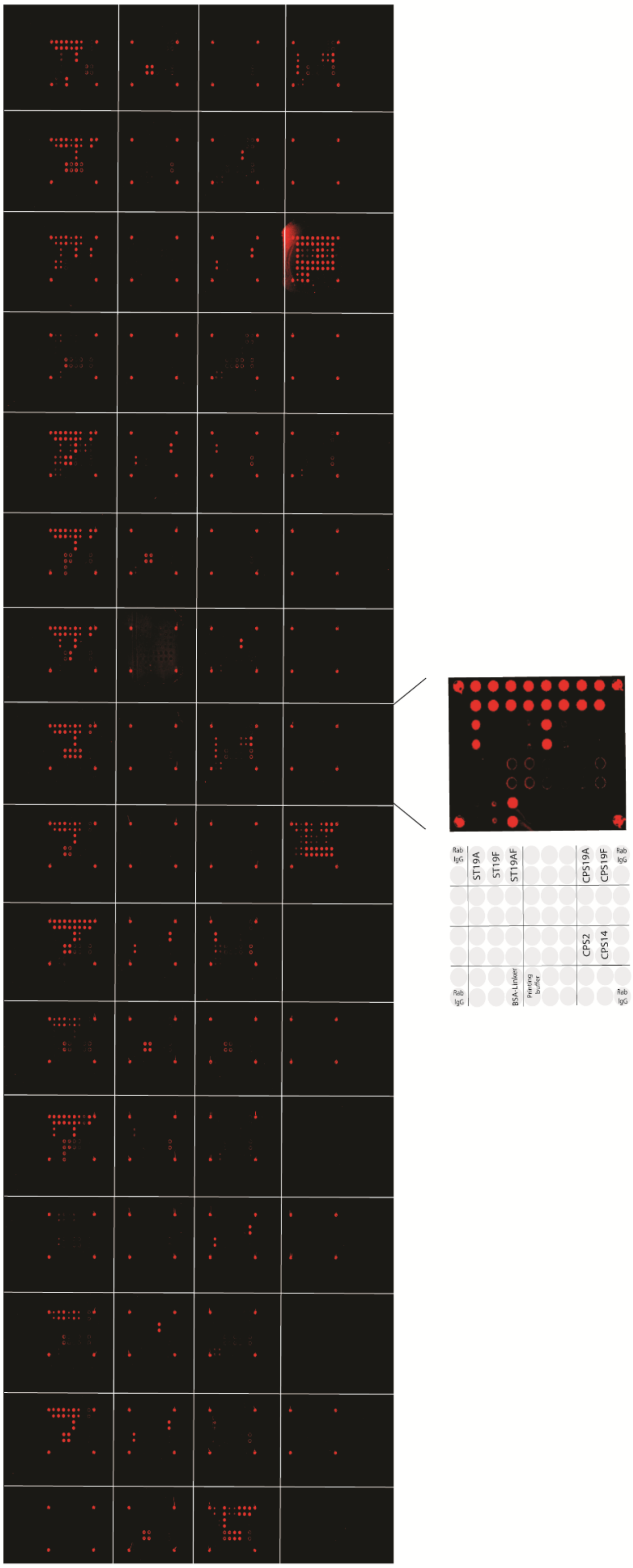

Fig. S3 Glycan microarray printing pattern. Sera from rabbits immunized with constructs ST19A, ST19F, and ST19AF were incubated with specific synthetic glycans, positive CPSs and negative CPSs controls printed on a glass slide. The dilution of 1:100 was used in the analysis. The reference serum 007 sp. (WHO007) was used as positive control and buffer (PBS-BSA 1%) as negative control. Printed rabbit IgG was used for anti-rabbit secondary antibody control.

8. In vitro Opsonophagocytic Killing Assay (OPKA).

The assay was performed as described previously[6]. Concisely, the effector HL-60 cell line (a human origin leukemia cell line) was used as phagocytic cell. For granulocyte differentiation, approximately 4 × 10^5 cells/mL were seeded in tissue culture flasks (Corning, N.Y.) in complete medium (90% RPMI 1640, 10% FCS, 1 mM L-glutamine and penicillin-streptomycin solution; PAN Biotech, Germany) containing 0.8% N,N-dimethylformamide (DMF; 99.8% purity; Fisher Scientific, Fair Lawn, N.J.) for 5-6 days at 37°C in the presence of 5% CO_2. After differentiation, the cells were harvested by centrifugation (300 × g, 5 min) and then viable cells were counted by using 1% trypan blue exclusion and resuspended in opsonophagocytic buffer (HBSS with Ca^{2+} and Mg^{2+}, 0.1% gelatin, and 10% FBS; HyClone) at a density of 1 x 10^7 cells/mL. For the opsonophagocytic killing assay, a ratio of 400:1 effector to target cells was used. Glycerol stock of *S. pneumoniae* serotypes 19A and 19F grown to mid-log phase (OD_{600}= 0.2–0.3) were gently thawed and diluted in opsonophagocytic buffer to a final density of 1000 CFU per 20 µL. Pooled heat inactivated (56°C, 30 min) rabbit sera samples (10 µL) were aliquoted in round bottom non-treated 96-well plates in triplicate at four-fold dilution intervals. The bacterial suspension (20 µL) was added to each well and incubated for 15 min at 37°C. Then 10 µL of baby rabbit complement (10% (v/v), rabbit complement, Cedarlane) and 4 × 10^5 differentiated HL-60 cells (in 40 µL) were added to each well. The plates were incubated for 45 min at 37°C in 5% CO2 with intermittent shaking. The phagocytic reaction was stopped by putting the plate on ice for 15 min. Viable extracellular pneumococci were determined by plating aliquots (5 µL) from each well on Columbia Agar plates with 5% (v/v) sheep blood and incubating at 37°C in 5% CO2. CFUs were counted after overnight growth. Negative control containing only bacteria, complement, HL-60 cells, and buffer was used to calculate the percent killing of pneumococci. The assay was repeated two times independently and in triplicates each. The percent killing was calculated as means ± SD of CFU. Opsonic index values where 50% bacterial killing occurs was obtained from four parameters logistic regression of individual opsonic curves[7].

9. Spectra

1H NMR, 400 MHz, $CDCl_3$

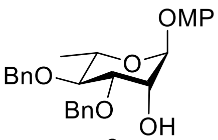

8

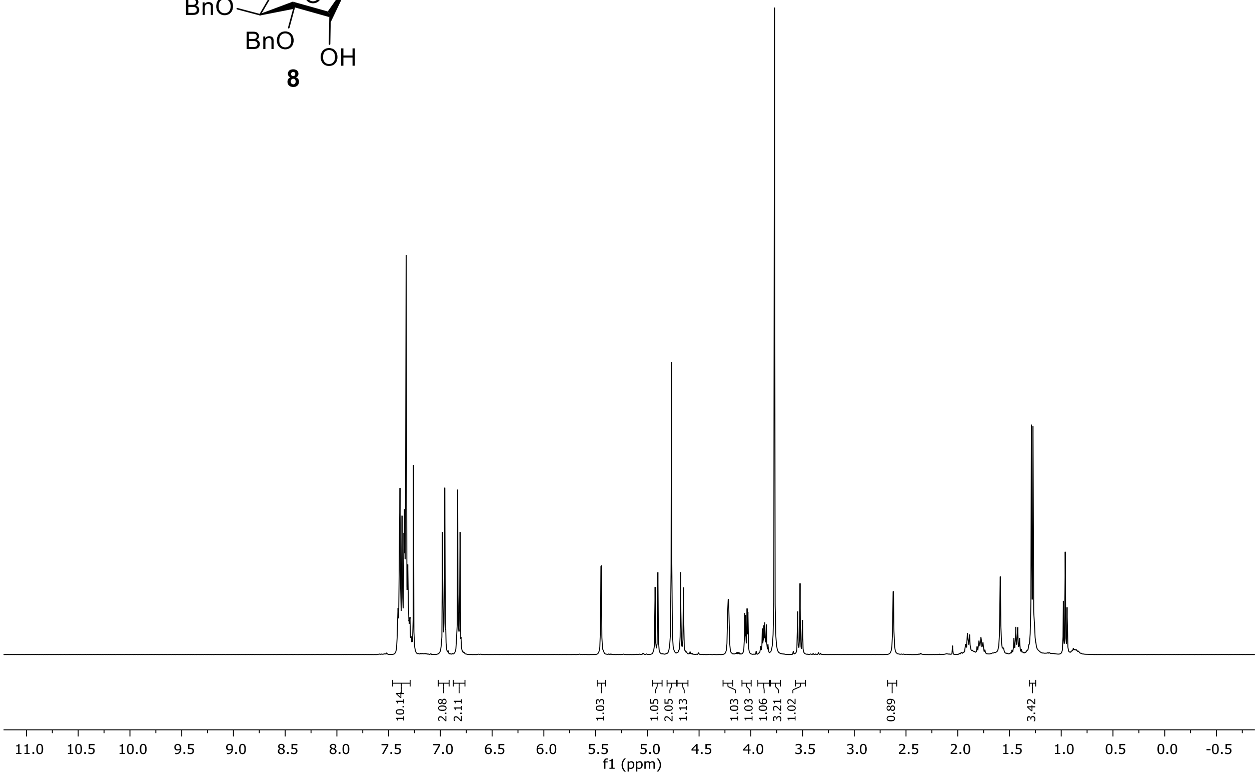

13C NMR, 101 MHz, $CDCl_3$

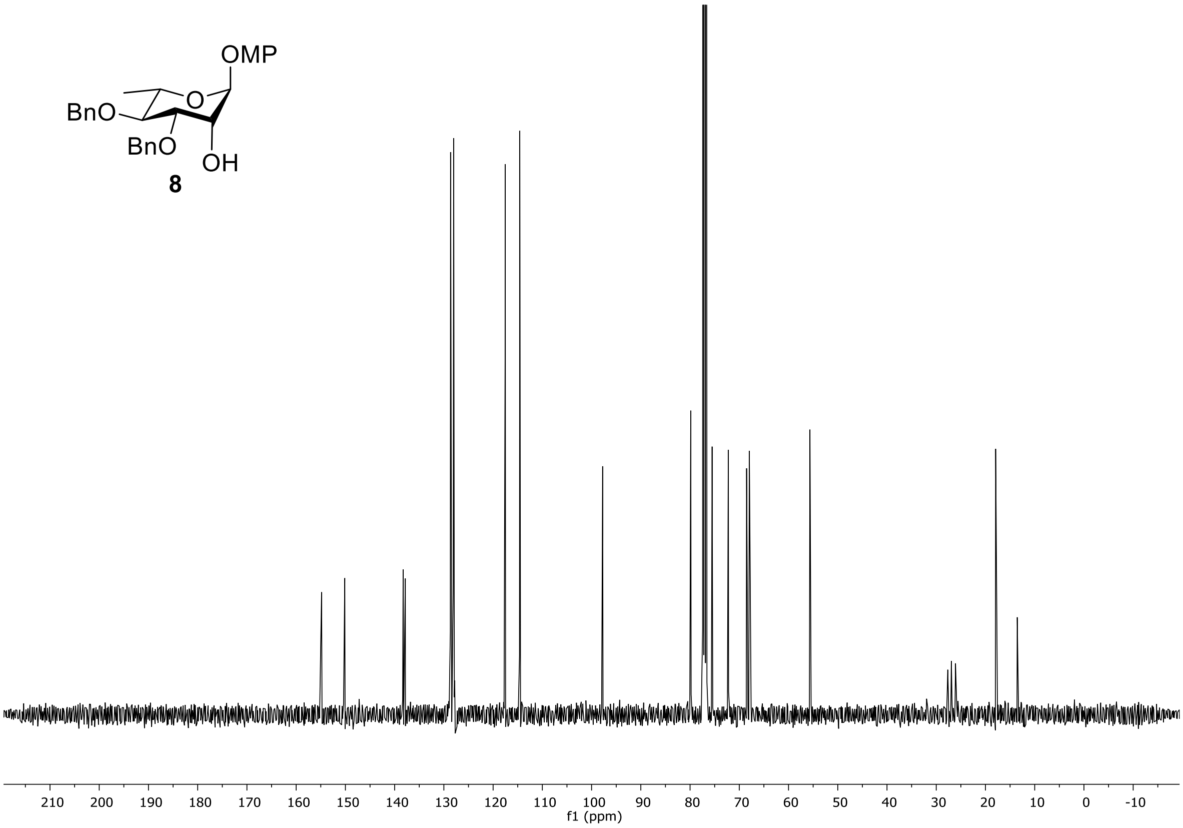

HH-COSY NMR, 600 MHz, $CDCl_3$

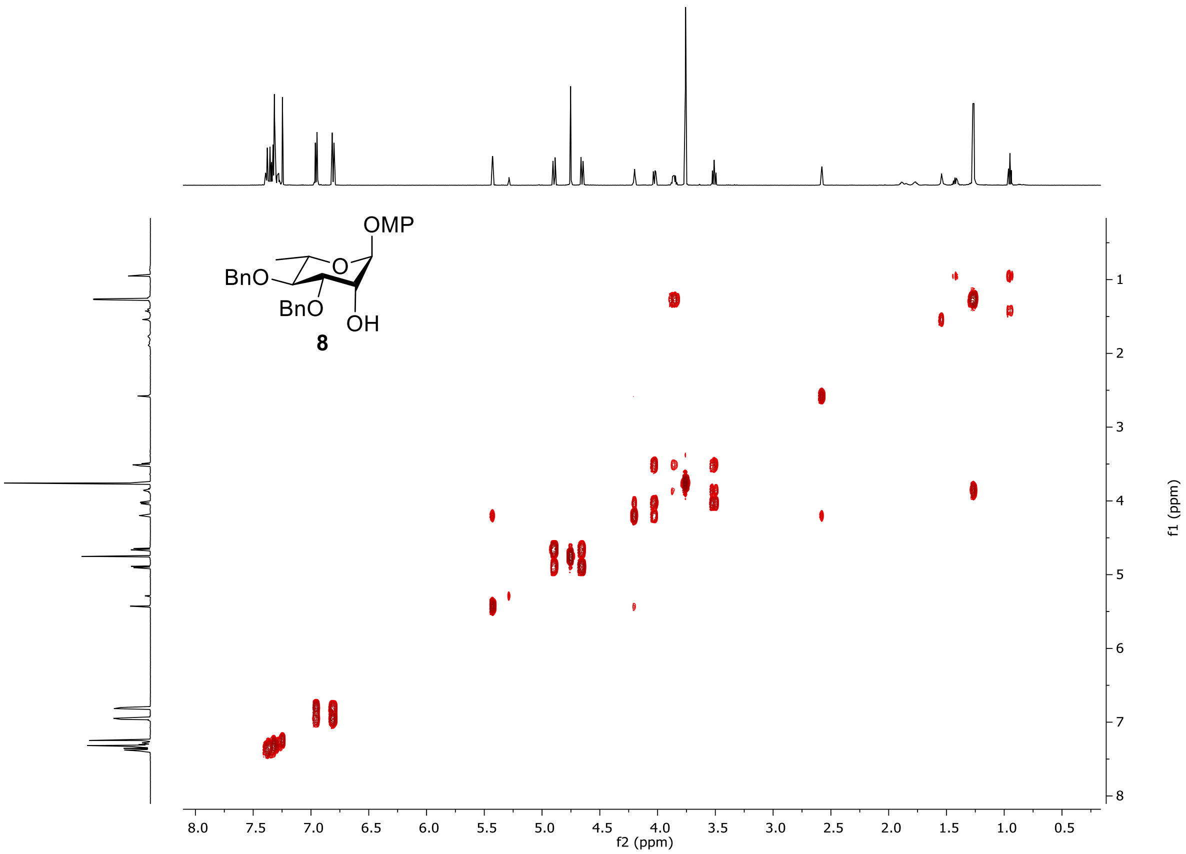

1H NMR, 400 MHz, $CDCl_3$

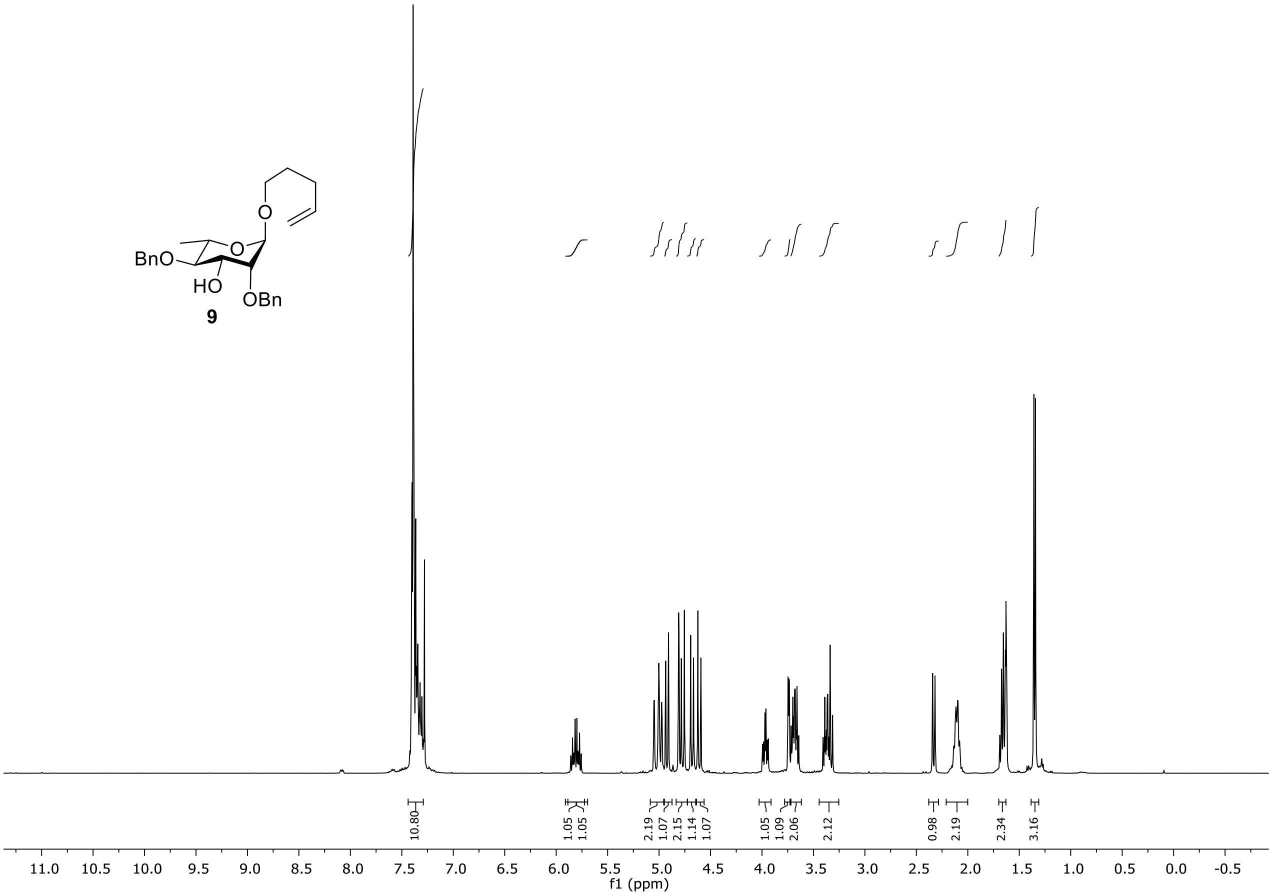

13C NMR, 101 MHz, CDCl3

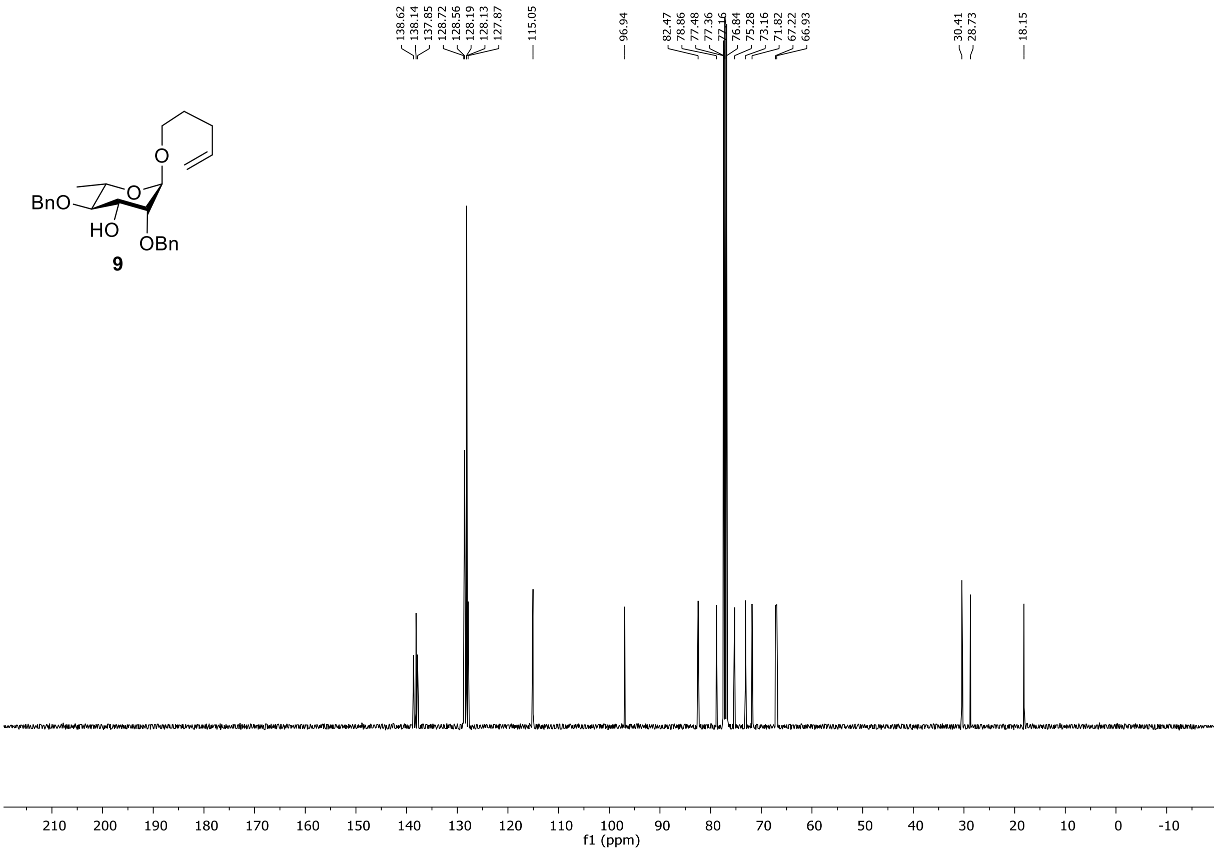

HH-COSY NMR, 400 MHz, CDCl3

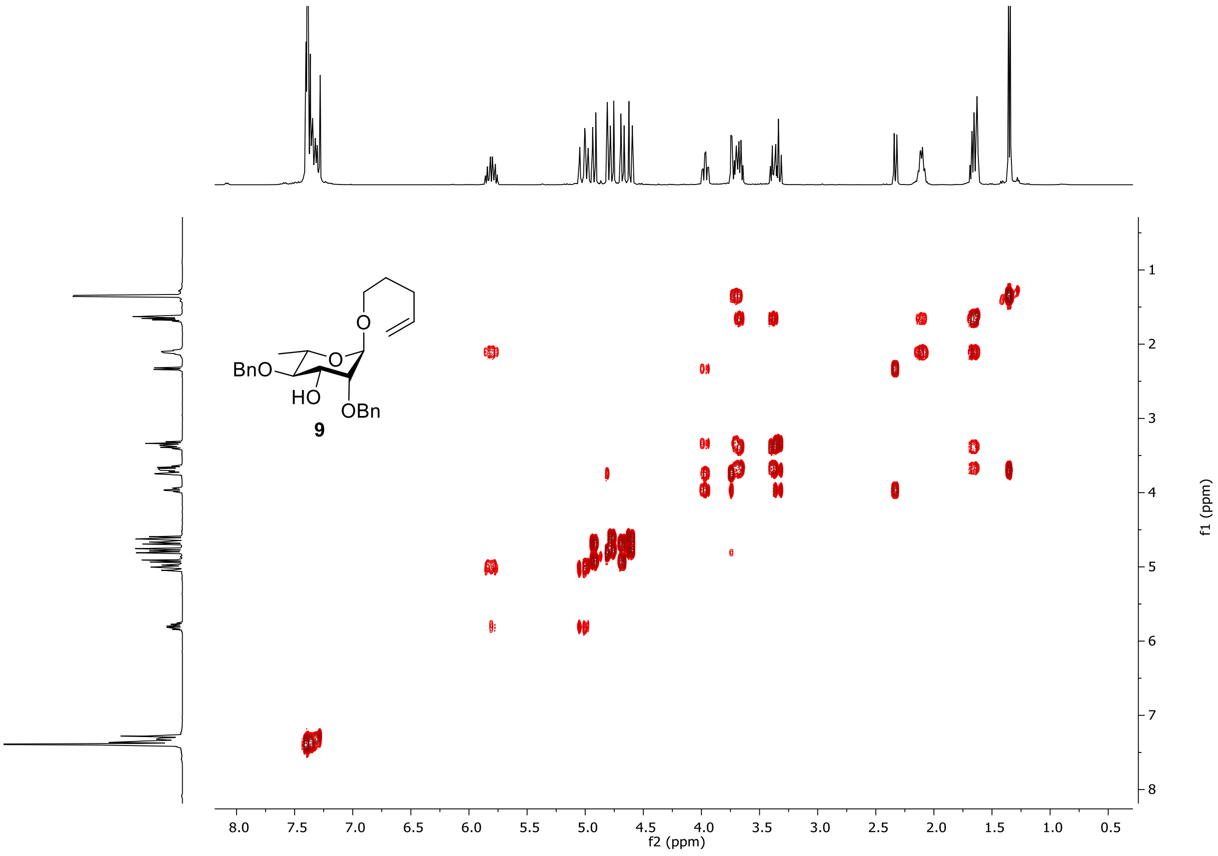

1H NMR, 400 MHz, $CDCl_3$

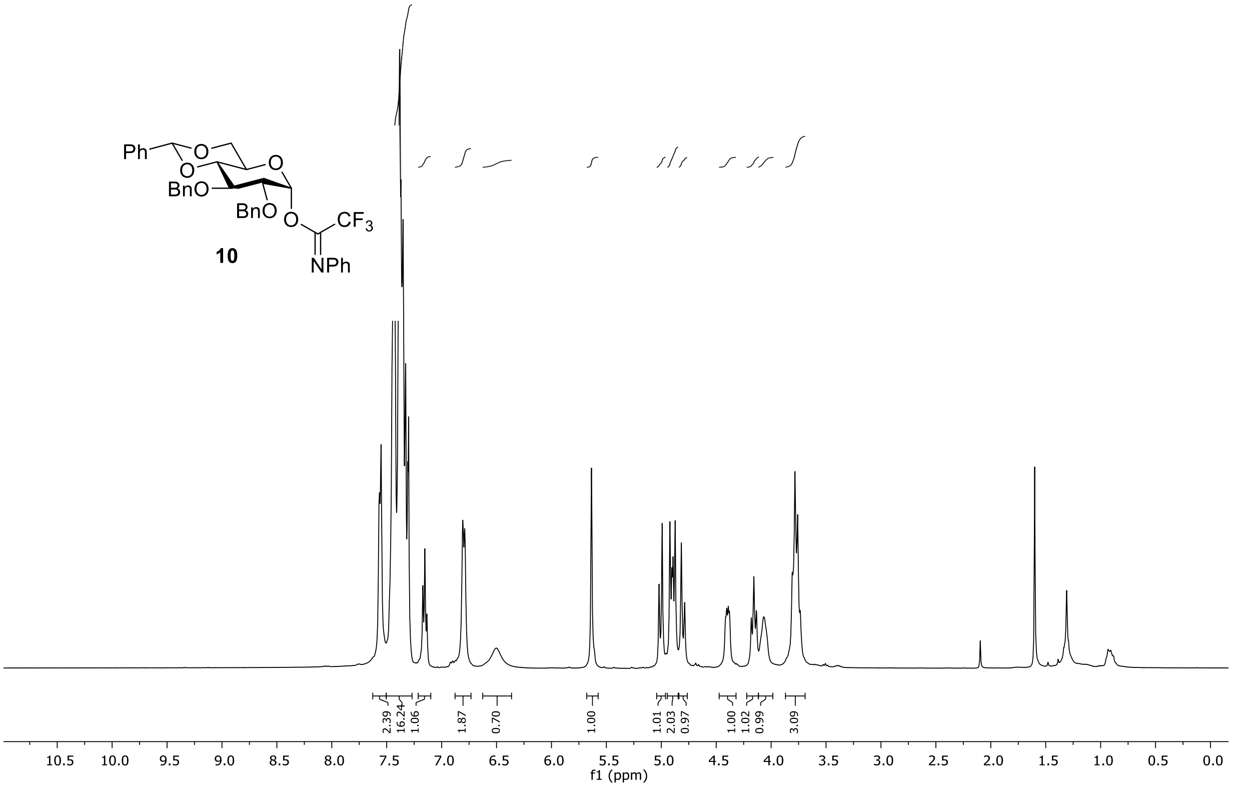

13C NMR, 101 MHz, $CDCl_3$

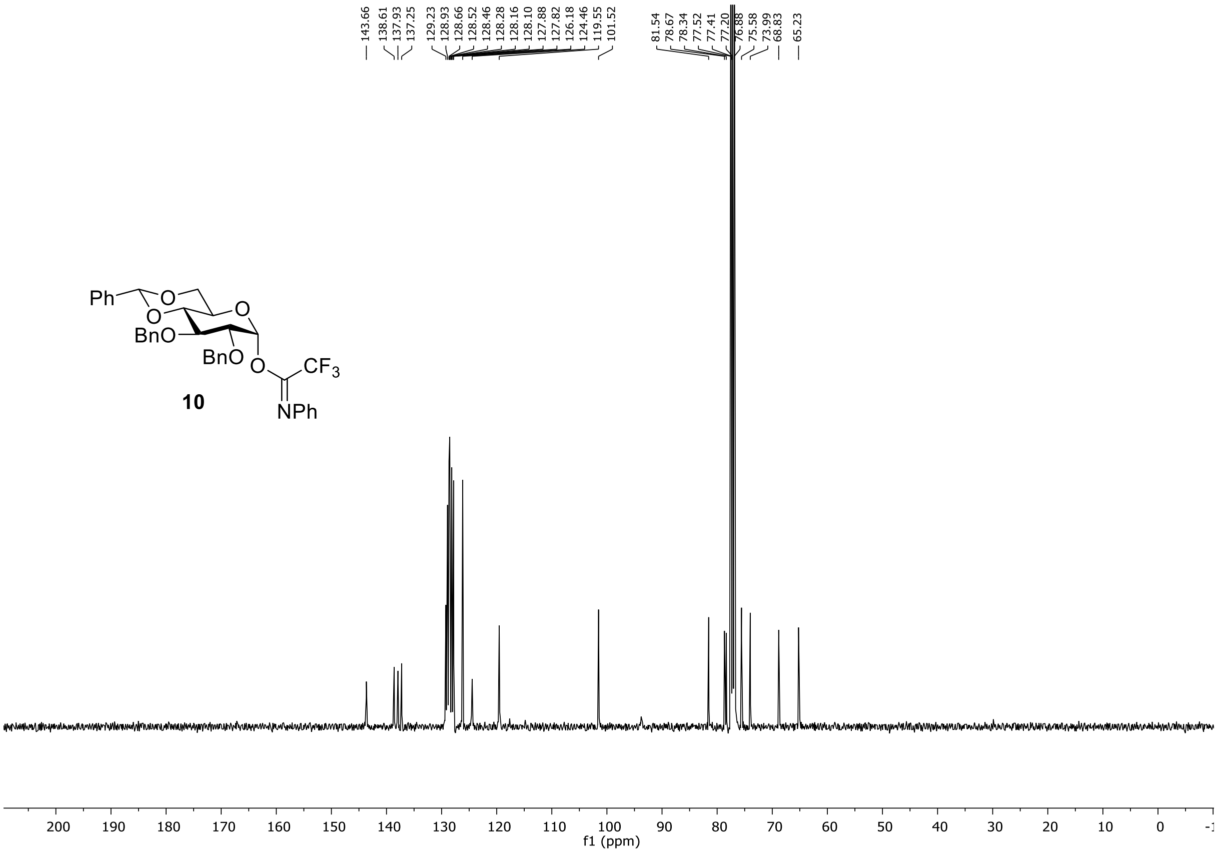

1H NMR, 400 MHz, $CDCl_3$

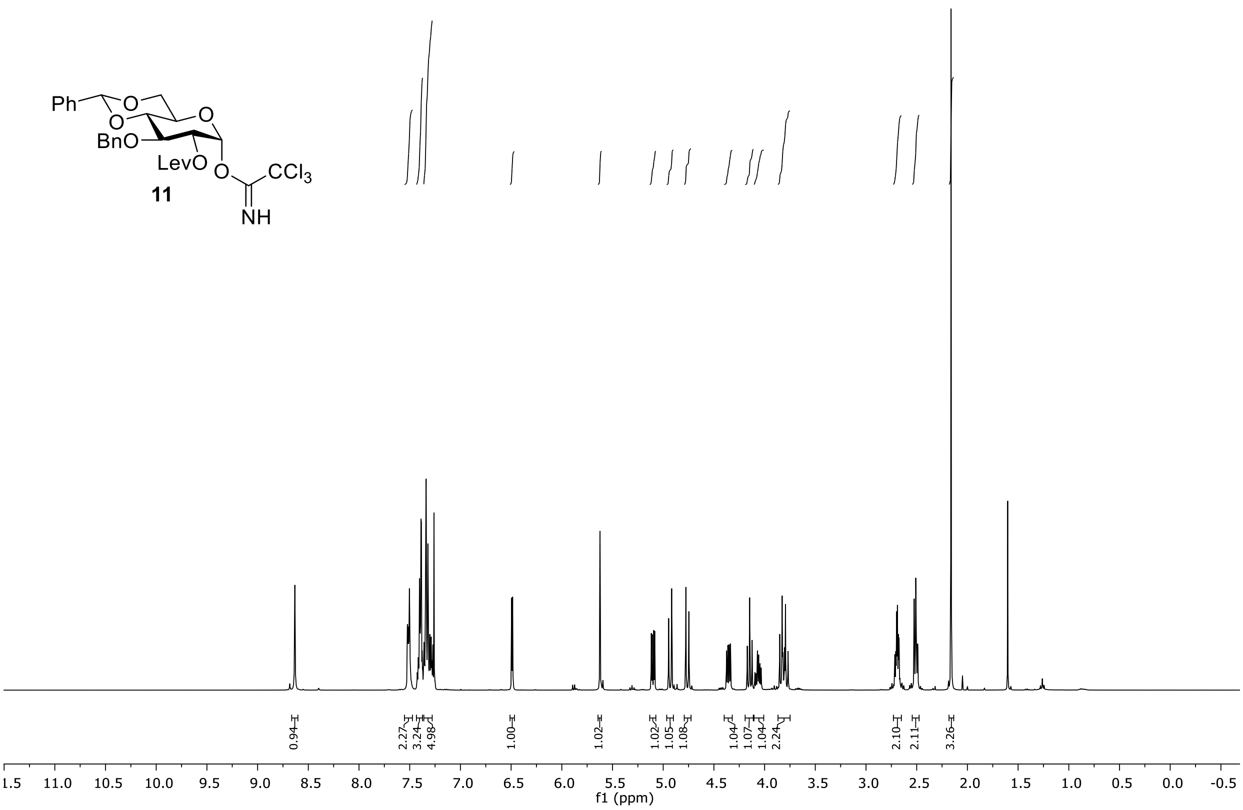

13C NMR, 101 MHz, $CDCl_3$

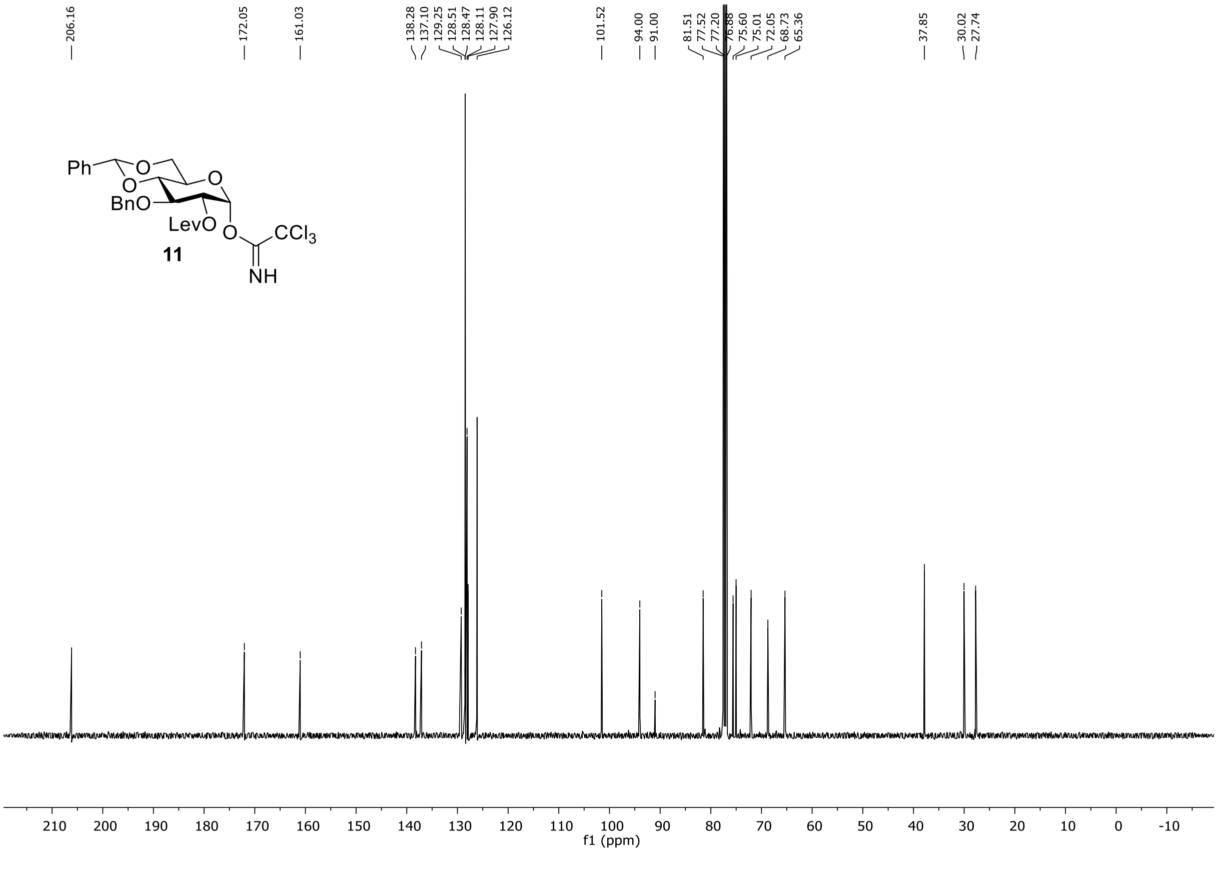

HH-COSY NMR, 400 MHz, $CDCl_3$

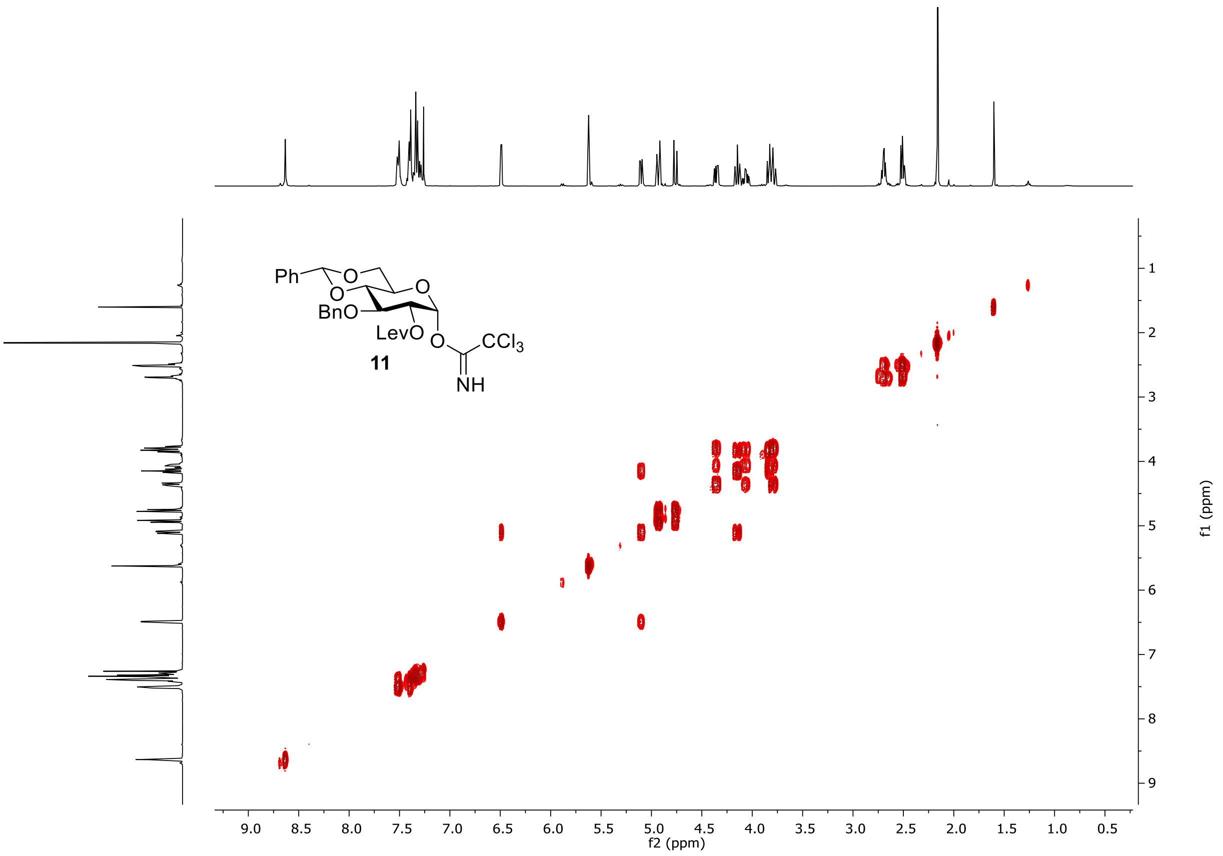

1H NMR, 400 MHz, $CDCl_3$

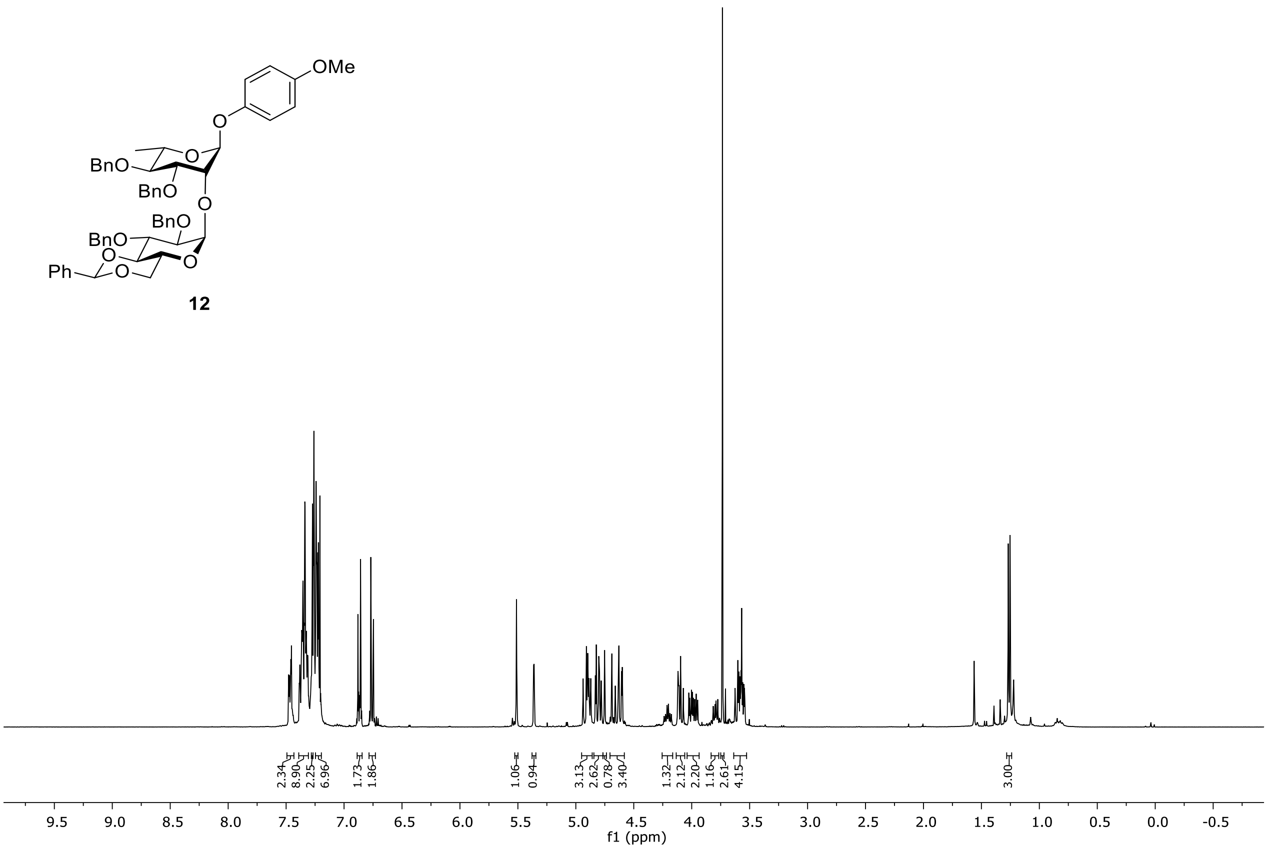

13C NMR, 101 MHz, CDCl3

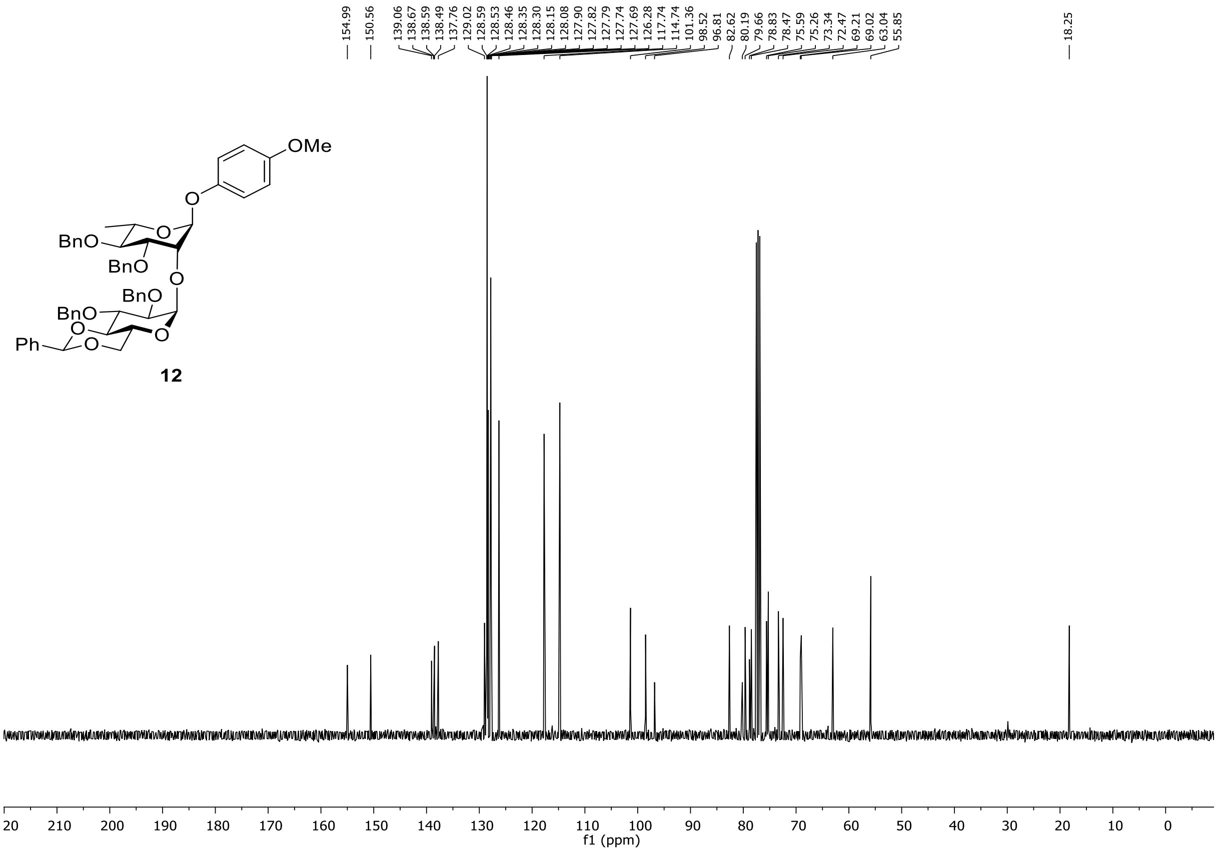

HH-COSY NMR, 400 MHz, CDCl3

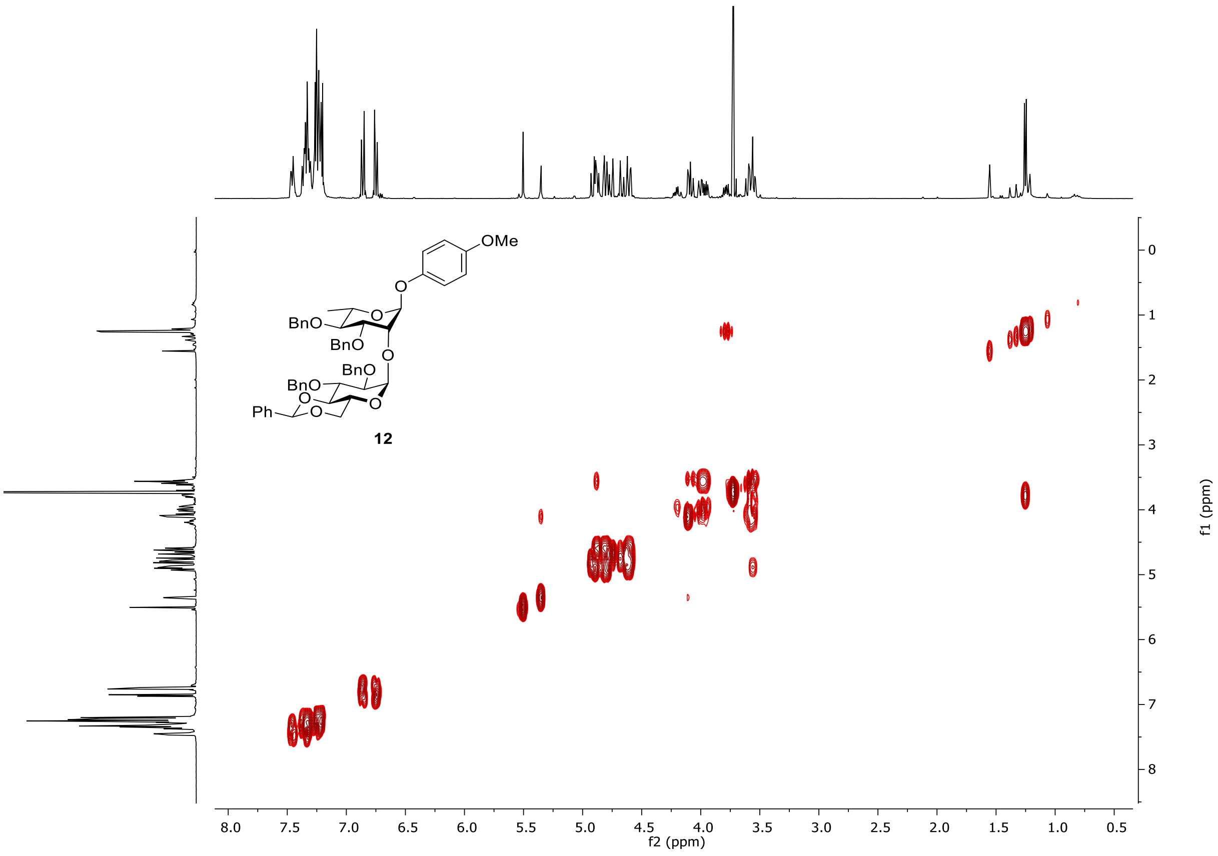

CH-HSQC NMR, 400 MHz, $CDCl_3$

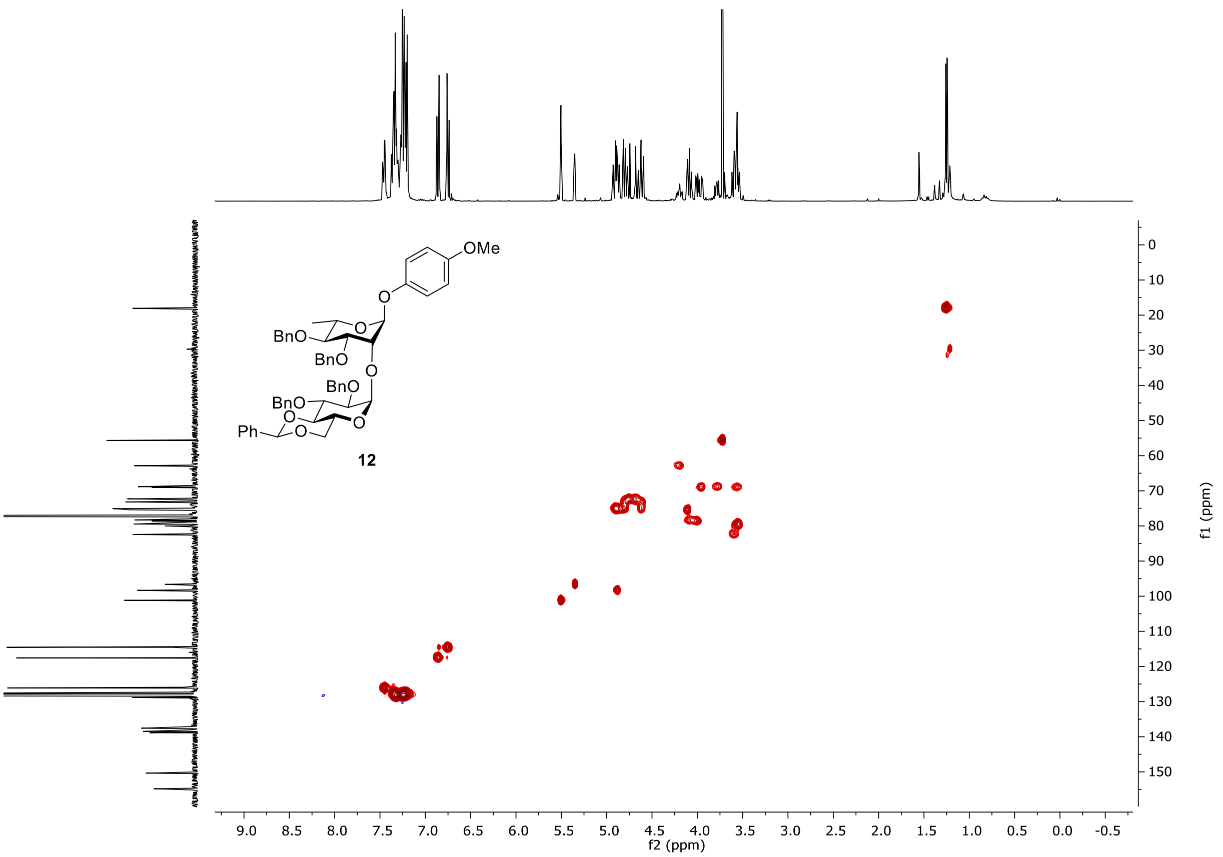

CH-HSQC NMR, 400 MHz, $CDCl_3$

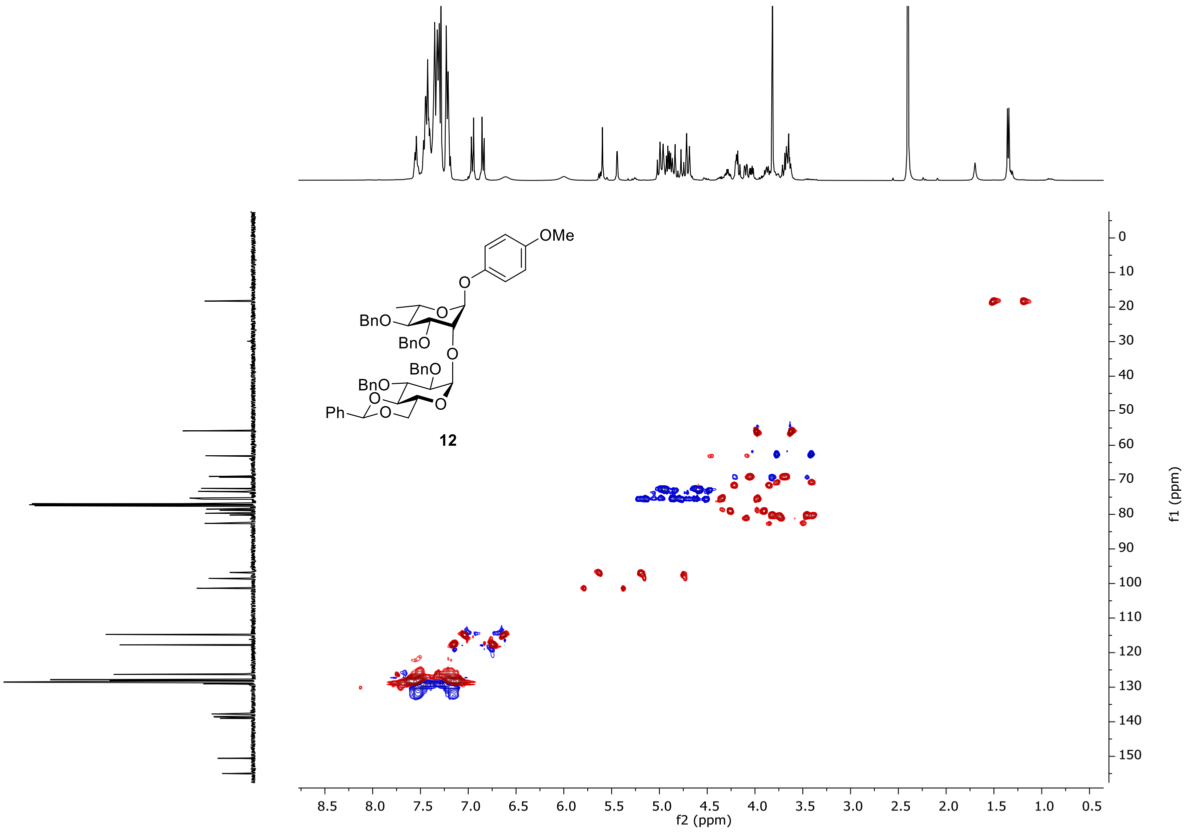

CH-HSQC NMR, 400 MHz, CDCl₃ (expansion)

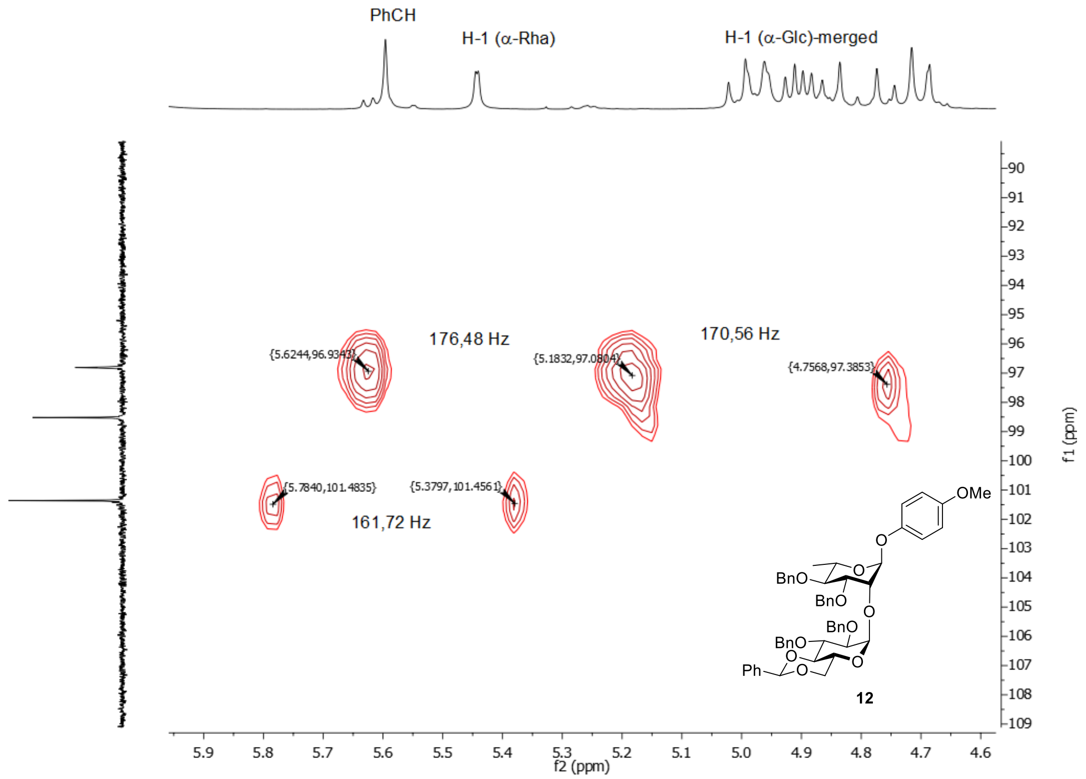

1H NMR, 400 MHz, CDCl₃

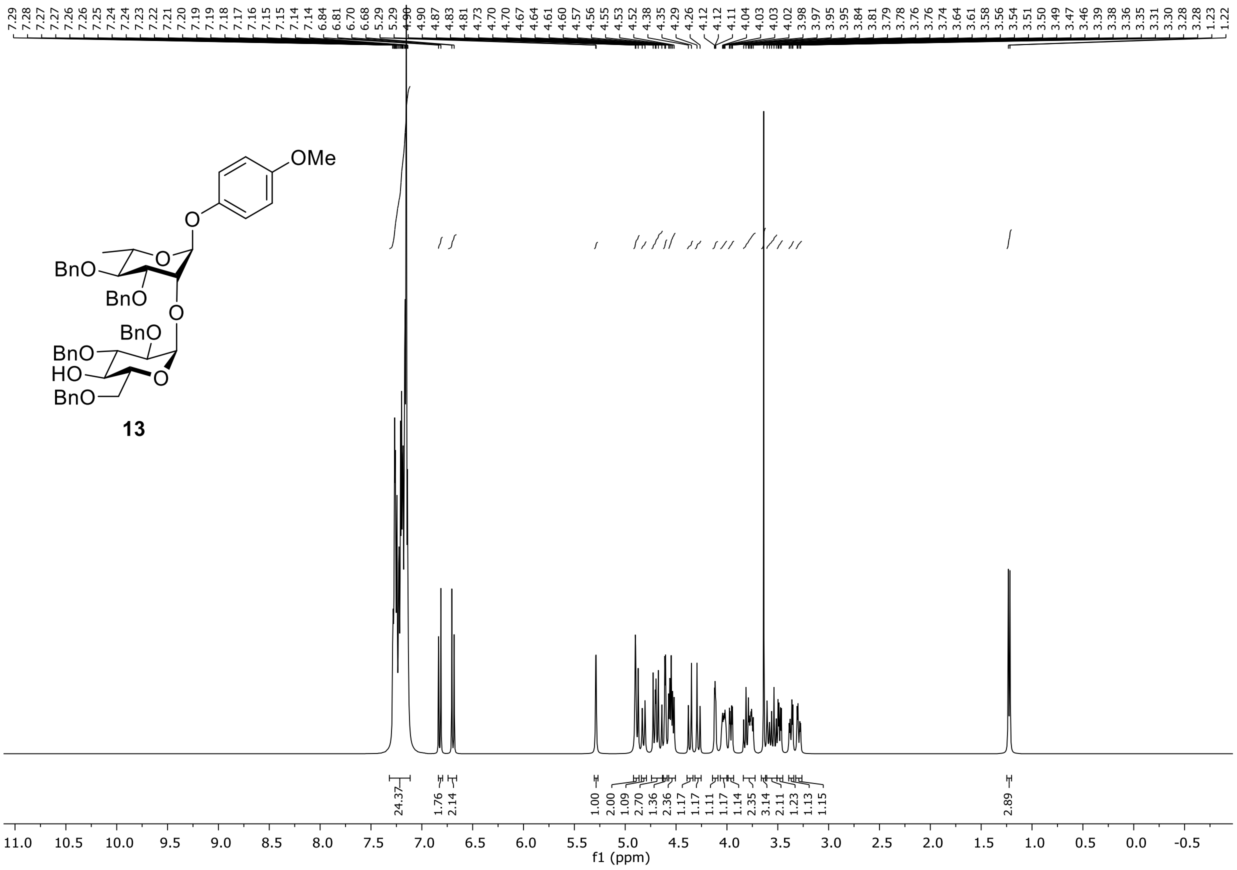

13C NMR, 101 MHz, $CDCl_3$

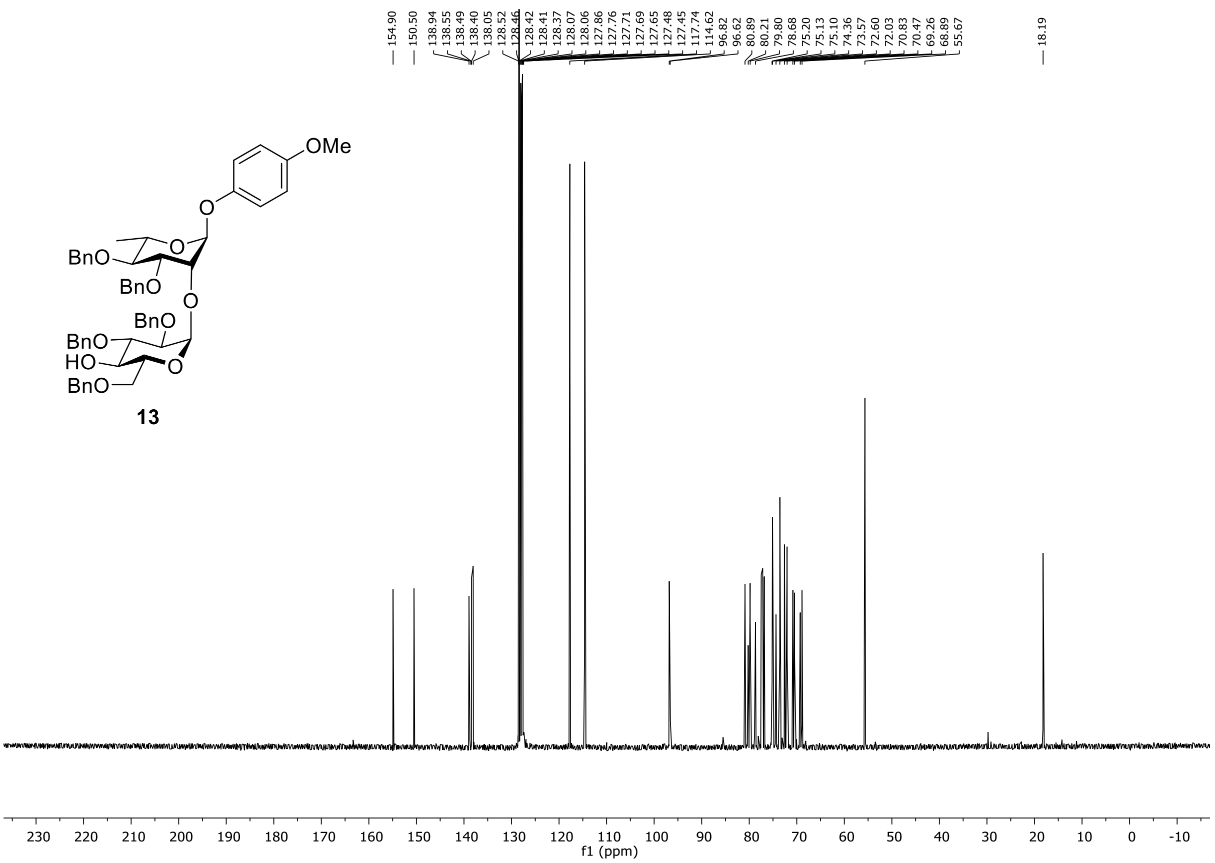

CH-HSQC NMR, 400 MHz, $CDCl_3$

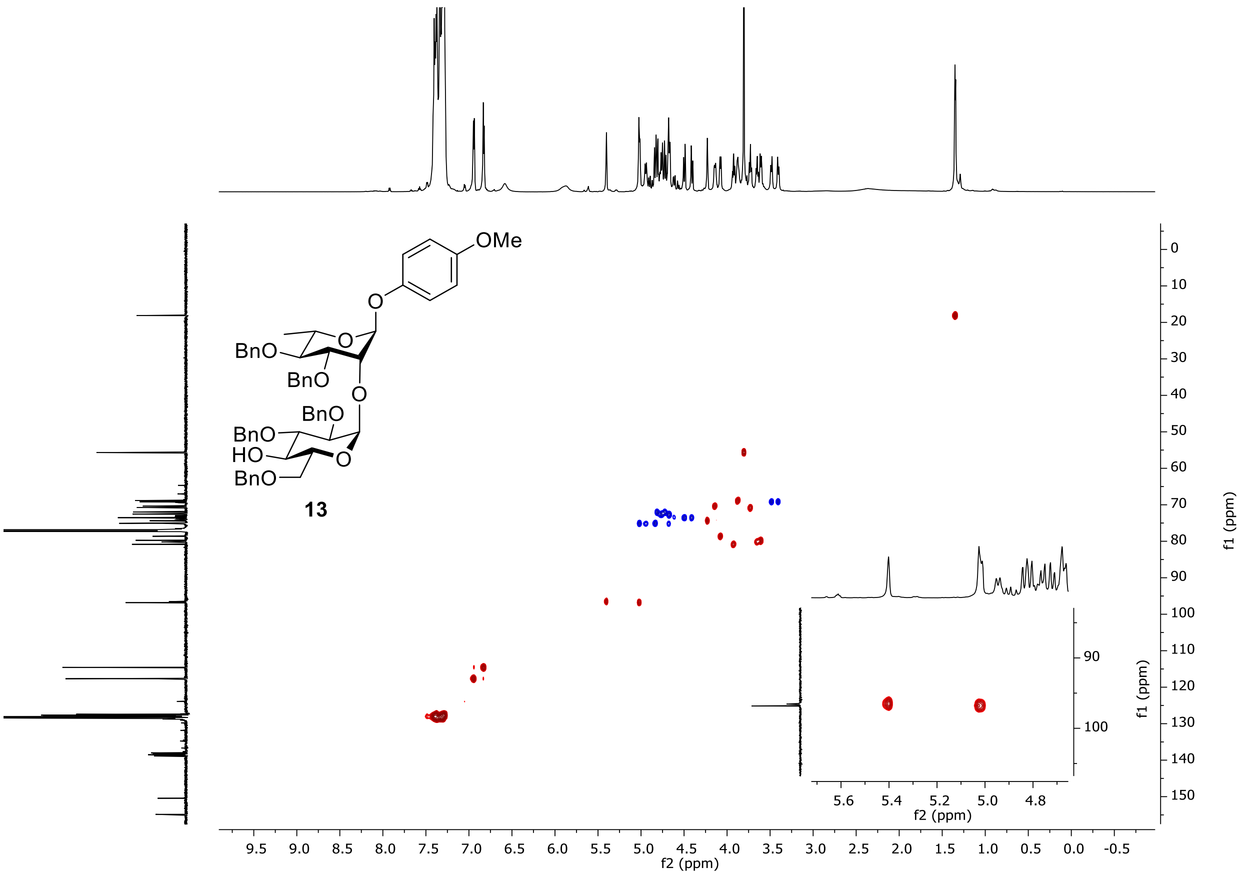

CH-HSQC NMR, 400 MHz, $CDCl_3$

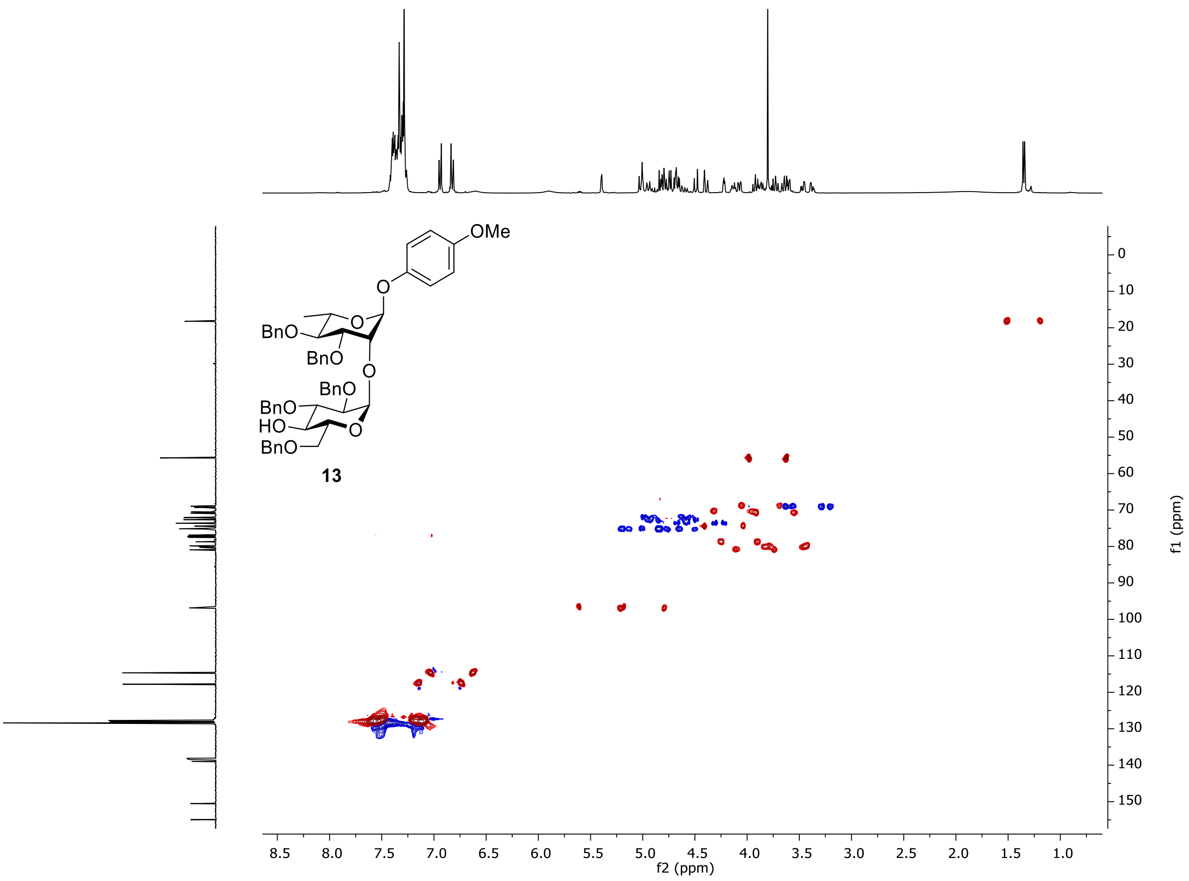

CH-HSQC NMR, 400 MHz, $CDCl_3$ (expansion)

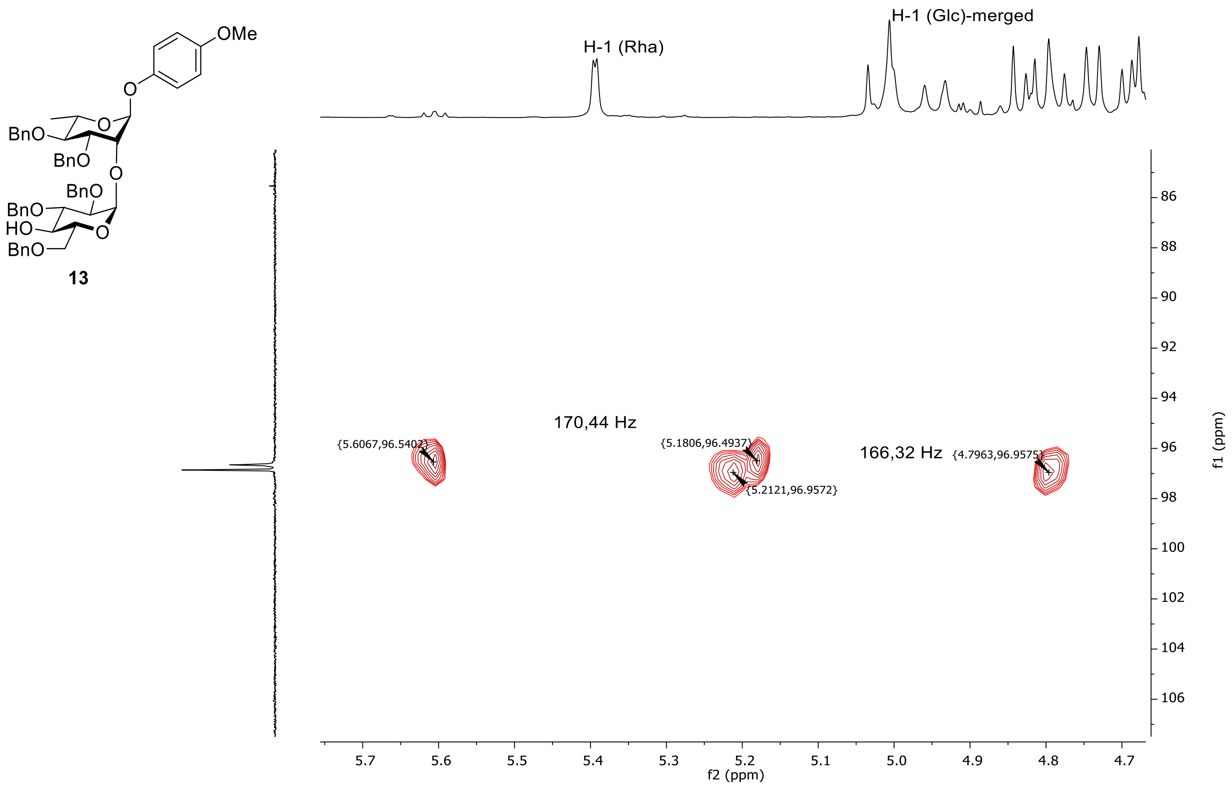

1H NMR, 400 MHz, CDCl3

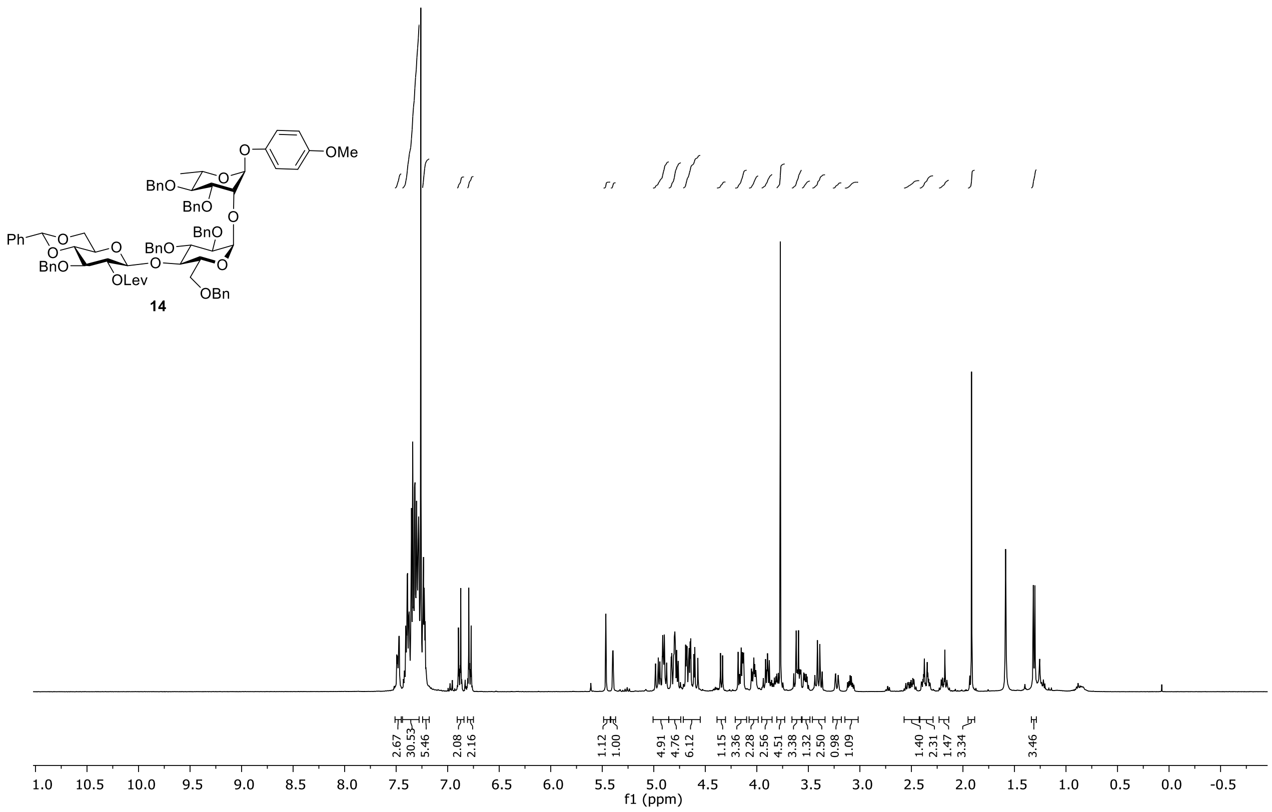

13C NMR, 101 MHz, CDCl3

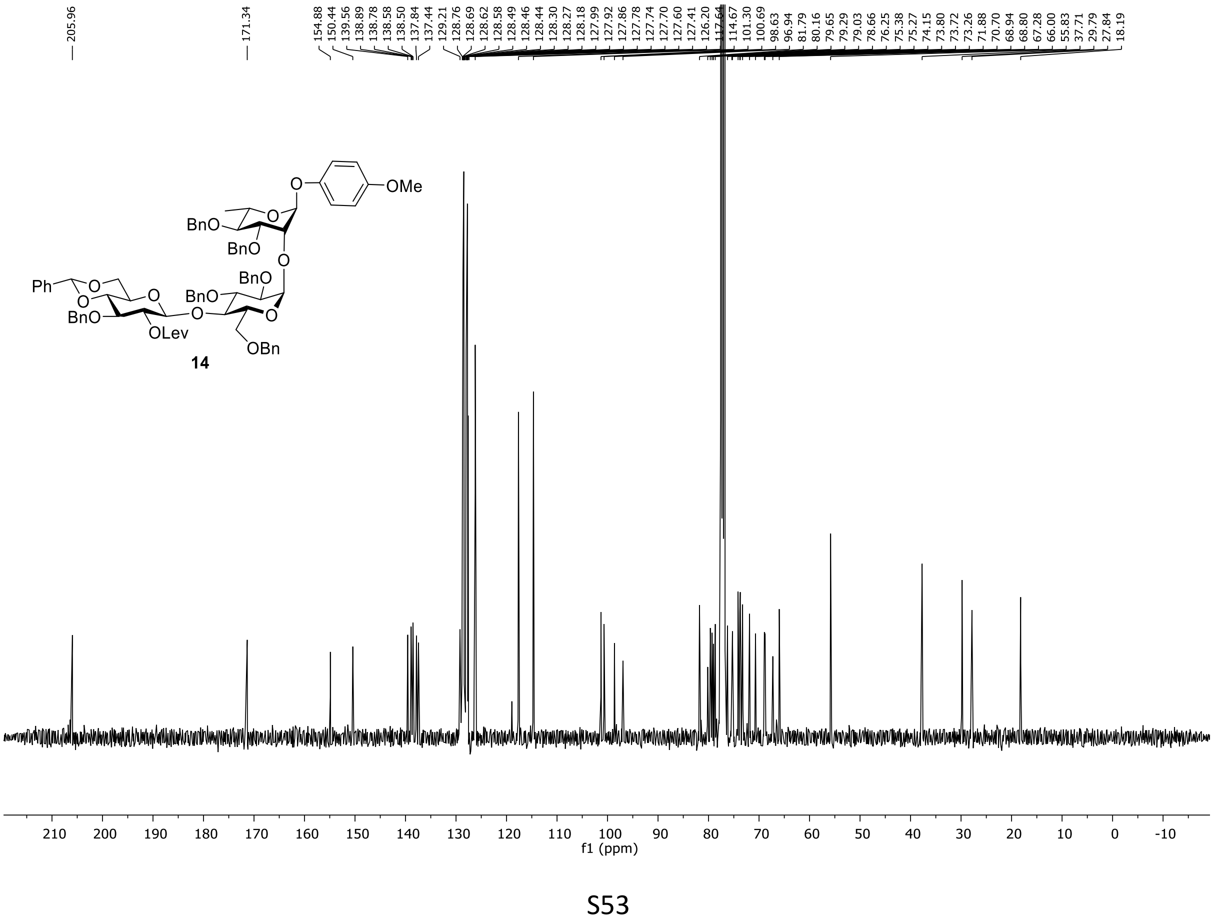

CH-HSQC NMR, 400 MHz, $CDCl_3$

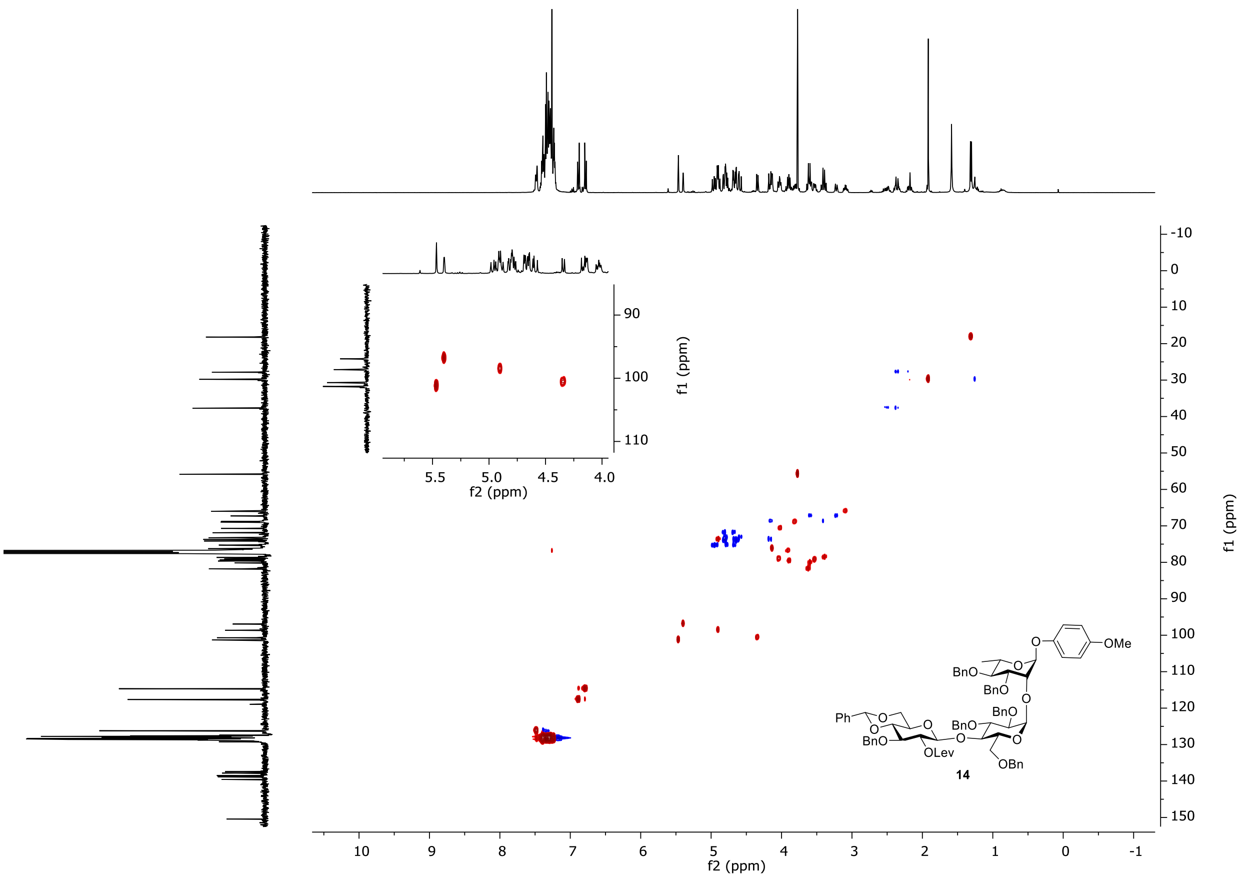

CH-HSQC NMR, 400 MHz, $CDCl_3$

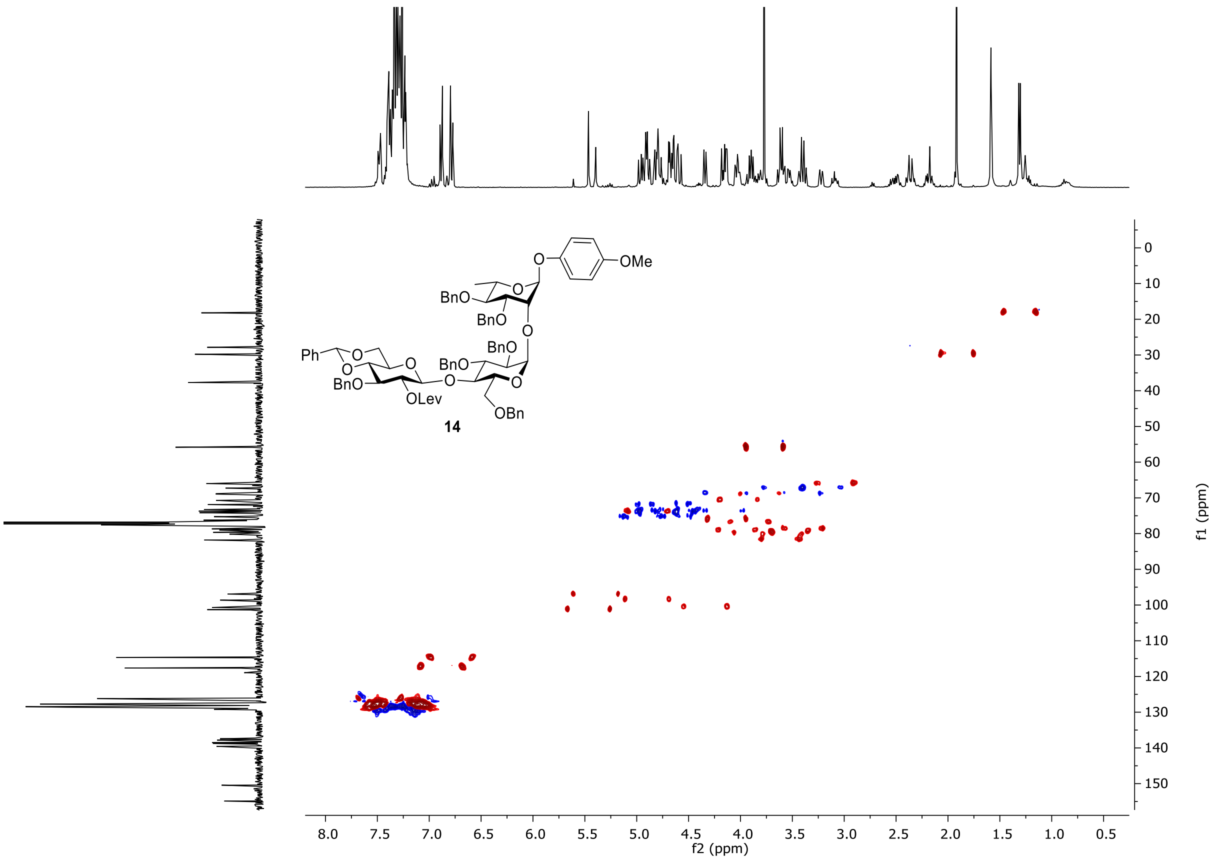

CH-HSQC NMR, 400 MHz, $CDCl_3$ (Expansion)

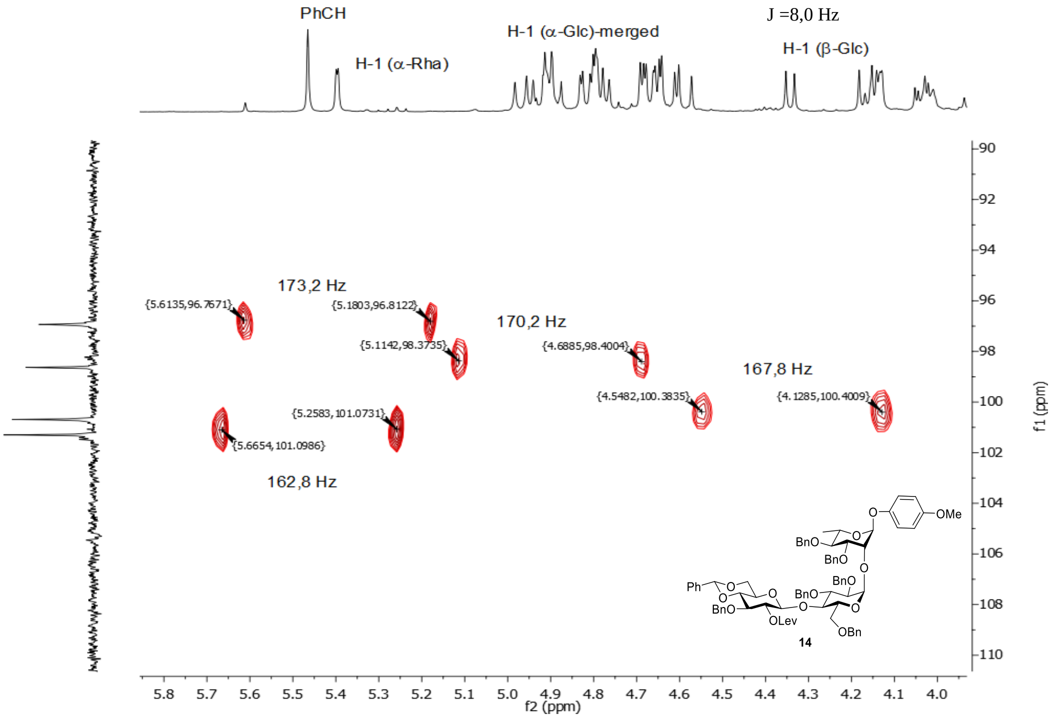

1H NMR, 400 MHz, $CDCl_3$

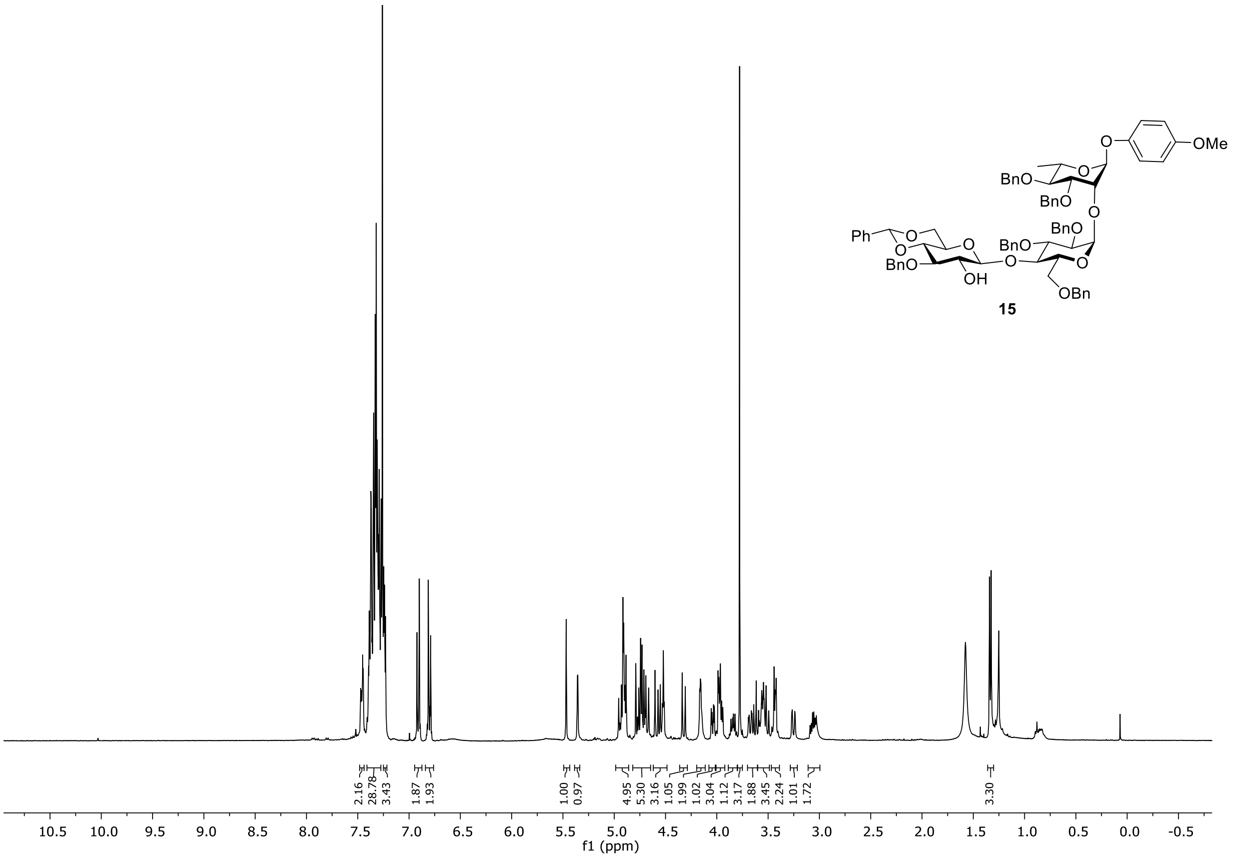

13C NMR, 101 MHz, $CDCl_3$

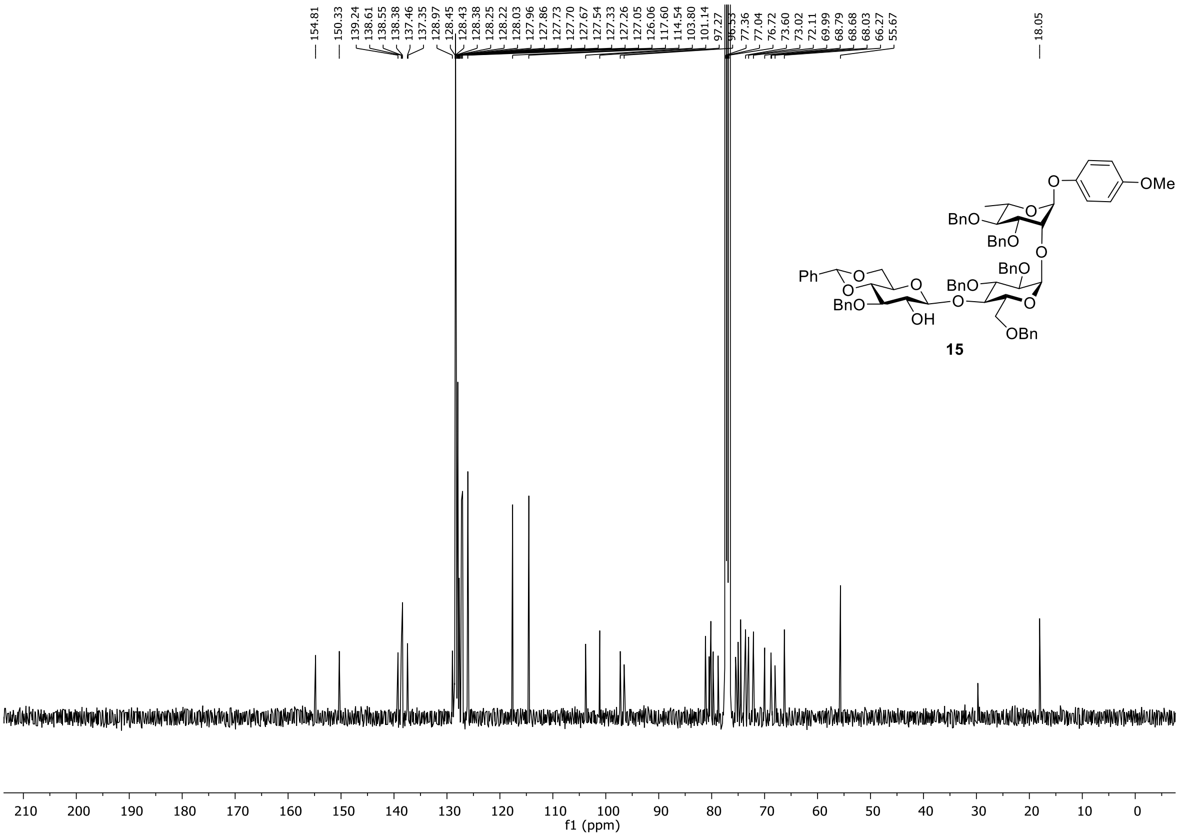

CH-HSQC NMR, 400 MHz, $CDCl_3$

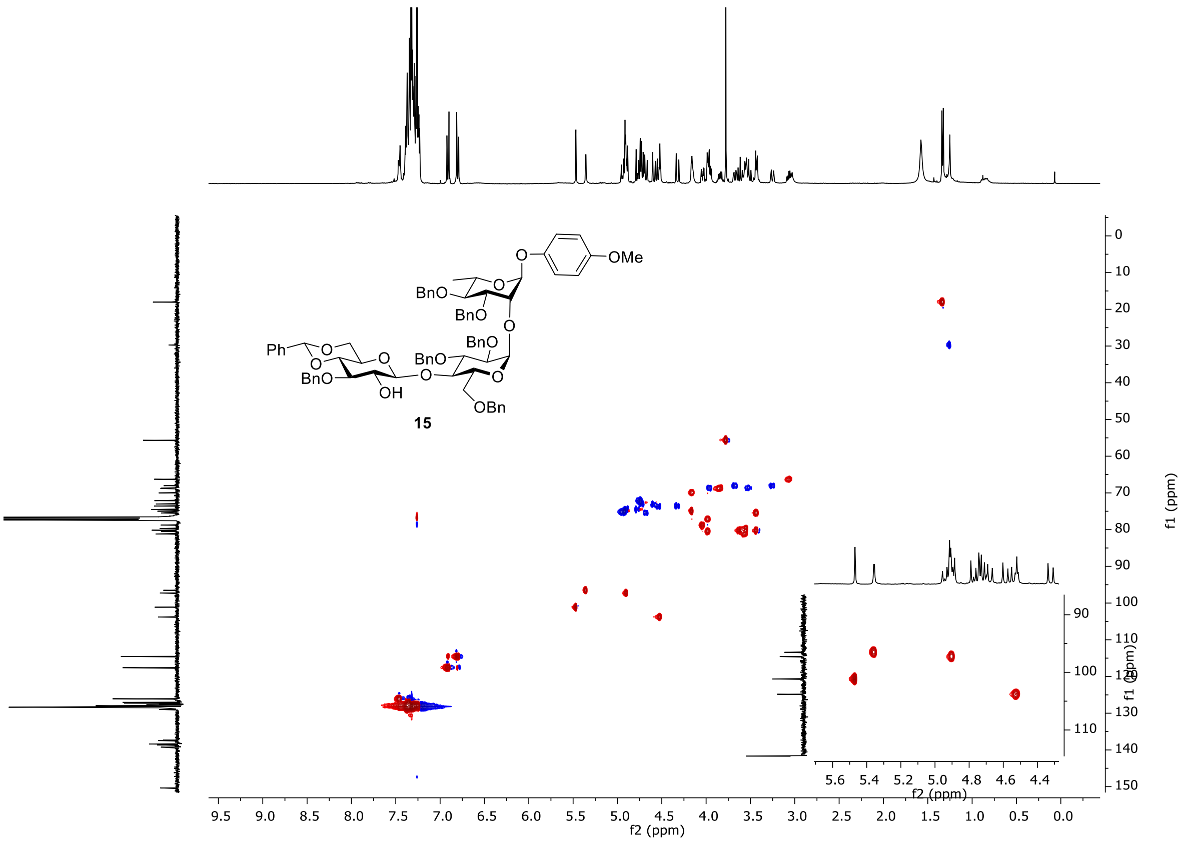

CH-HSQC NMR, 400 MHz, CDCl3

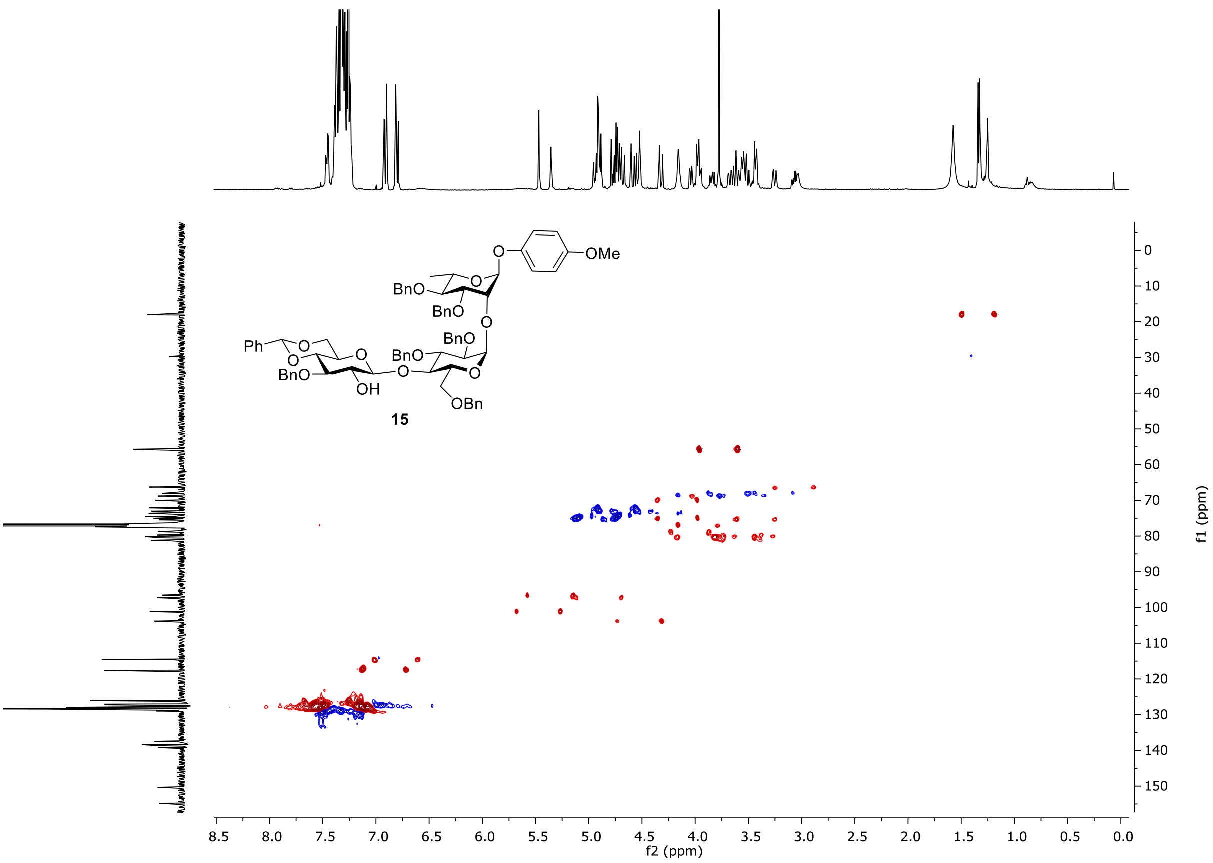

CH-HSQC NMR, 400 MHz, CDCl3 (expansion)

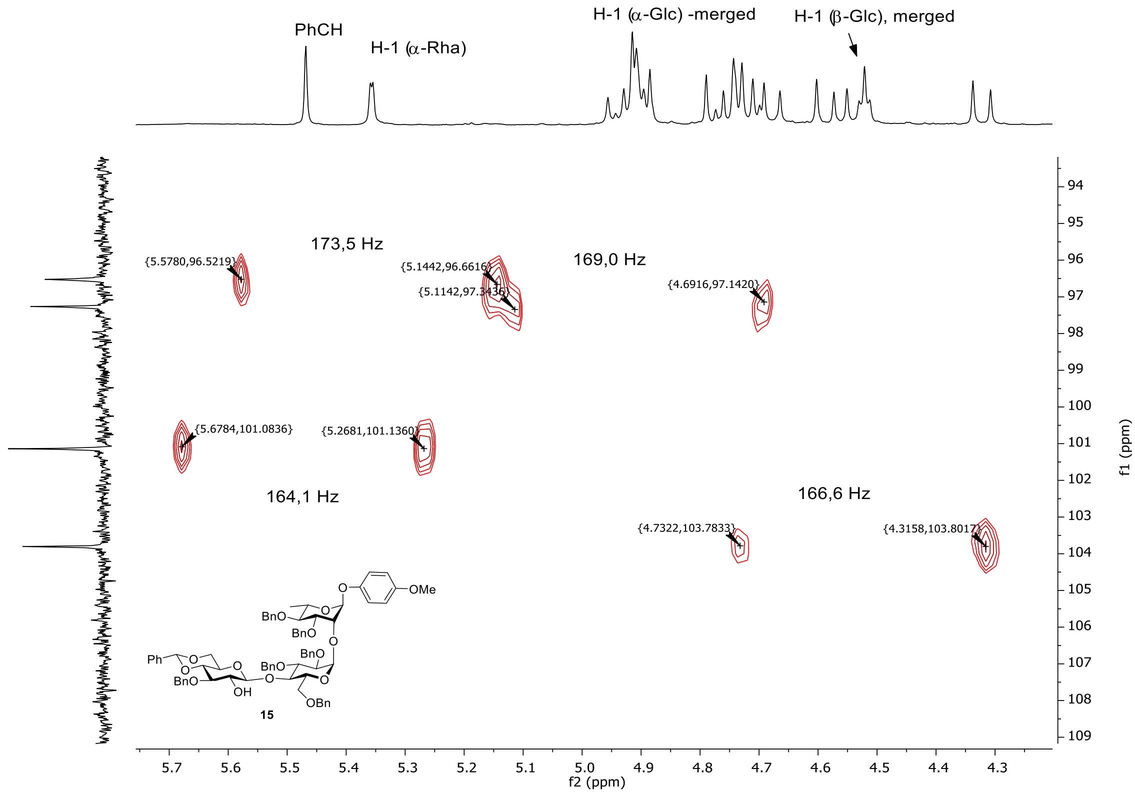

1H NMR, 400 MHz, $CDCl_3$

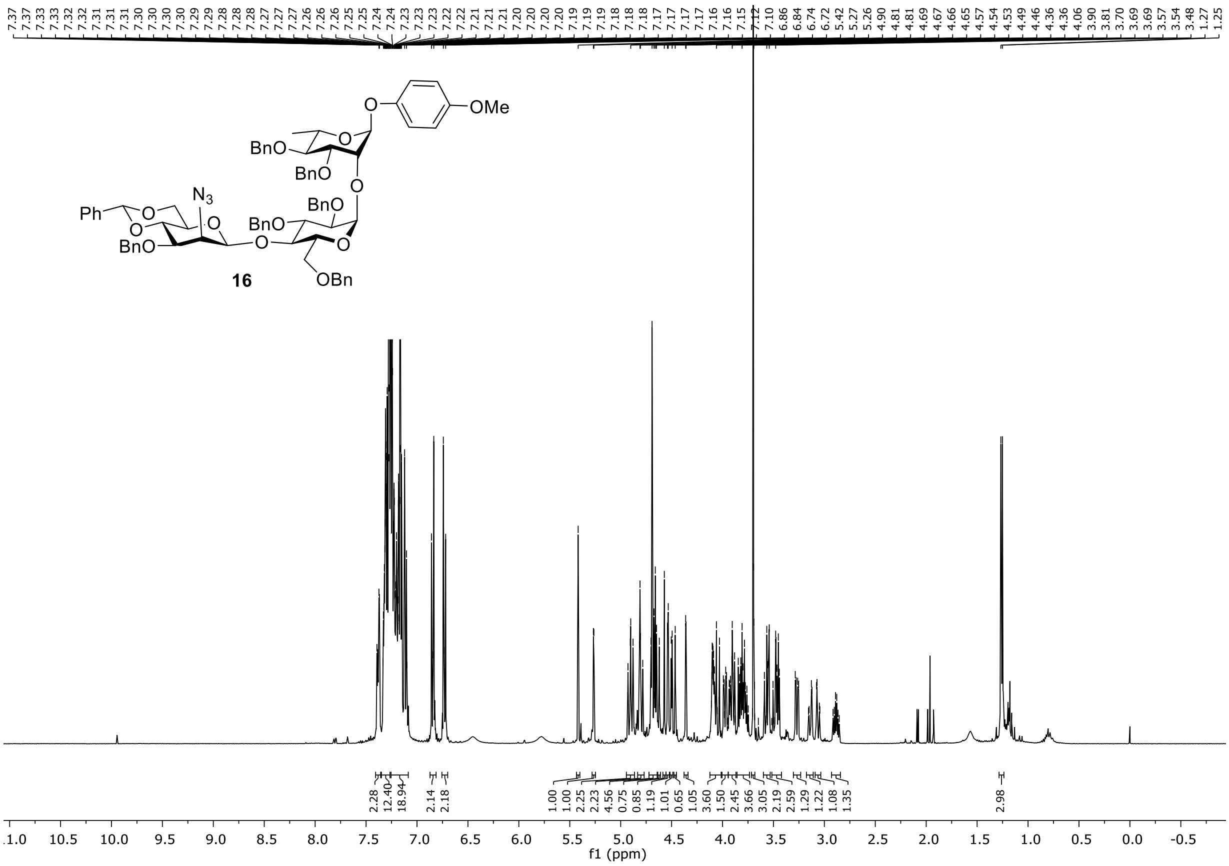

13C NMR, 101 MHz, $CDCl_3$

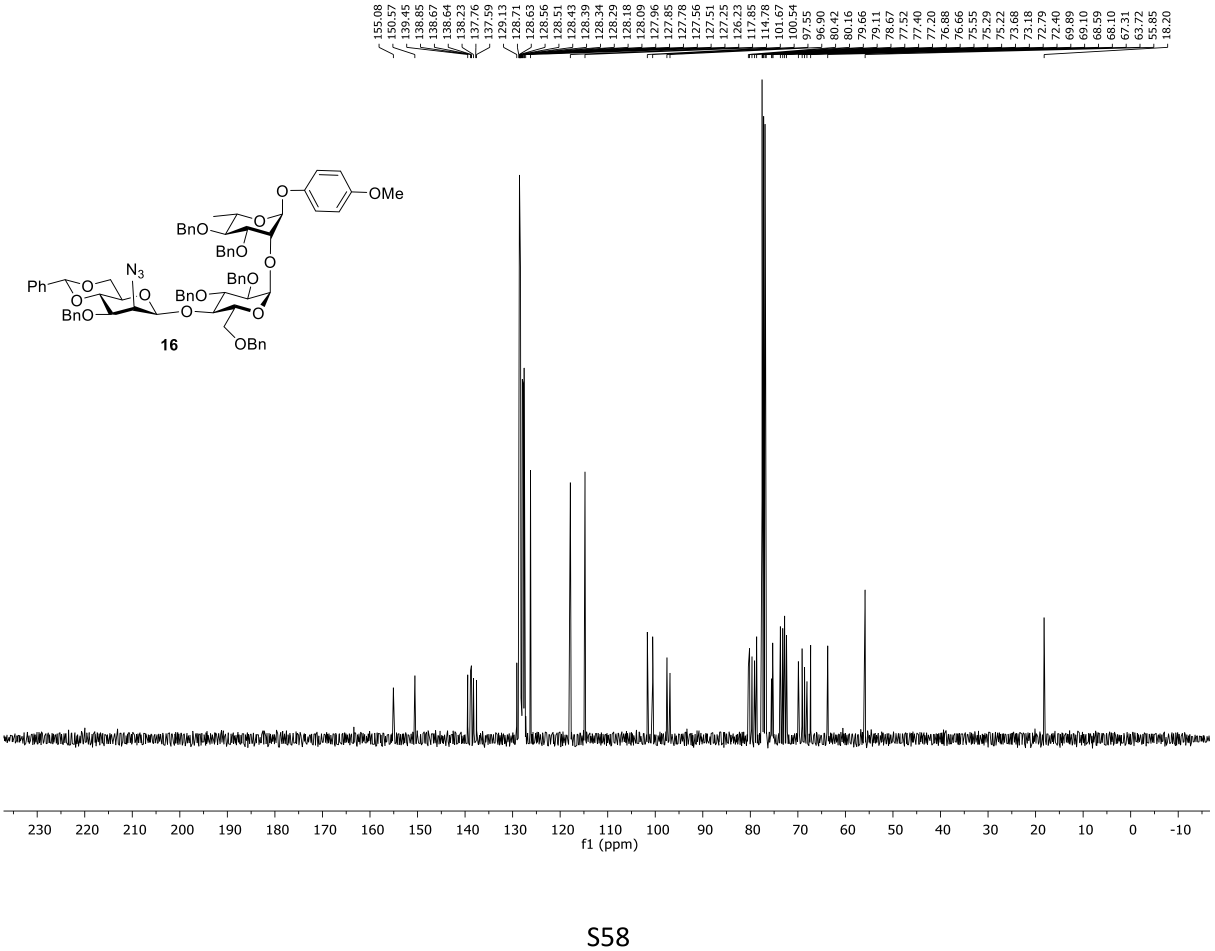

CH-HSQC NMR, 400 MHz, $CDCl_3$

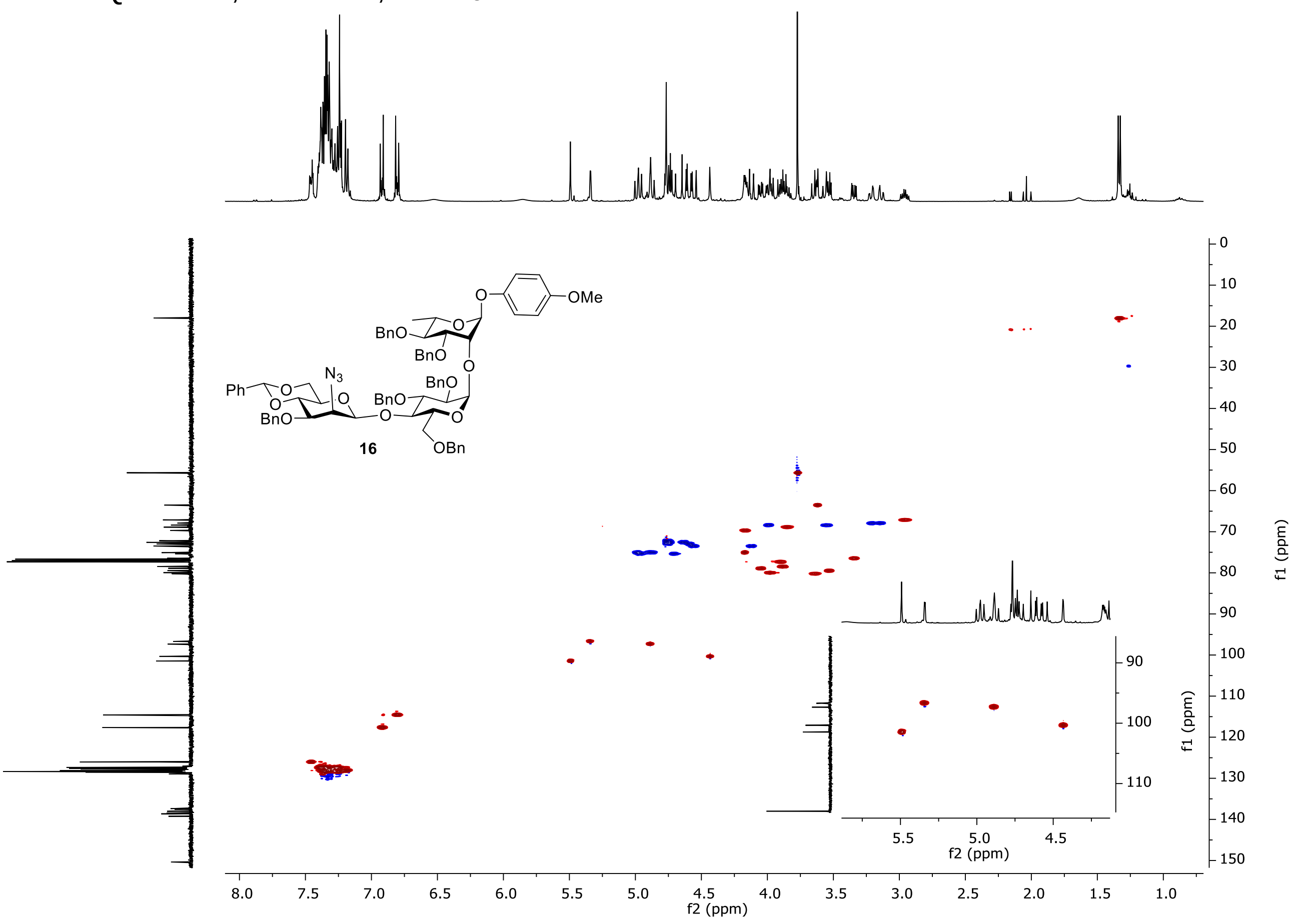

CH-HSQC NMR, 400 MHz, $CDCl_3$

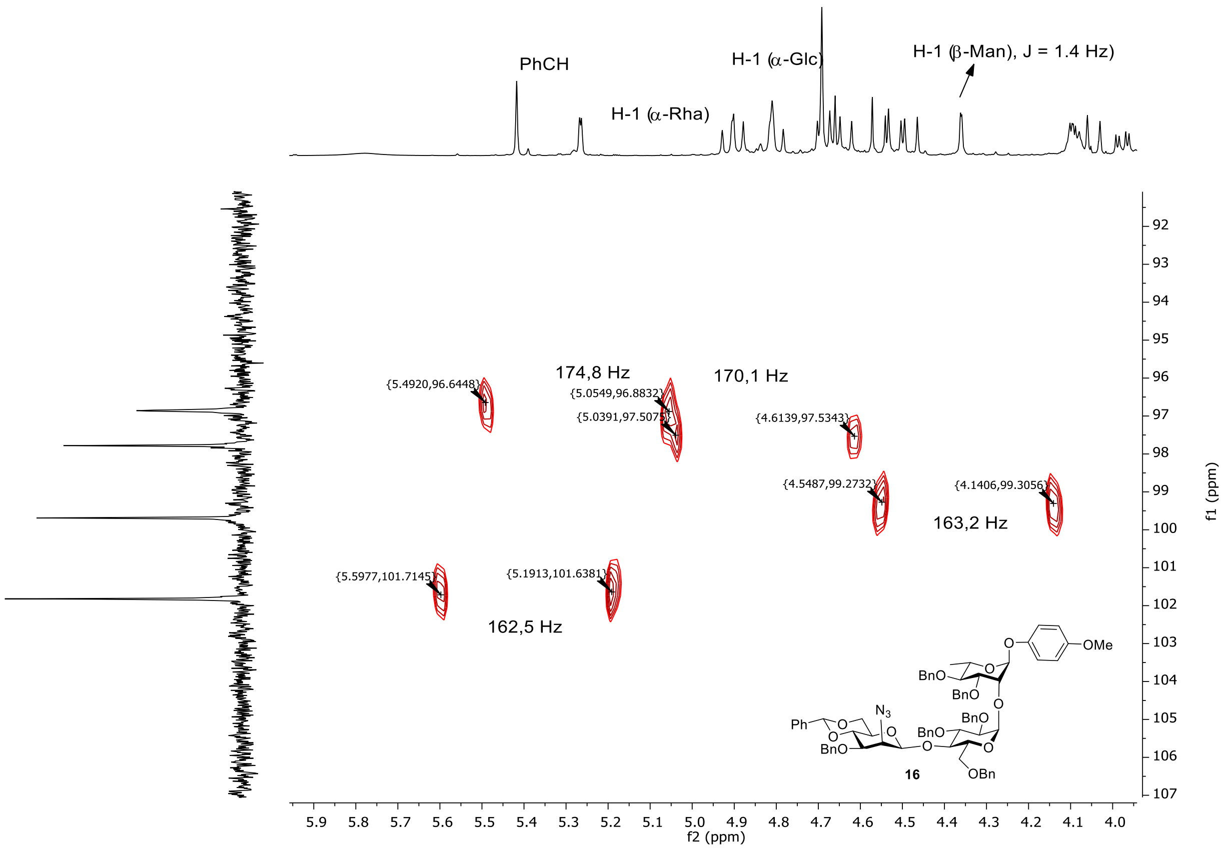

1H NMR, 400 MHz, CDCl₃

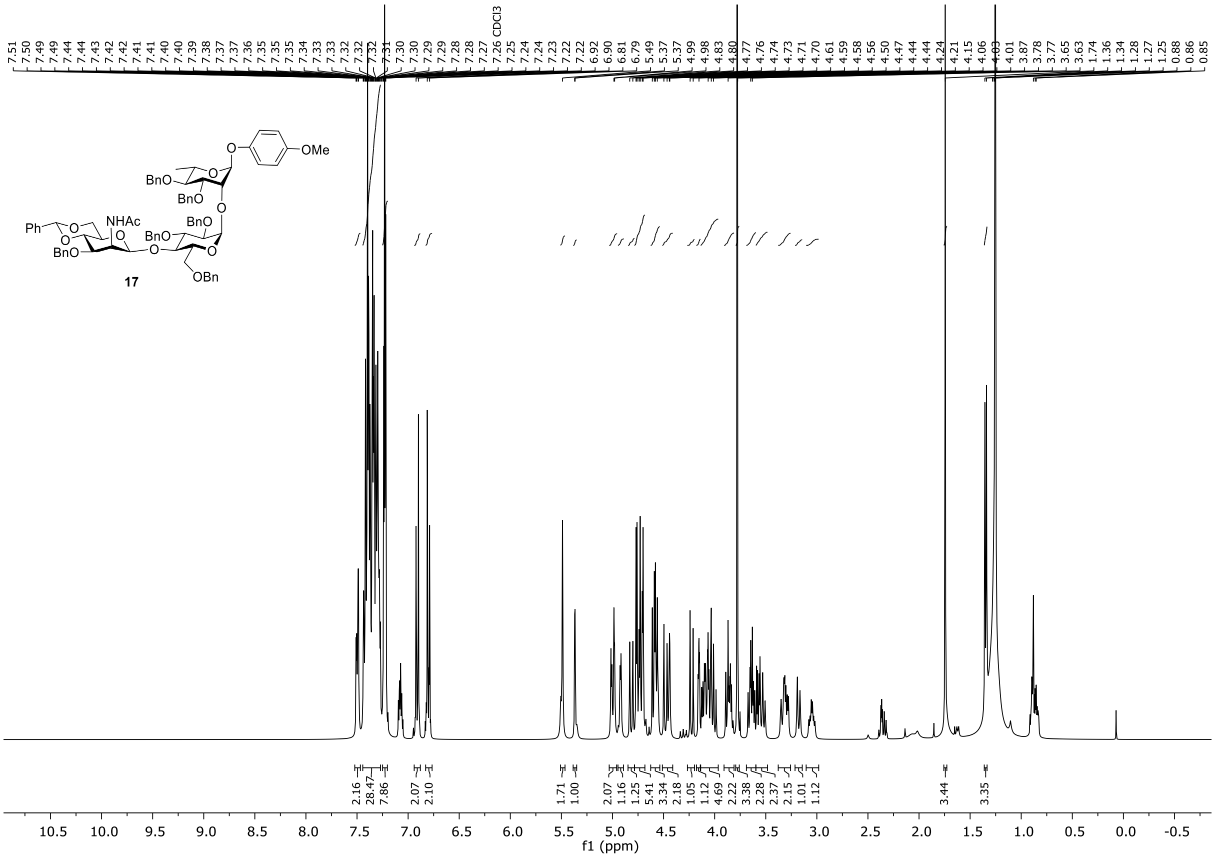

13C NMR, 101 MHz, CDCl₃

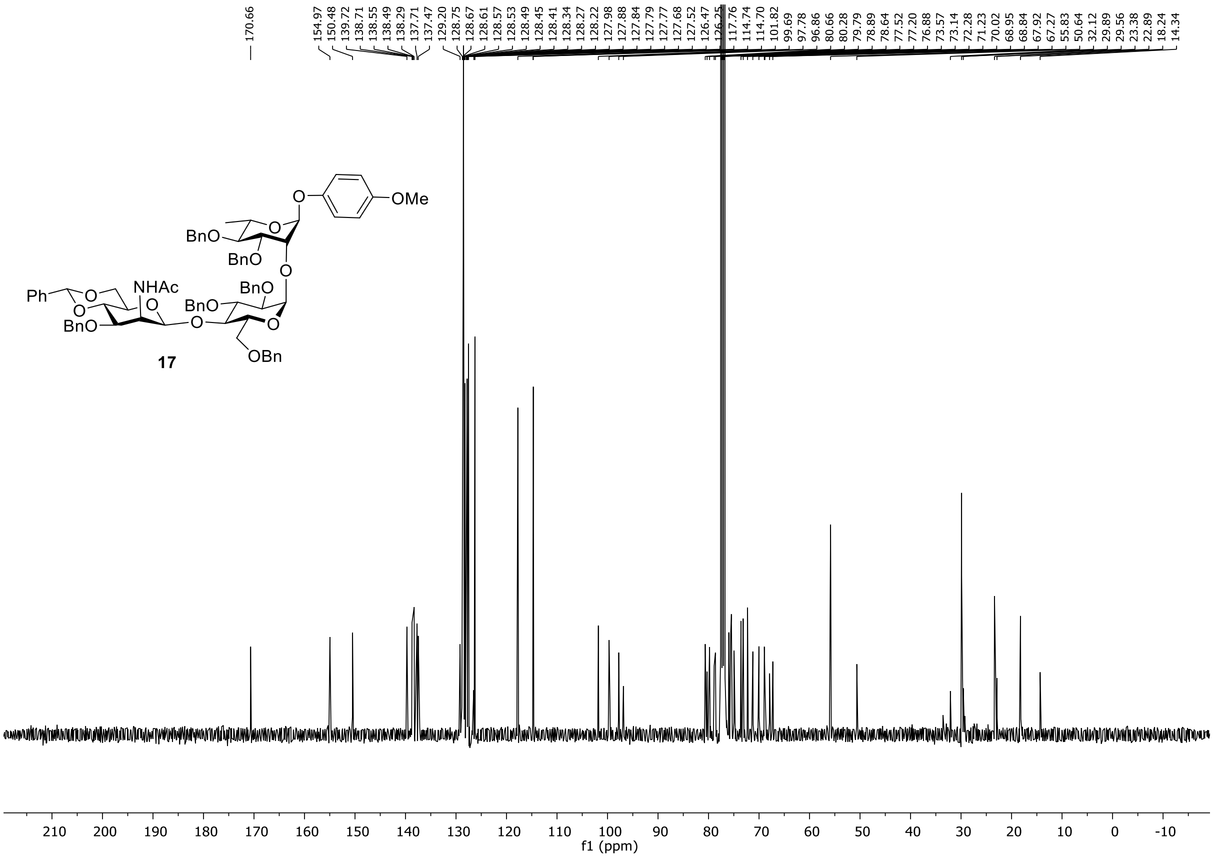

CH-HSQC NMR, 400 MHz, $CDCl_3$

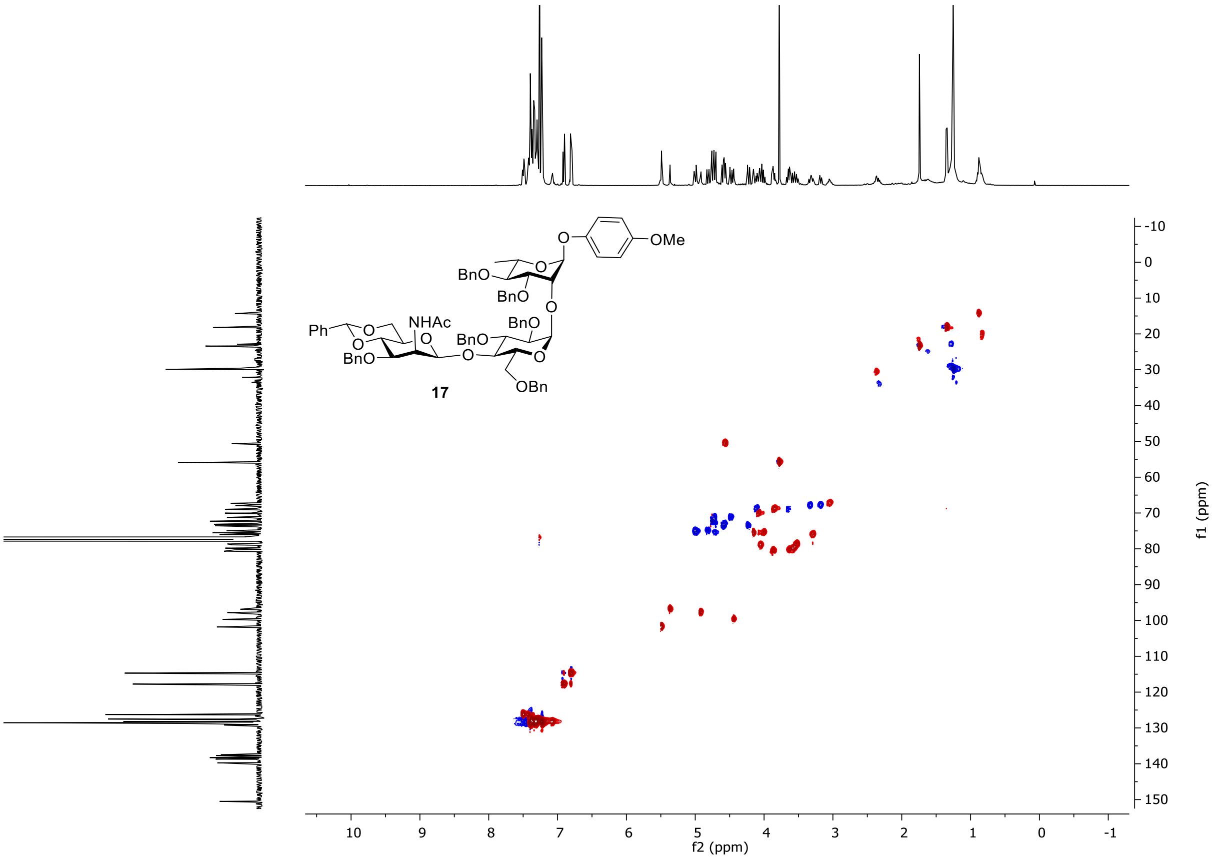

CH-HSQC NMR, 400 MHz, $CDCl_3$

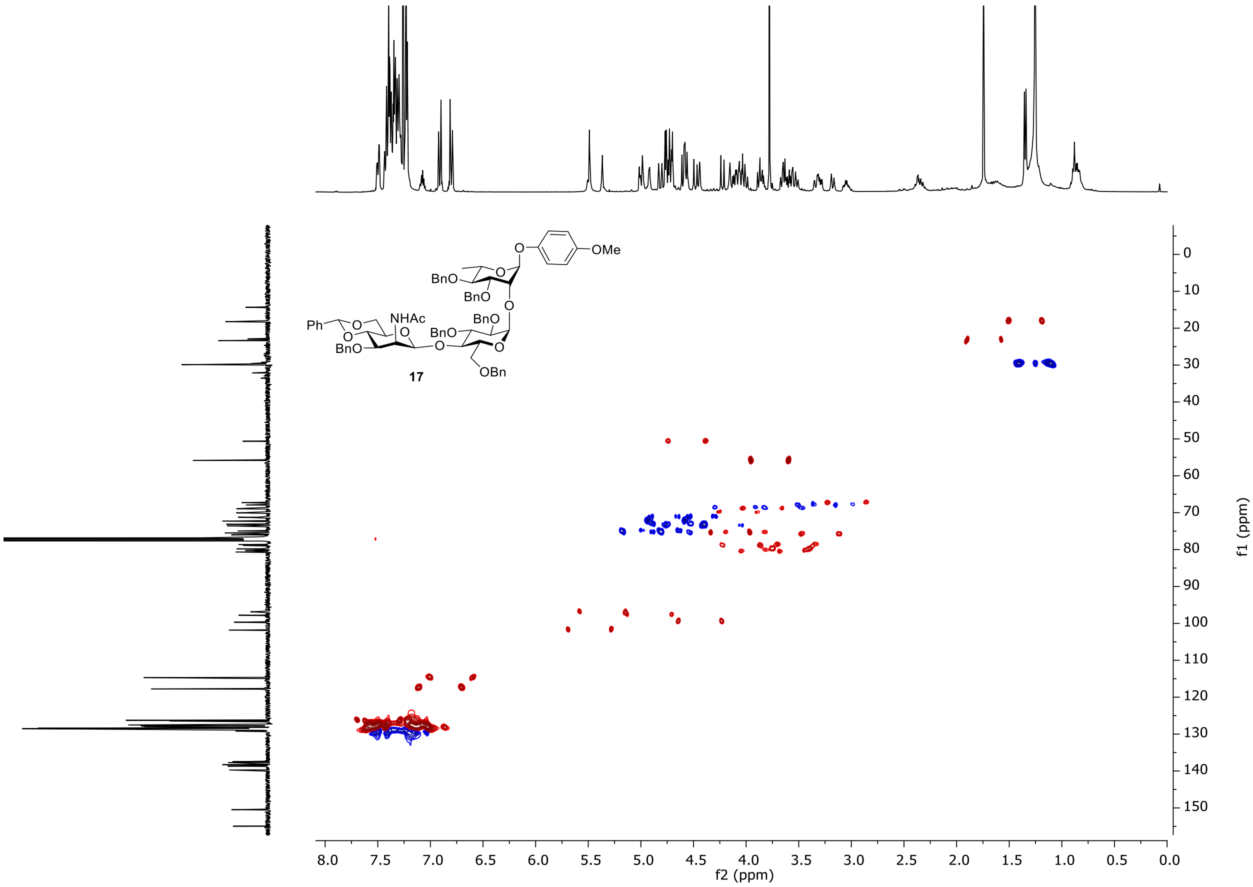

CH-HSQC NMR, 400 MHz, CDCl3 (expansion)

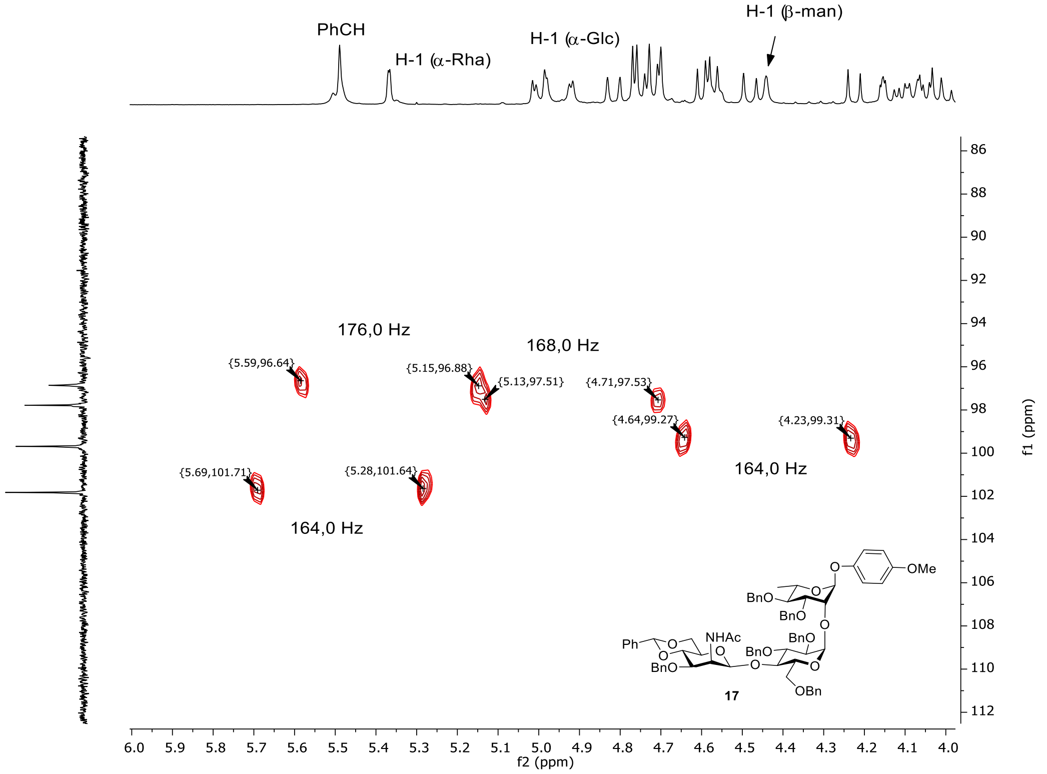

1H NMR, 400 MHz, CDCl3

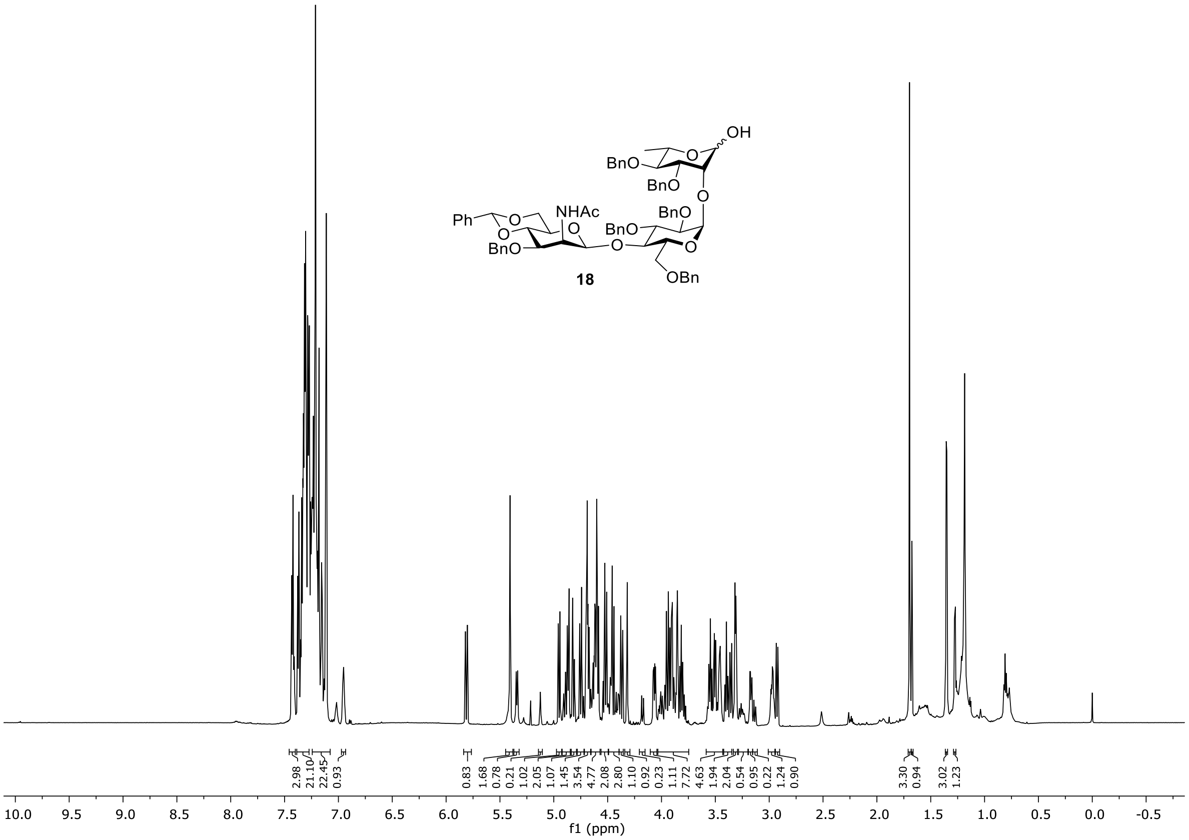

13C NMR, 101 MHz, CDCl3

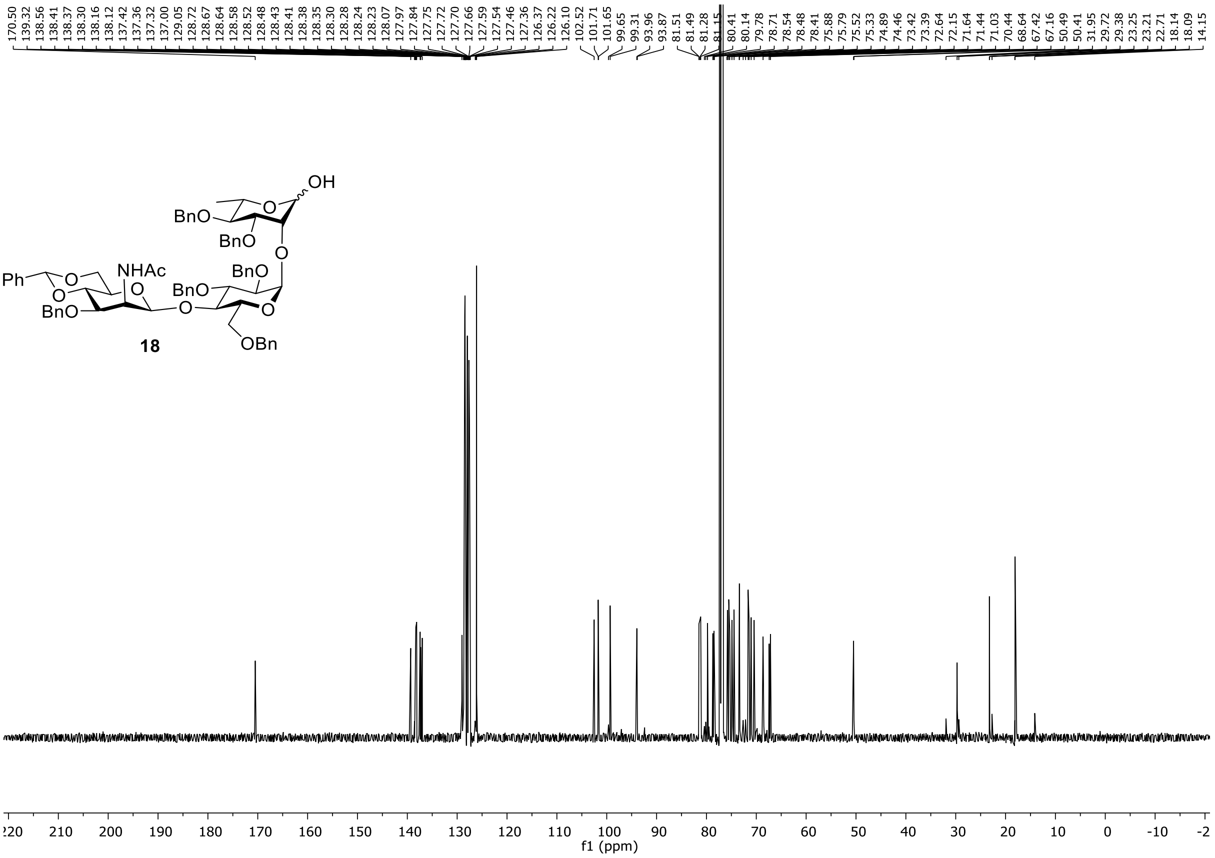

CH-HSQC NMR, 400 MHz, $CDCl_3$

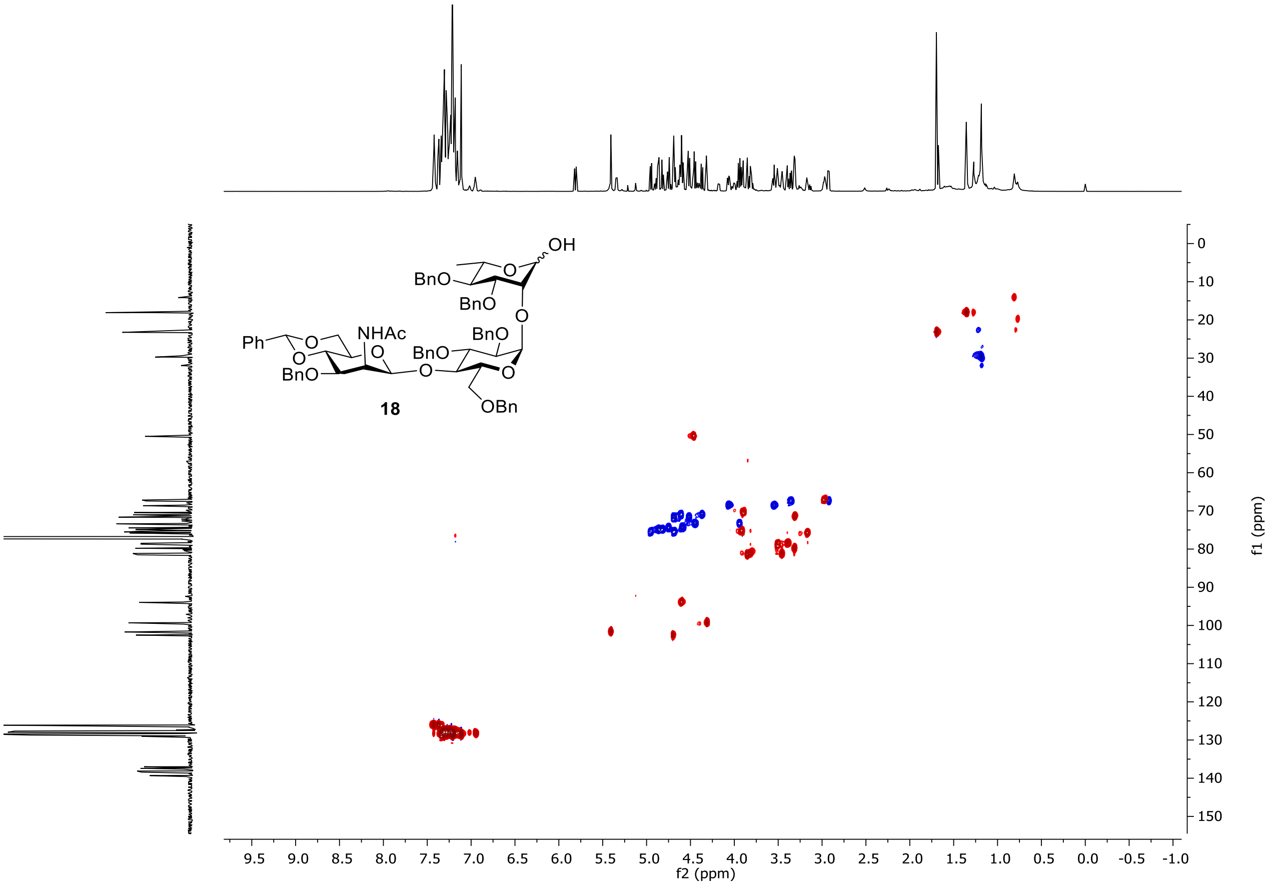

1H NMR, 700 MHz, CDCl₃

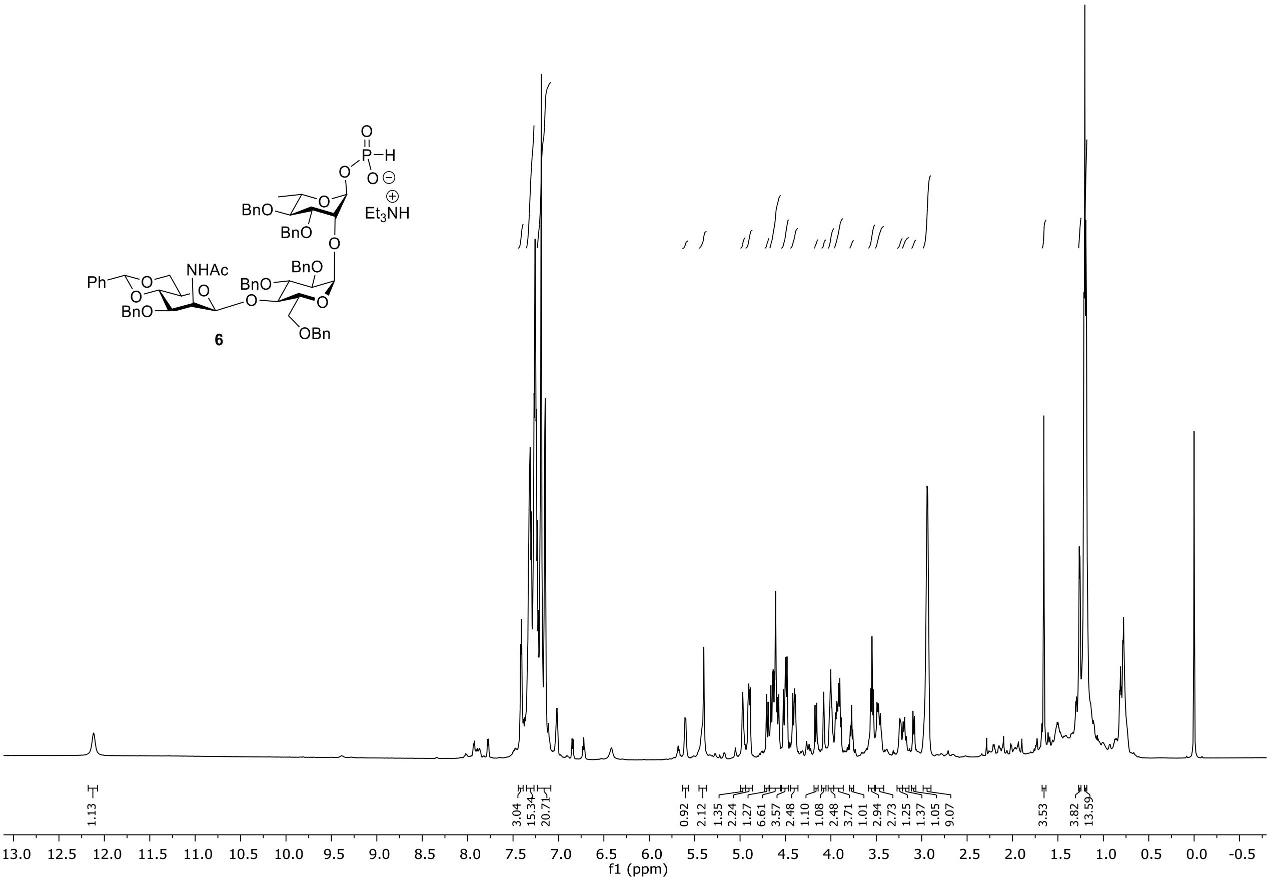

31P NMR, 162 MHz, CDCl₃

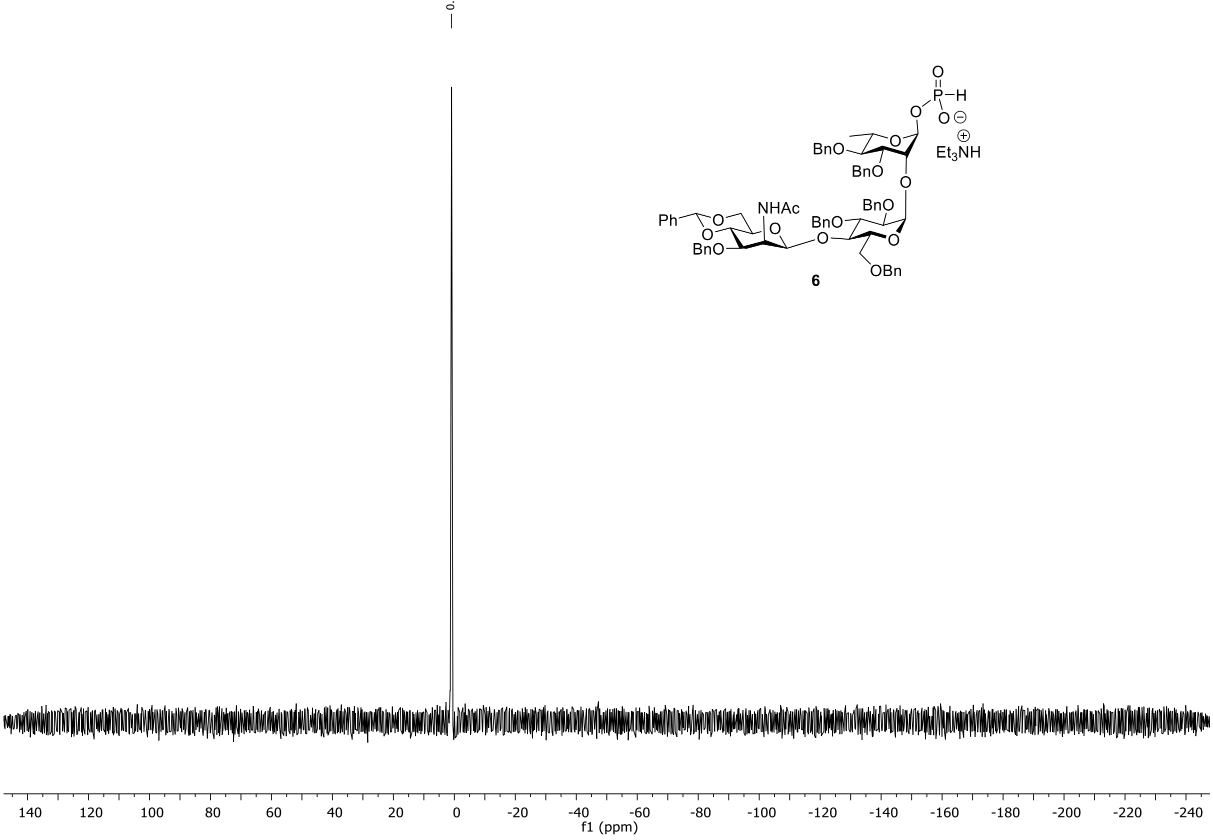

31P NMR, 162 MHz, $CDCl_3$

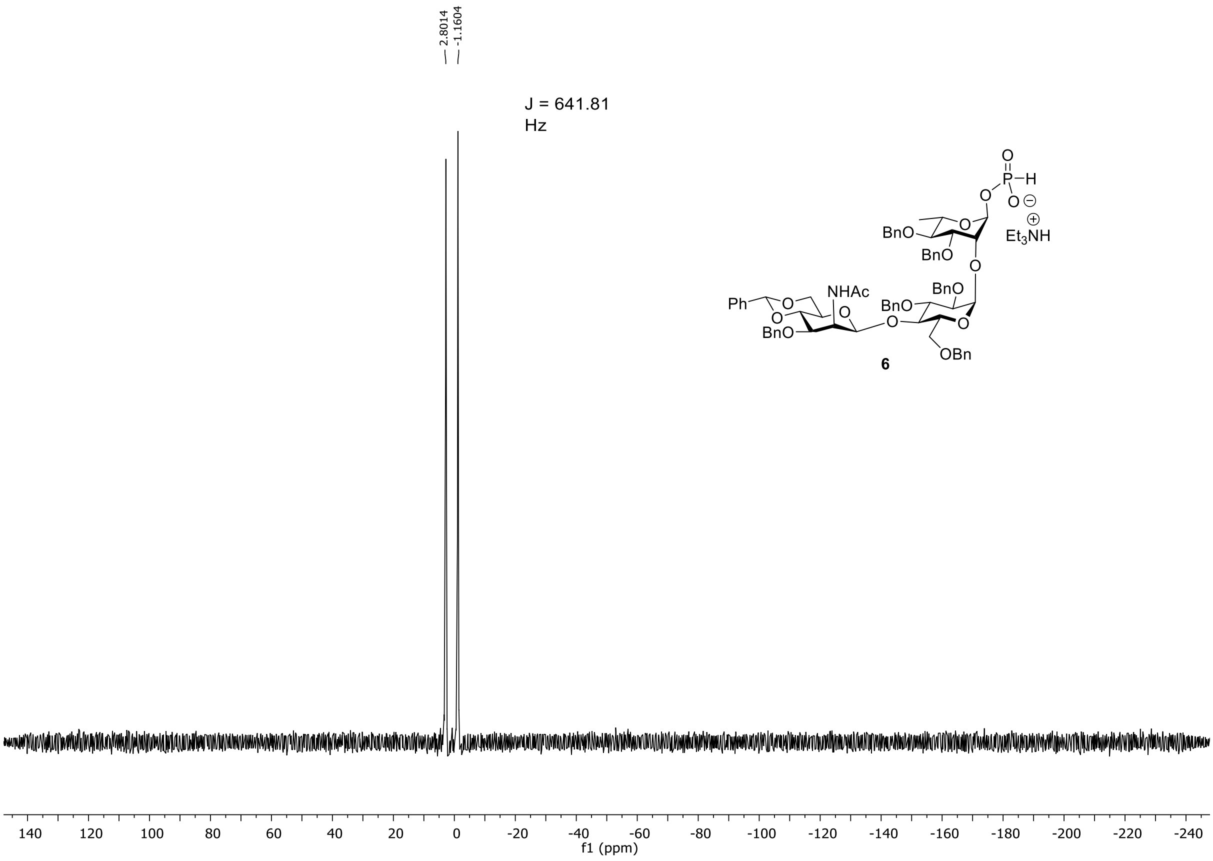

13C NMR, 101 MHz, $CDCl_3$

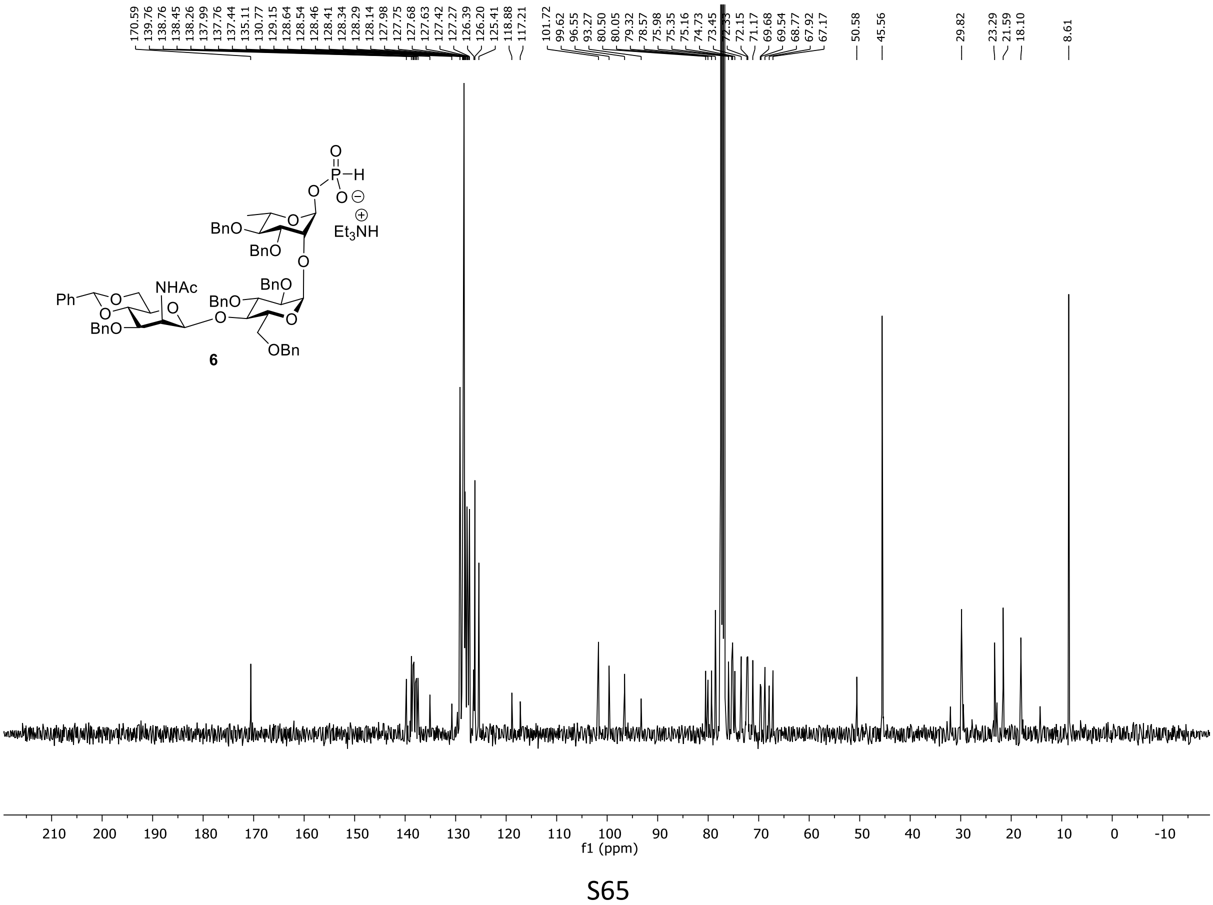

CH-HSQC NMR, 400 MHz, $CDCl_3$

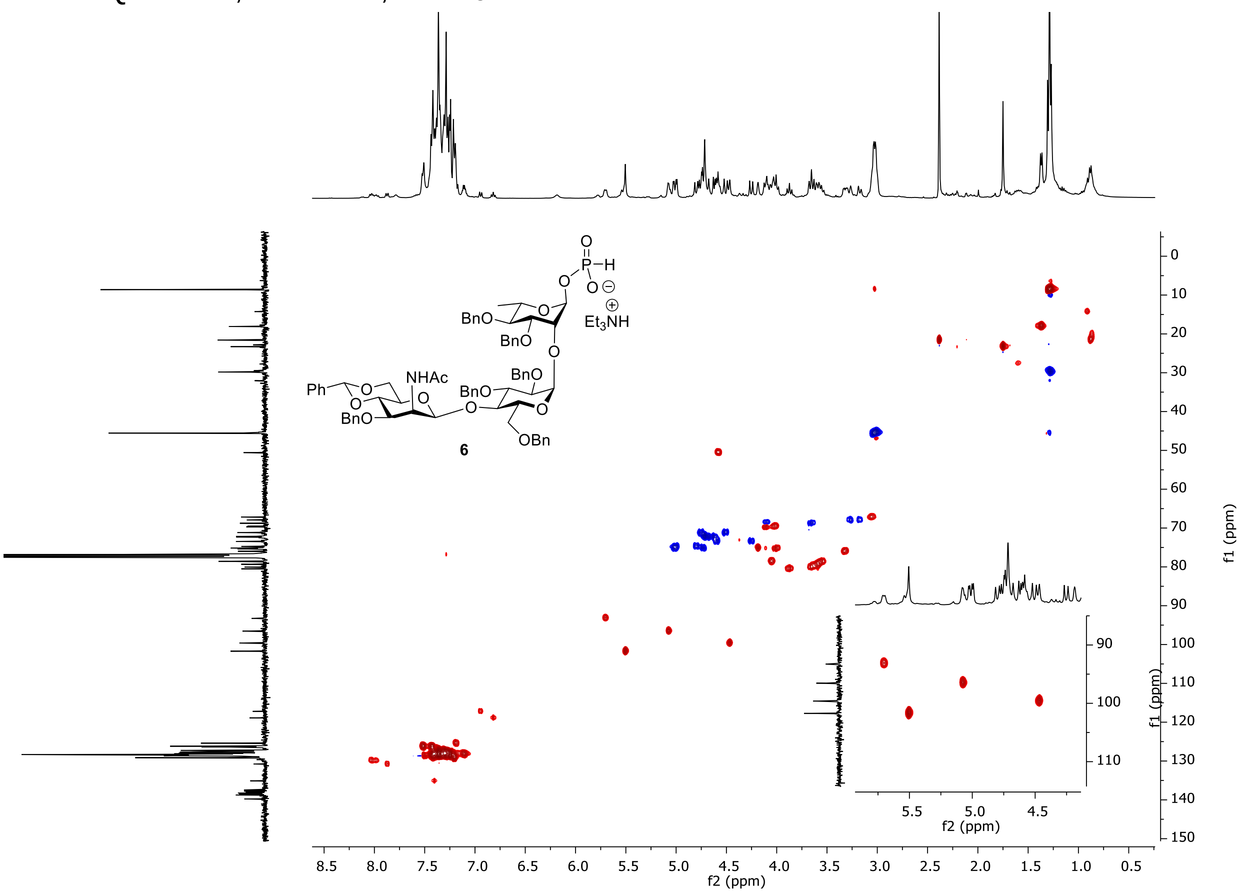

CH-HSQC NMR, 400 MHz, $CDCl_3$

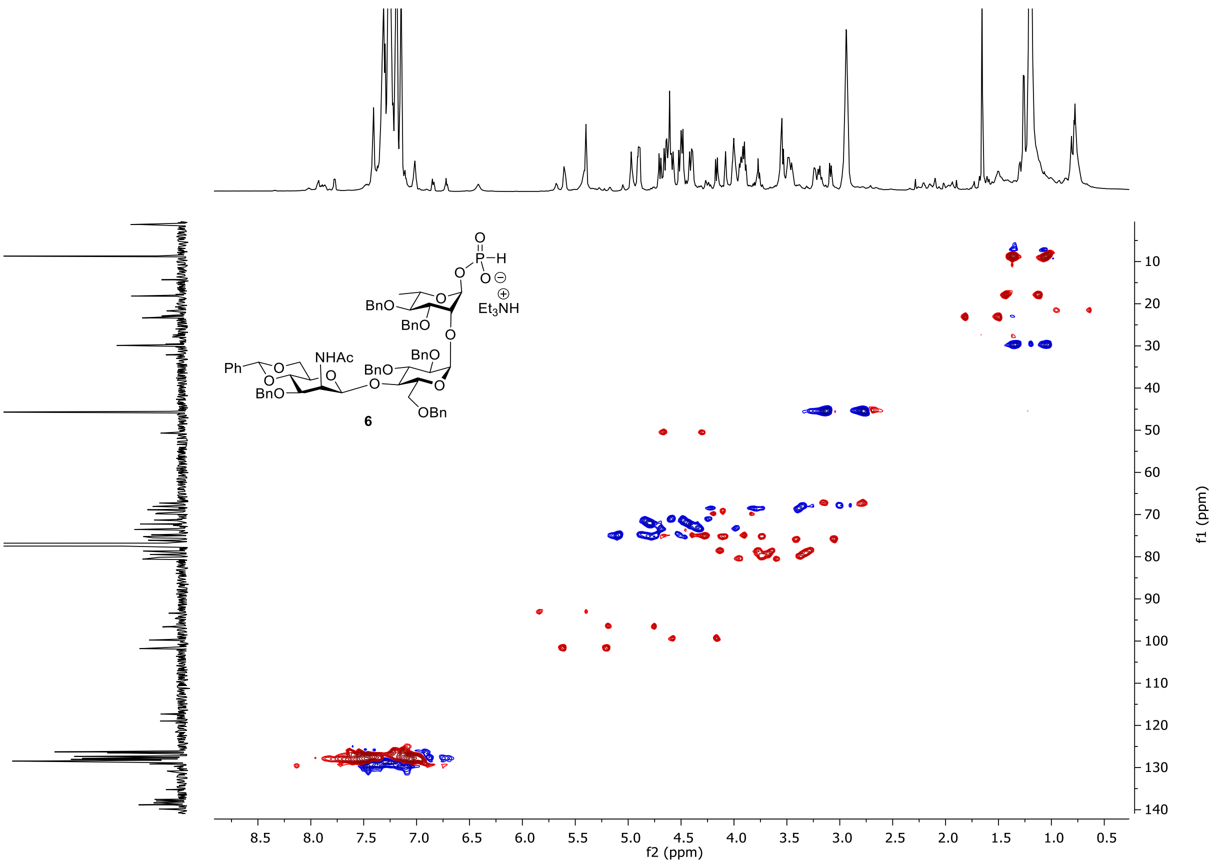

1H NMR, 600 MHz, CDCl3

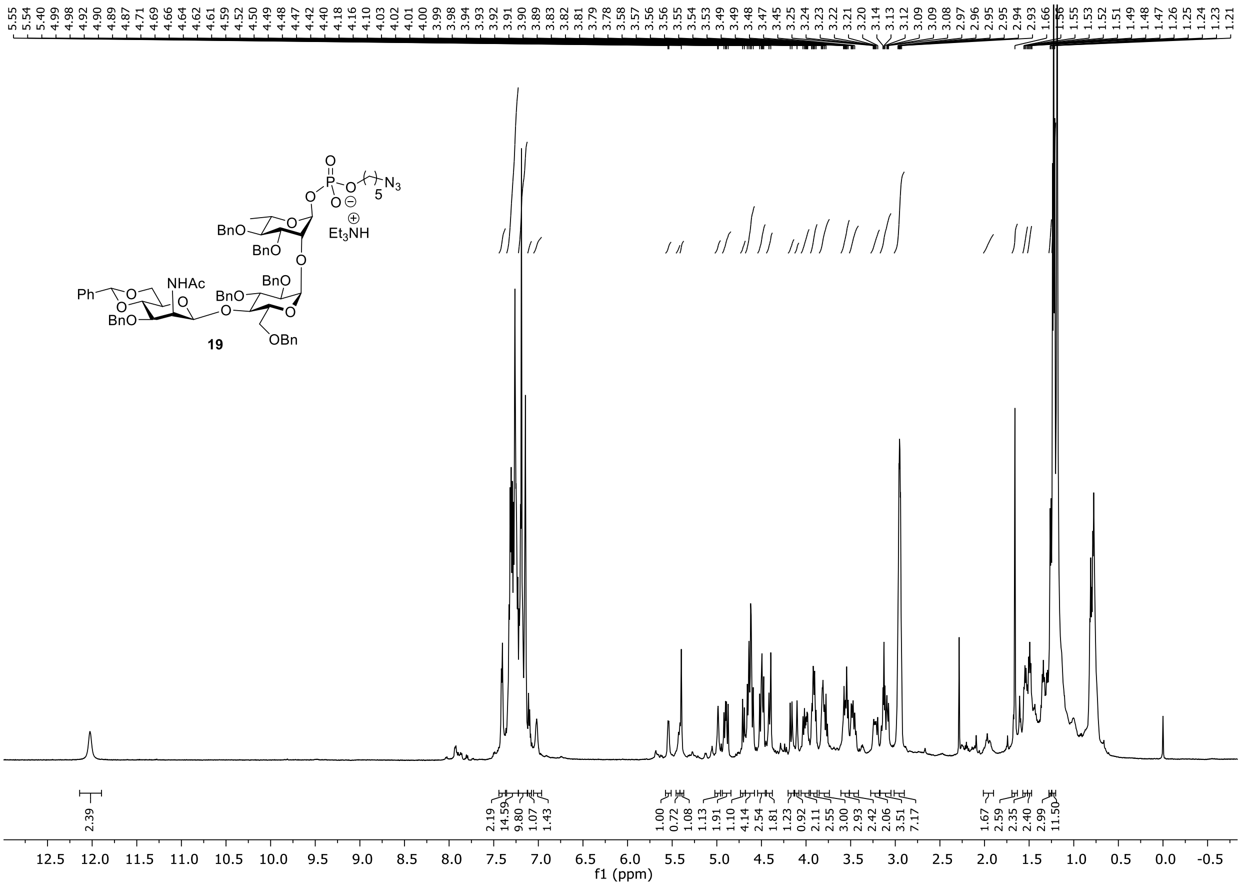

31P NMR, 162 MHz, CDCl3

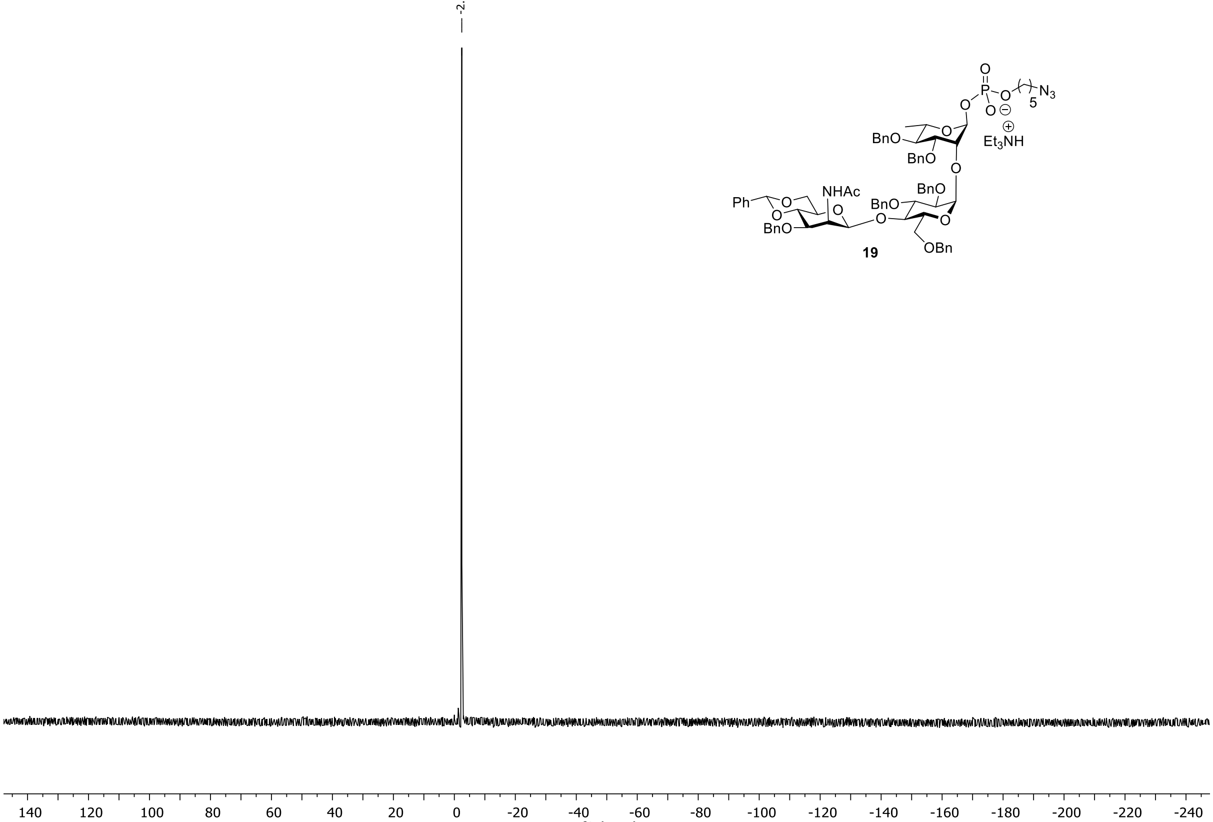

13C NMR, 151 MHz, CDCl₃

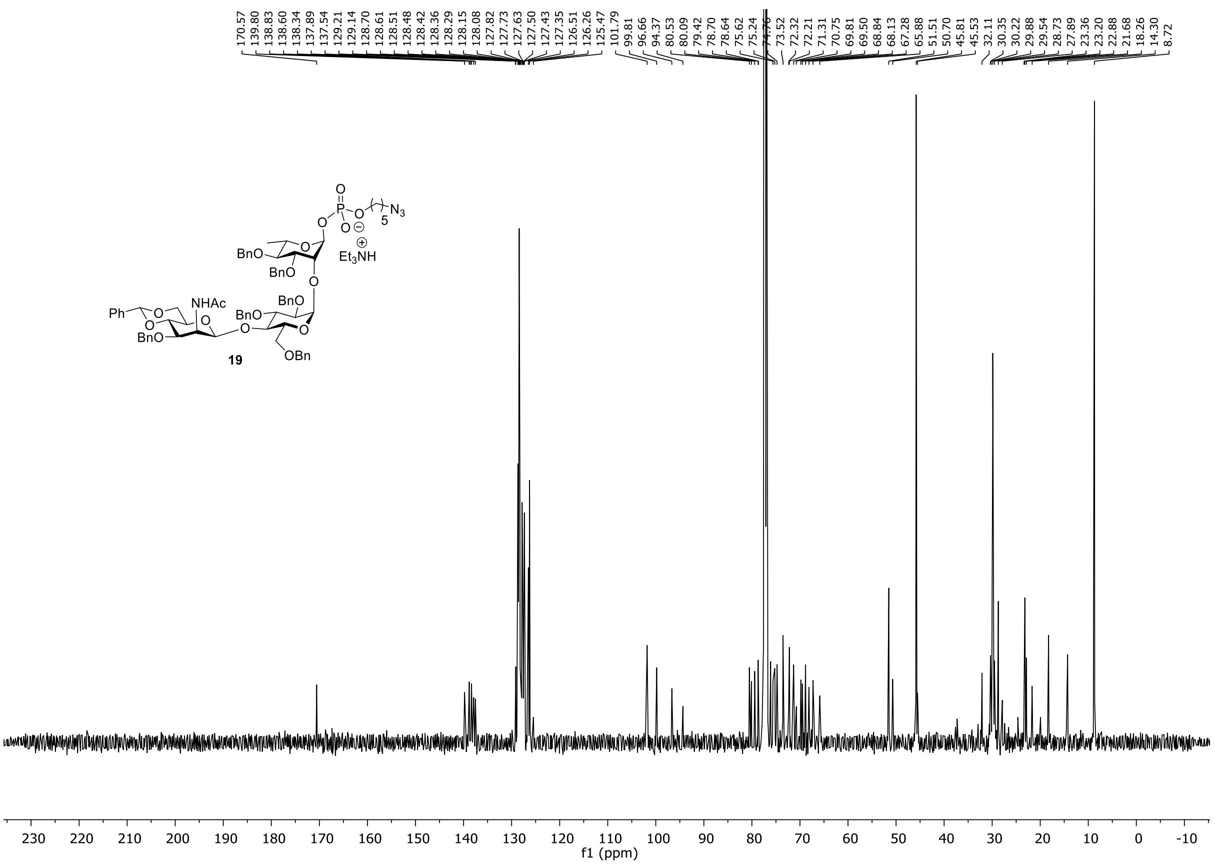

CH-HSQC NMR, 600 MHz, CDCl₃

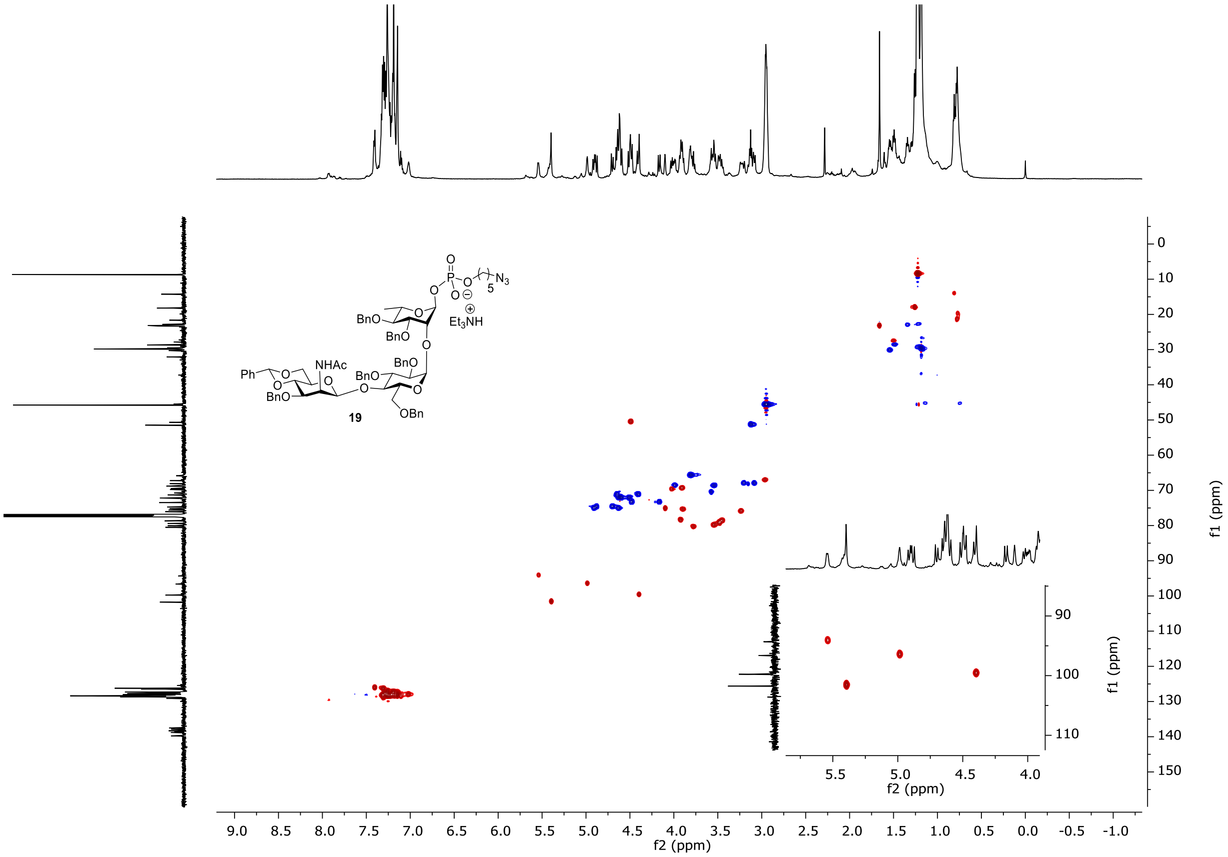

CH-HSQC NMR, 600 MHz, $CDCl_3$

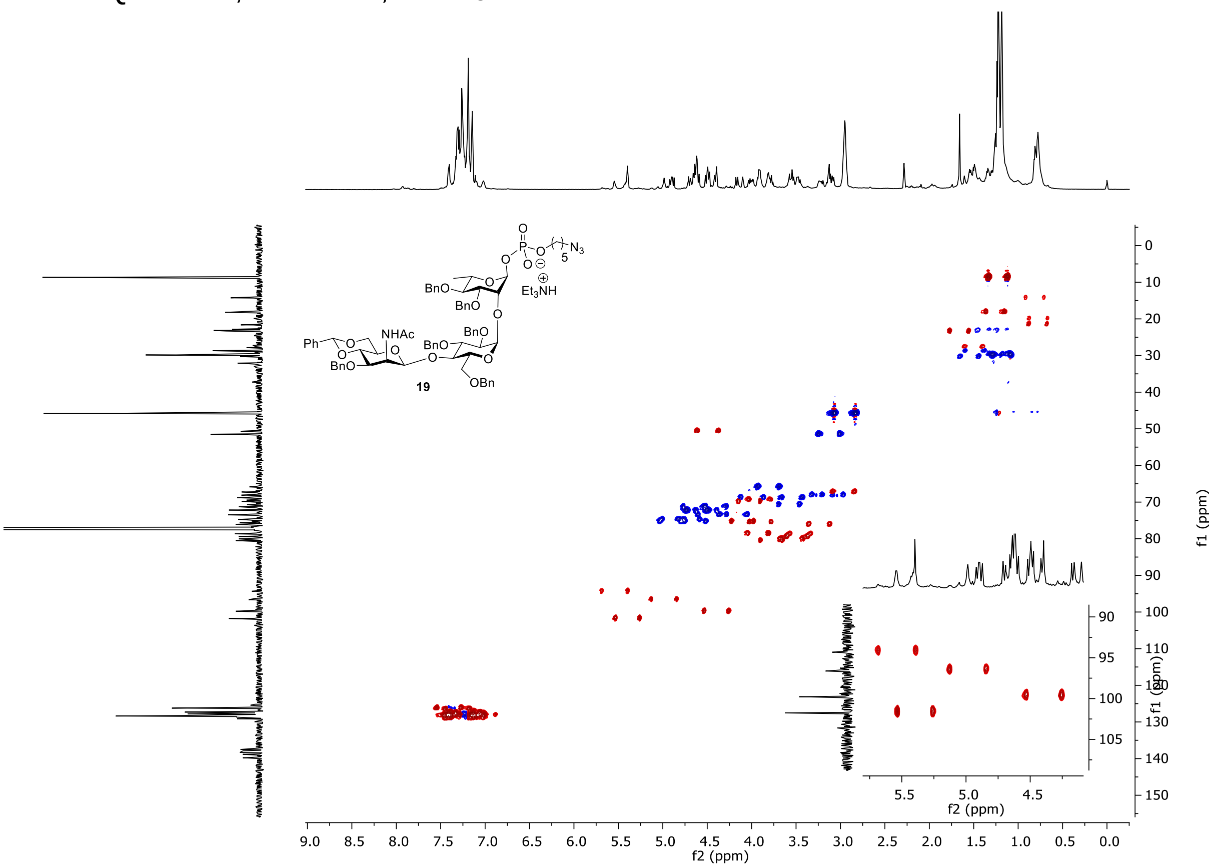

CH-HSQC NMR, 600 MHz, $CDCl_3$ (expansion)

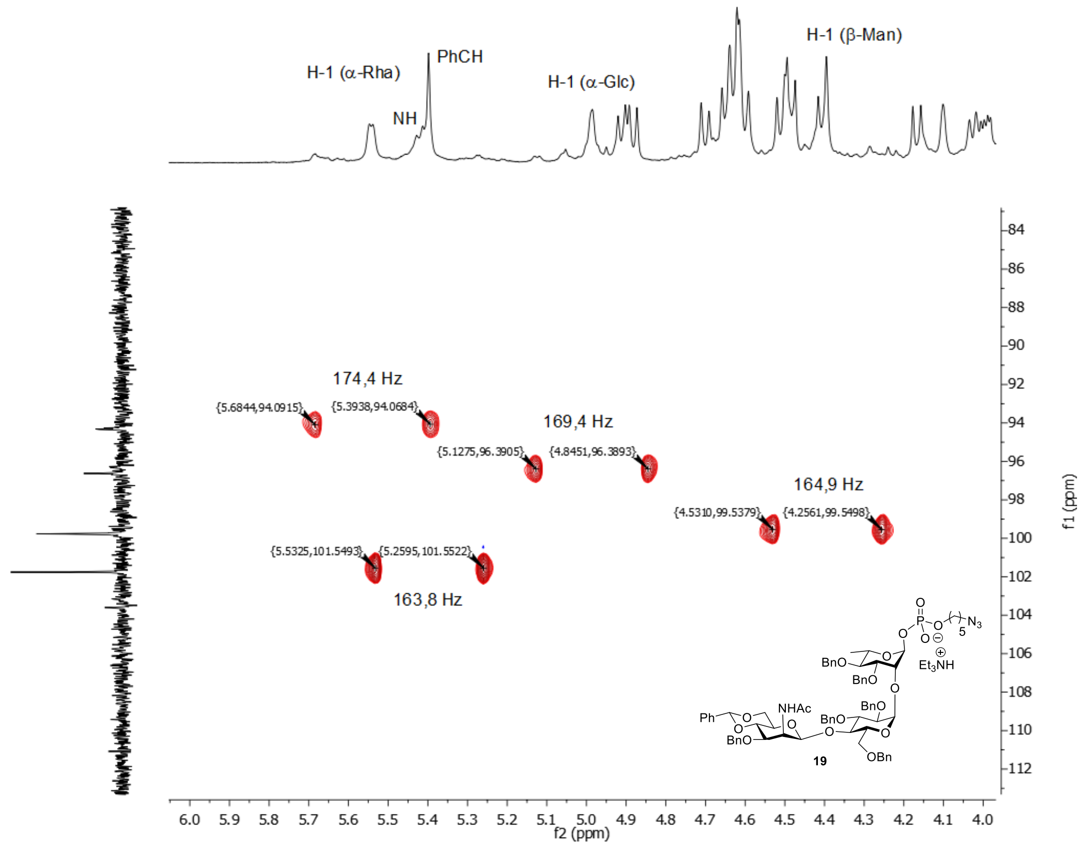

1H NMR, 600 MHz, D2O

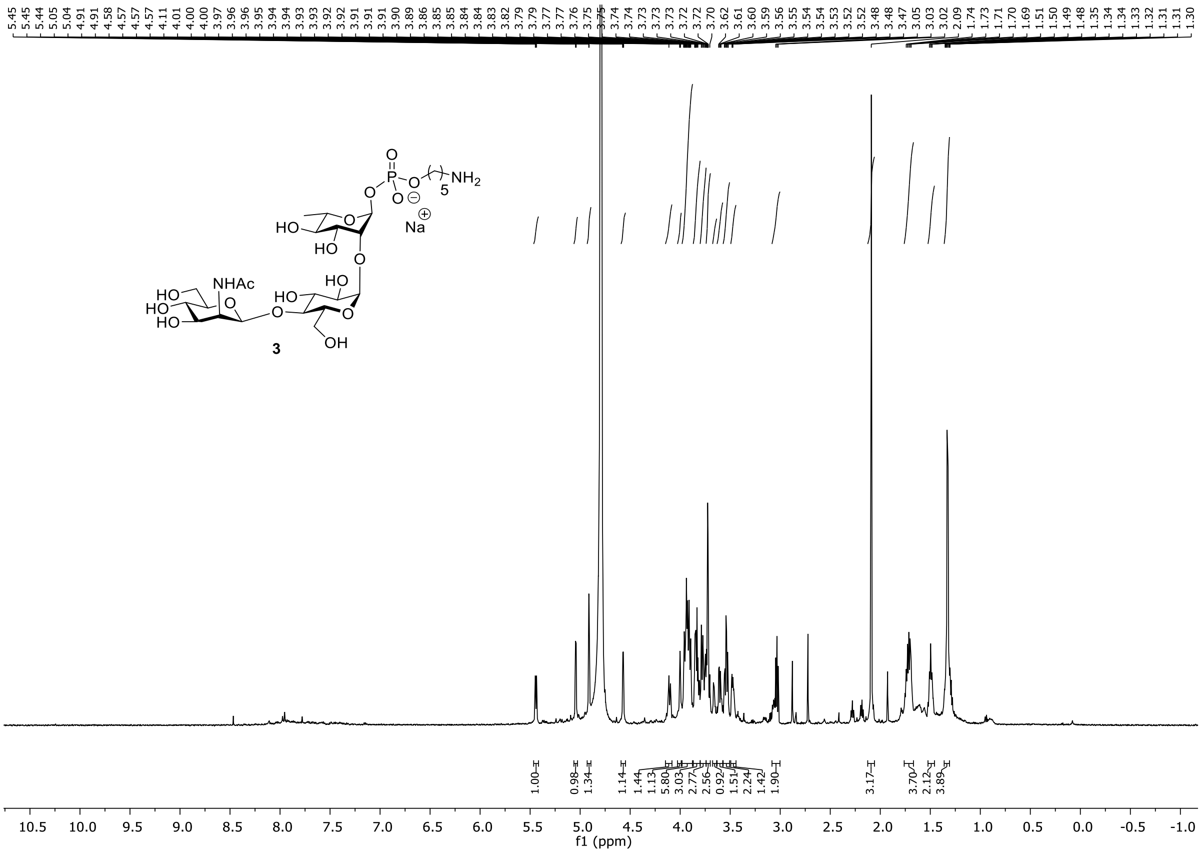

31P NMR, 243 MHz, D2O

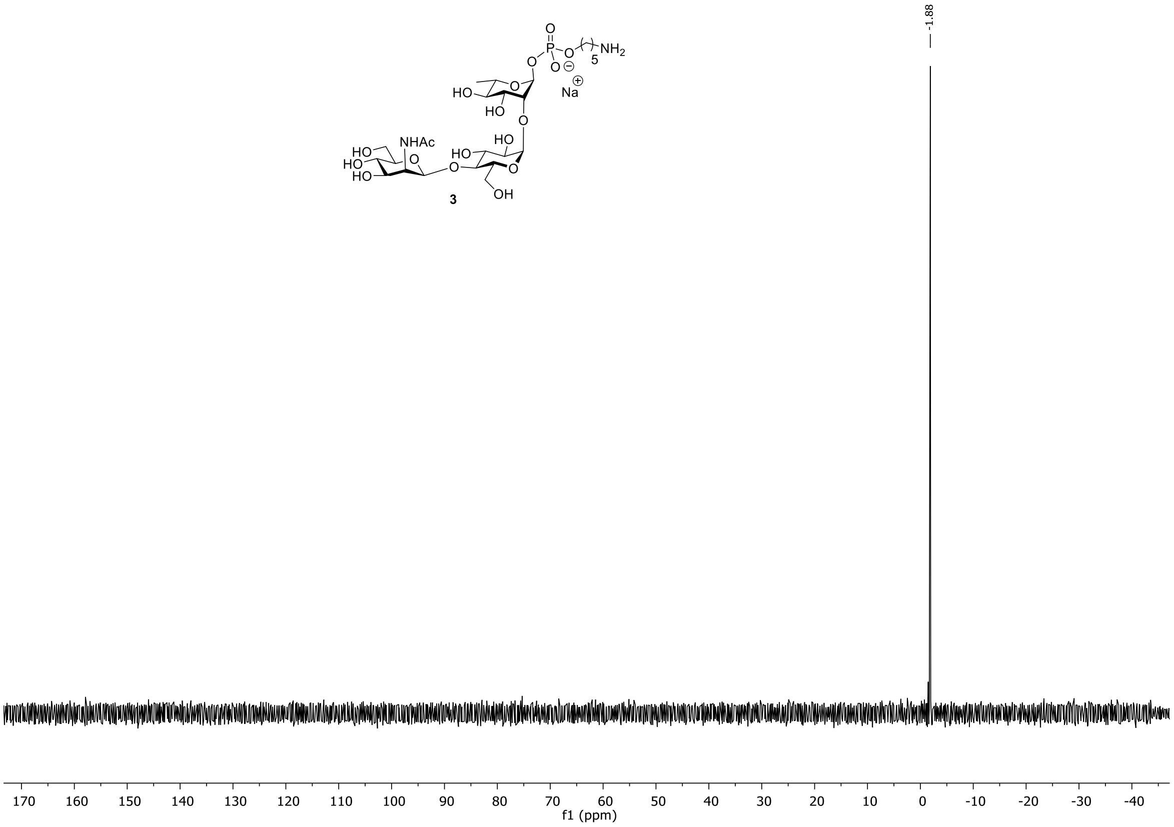

13C NMR, 176 MHz, D2O

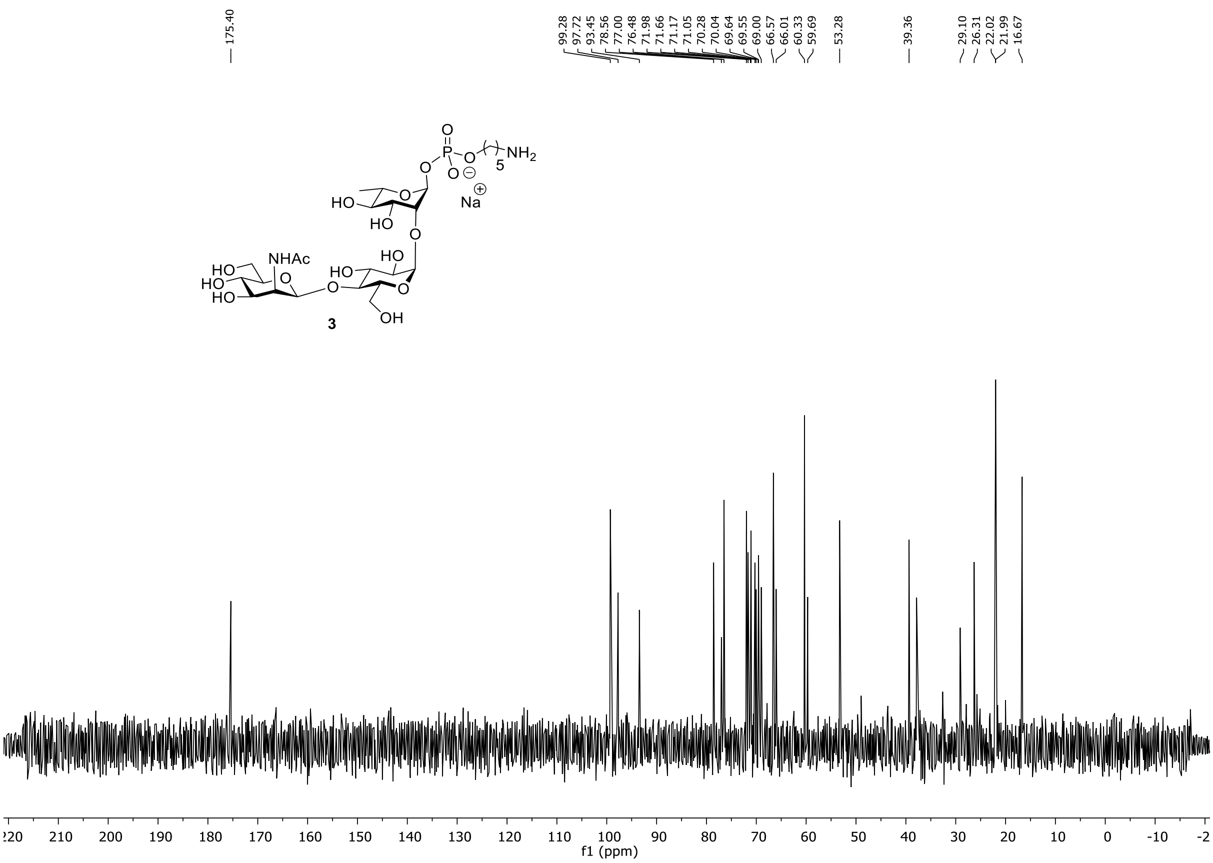

CH-HSQC NMR, 600 MHz, D2O

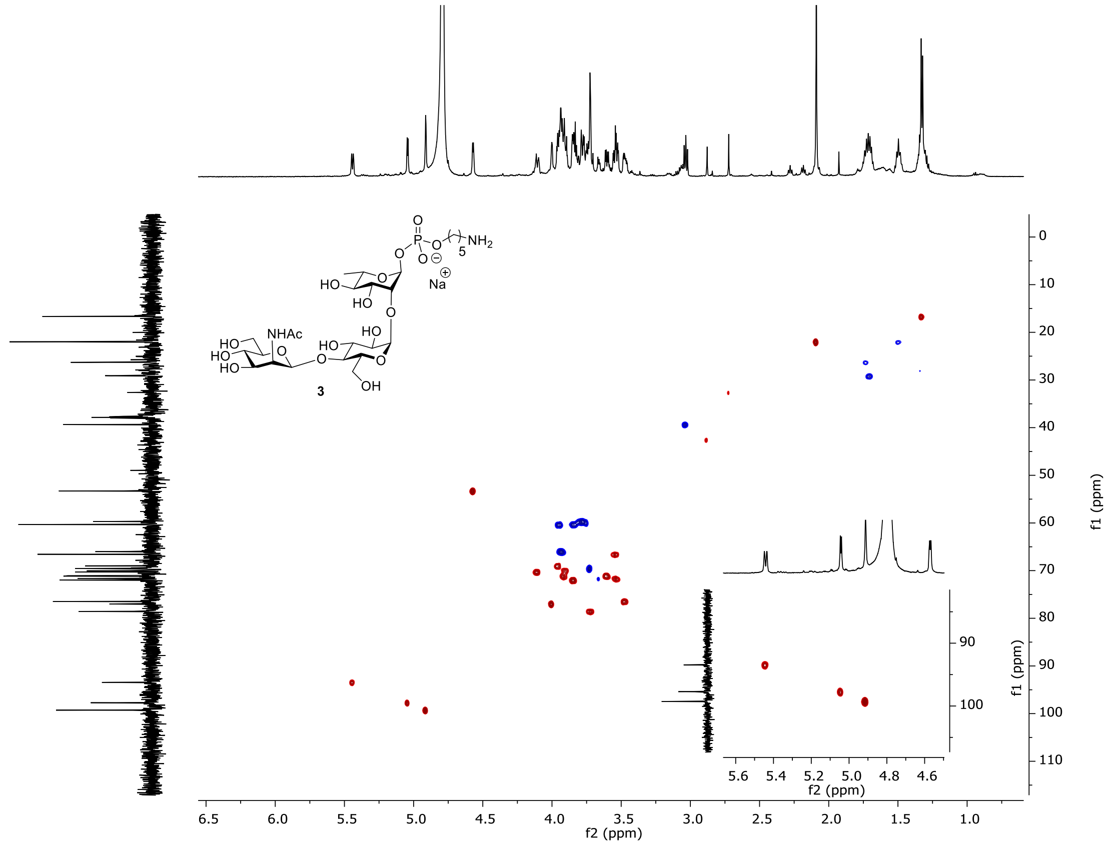

CH-HSQC NMR, 600 MHz, D_2O

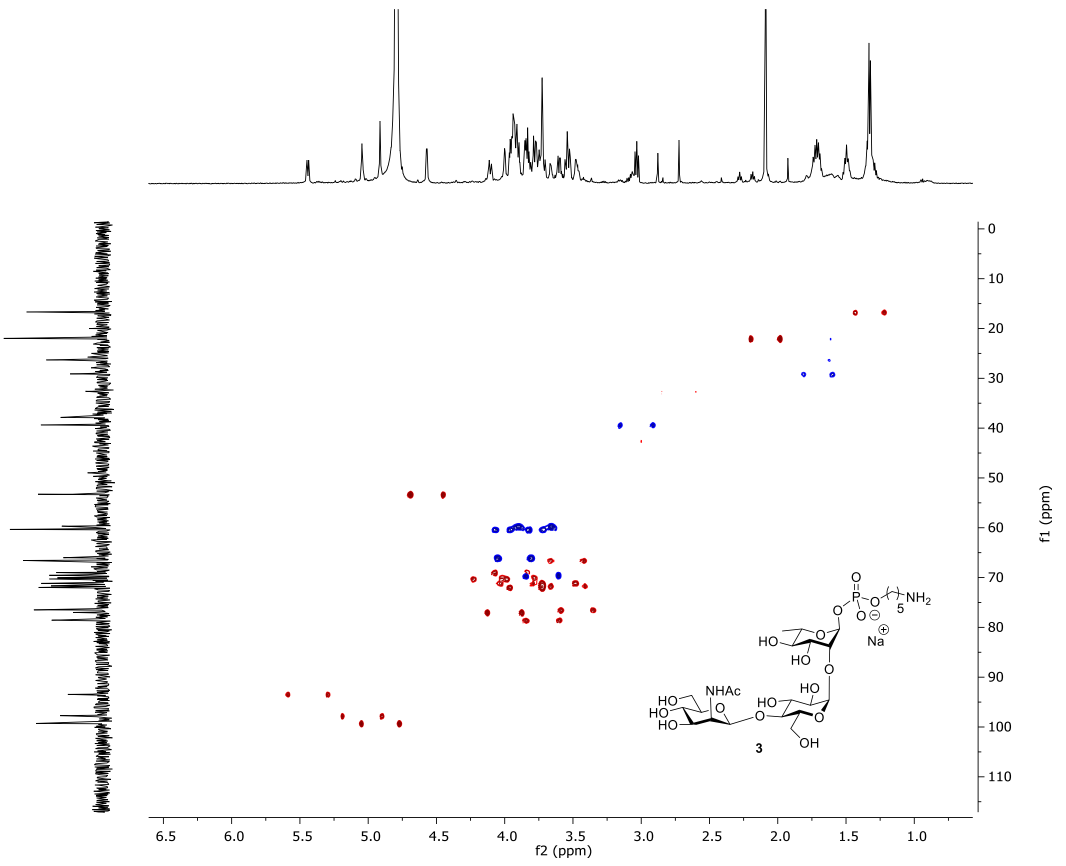

CH-HSQC NMR, 600 MHz, D_2O (Expansion)

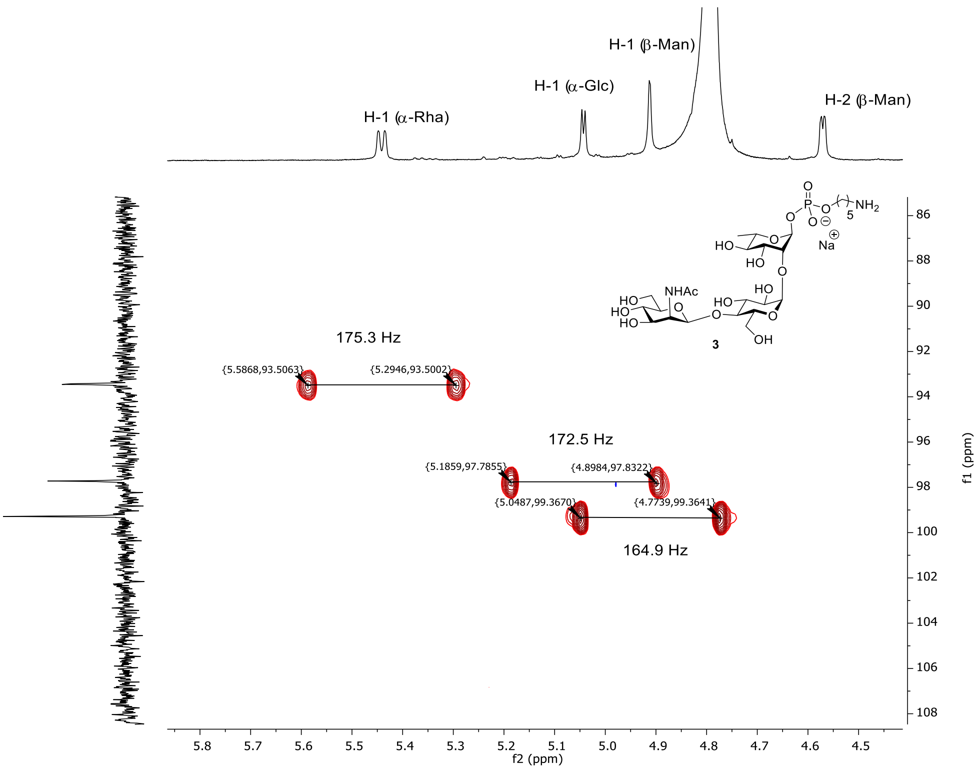

HRMS (Q-Tof)

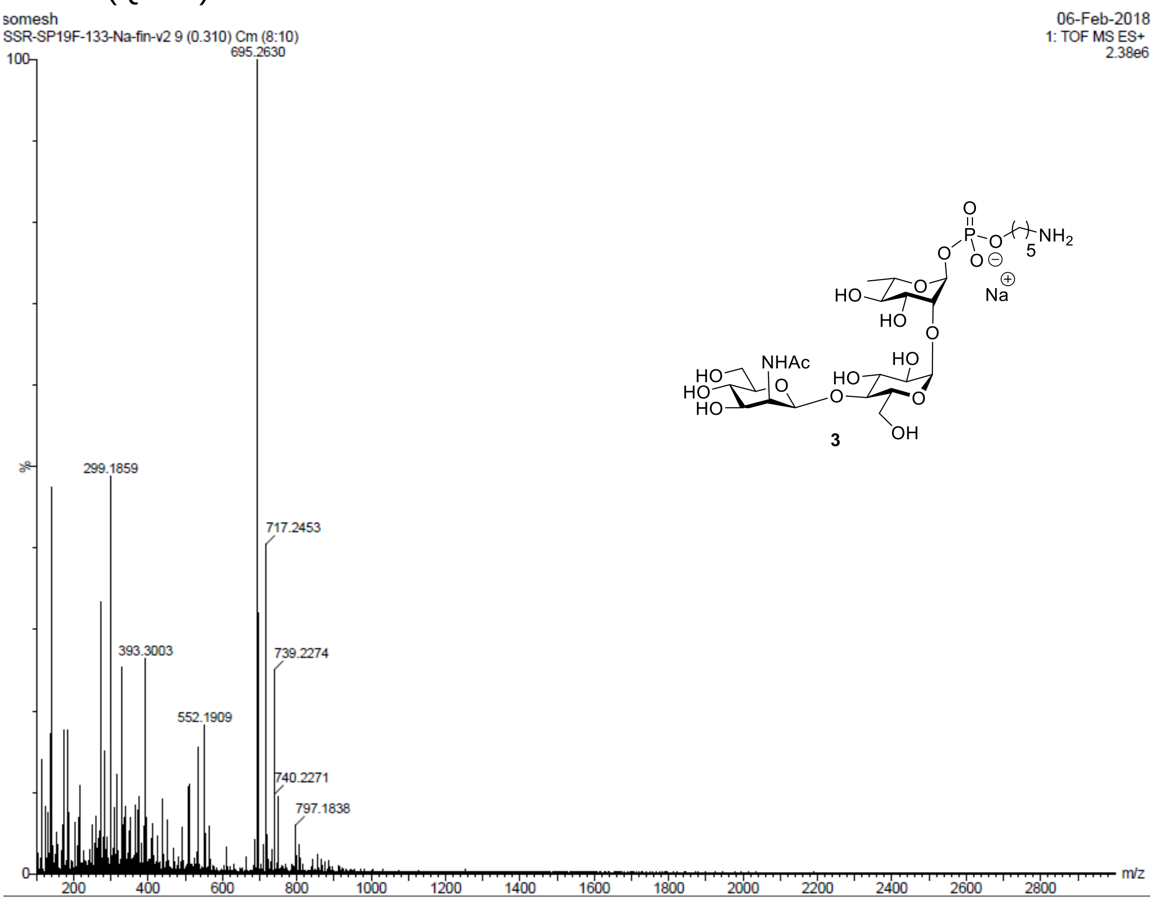

H NMR, 400 MHz, $CDCl_3$

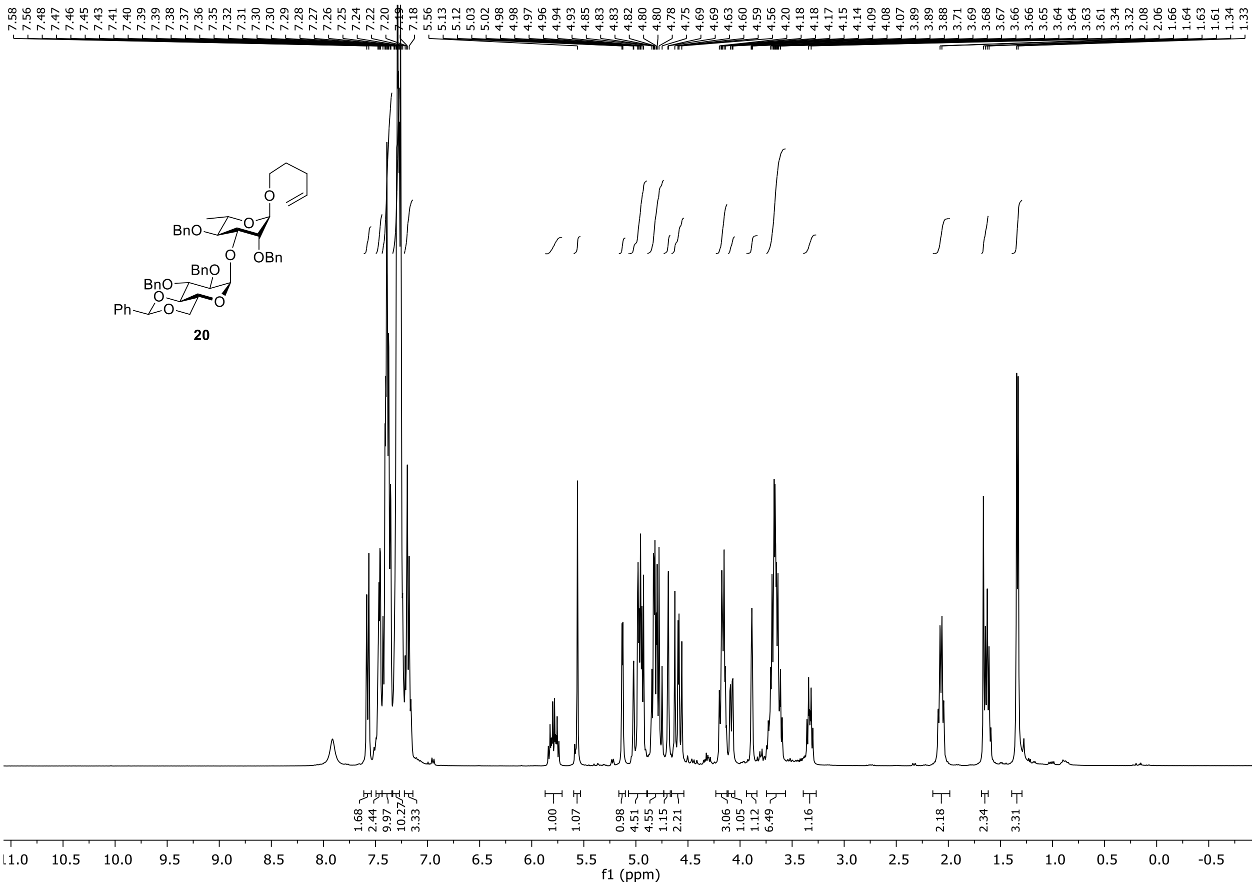

13C NMR, 101 MHz, CDCl3

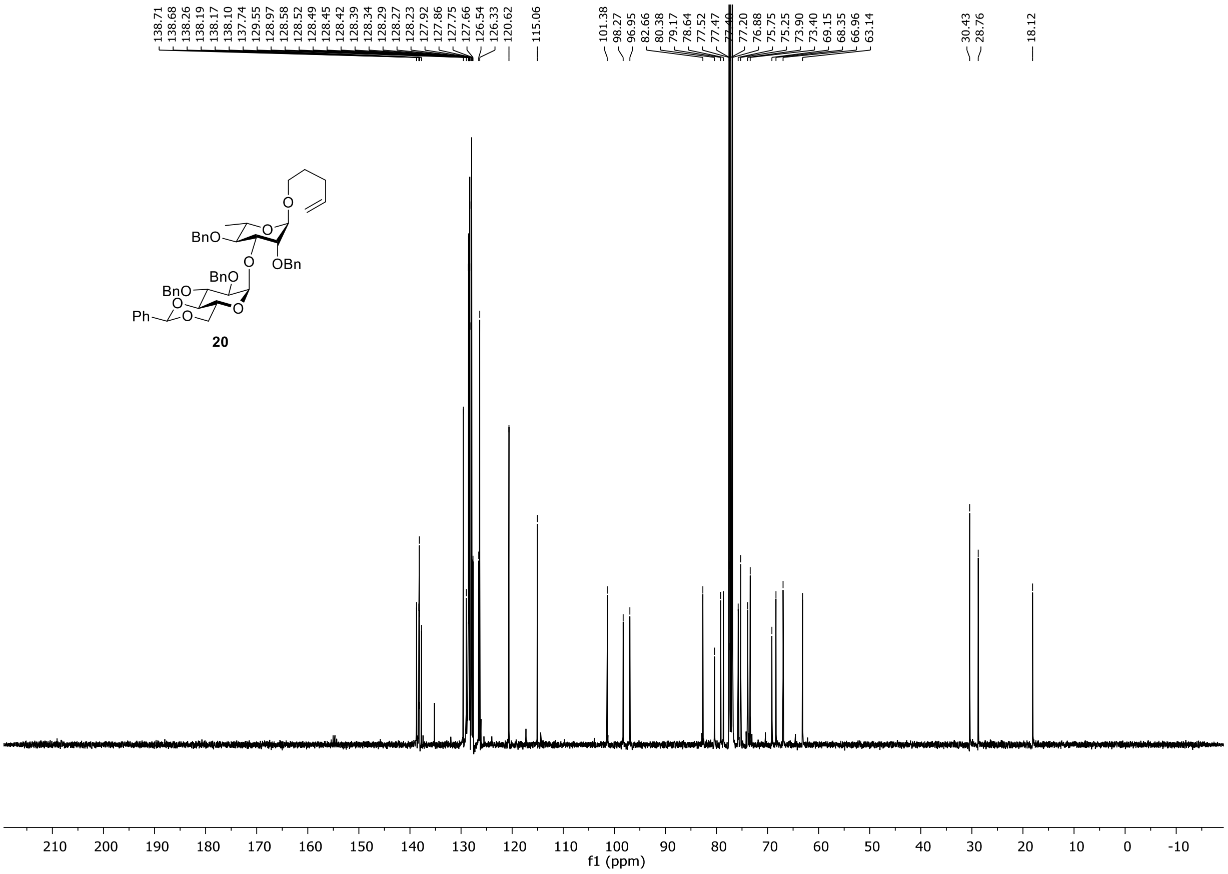

CH-HSQC NMR, 400 MHz, CDCl3

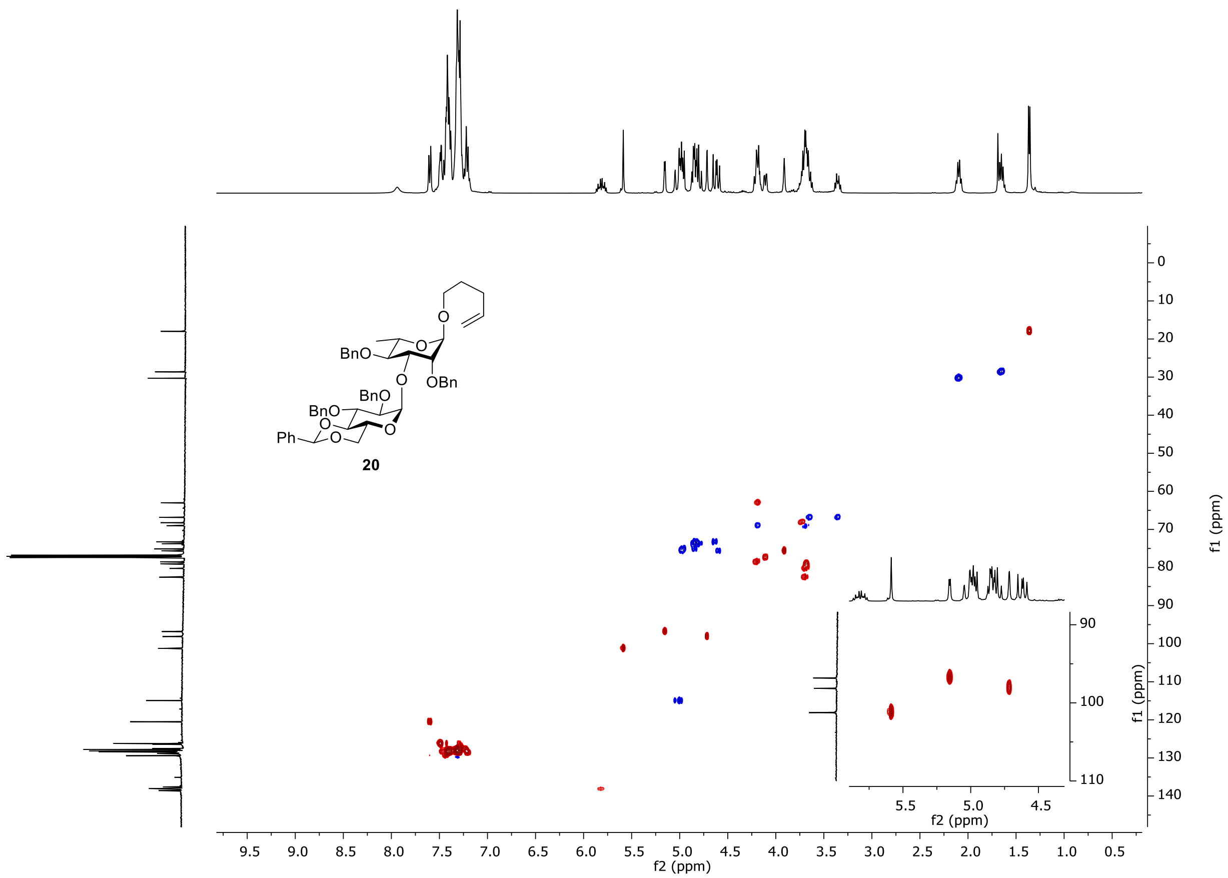

CH-HSQC NMR, 400 MHz, CDCl3

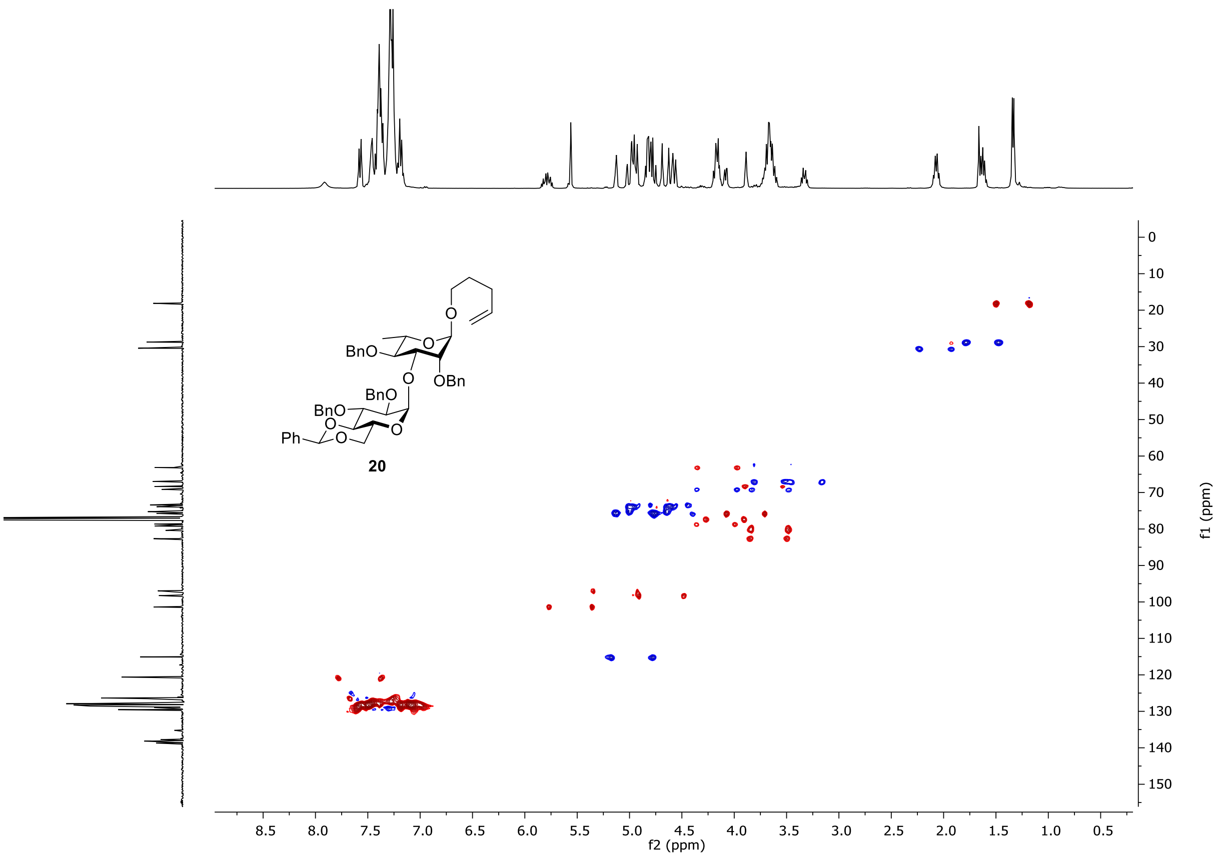

CH-HSQC NMR, 400 MHz, CDCl3 (expansion)

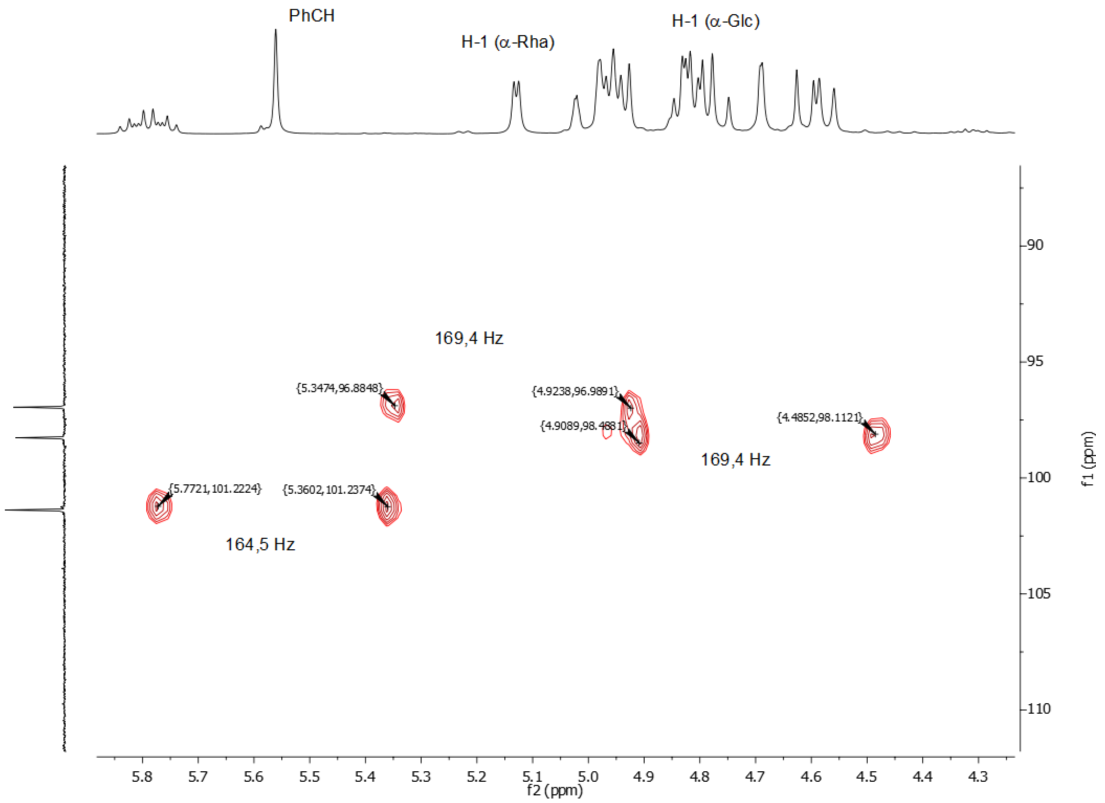

1H NMR, 600 MHz, CDCl3

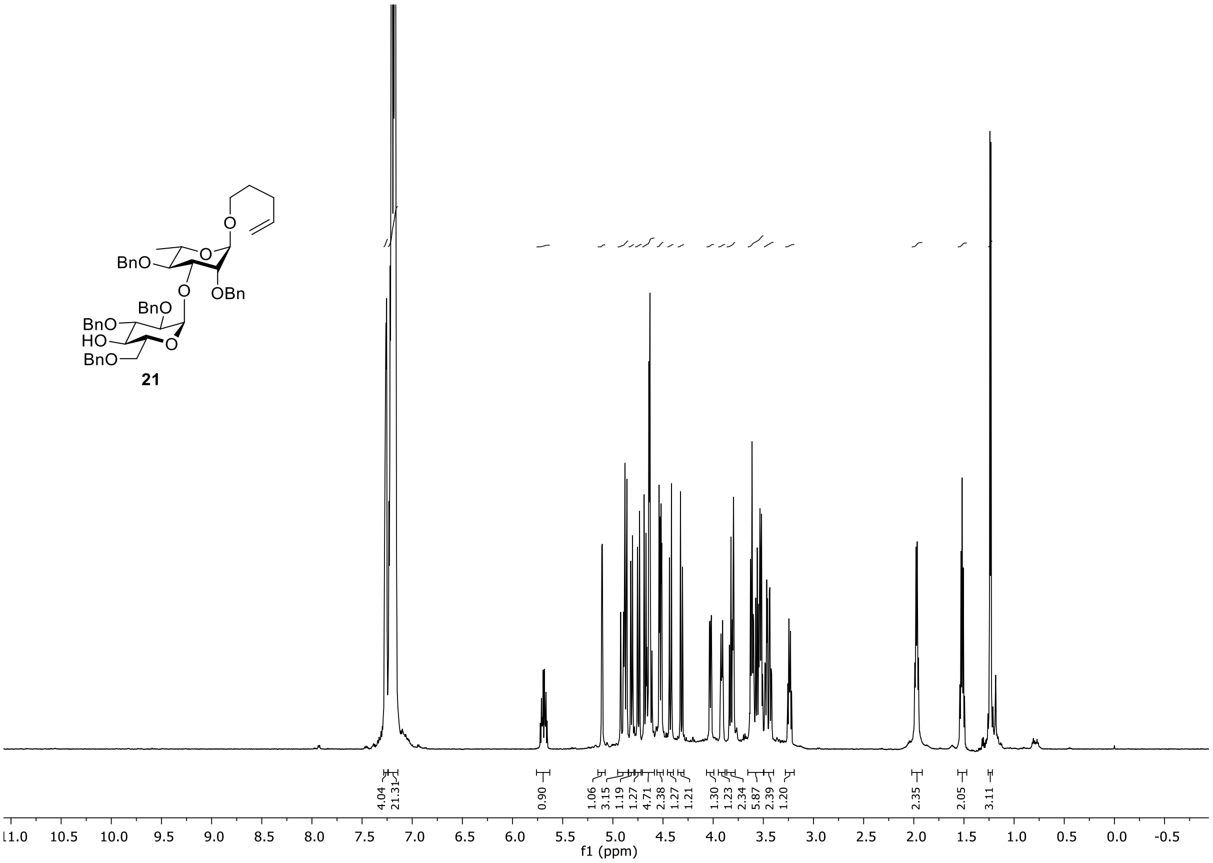

13C NMR, 151 MHz, CDCl3

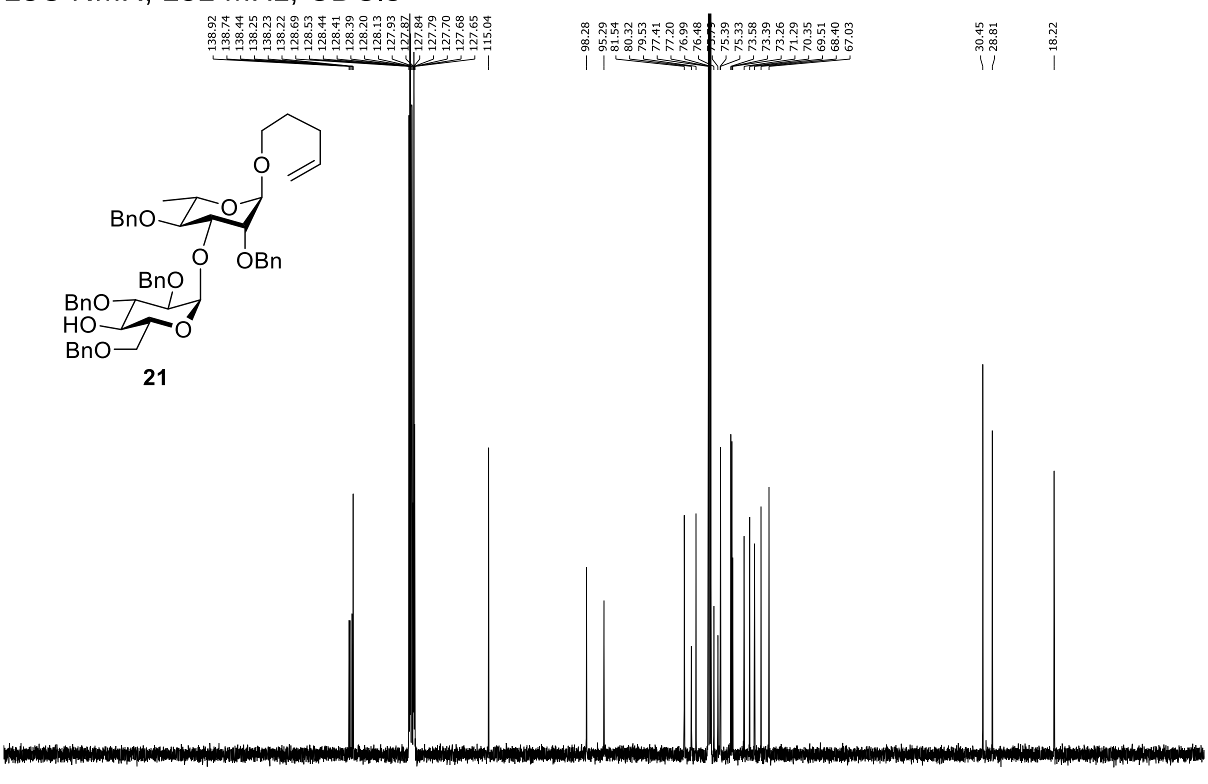

CH-HSQC NMR, 400 MHz, CDCl3

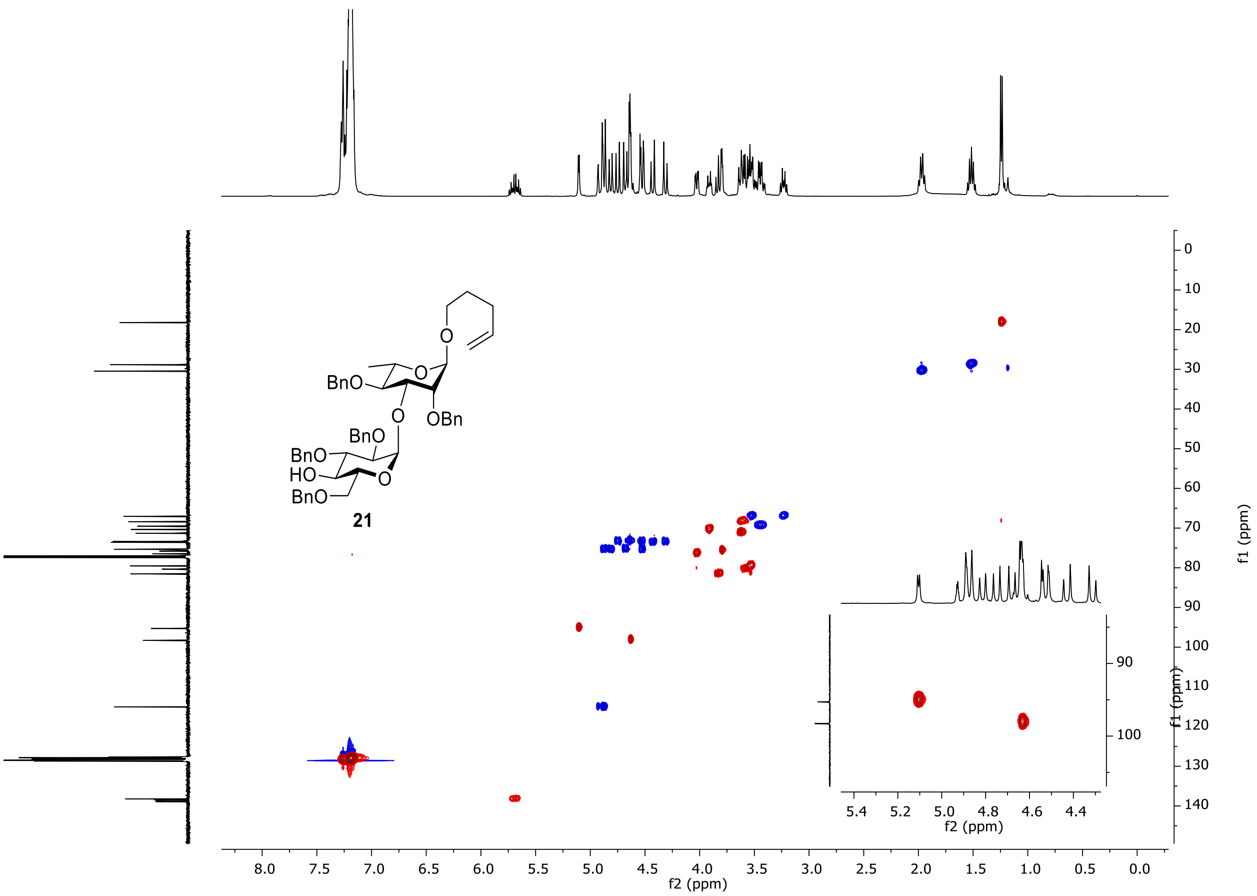

CH-HSQC NMR, 600 MHz, CDCl3

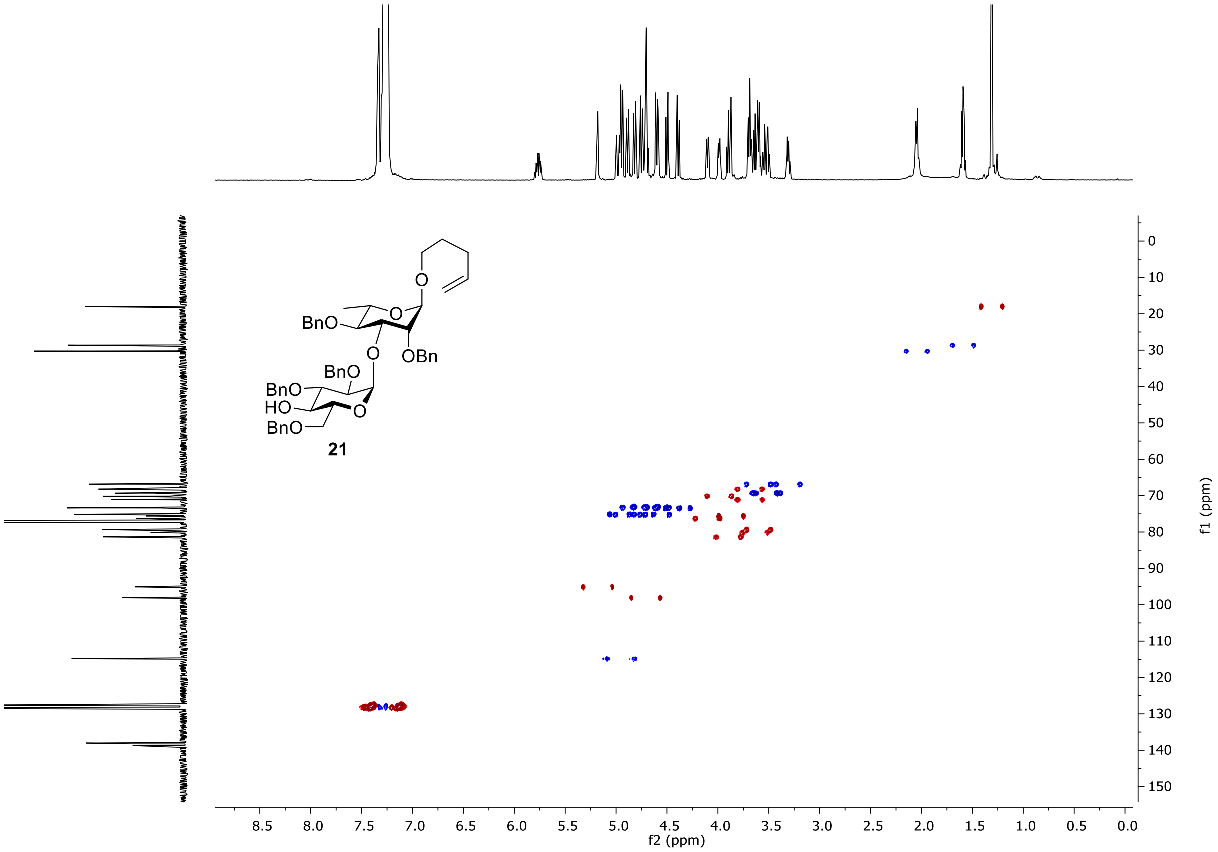

CH-HSQC NMR, 600 MHz, $CDCl_3$ (expansion)

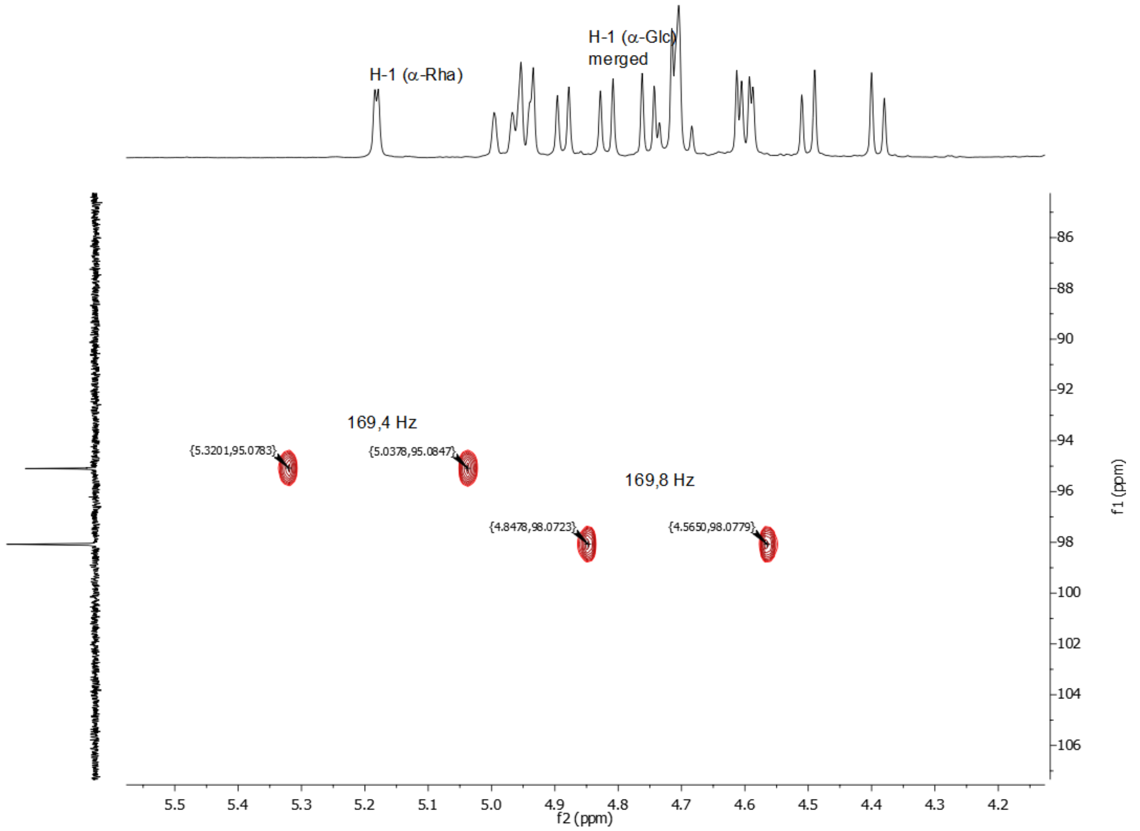

1H NMR, 400 MHz, $CDCl_3$

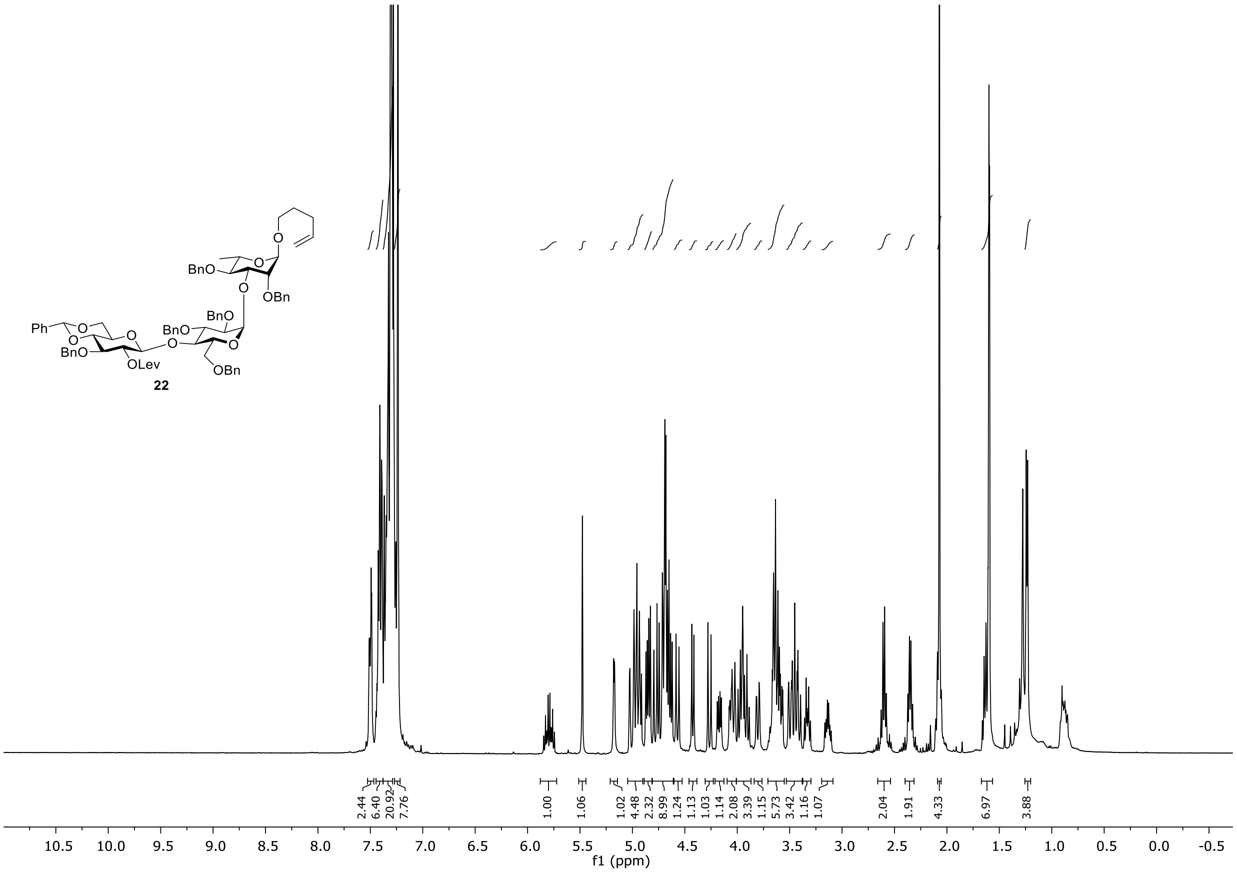

13C NMR, 101 MHz, CDCl3

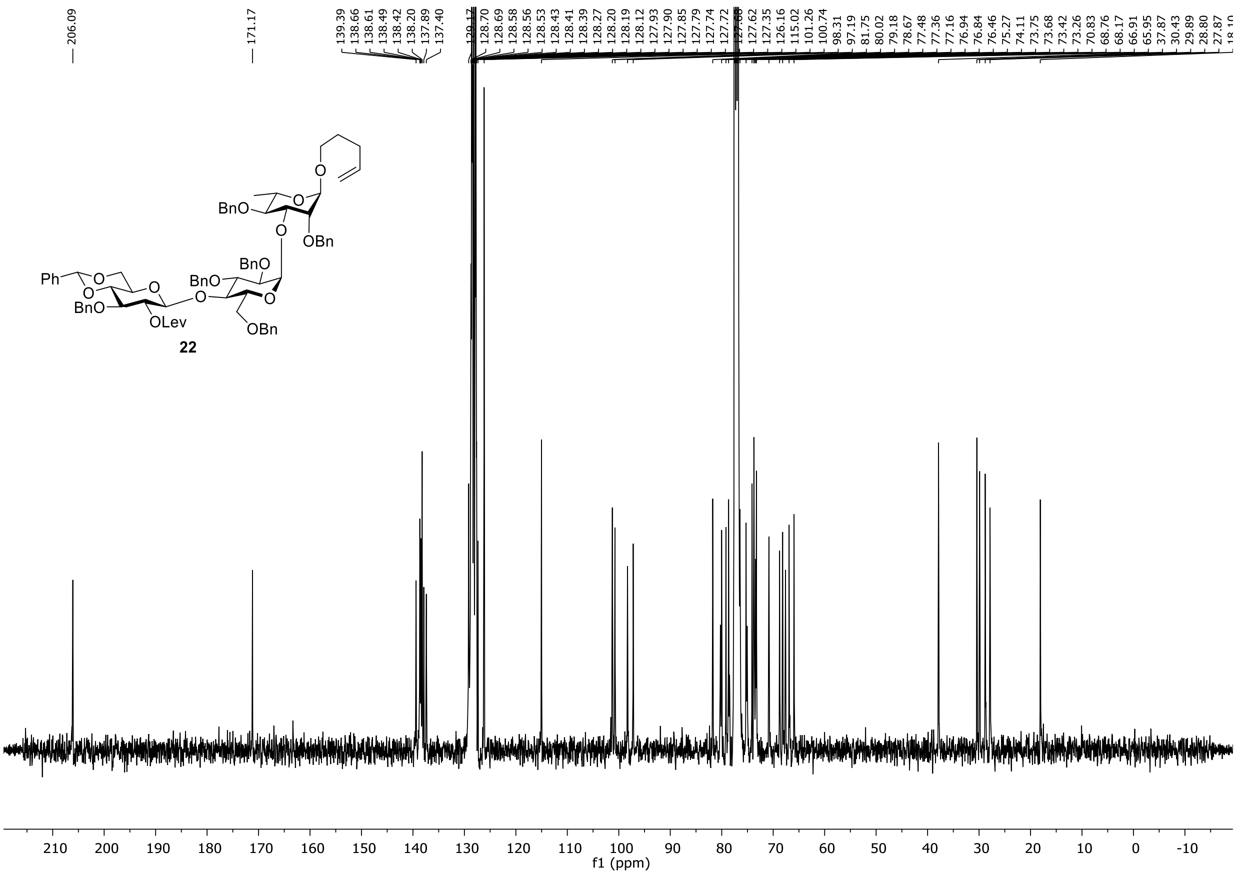

CH-HSQC NMR, 400 MHz, CDCl3

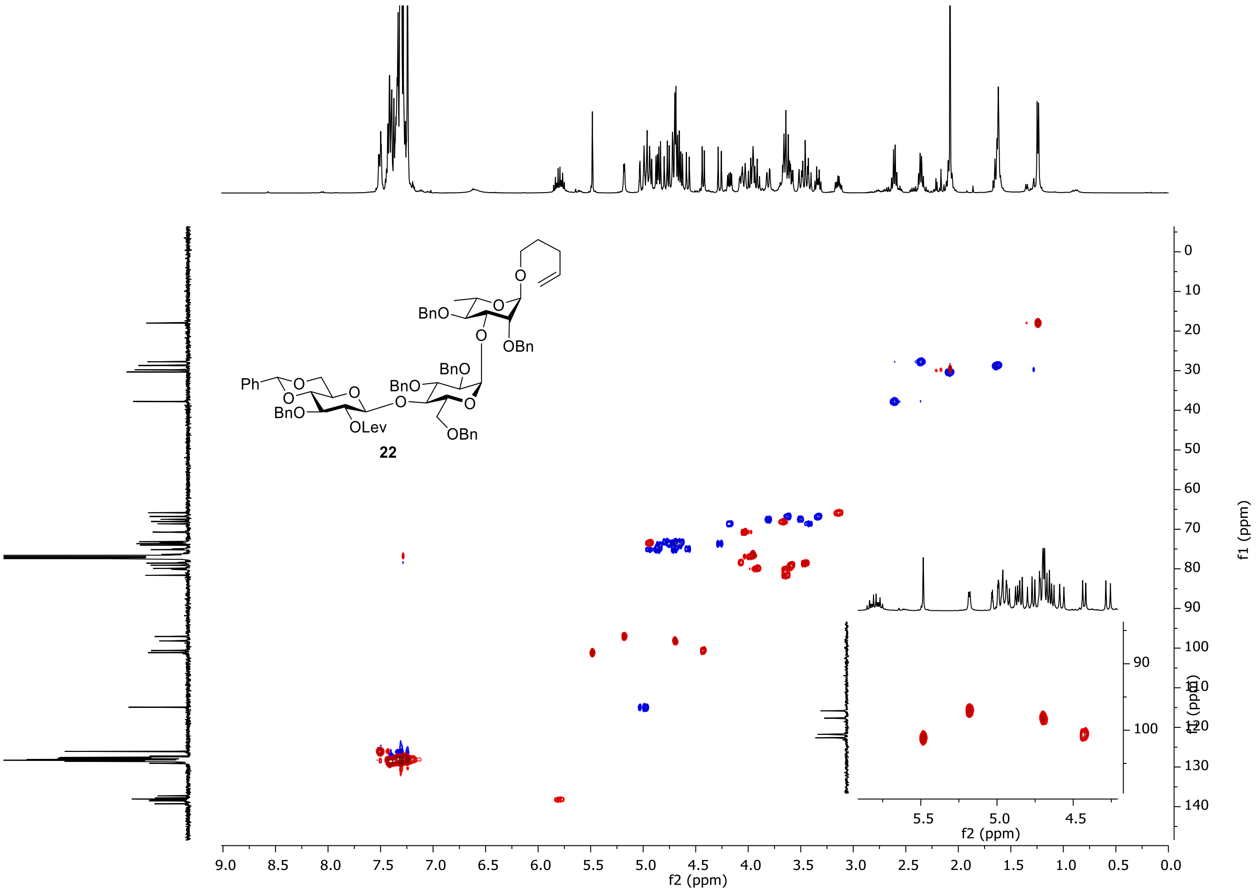

CH-HSQC NMR, 400 MHz, $CDCl_3$

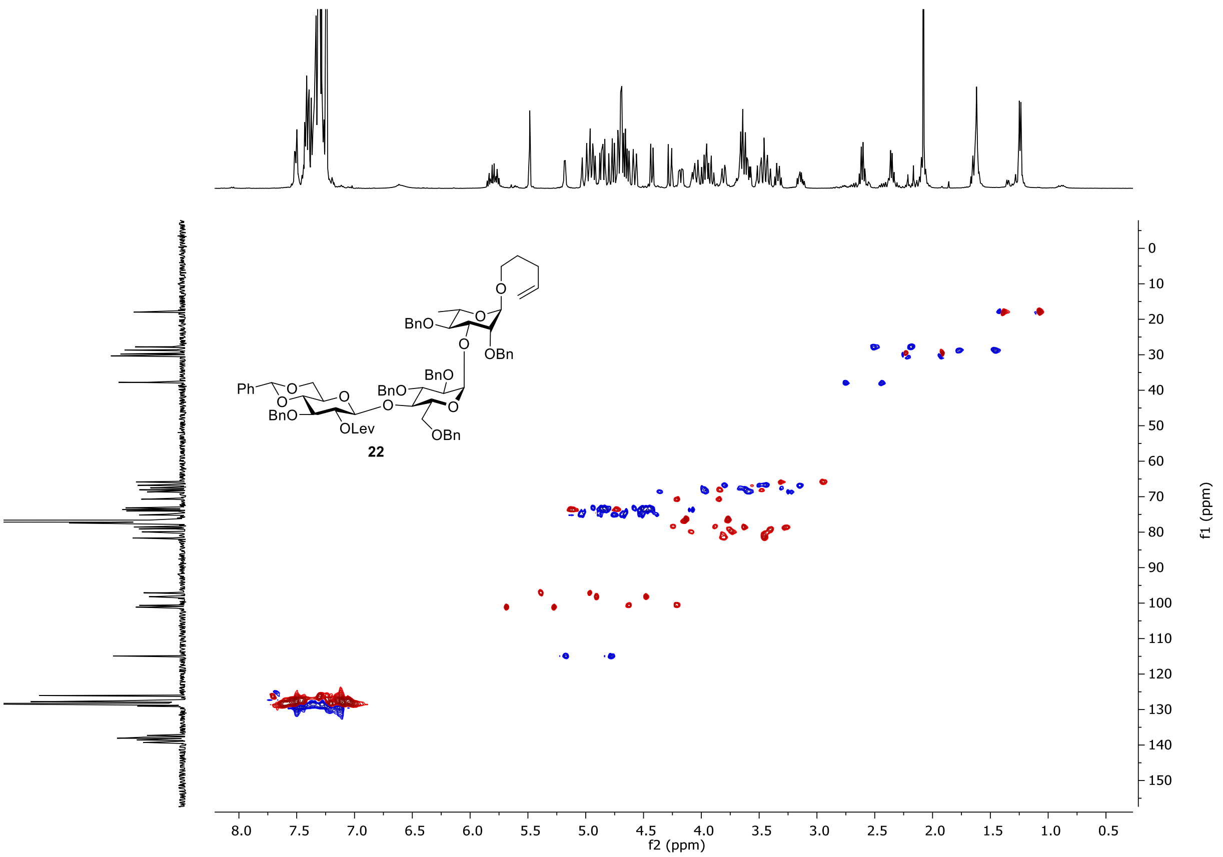

CH-HSQC NMR, 400 MHz, $CDCl_3$ (expansion)

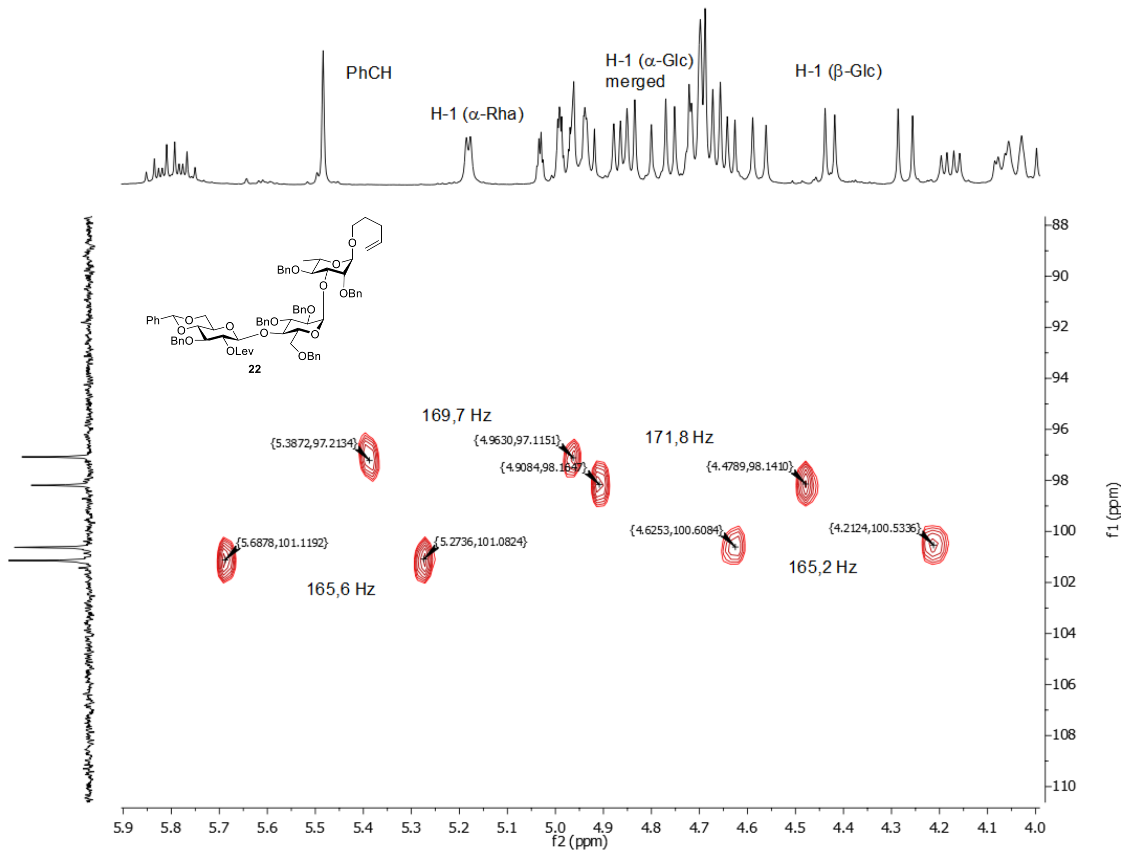

1H NMR, 400 MHz, $CDCl_3$

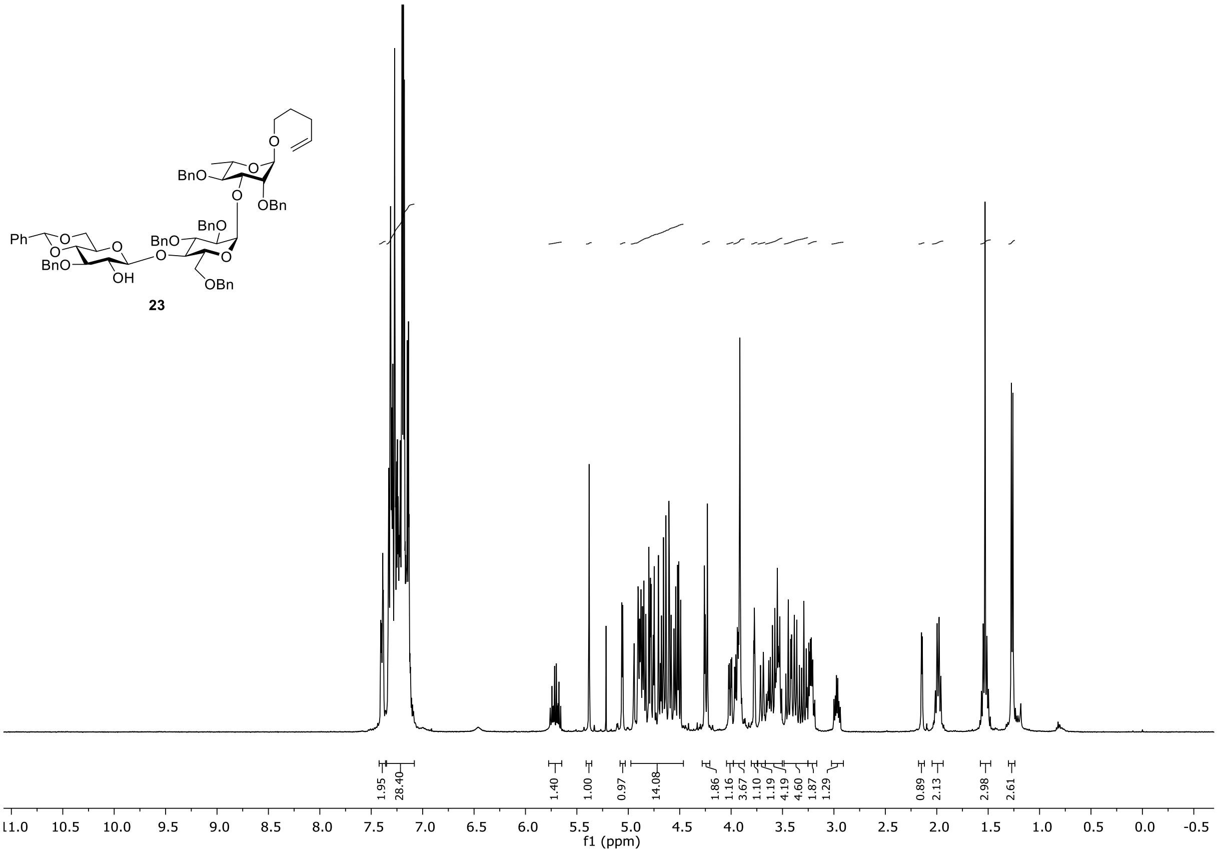

13C NMR, 101 MHz, $CDCl_3$

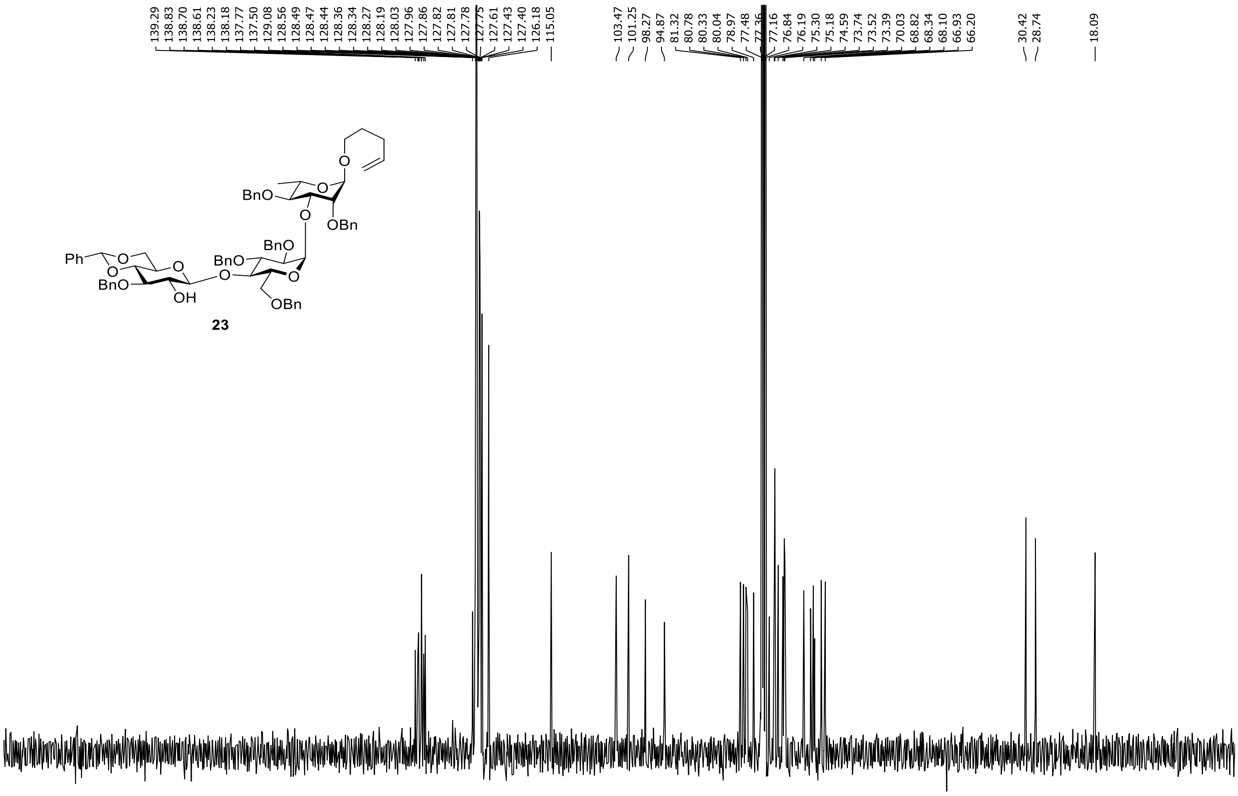

CH-HSQC NMR, 400 MHz, $CDCl_3$

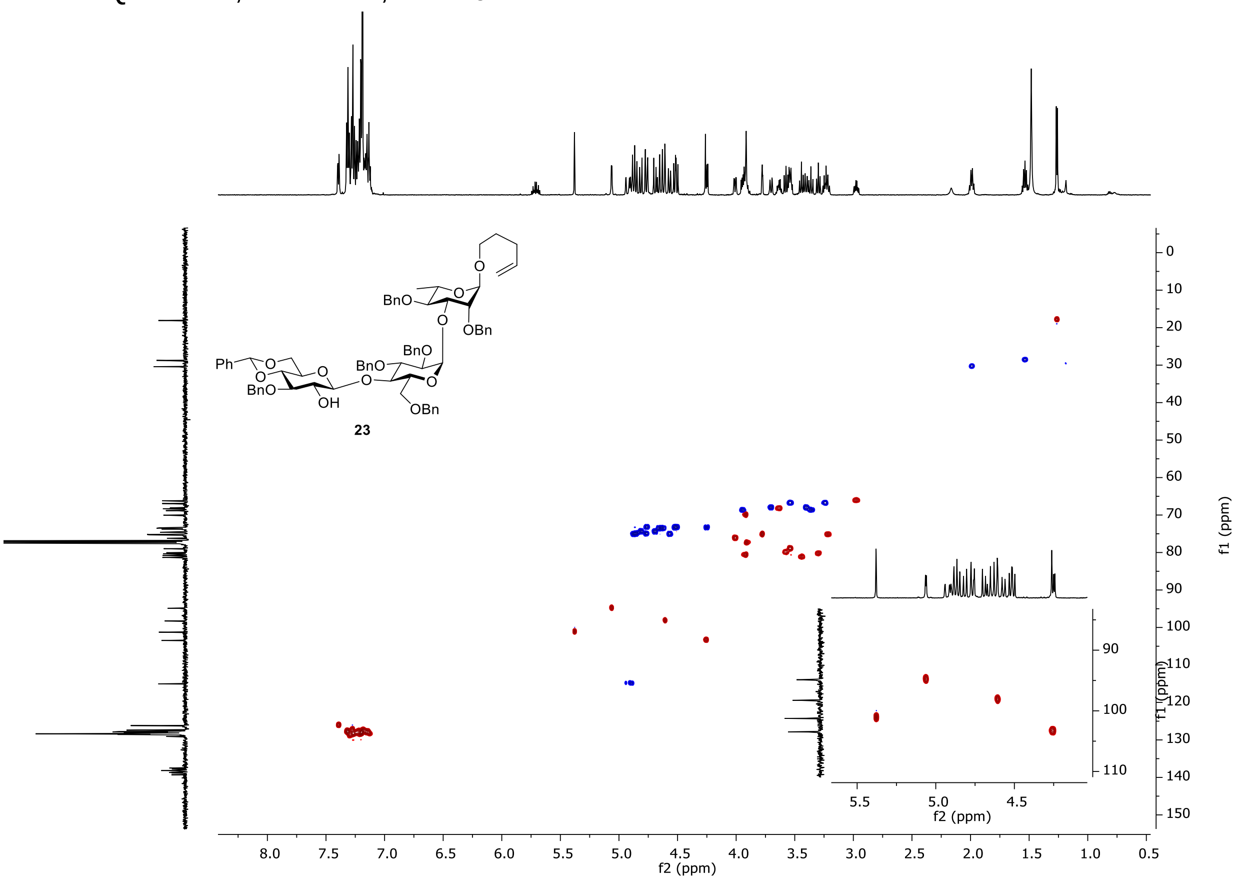

CH-HSQC NMR, 400 MHz, $CDCl_3$

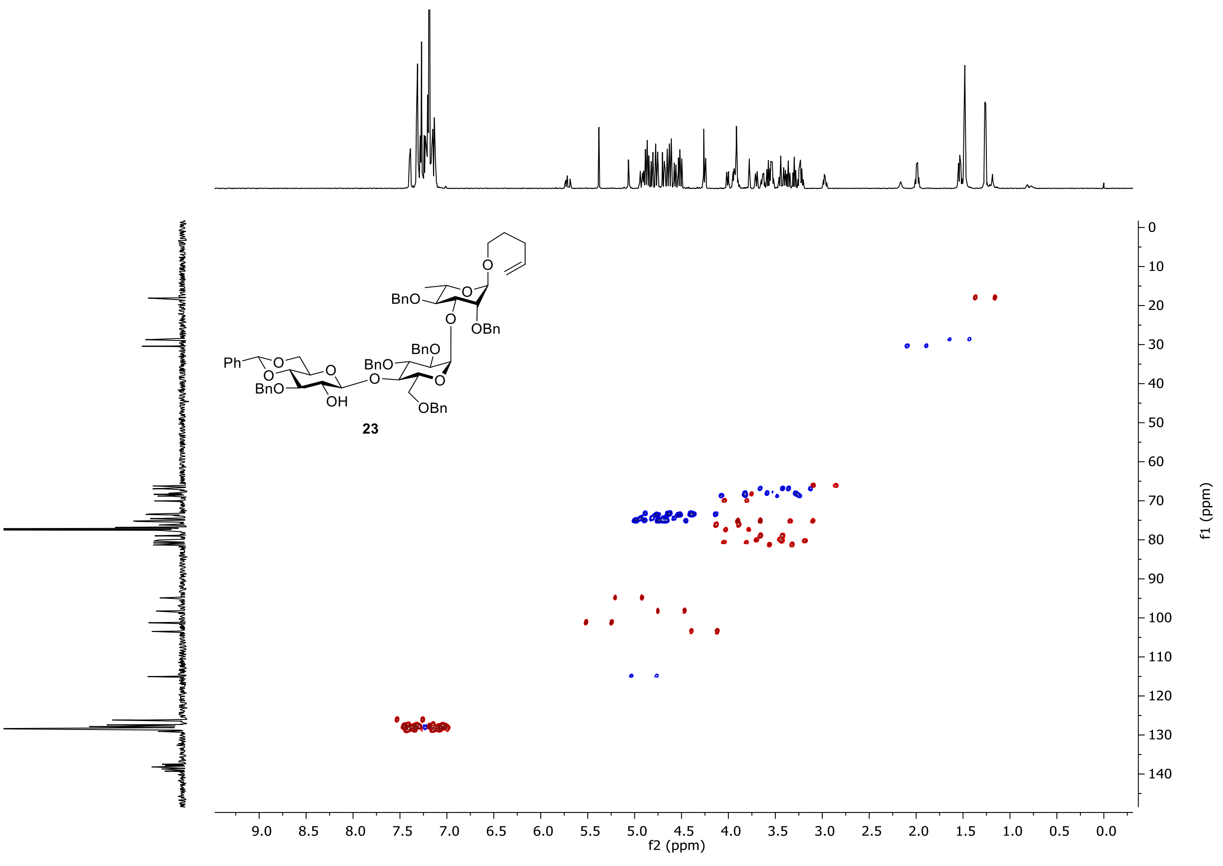

CH-HSQC NMR, 400 MHz, $CDCl_3$ (expansion)

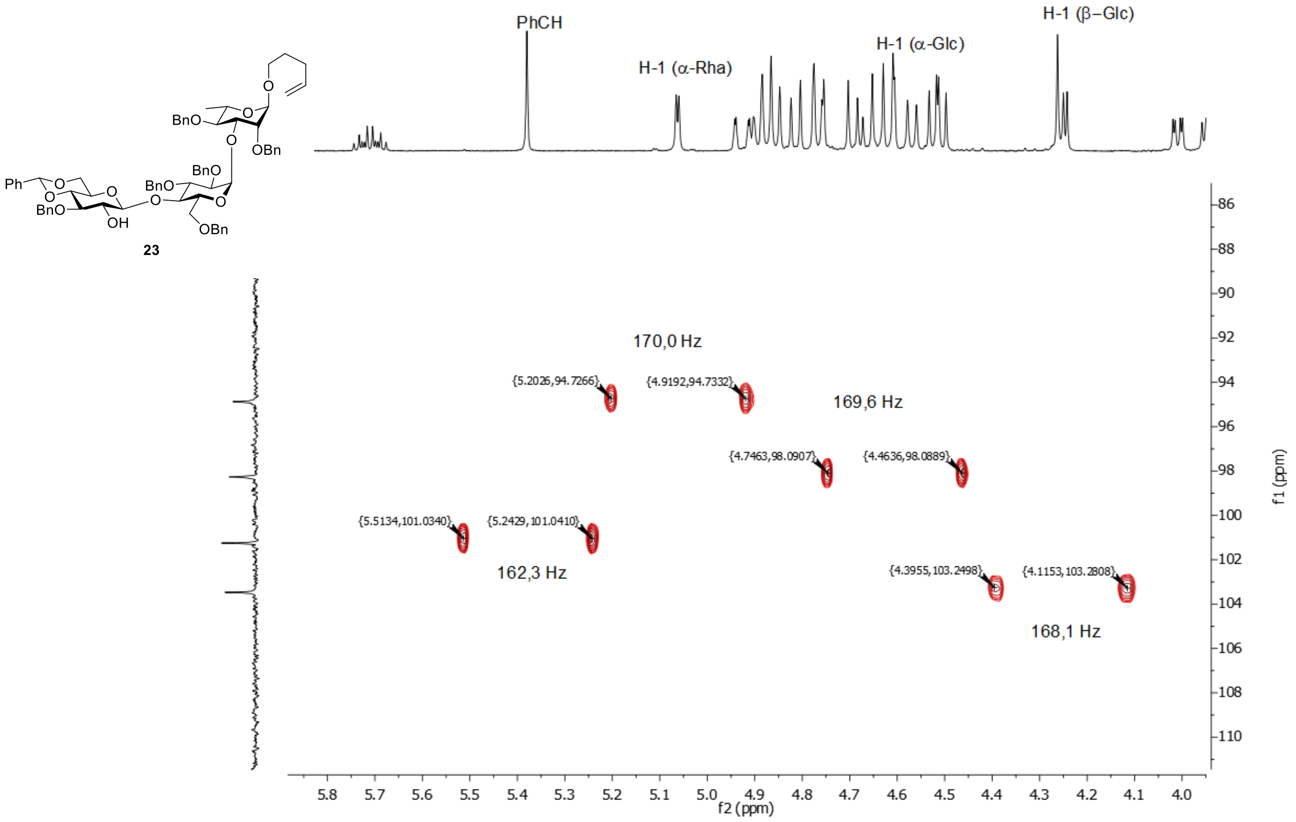

1H NMR, 400 MHz, $CDCl_3$

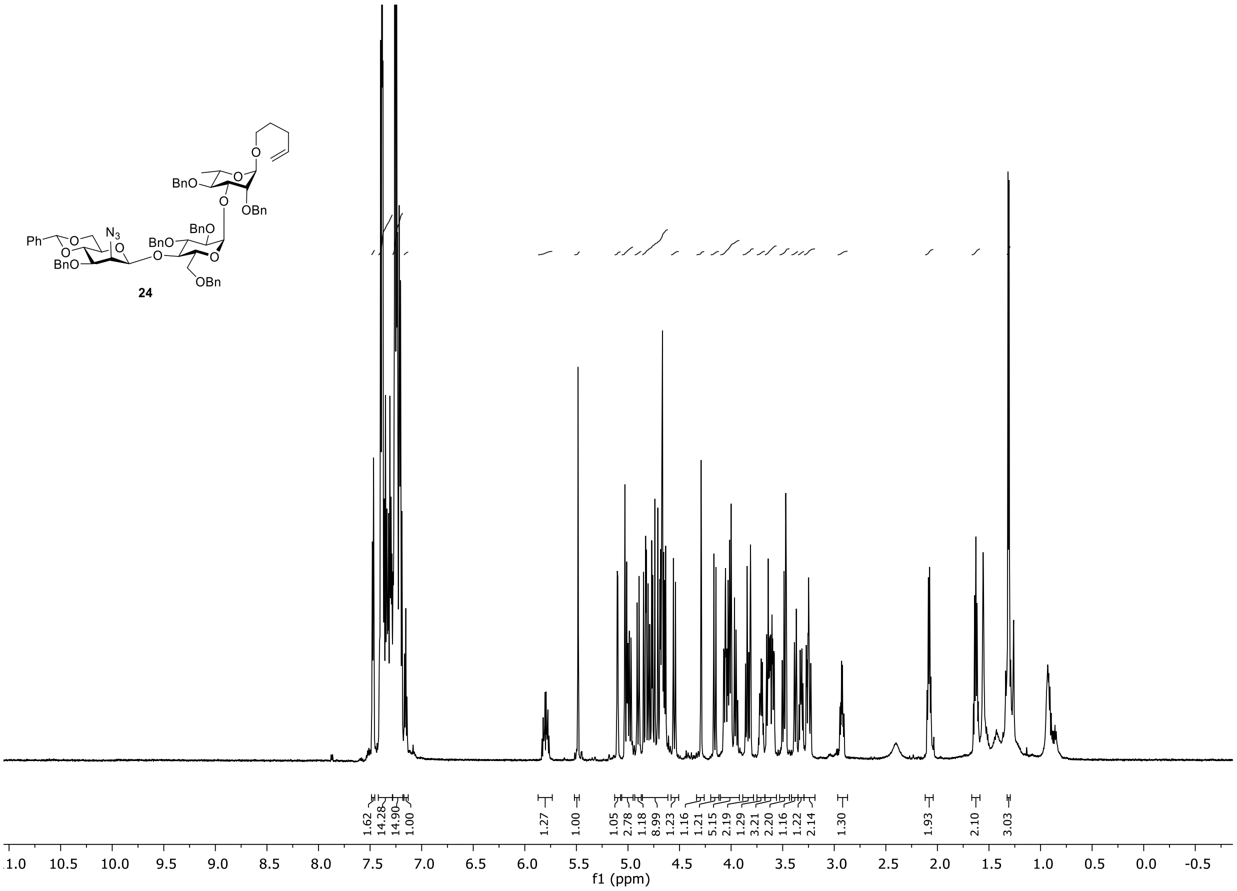

13C NMR, 400 MHz, CDCl3

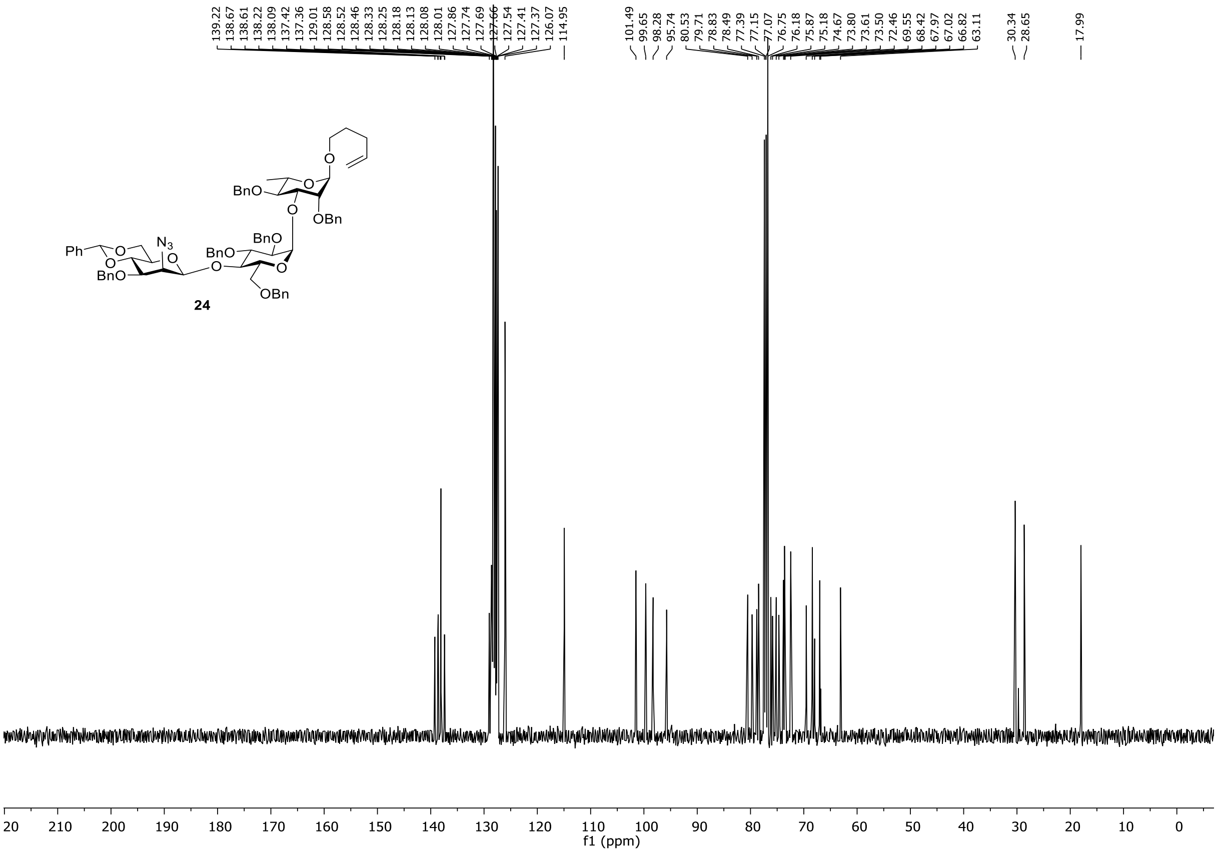

1H-1H COSY NMR, 600 MHz, CDCl3

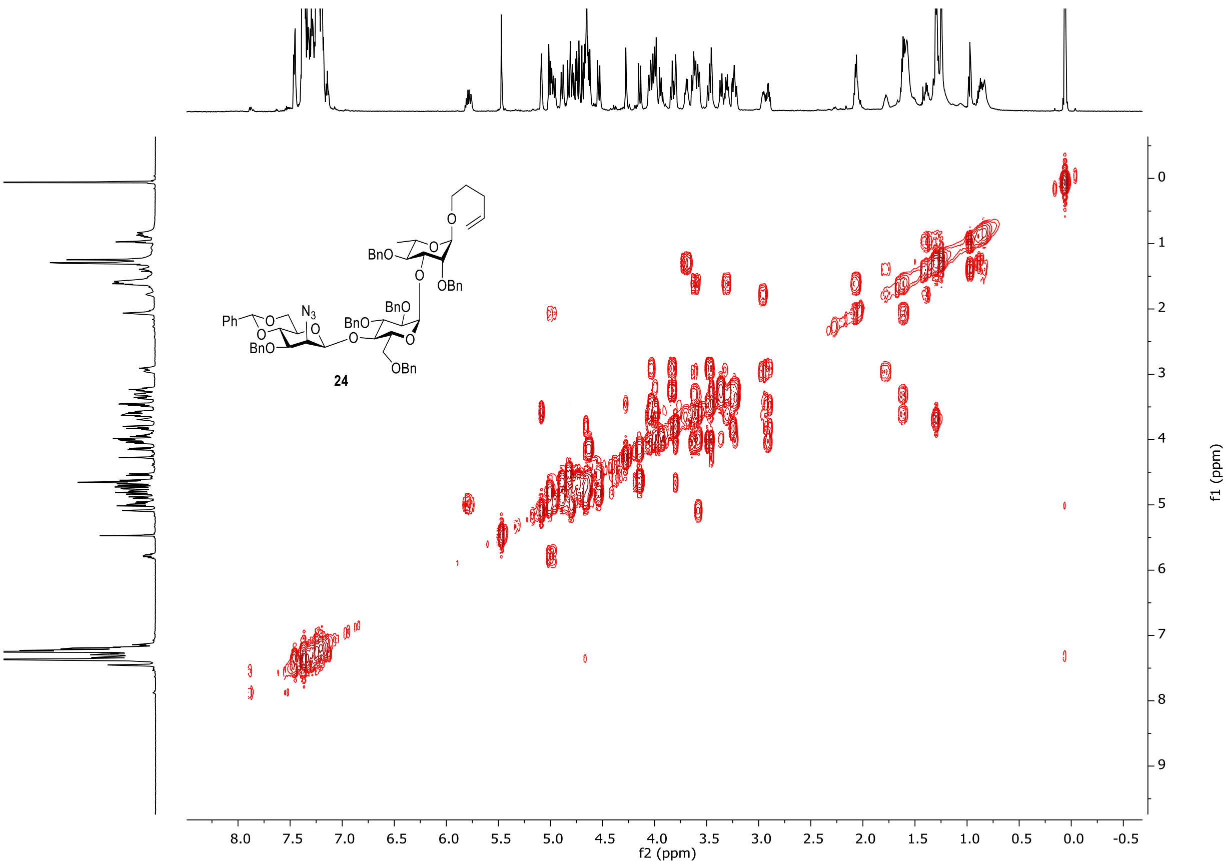

CH-HSQC NMR, 400 MHz, CDCl$_3$

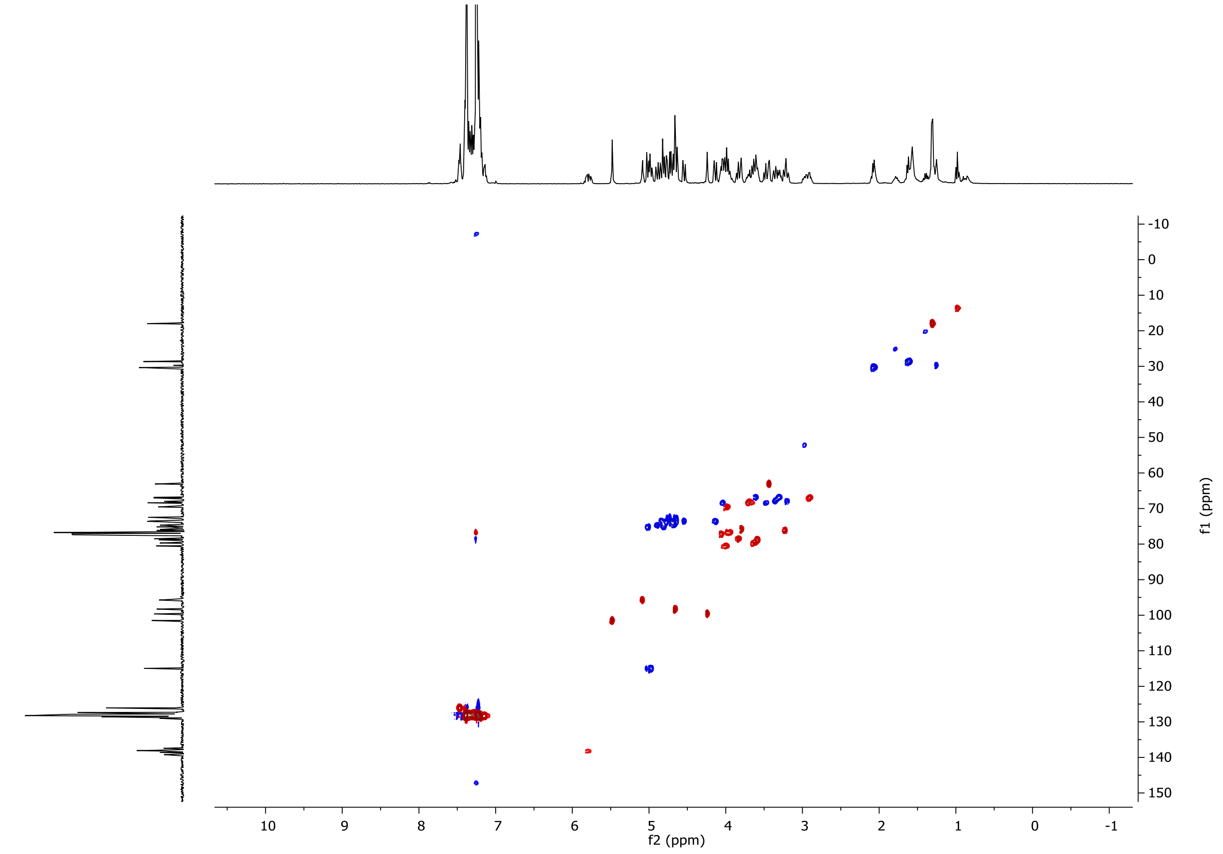

CH-HSQC NMR, 600 MHz, CDCl$_3$

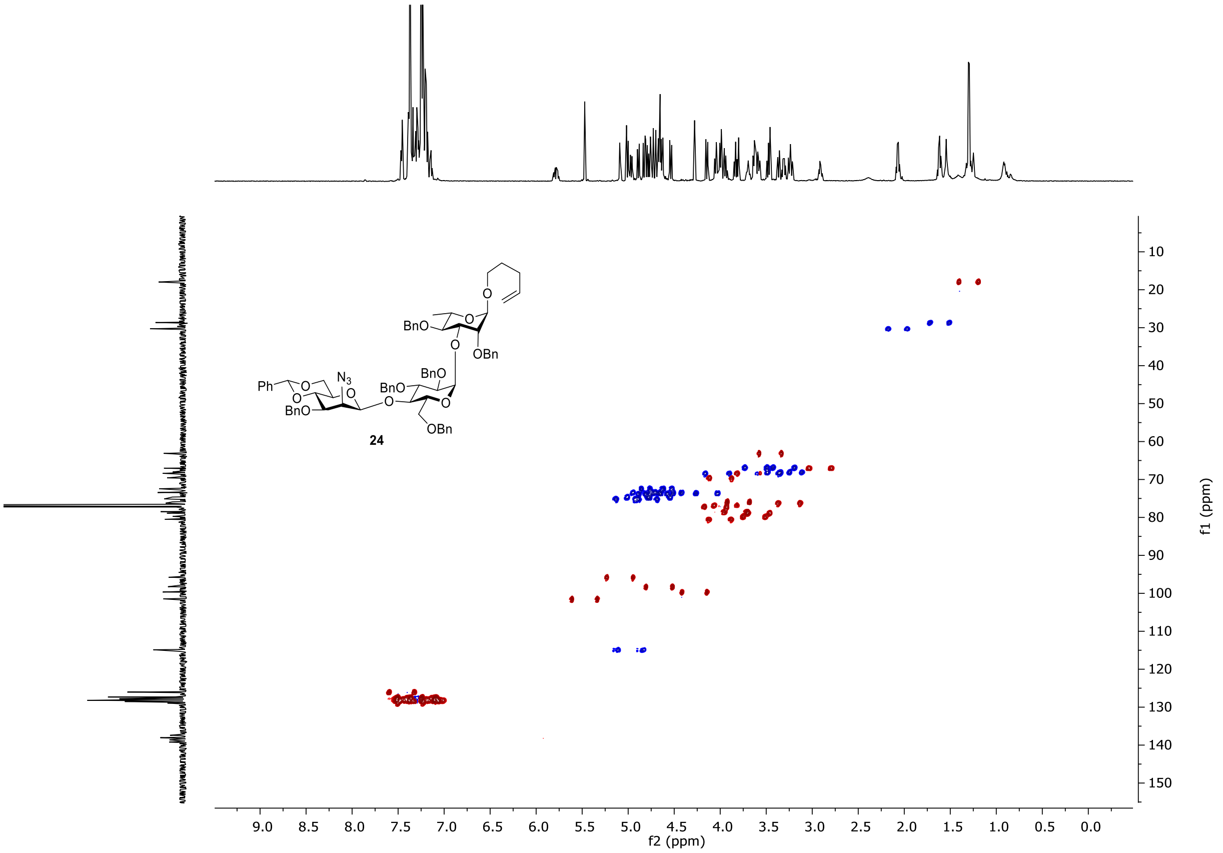

CH-HSQC NMR, 600 MHz, $CDCl_3$ (expansion)

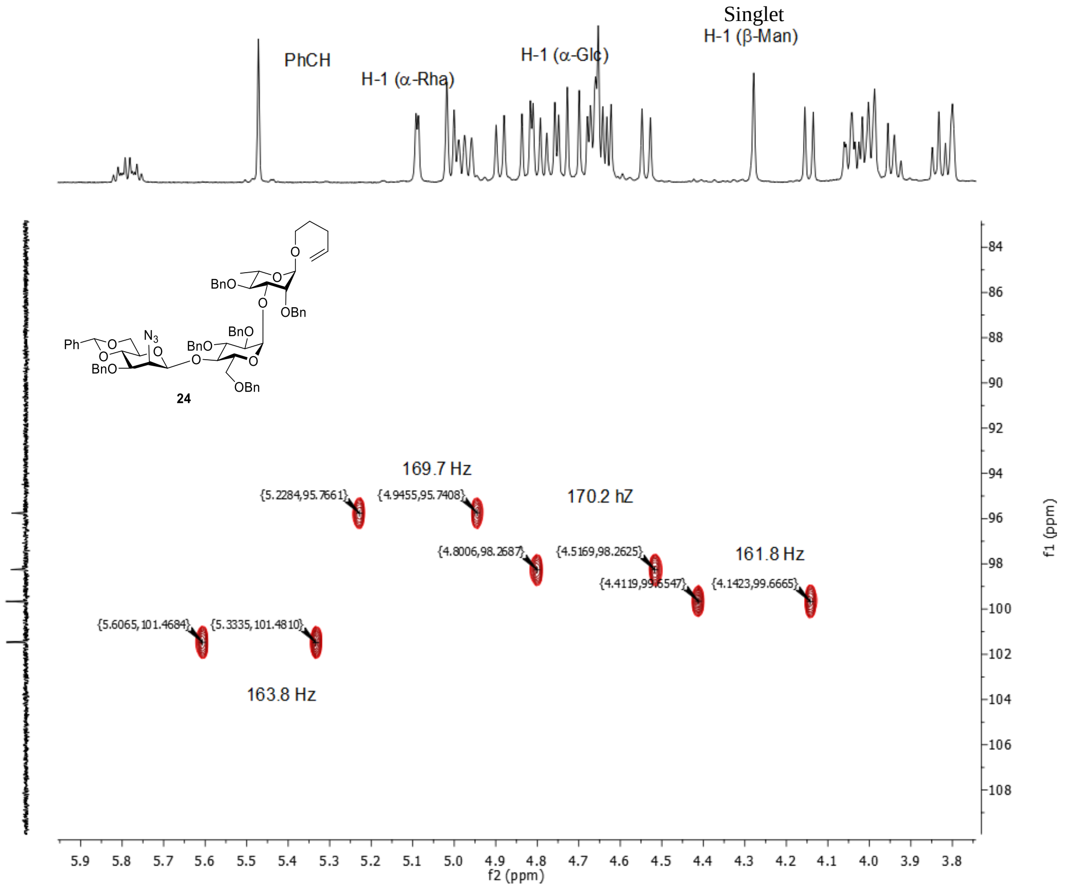

1H NMR, 400 MHz, $CDCl_3$

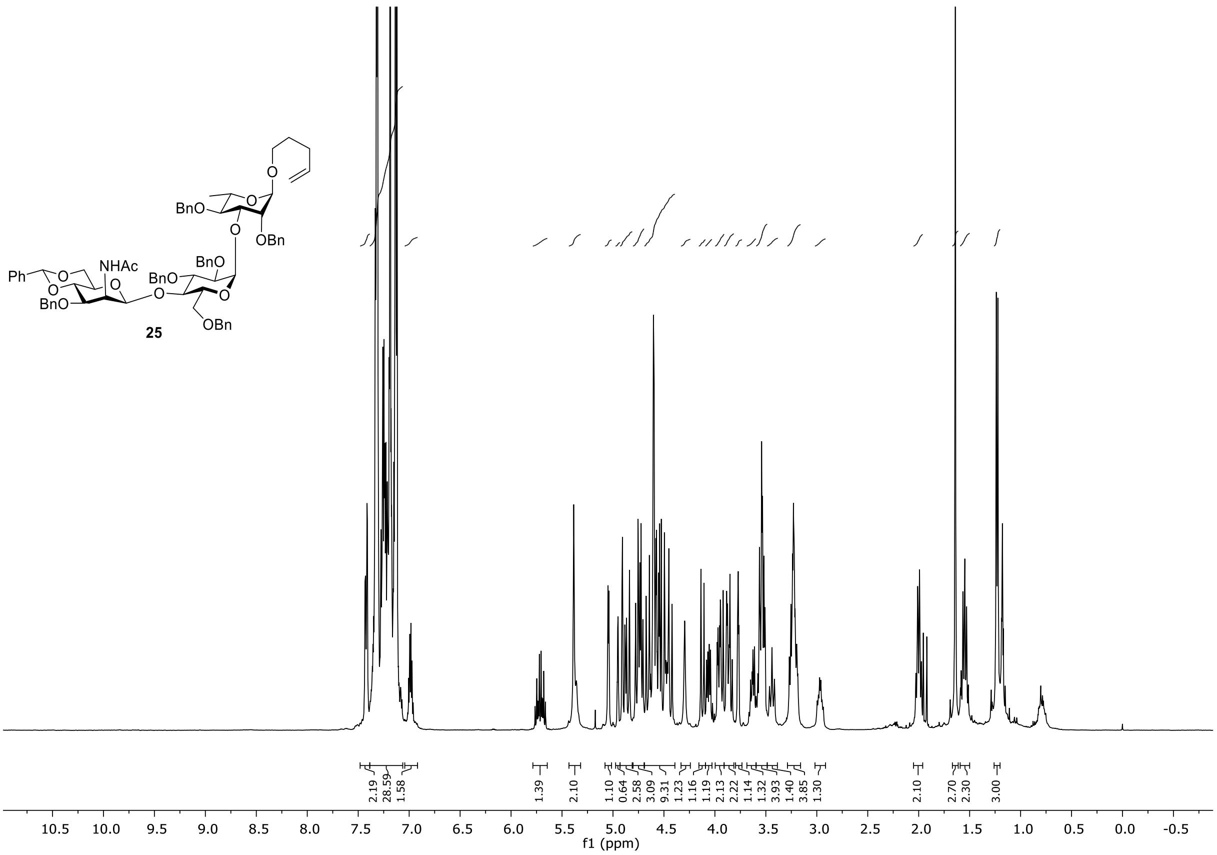

13C NMR, 101 MHz, CDCl3

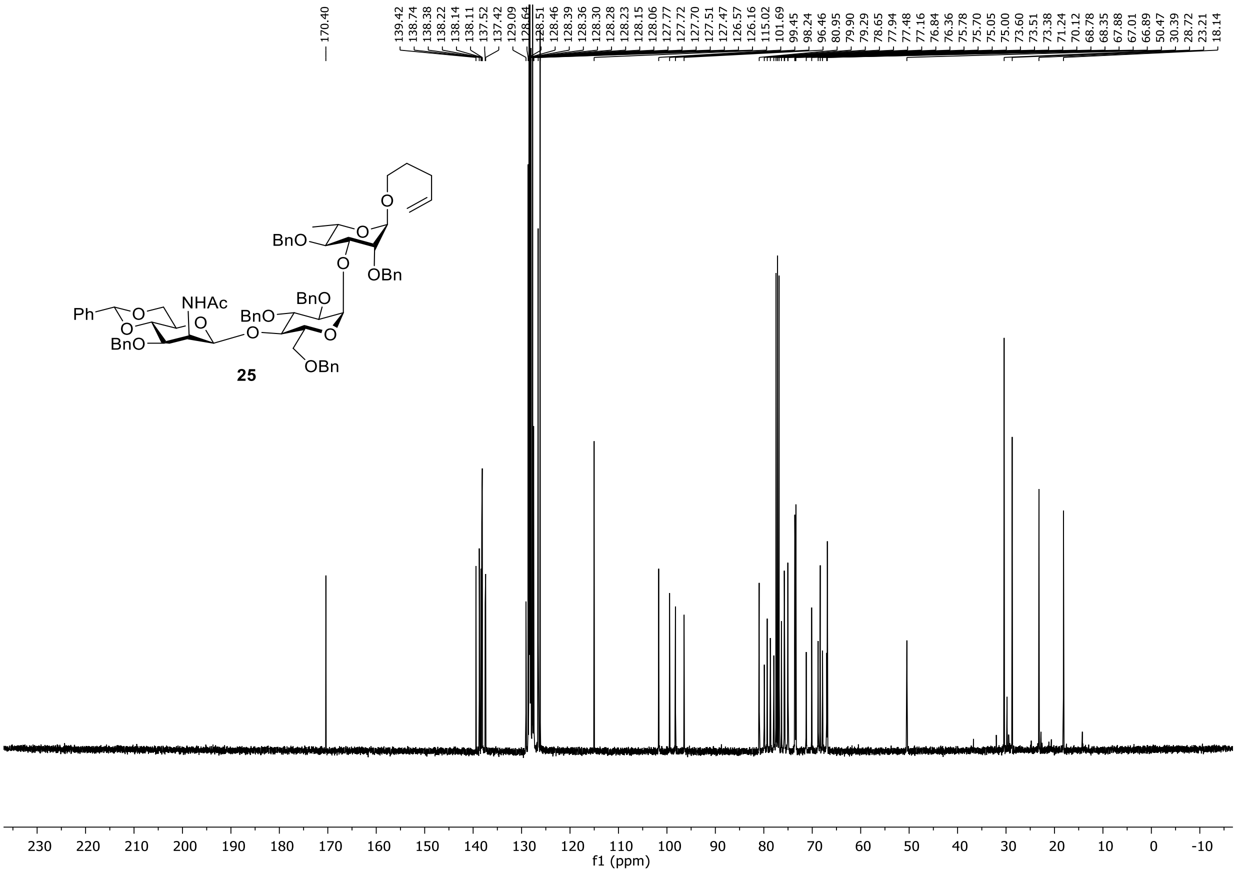

CH-HSQC NMR, 600 MHz, CDCl3

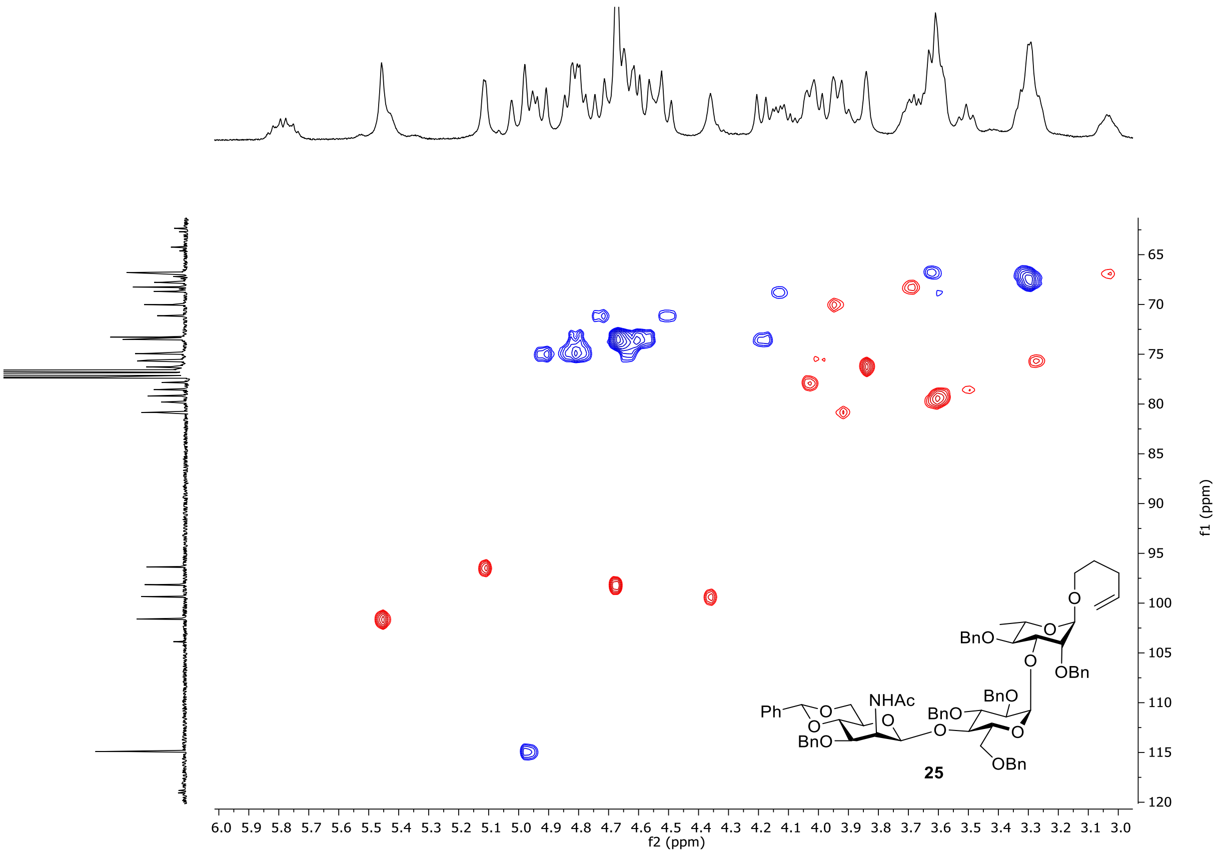

1H NMR, 400 MHz, CDCl3

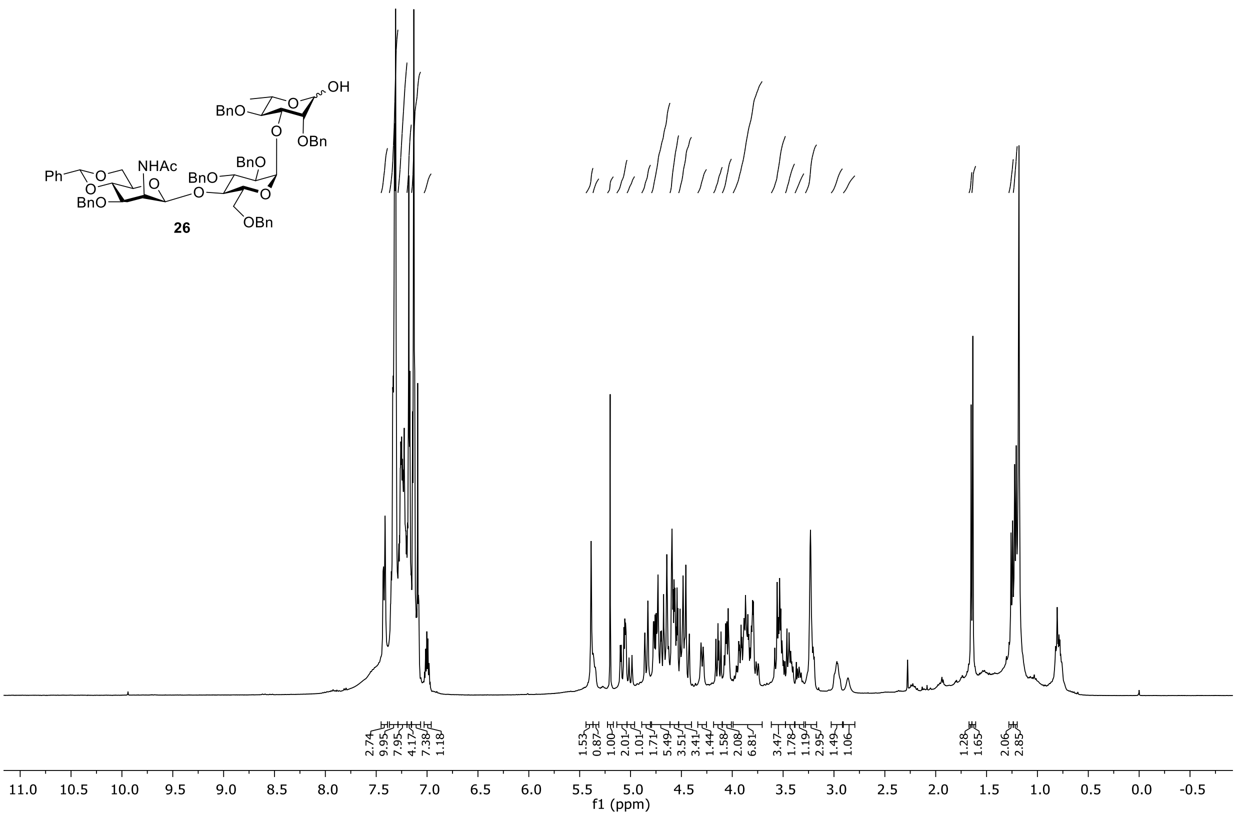

13C NMR, 400 MHz, CDCl3

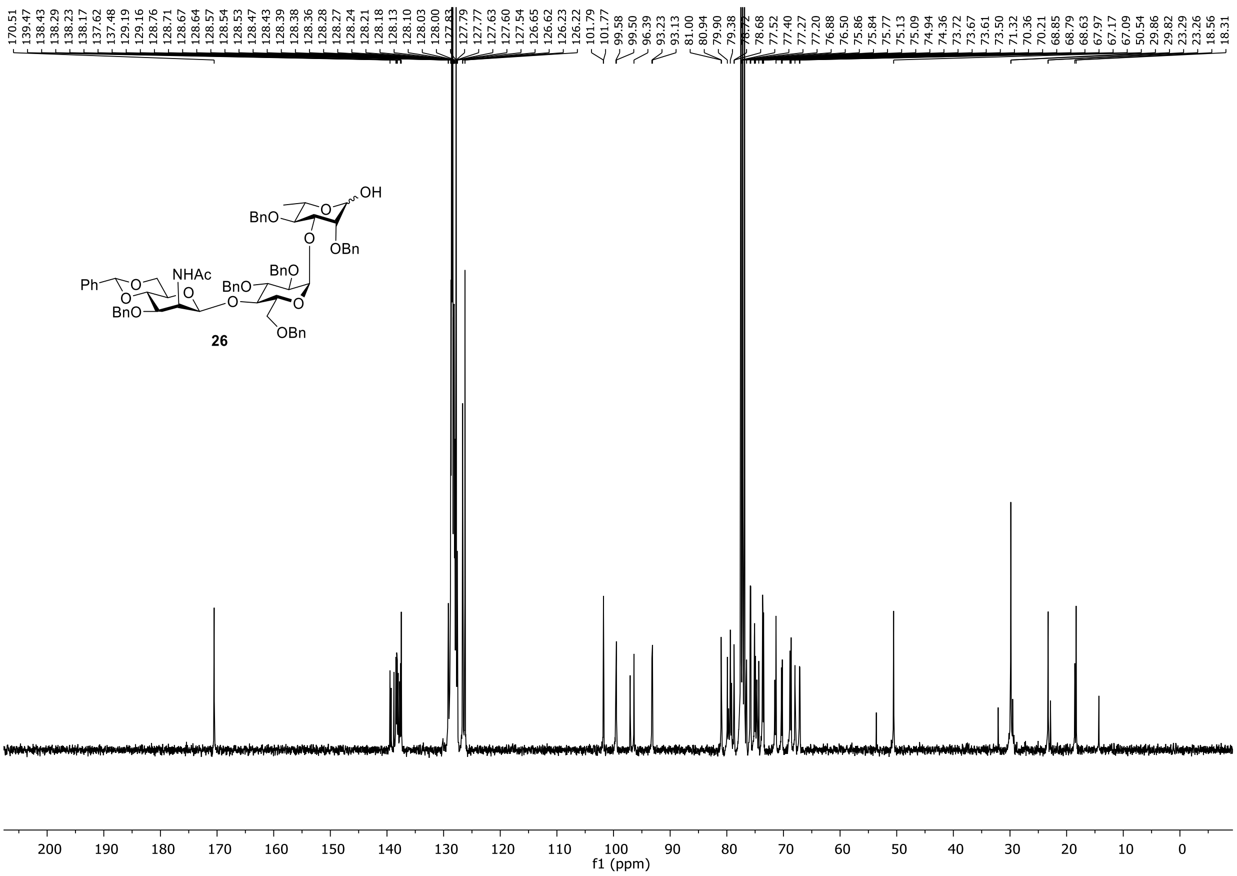

CH-HSQC NMR, 400 MHz, $CDCl_3$

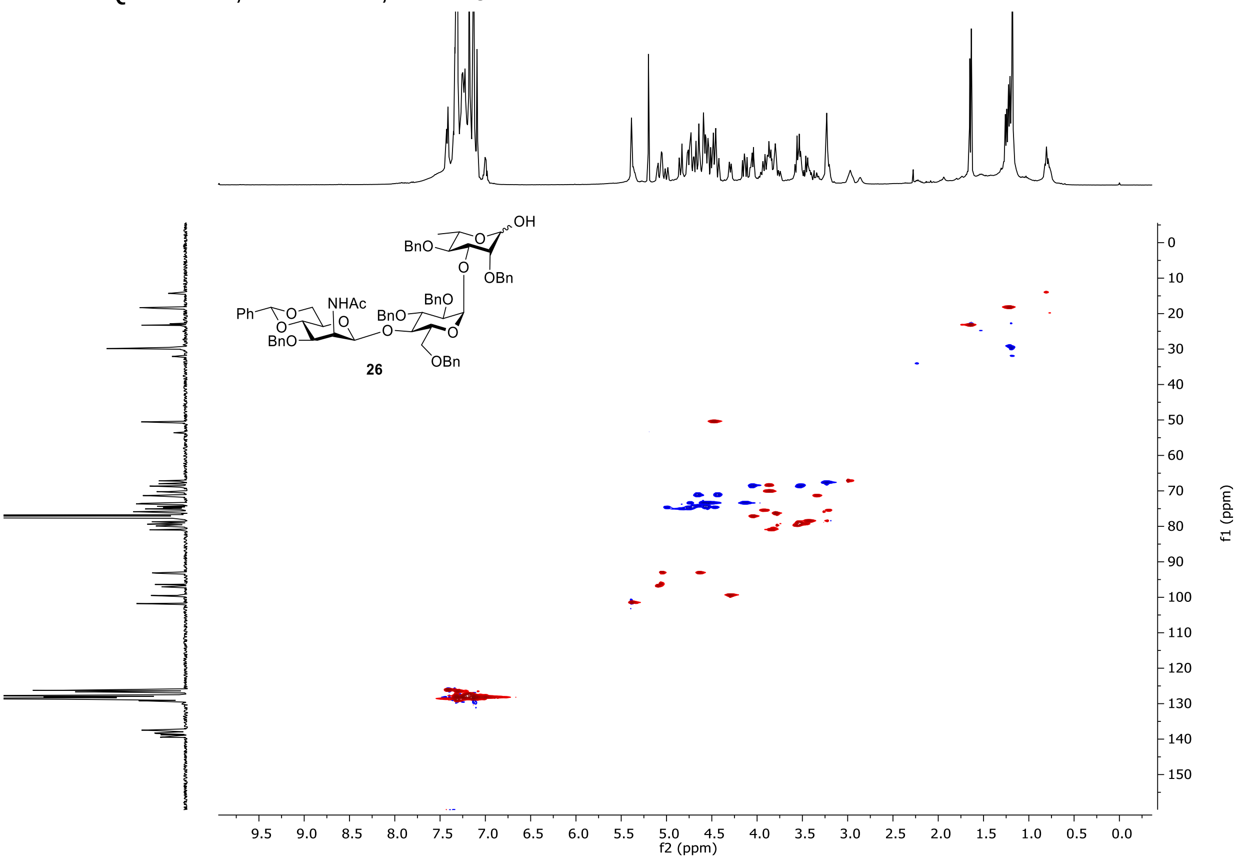

1H NMR, 400 MHz, $CDCl_3$

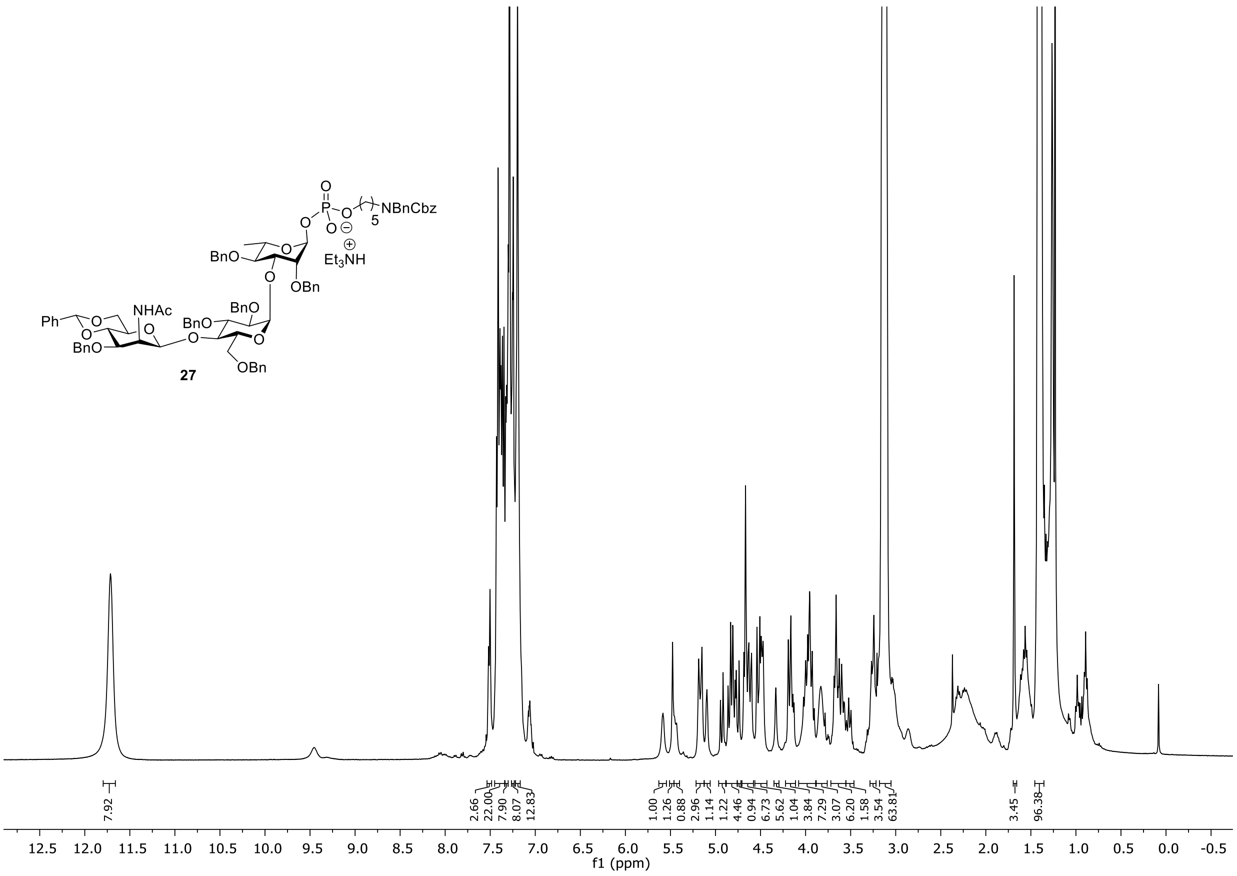

31P NMR, 162 MHz, $CDCl_3$

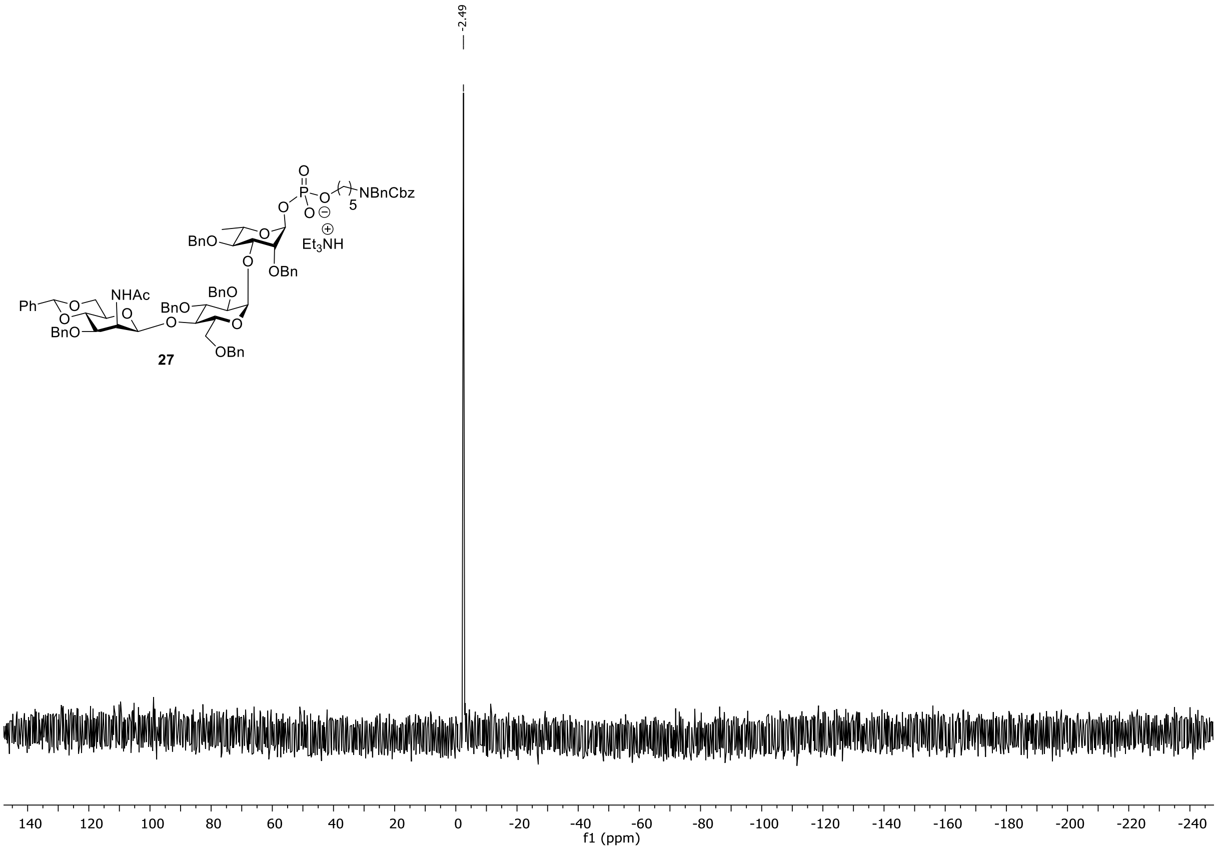

13C NMR, 151 MHz, $CDCl_3$

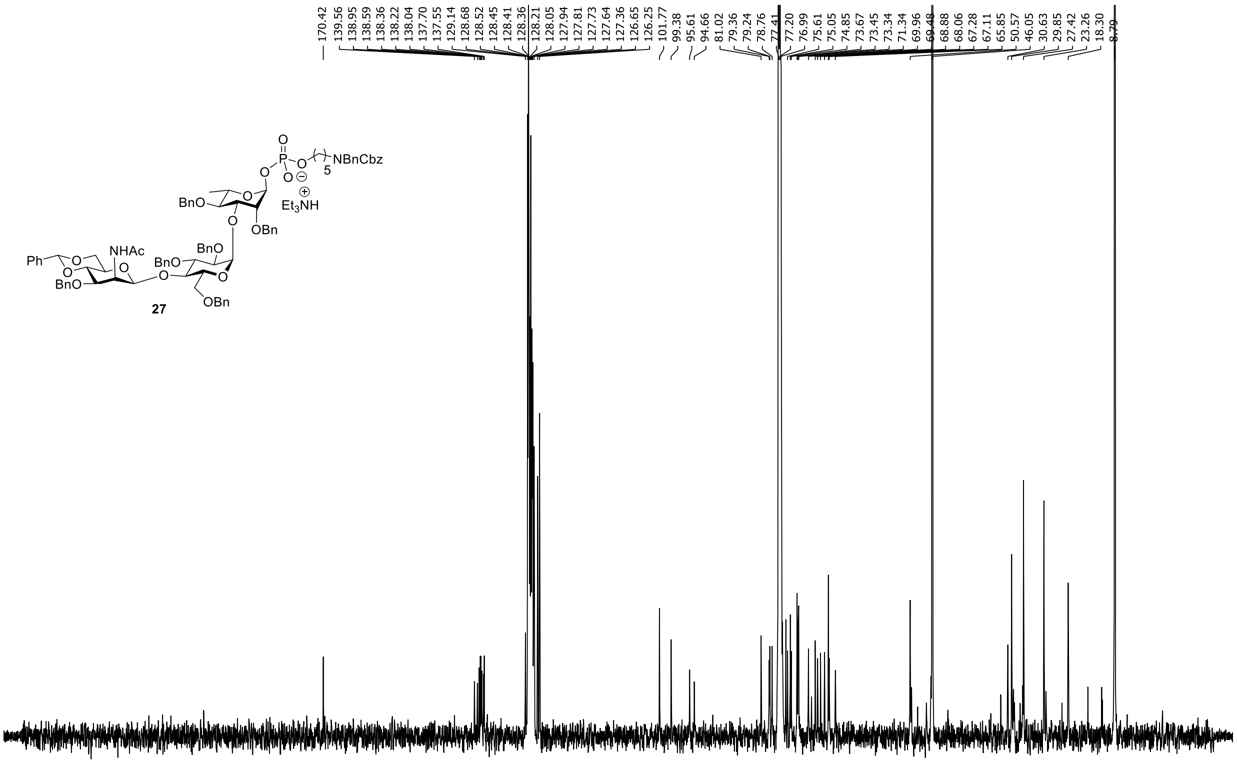

CH-HSQC NMR, 400 MHz, $CDCl_3$

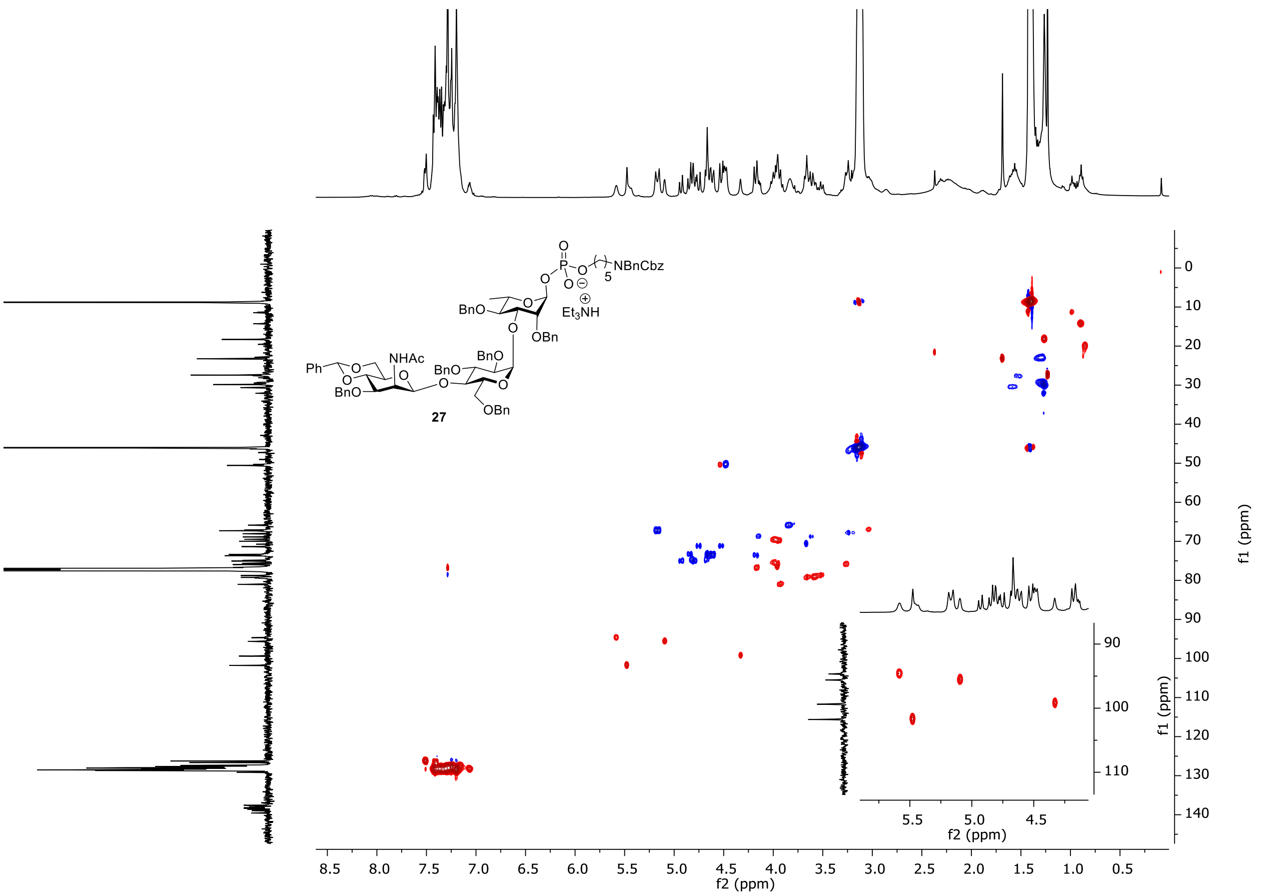

CH-HSQC NMR, 400 MHz, $CDCl_3$

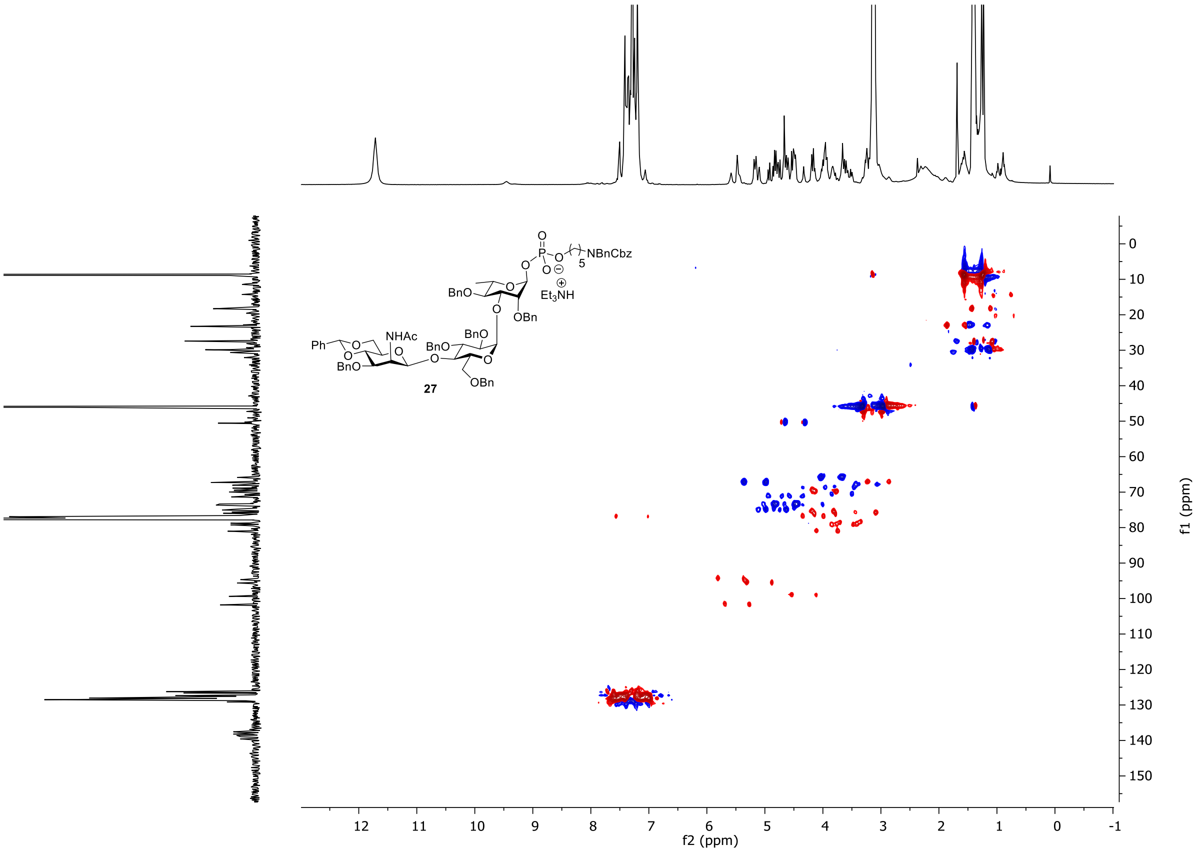

CH-HSQC NMR, 400 MHz, $CDCl_3$

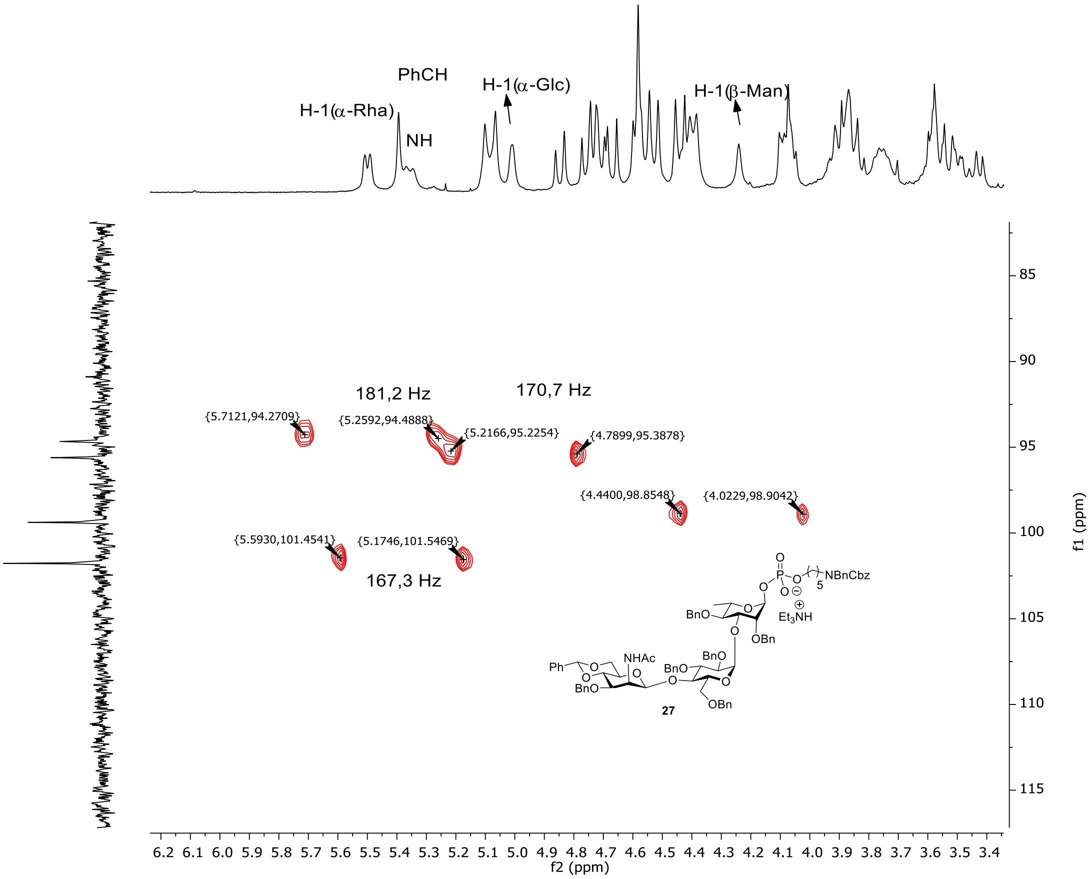

1H NMR, 600 MHz, D_2O

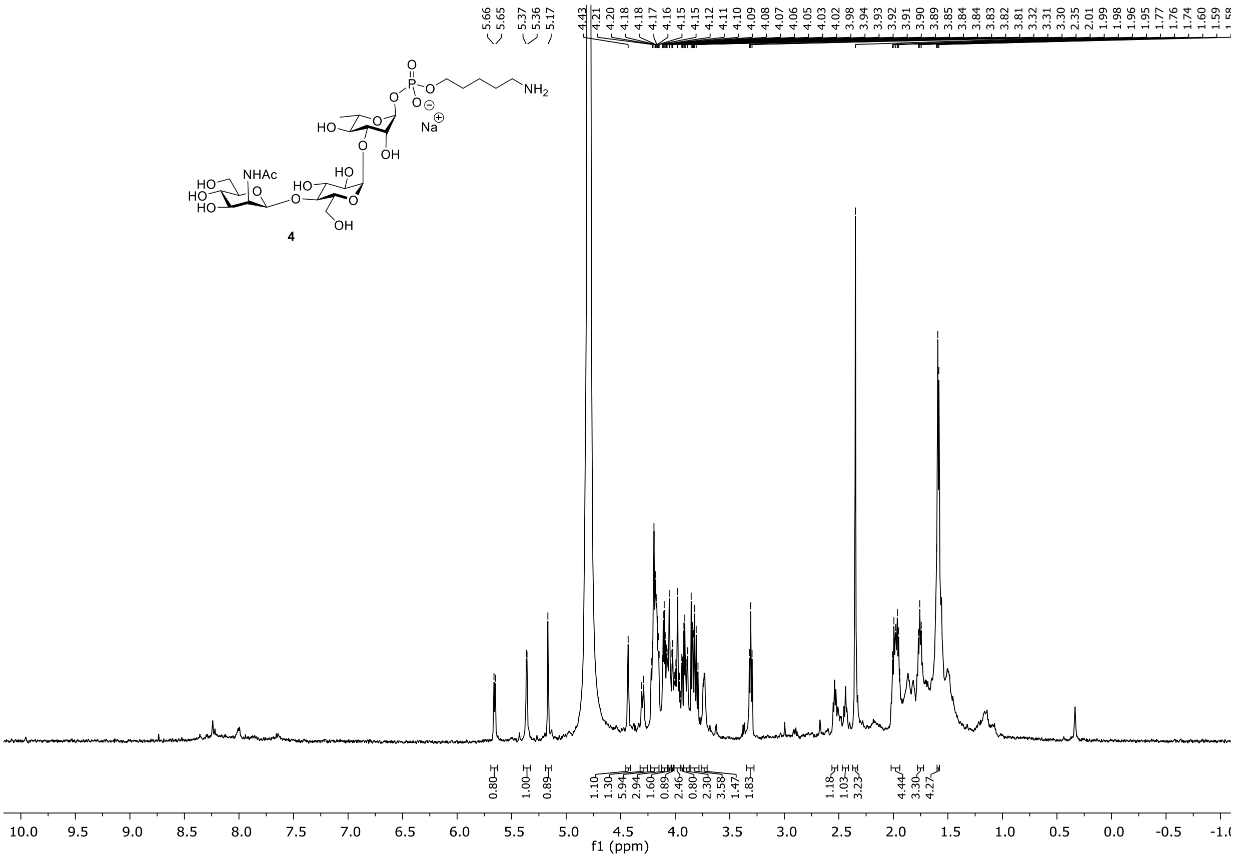

31P NMR, 243 MHz, D2O

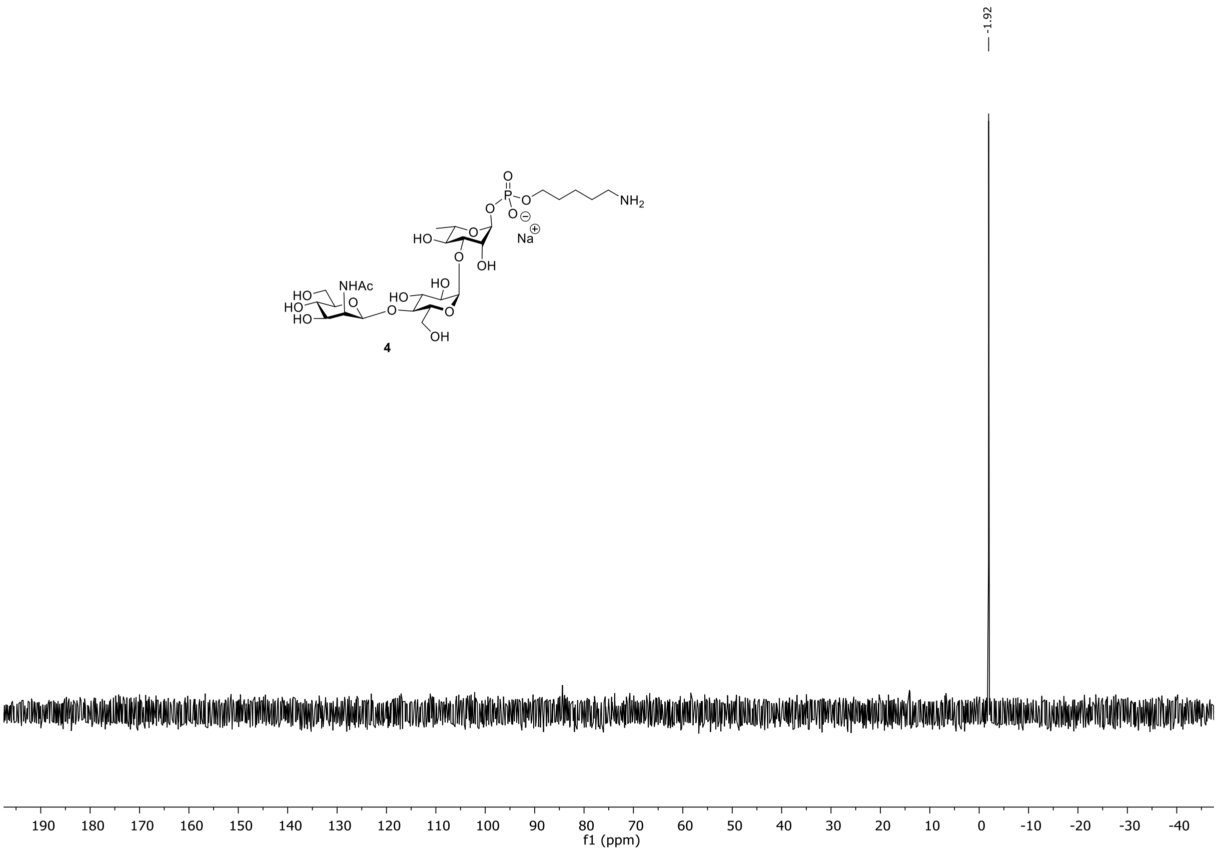

13C NMR, 176 MHz, D2O

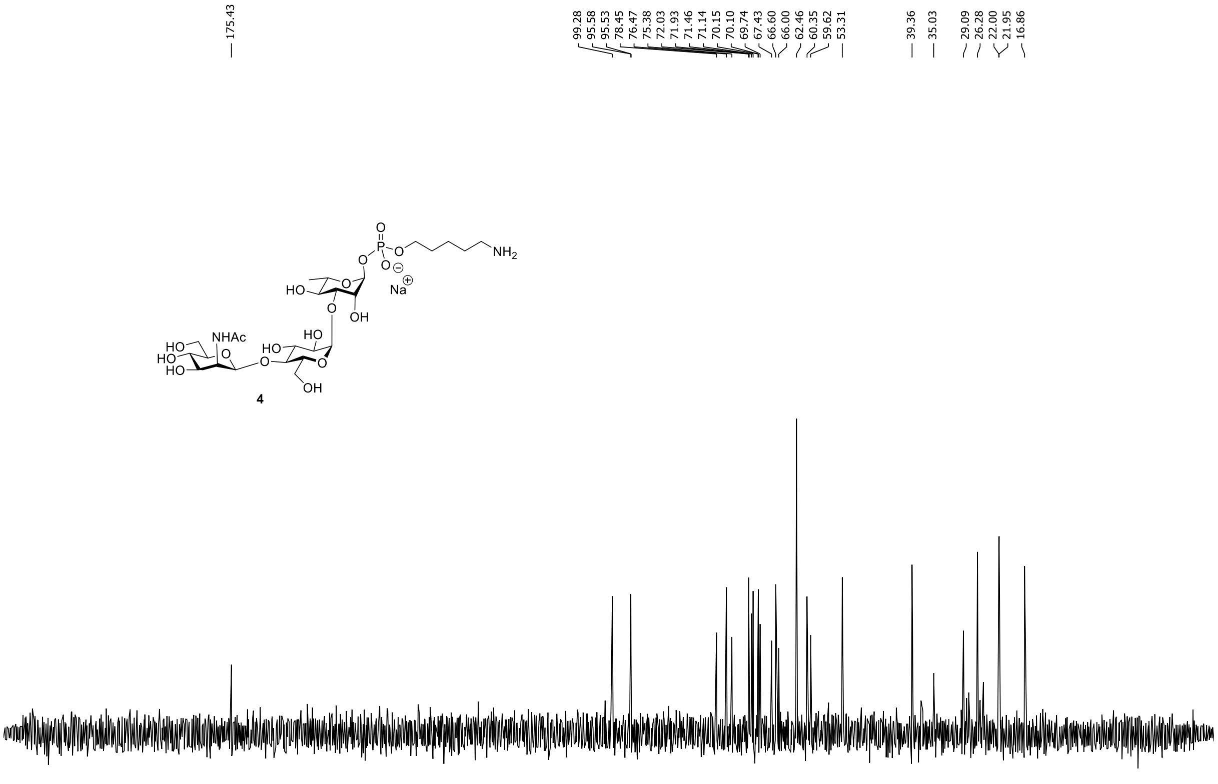

CH-HSQC NMR, 600 MHz, D_2O

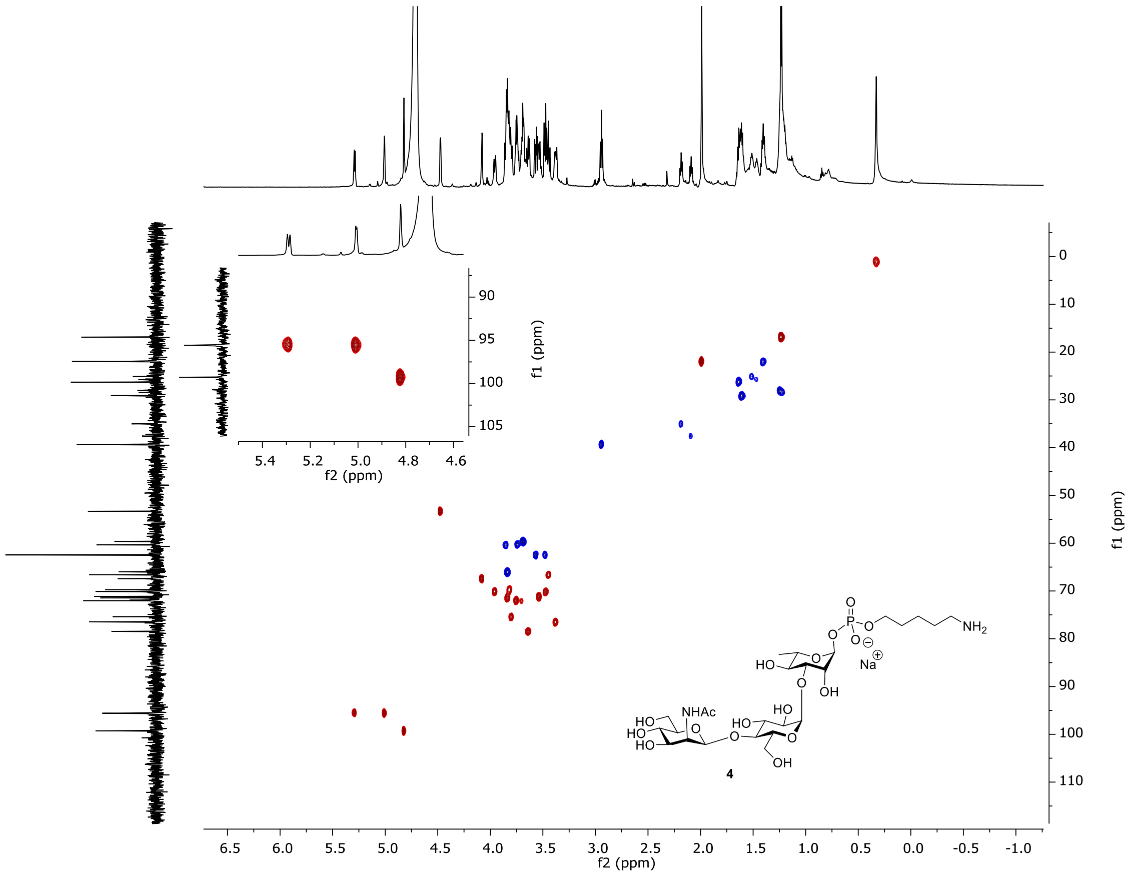

CH-HSQC NMR, 600 MHz, D_2O

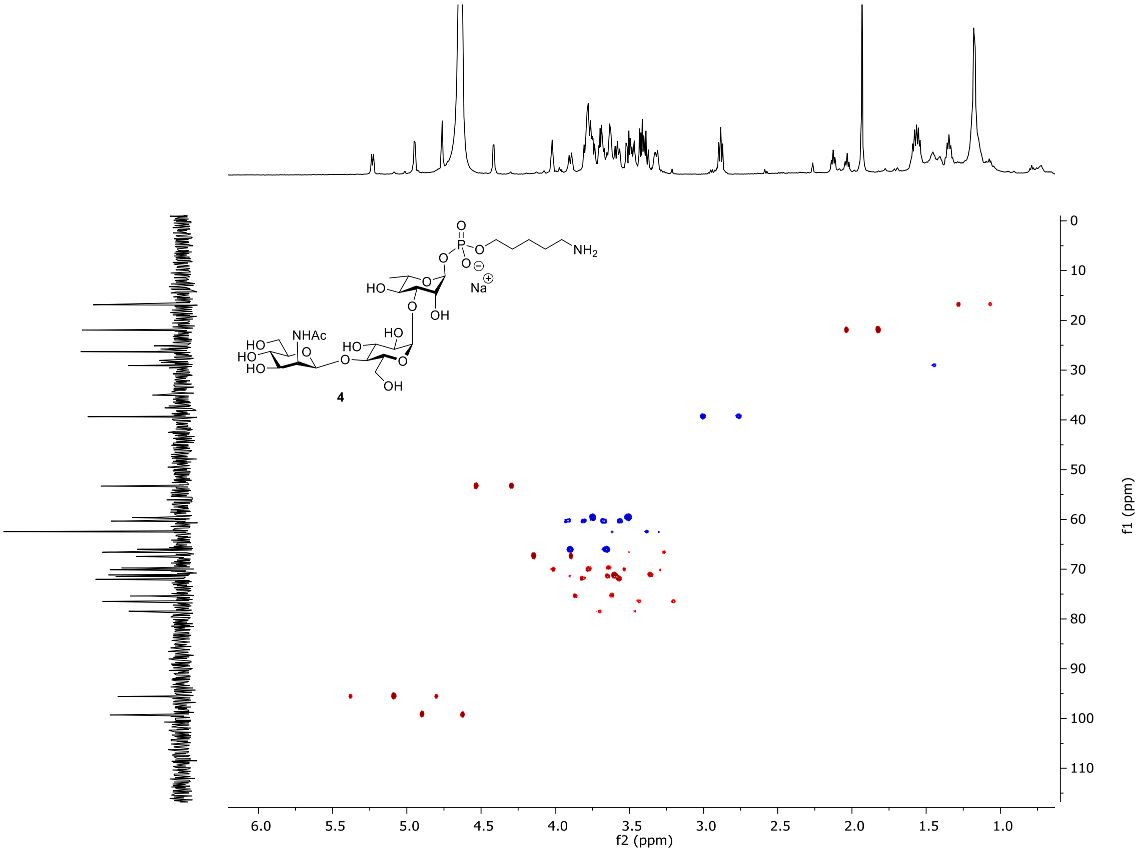

CH-HSQC NMR, 600 MHz, D2O (Expansion)

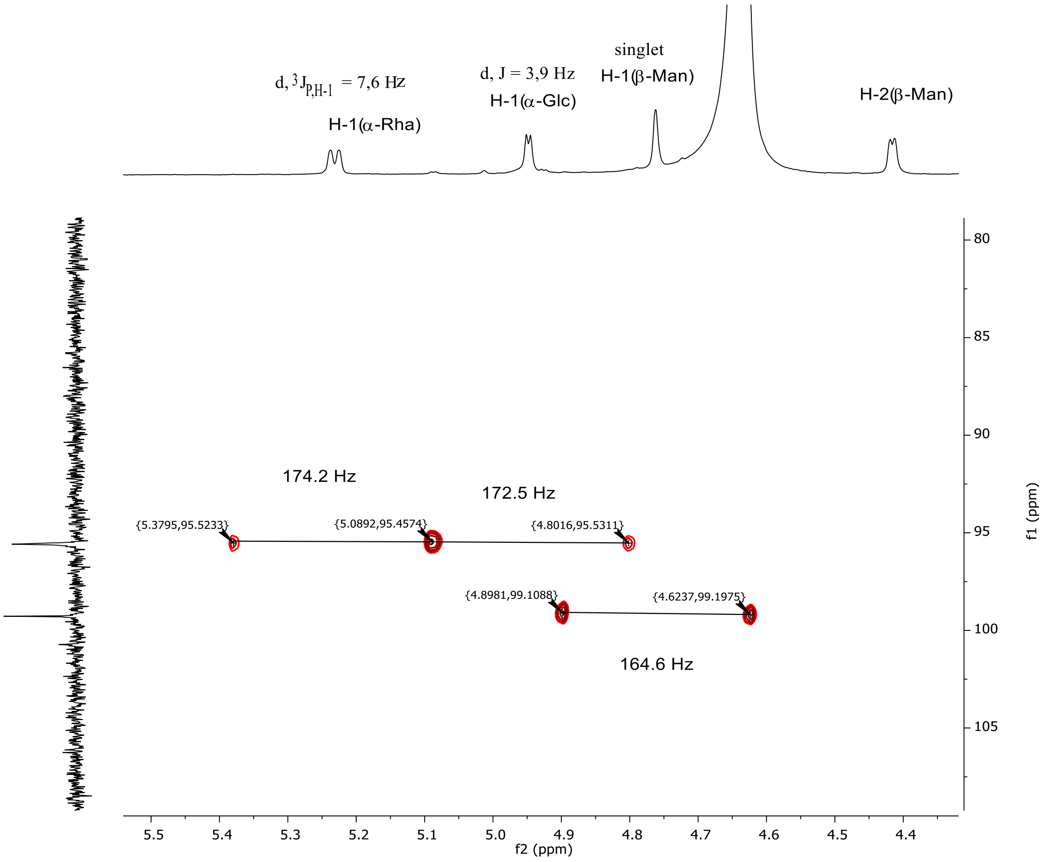

HRMS (Q-Tof)

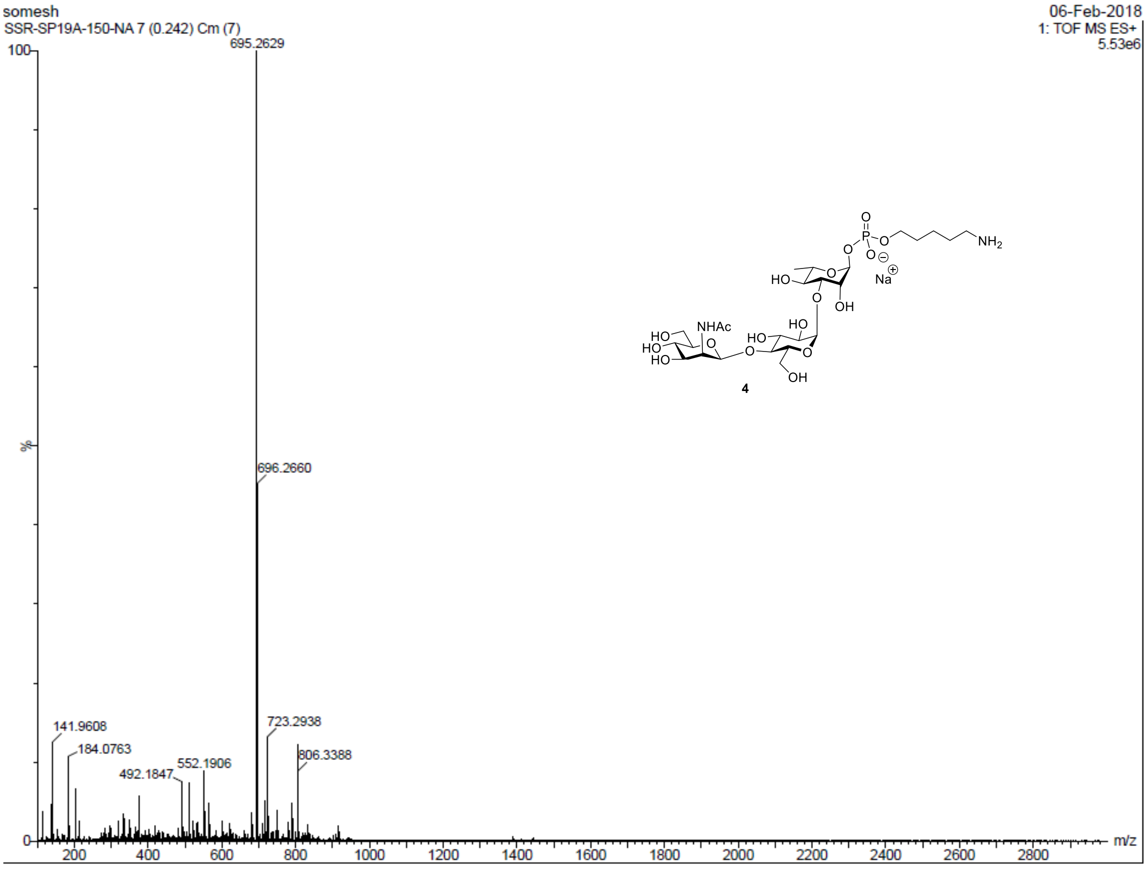

4

1H NMR, 700 MHz, $CDCl_3$

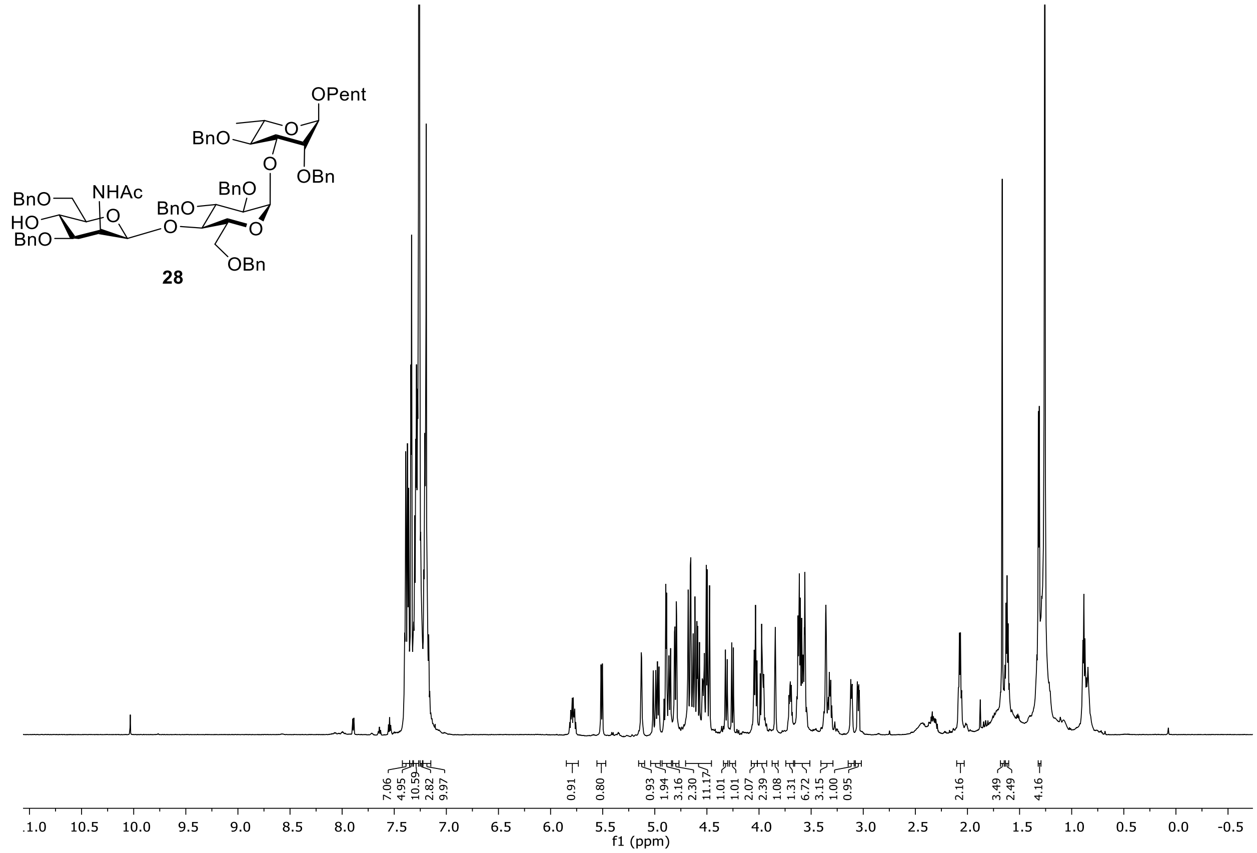

13C NMR, 176 MHz, $CDCl_3$

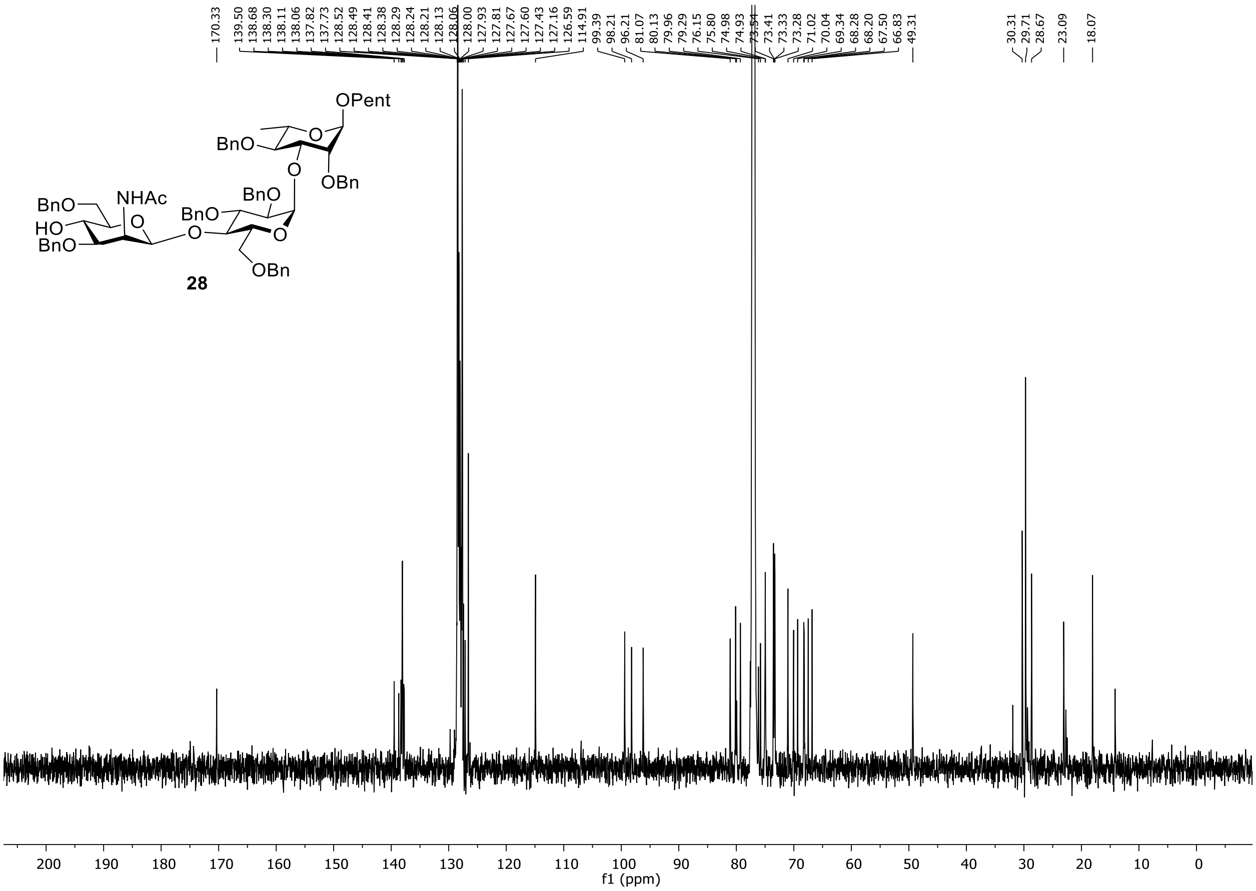

CH-HSQC NMR, 700 MHz, $CDCl_3$

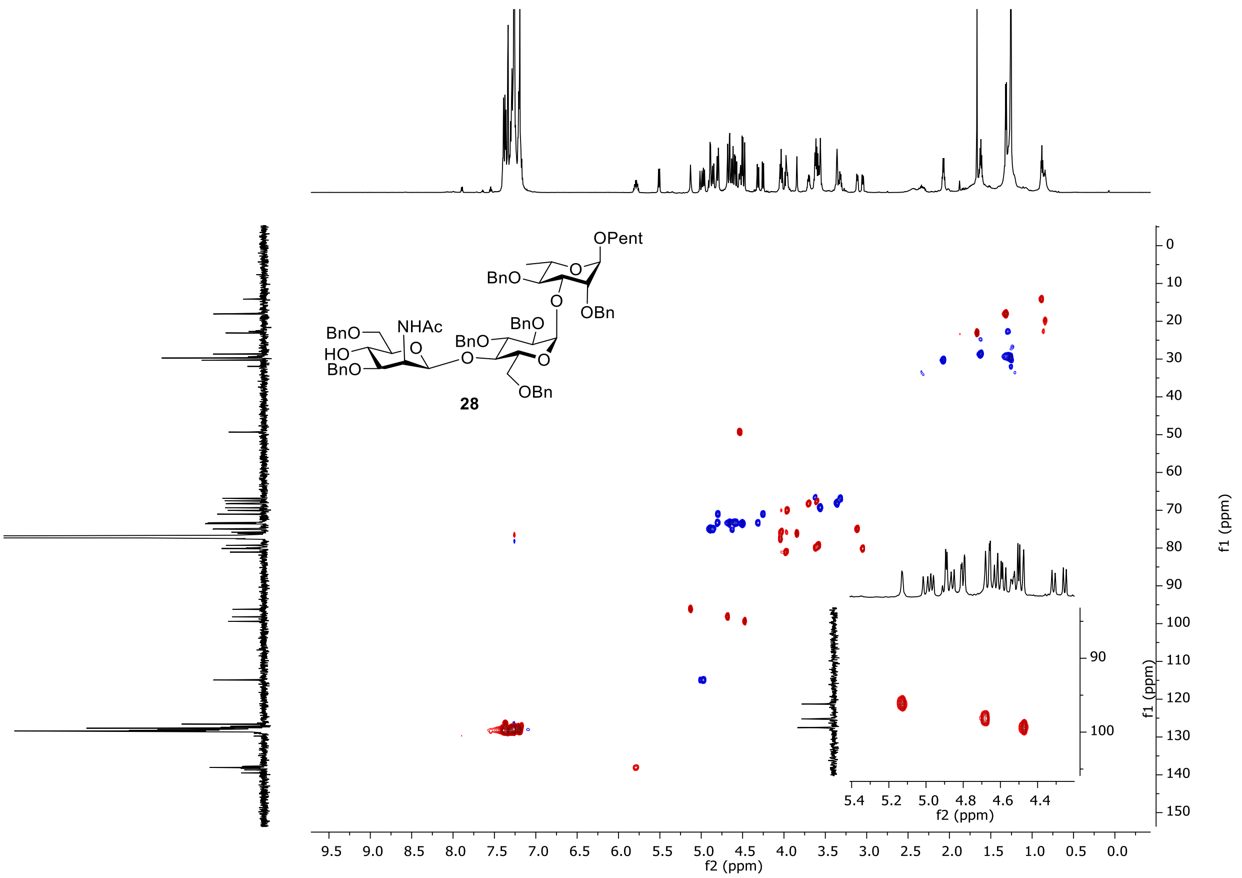

CH-HSQC NMR, 400 MHz, $CDCl_3$

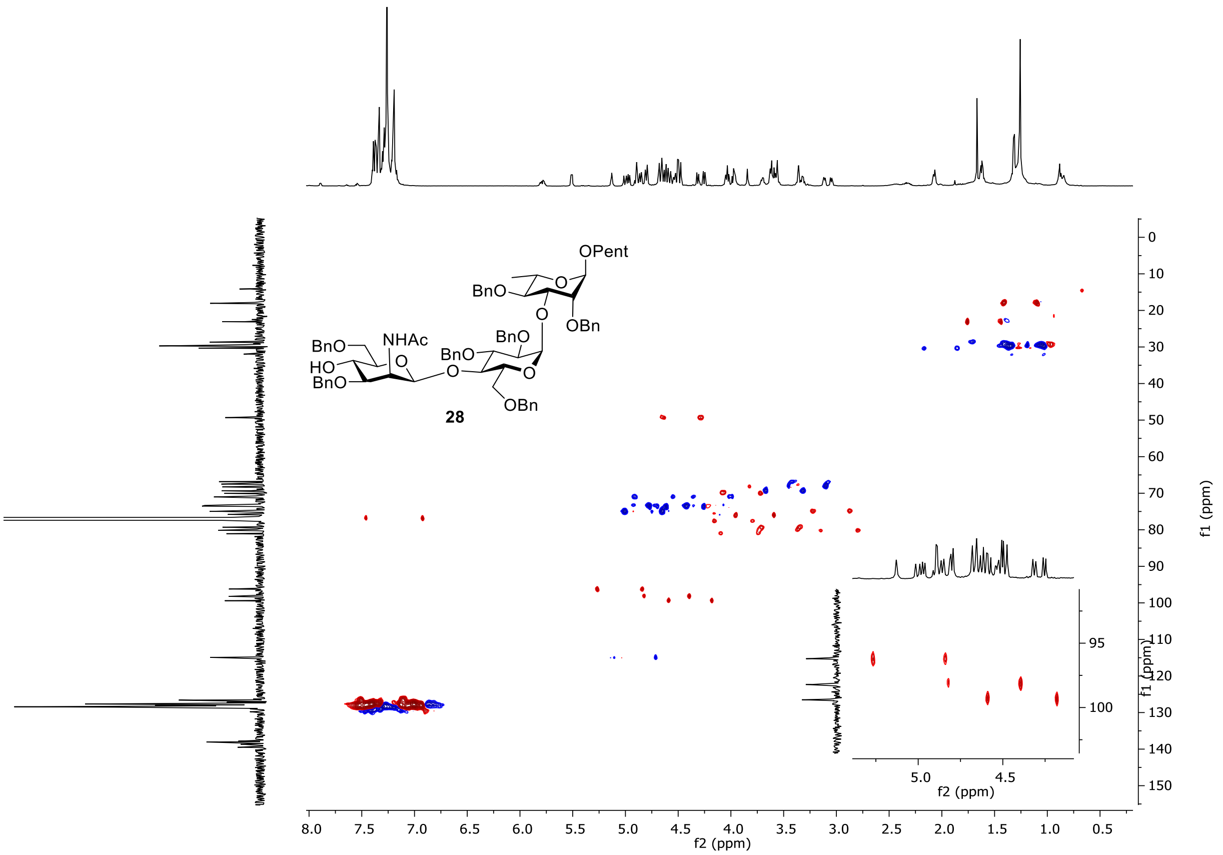

1H NMR, 600 MHz, $CDCl_3$

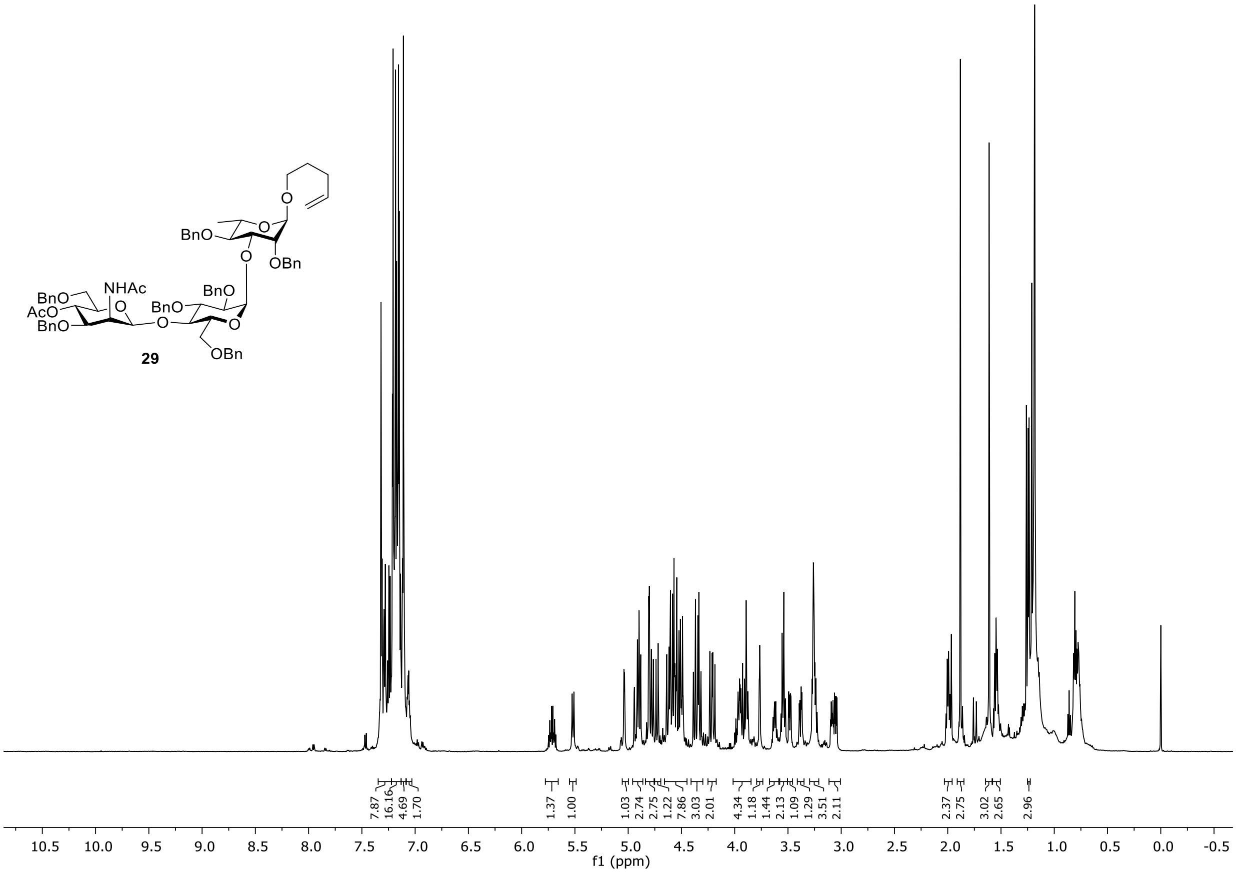

13C NMR, 151 MHz, $CDCl_3$

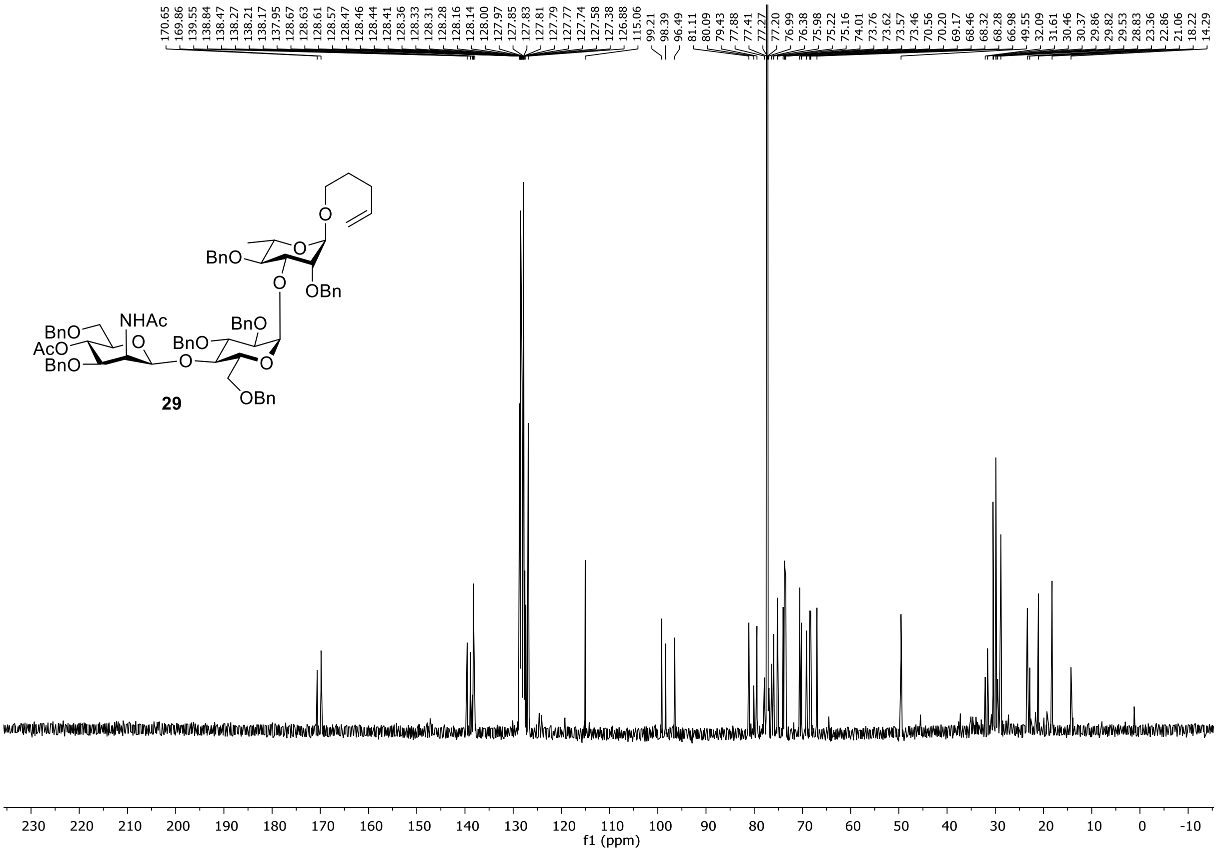

CH-HSQC NMR, 600 MHz, $CDCl_3$

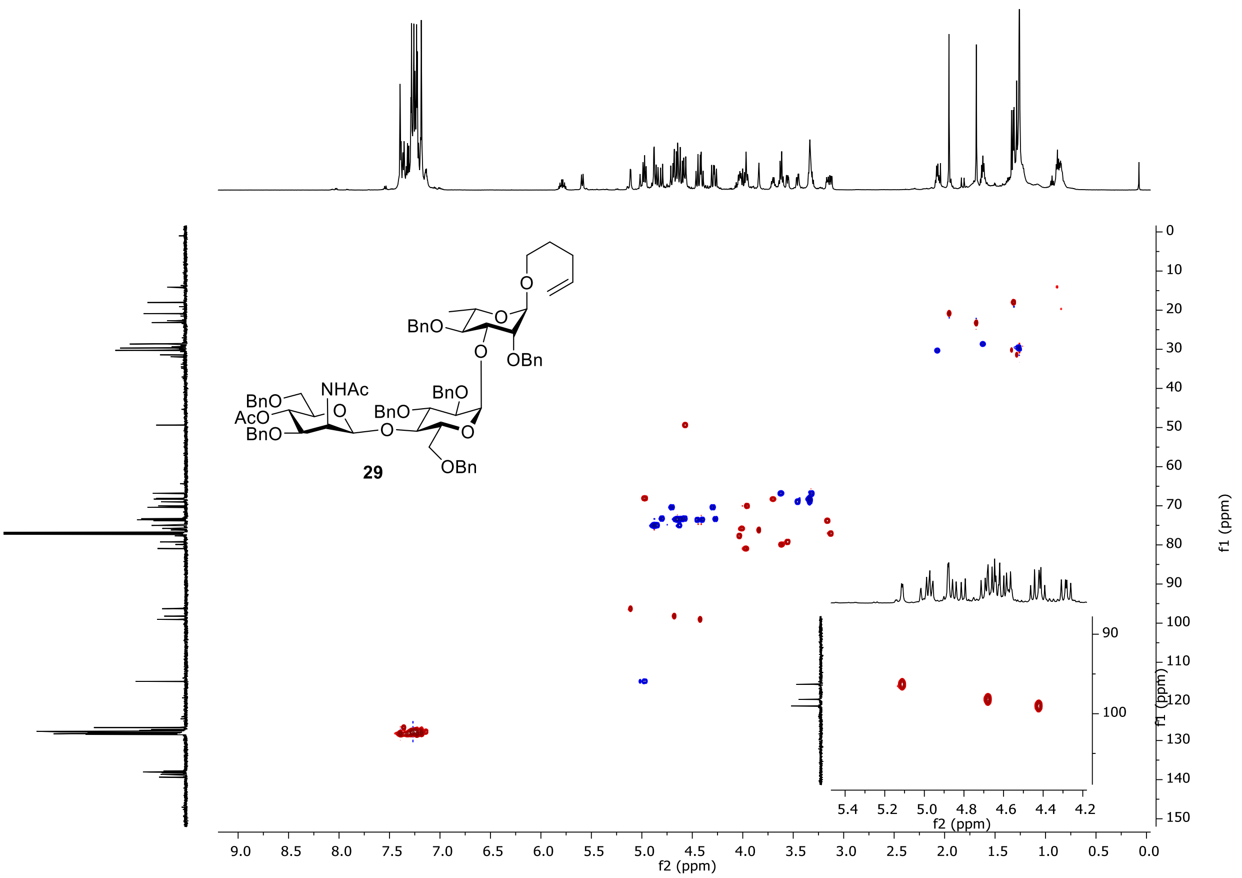

CH-HSQC NMR, 600 MHz, $CDCl_3$

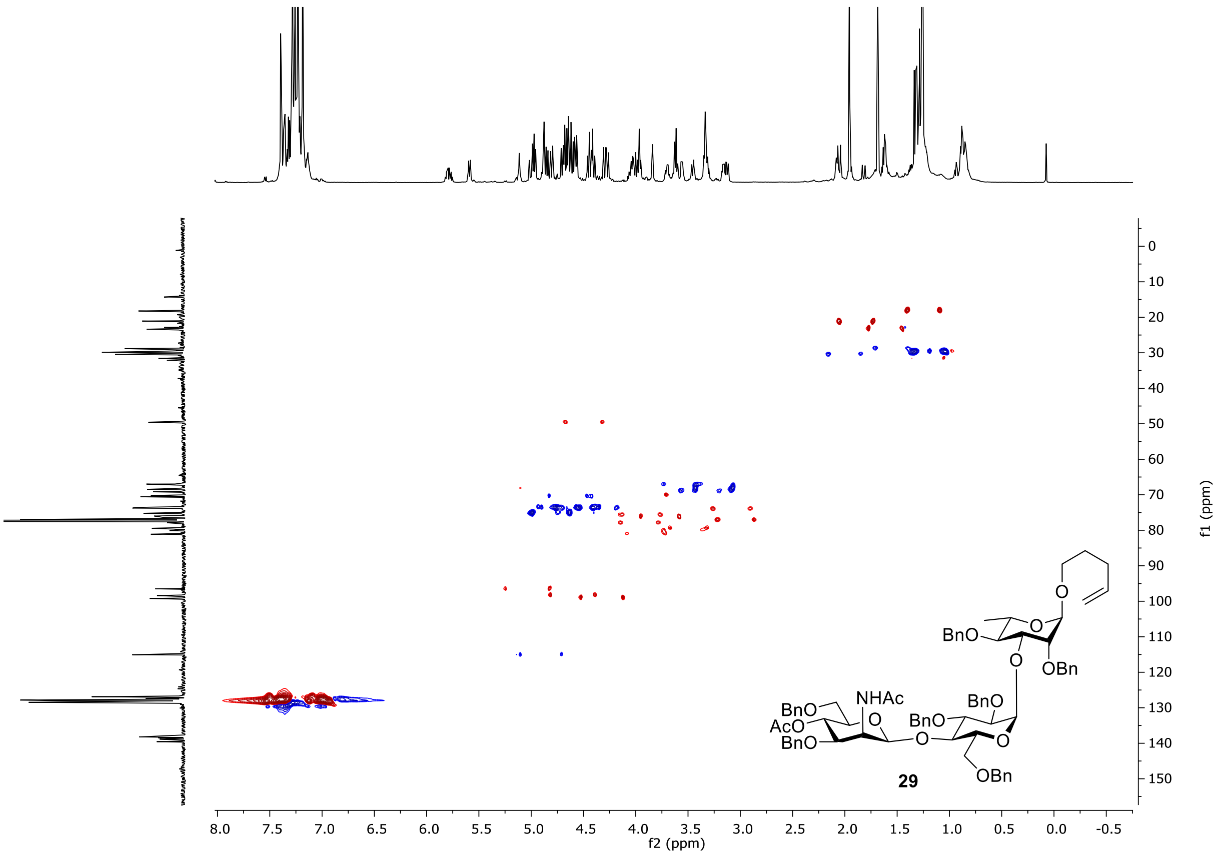

1H NMR, 600 MHz, $CDCl_3$

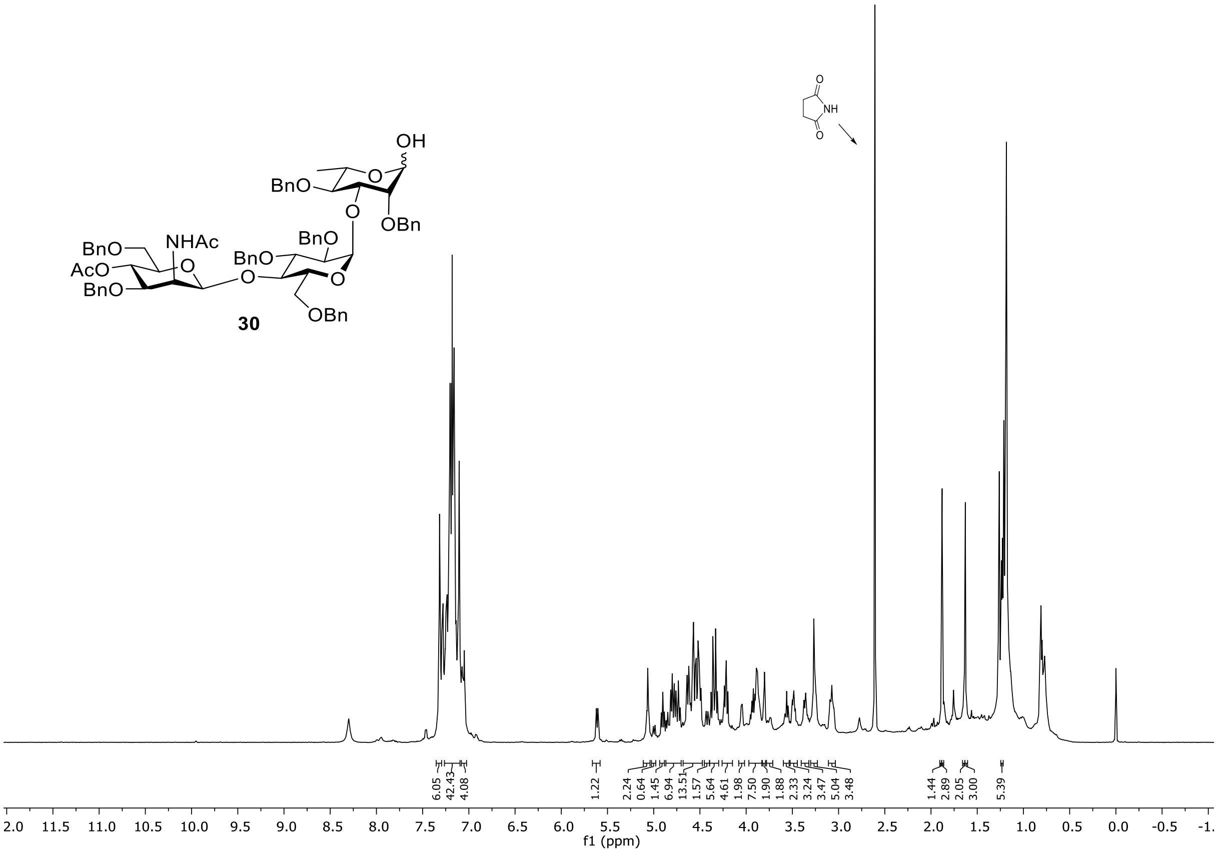

13C NMR, 400 MHz, $CDCl_3$

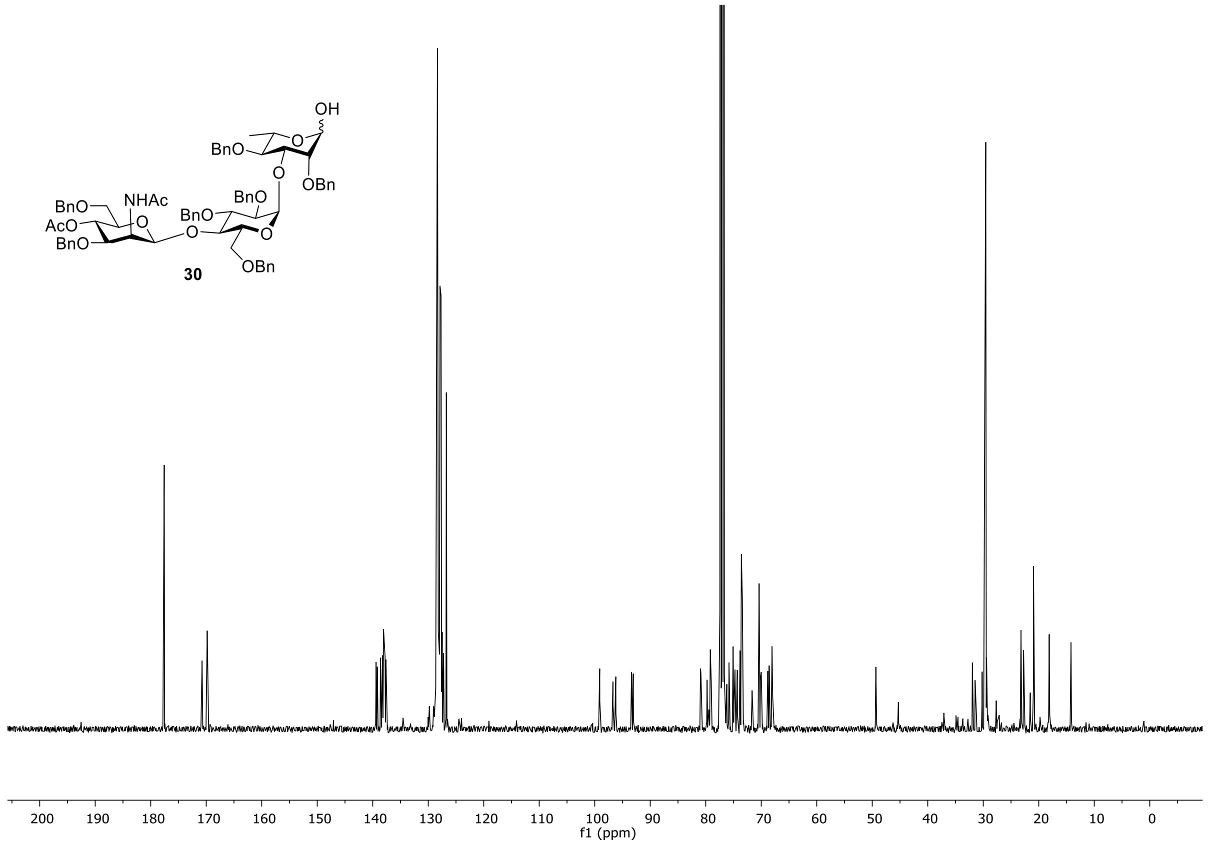

CH-HSQC NMR, 600 MHz, $CDCl_3$

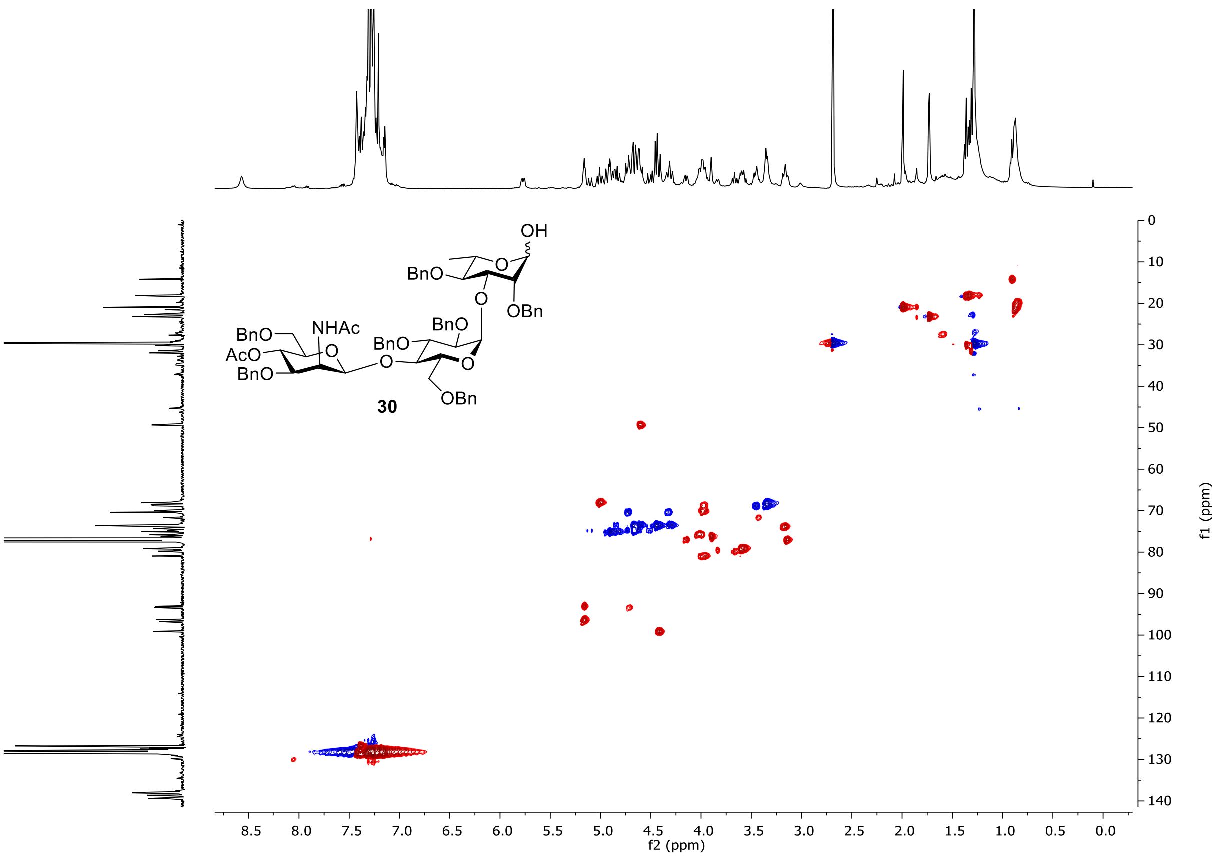

1H NMR, 600 MHz, $CDCl_3$

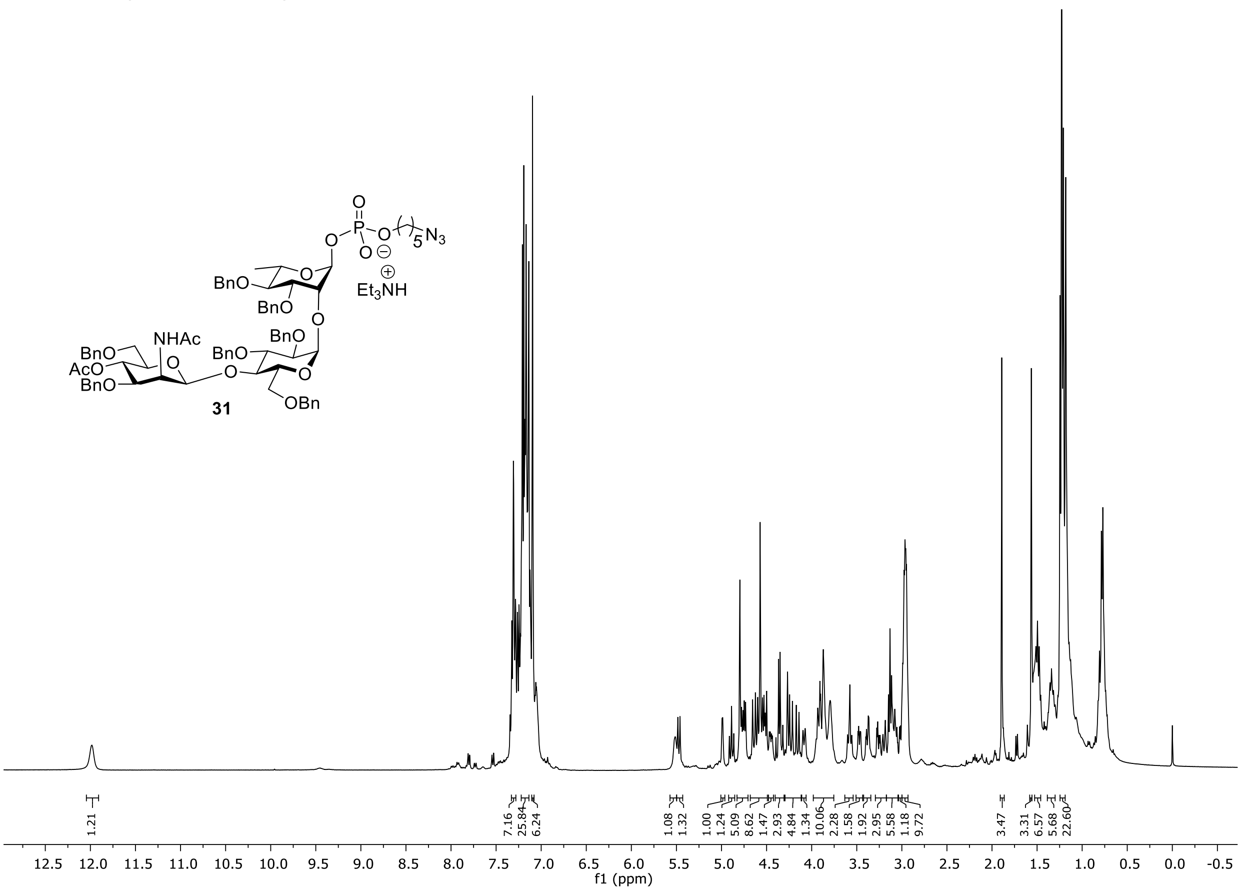

31P NMR, 600 MHz, $CDCl_3$

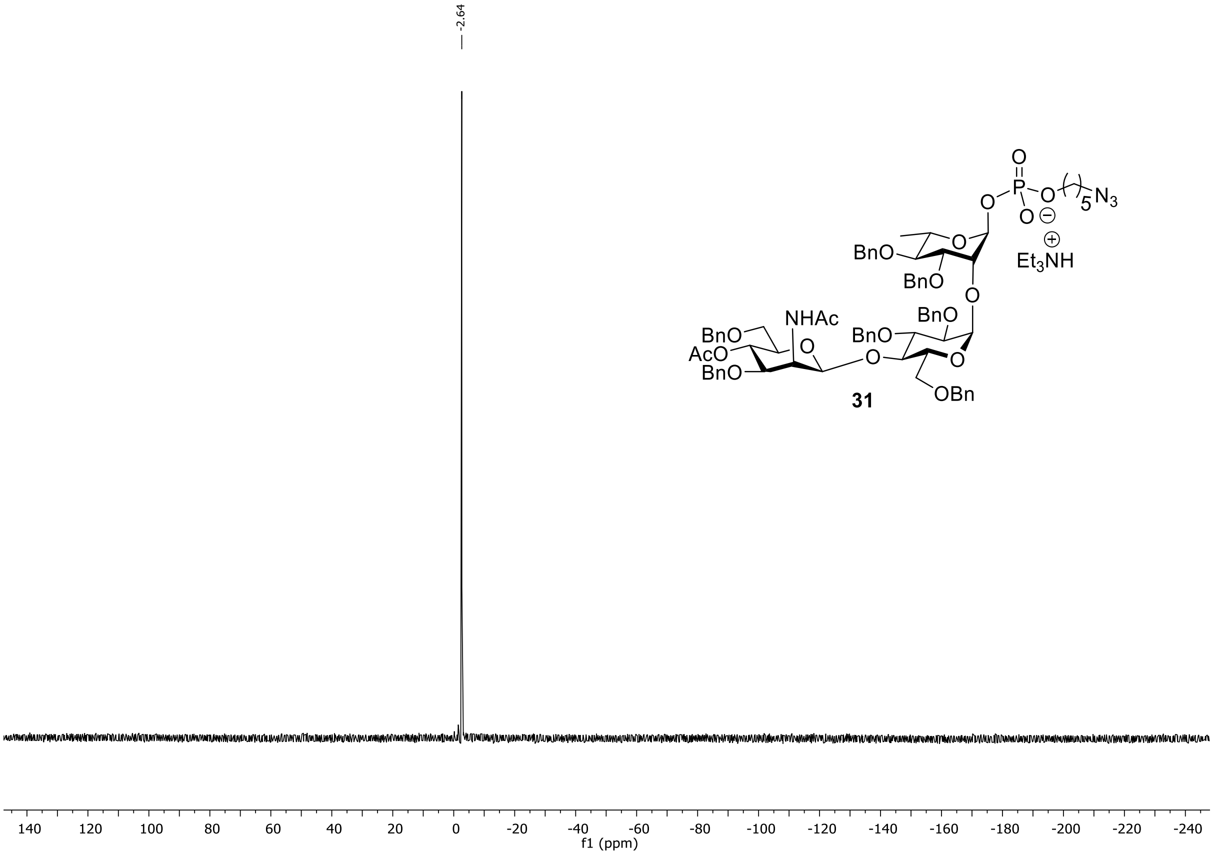

13C NMR, 600 MHz, $CDCl_3$

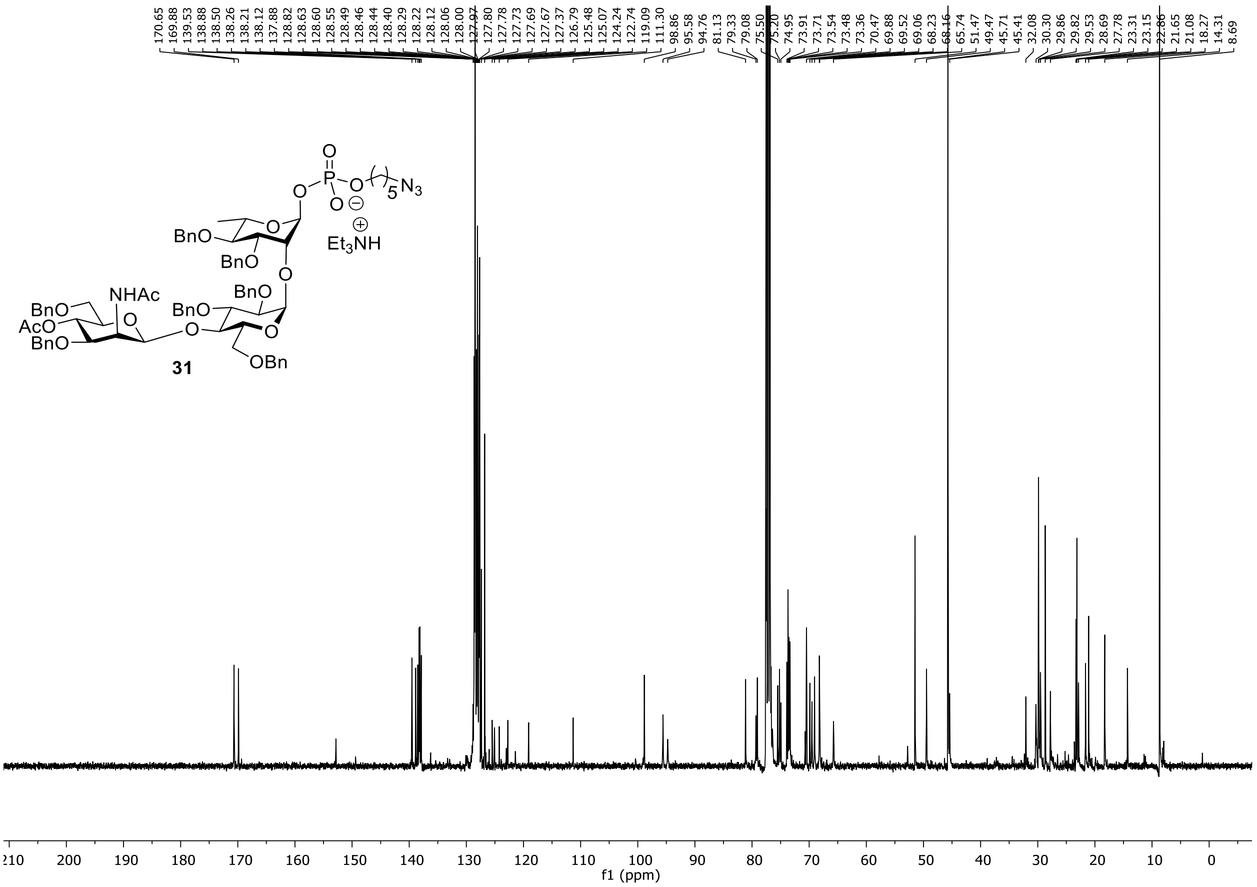

CH-HSQC NMR, 600 MHz, $CDCl_3$

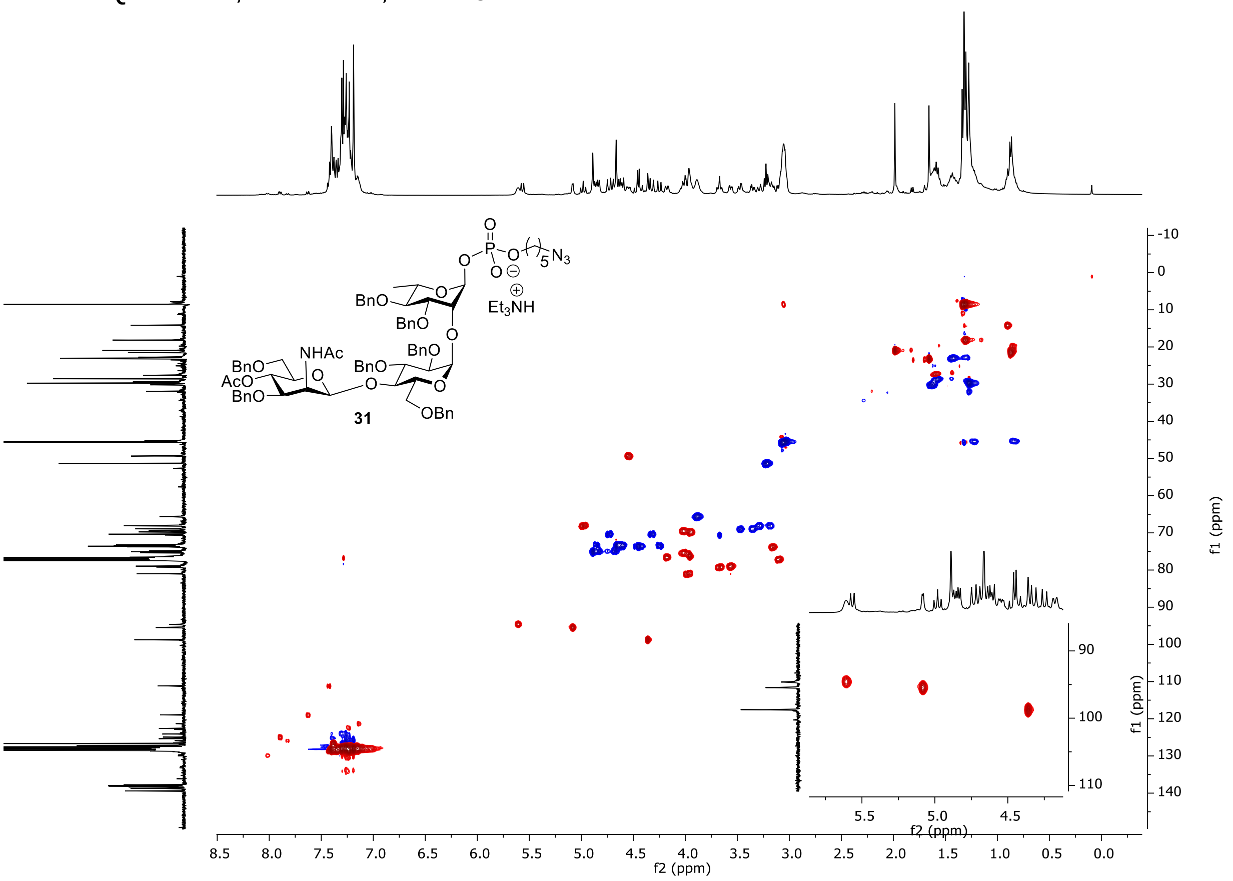

CH-HSQC NMR, 600 MHz, $CDCl_3$

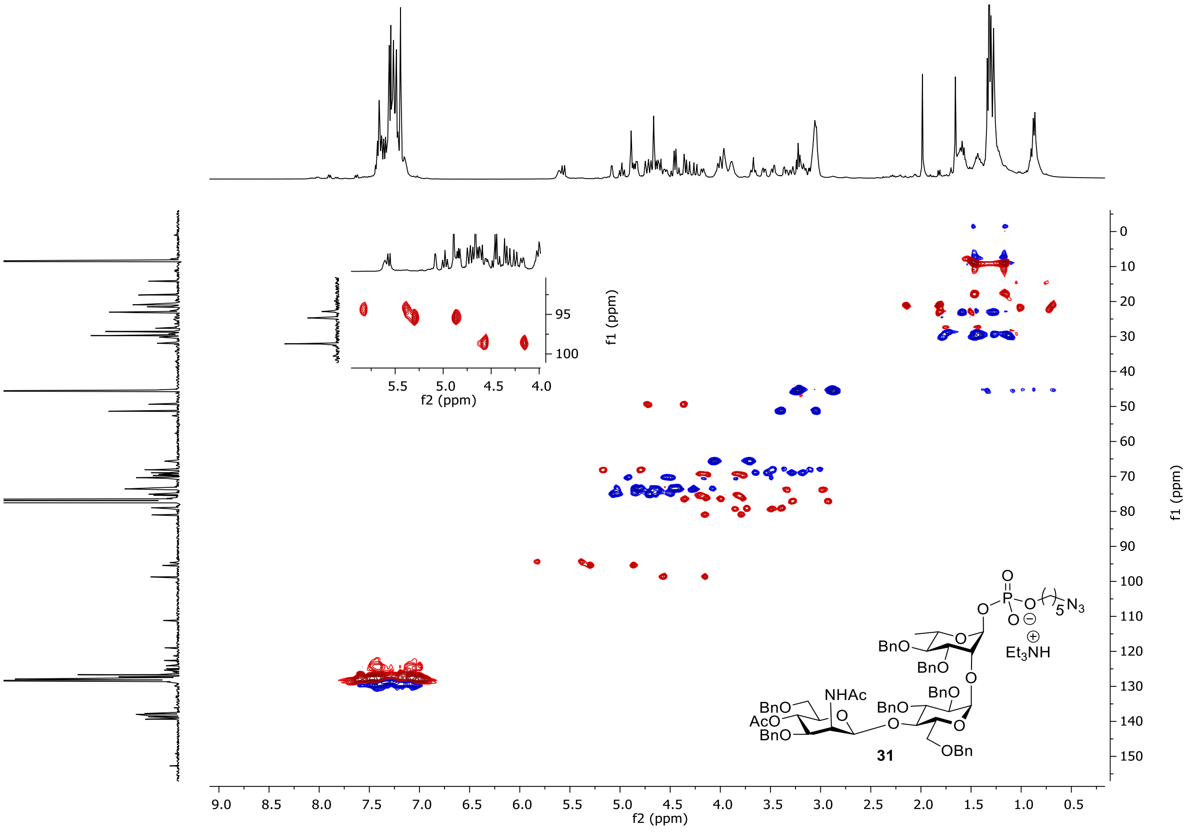

CH-HSQC NMR, 600 MHz, $CDCl_3$ (expansion)

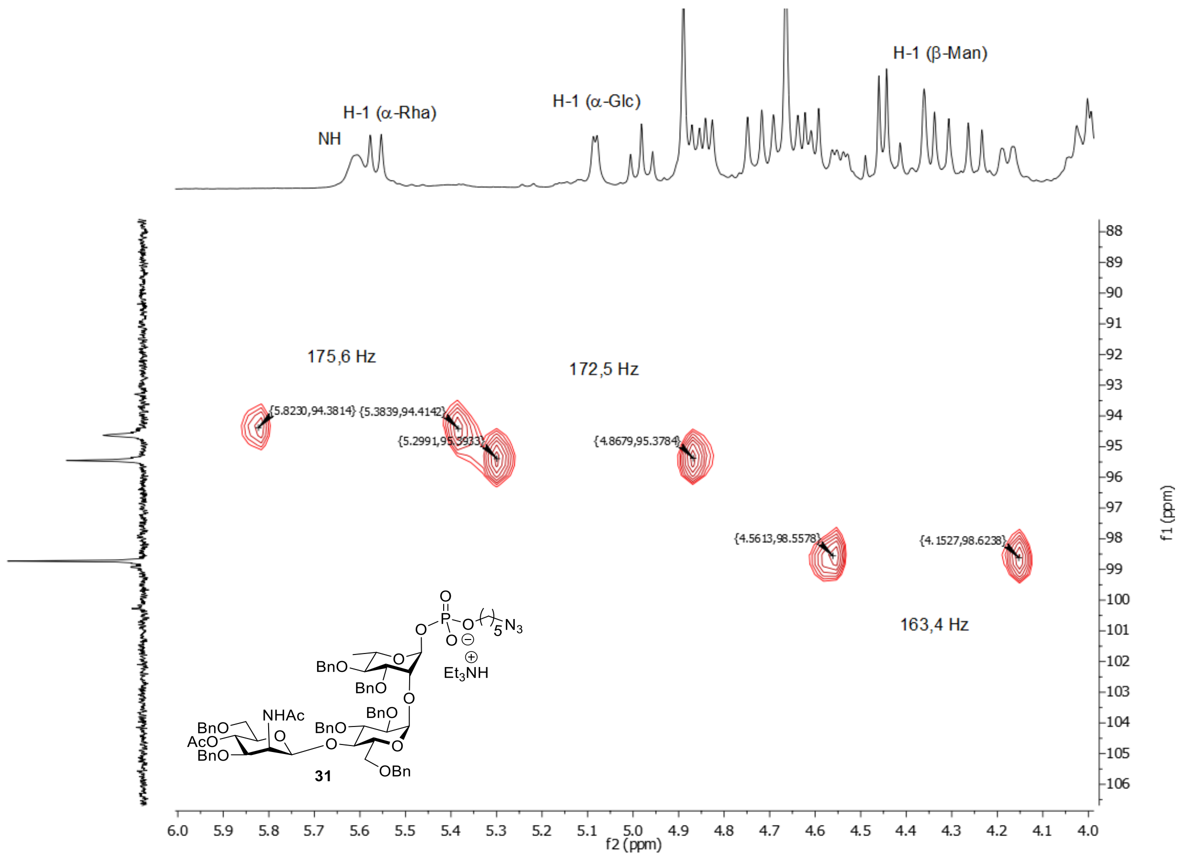

1H NMR, 600 MHz, $CDCl_3$

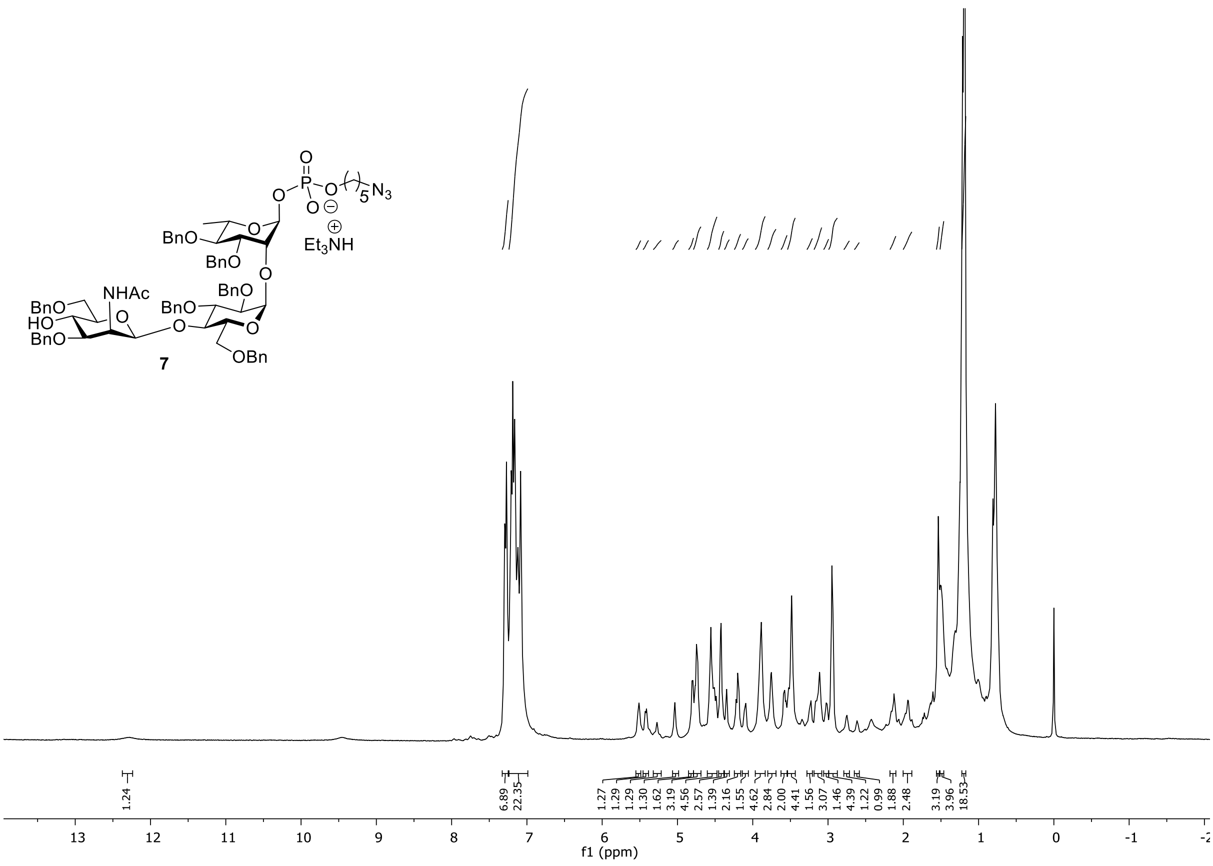

31P NMR, 162 MHz, CDCl3

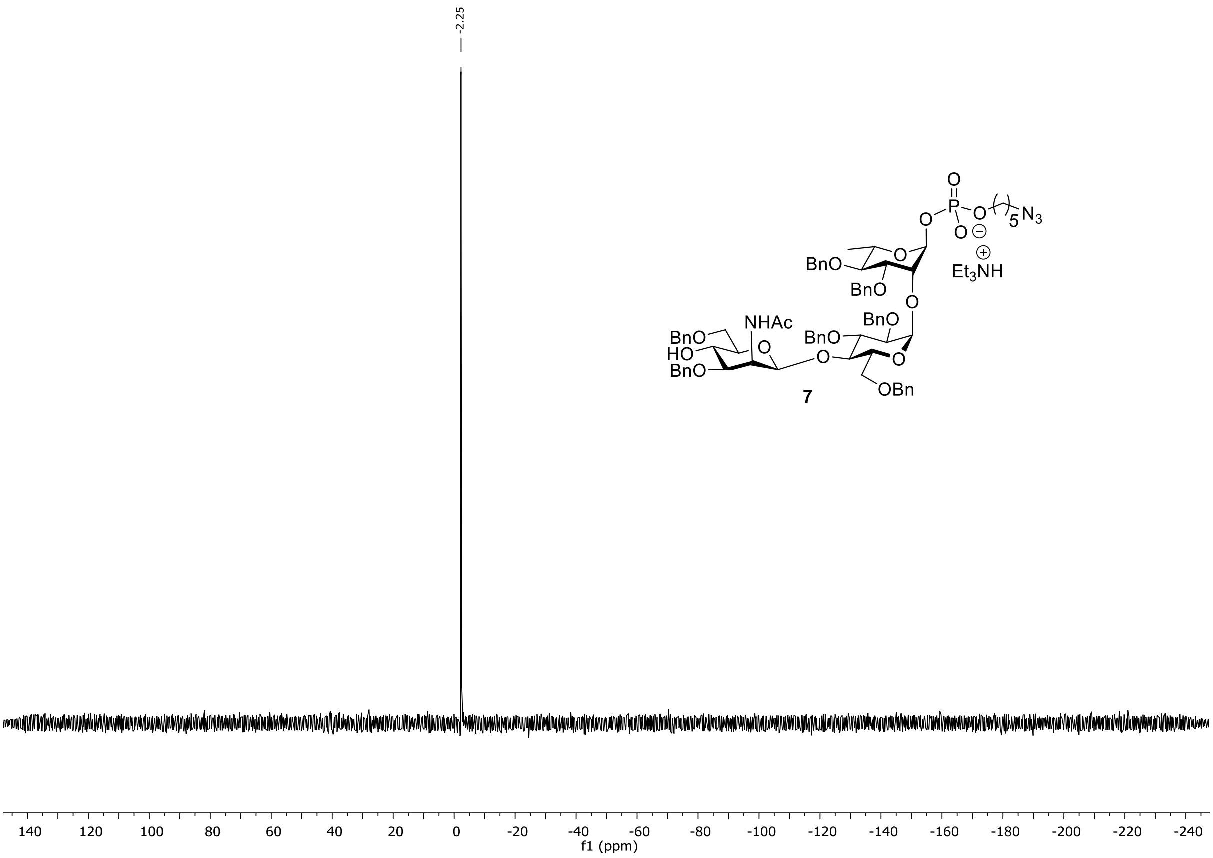

13C NMR, 176 MHz, CDCl3

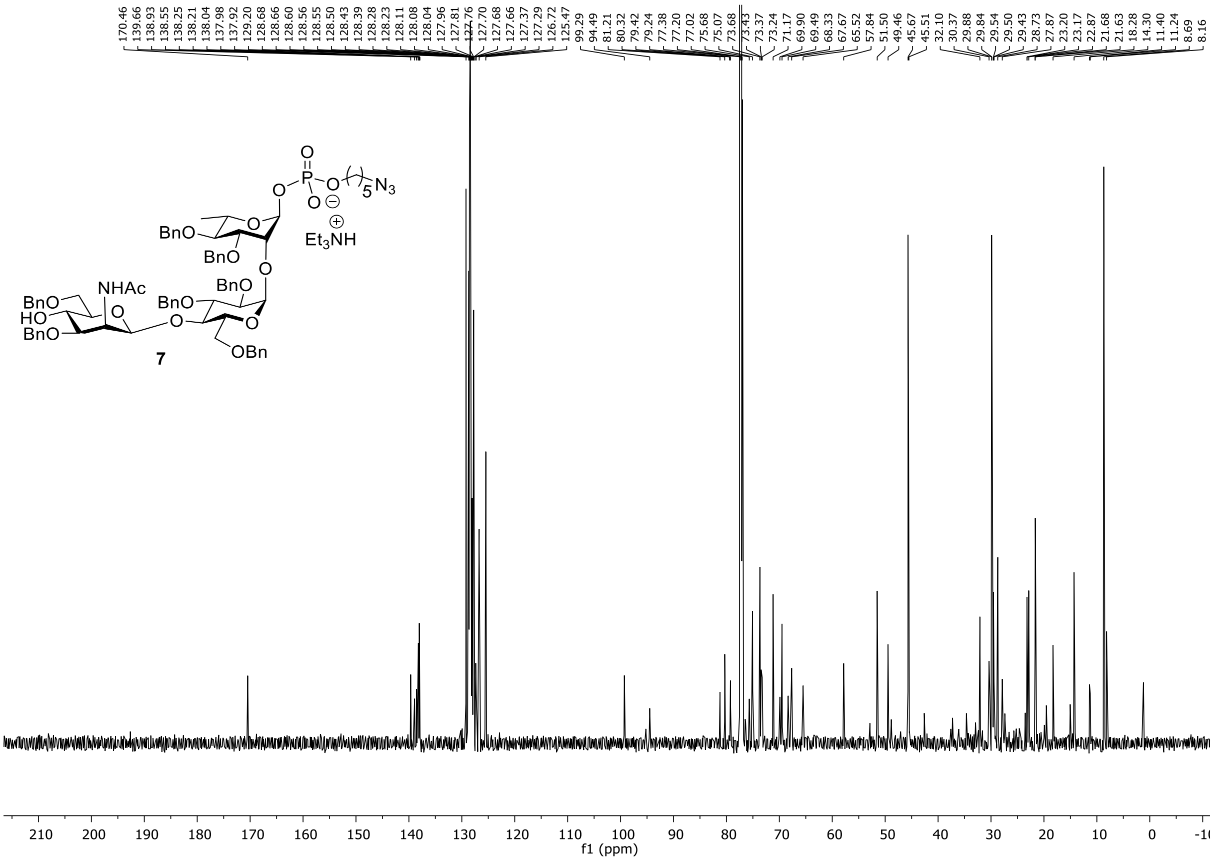

CH-HSQC NMR, 600 MHz, $CDCl_3$

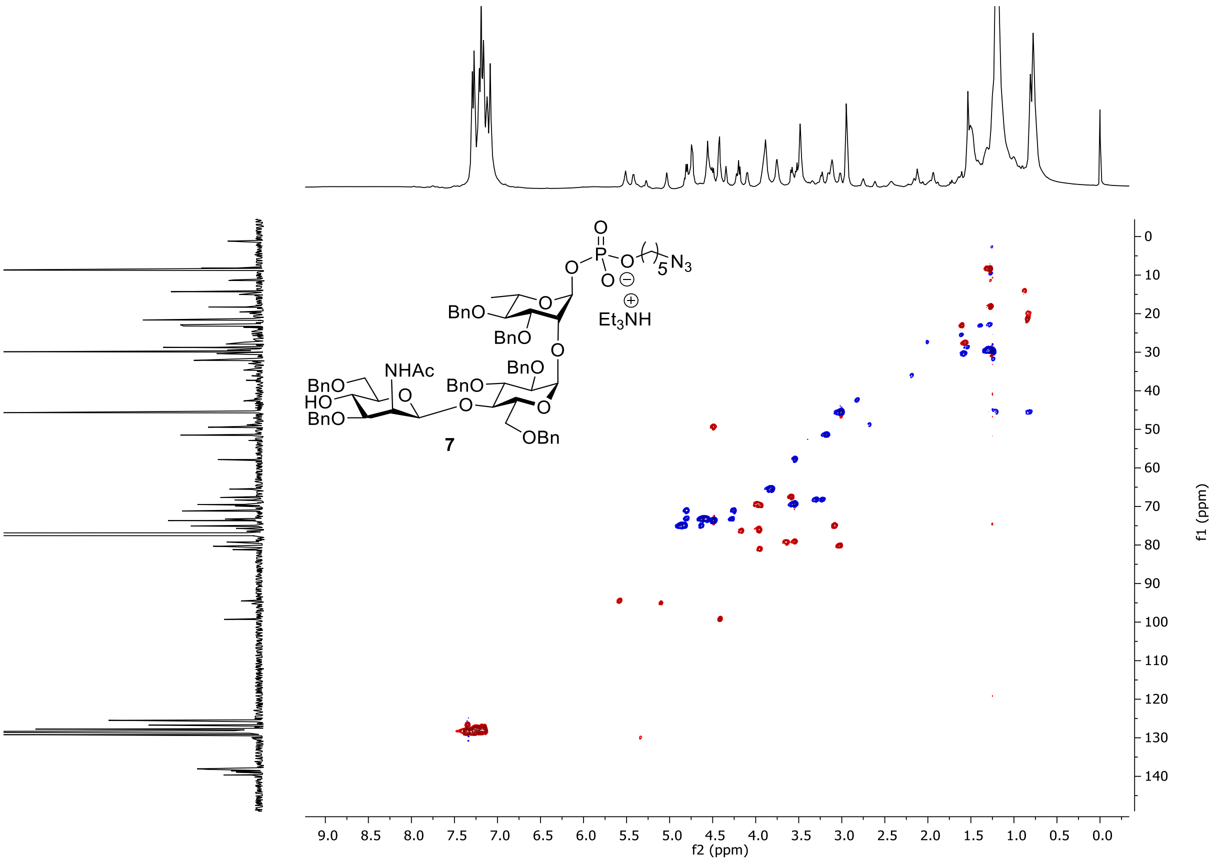

CH-HSQC NMR, 600 MHz, $CDCl_3$

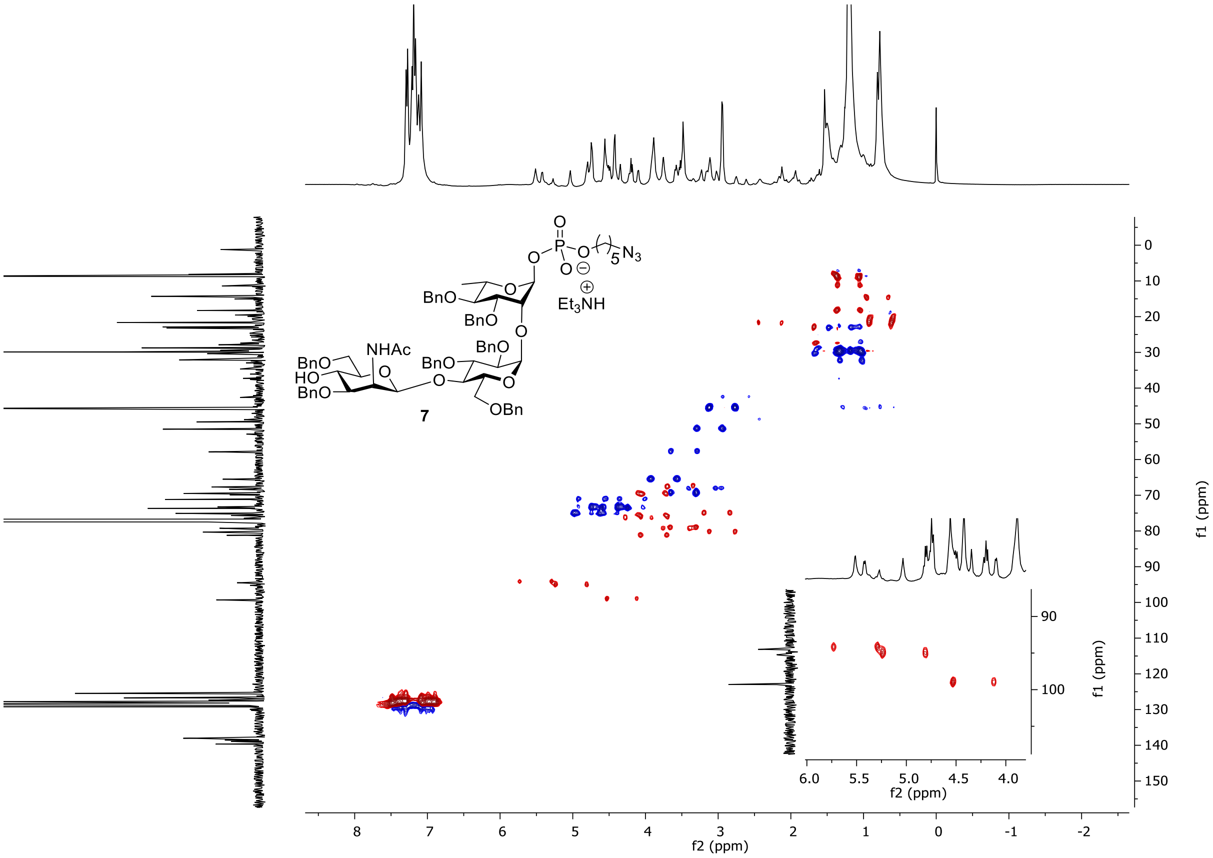

1H NMR, 600 MHz, $CDCl_3$

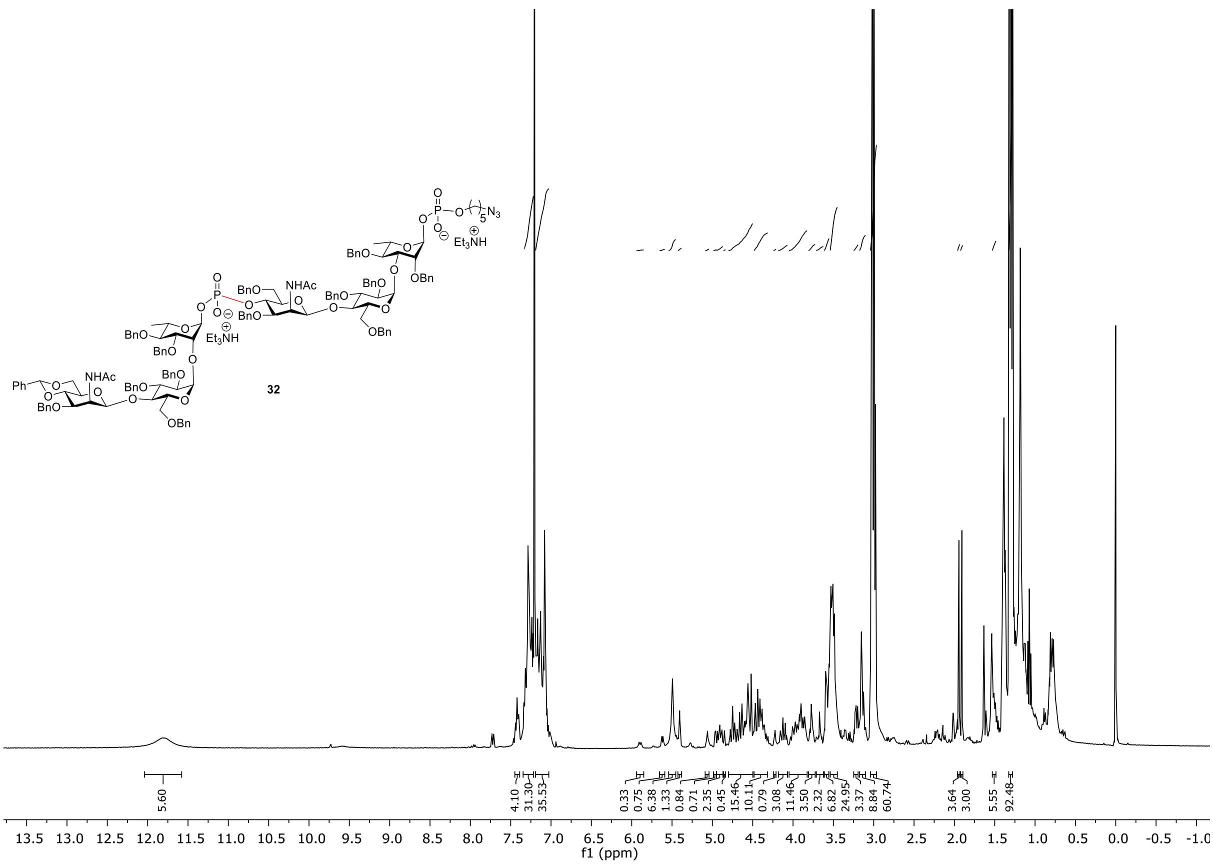

31P NMR, 243 MHz, $CDCl_3$

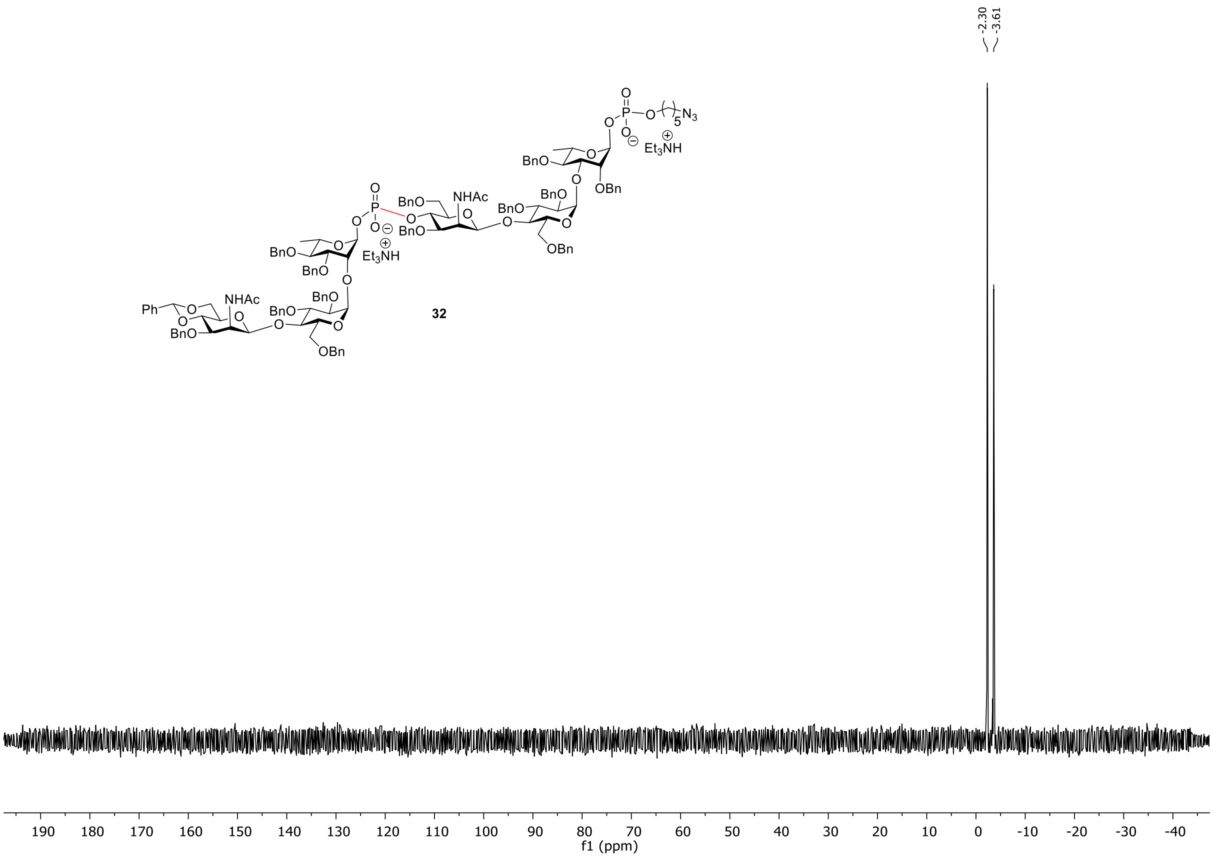

13C NMR, 176 MHz, $CDCl_3$

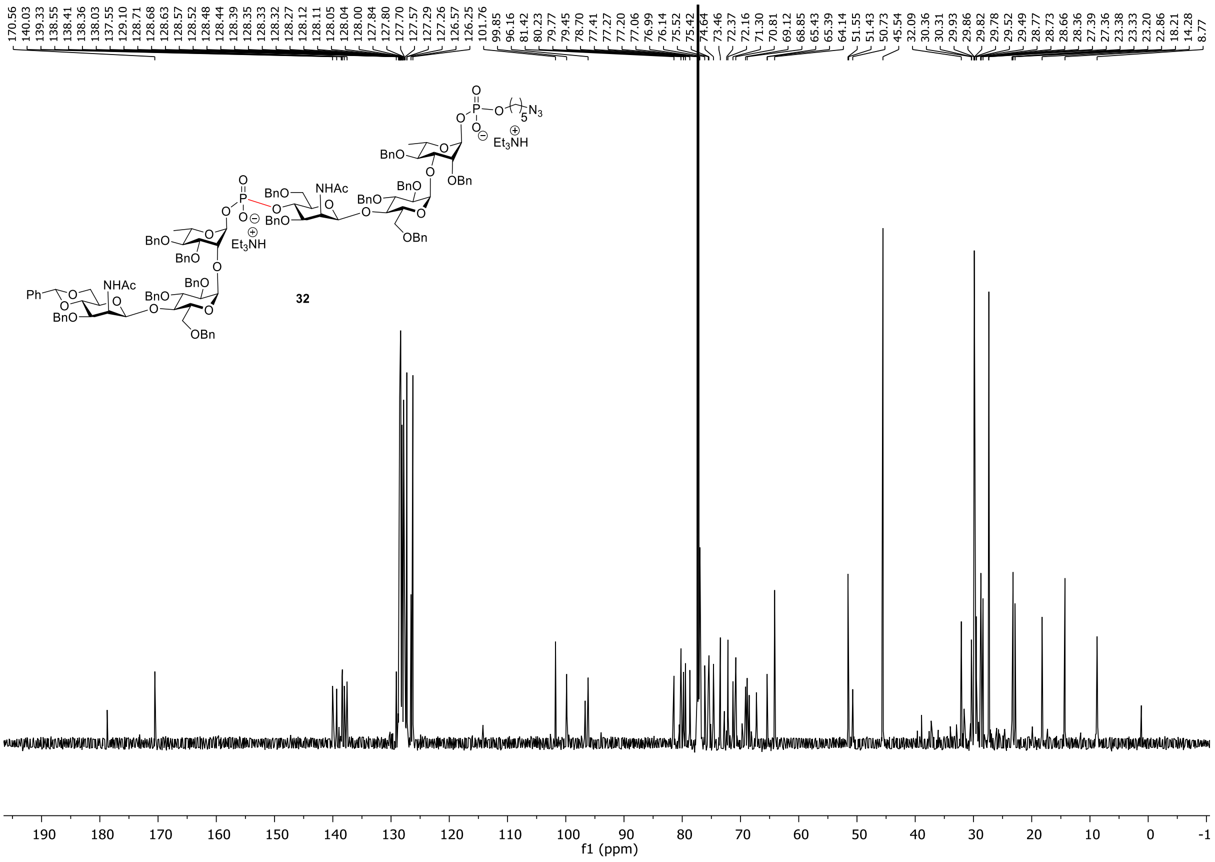

CH-HSQC NMR, 600 MHz, $CDCl_3$

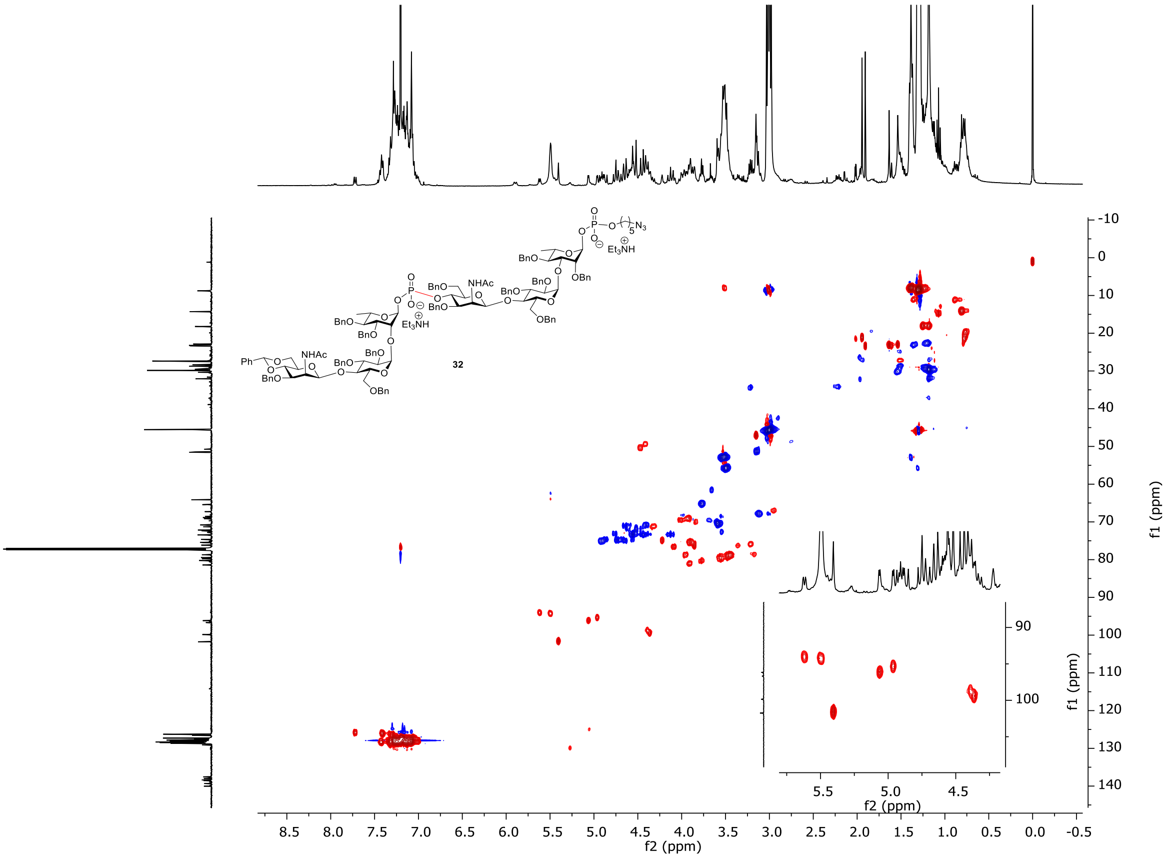

CH-HSQC NMR, 600 MHz, $CDCl_3$

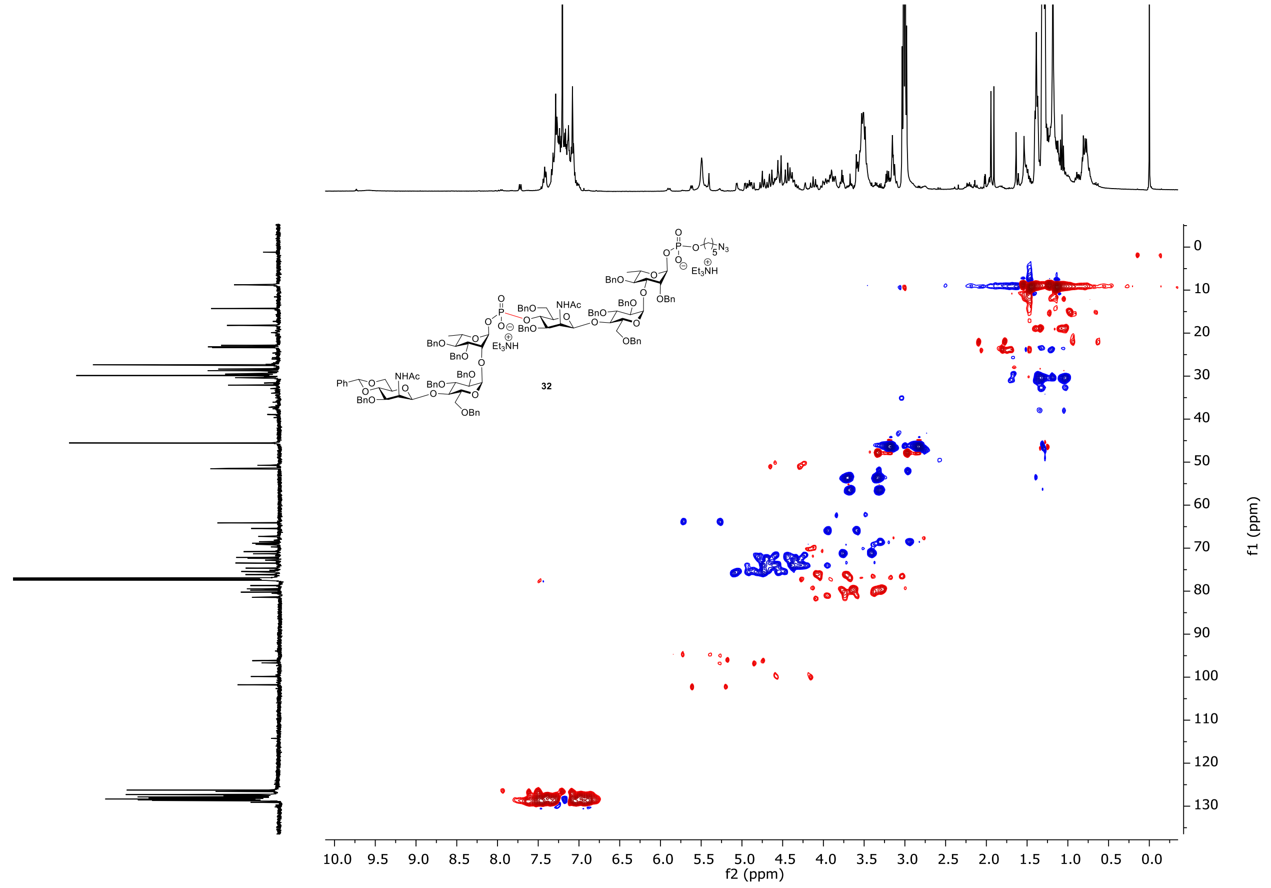

1H NMR, 600 MHz, D_2O

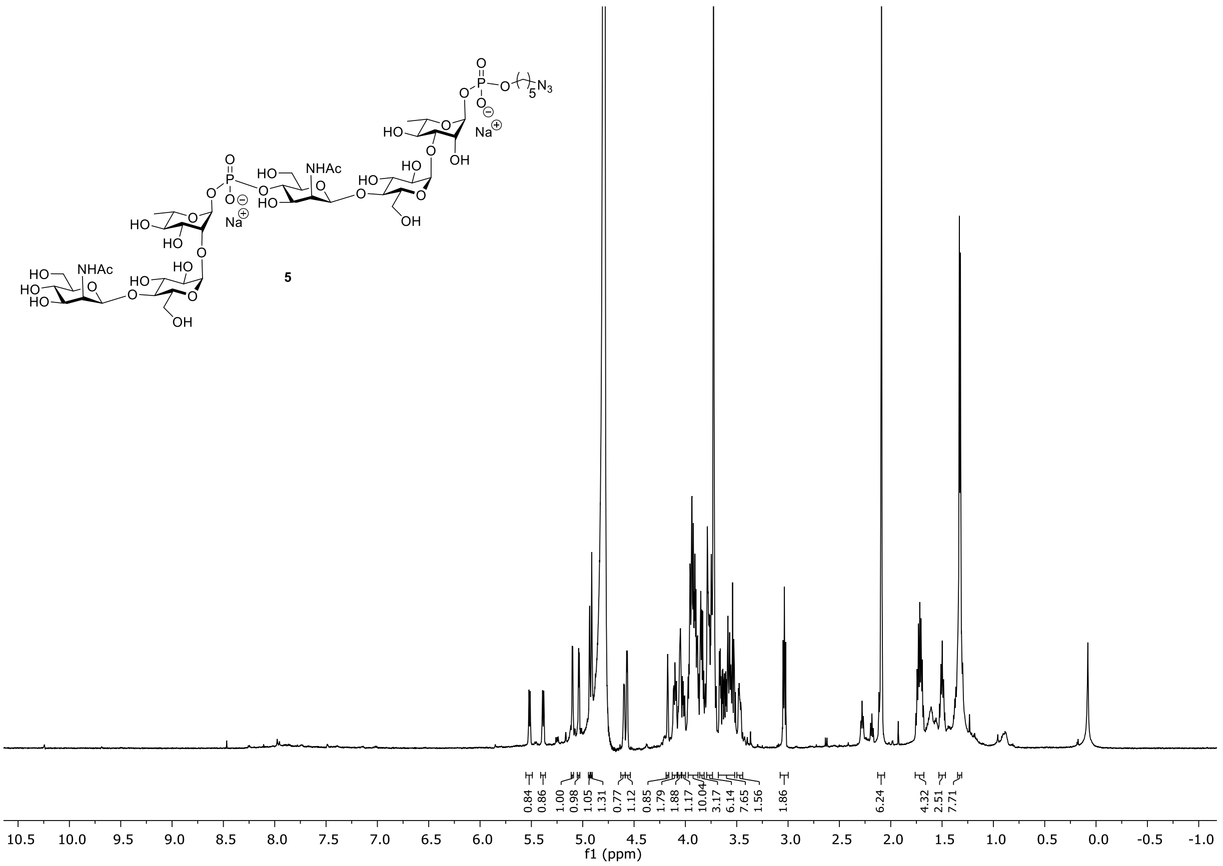

31 P NMR, 162 MHz, D2O

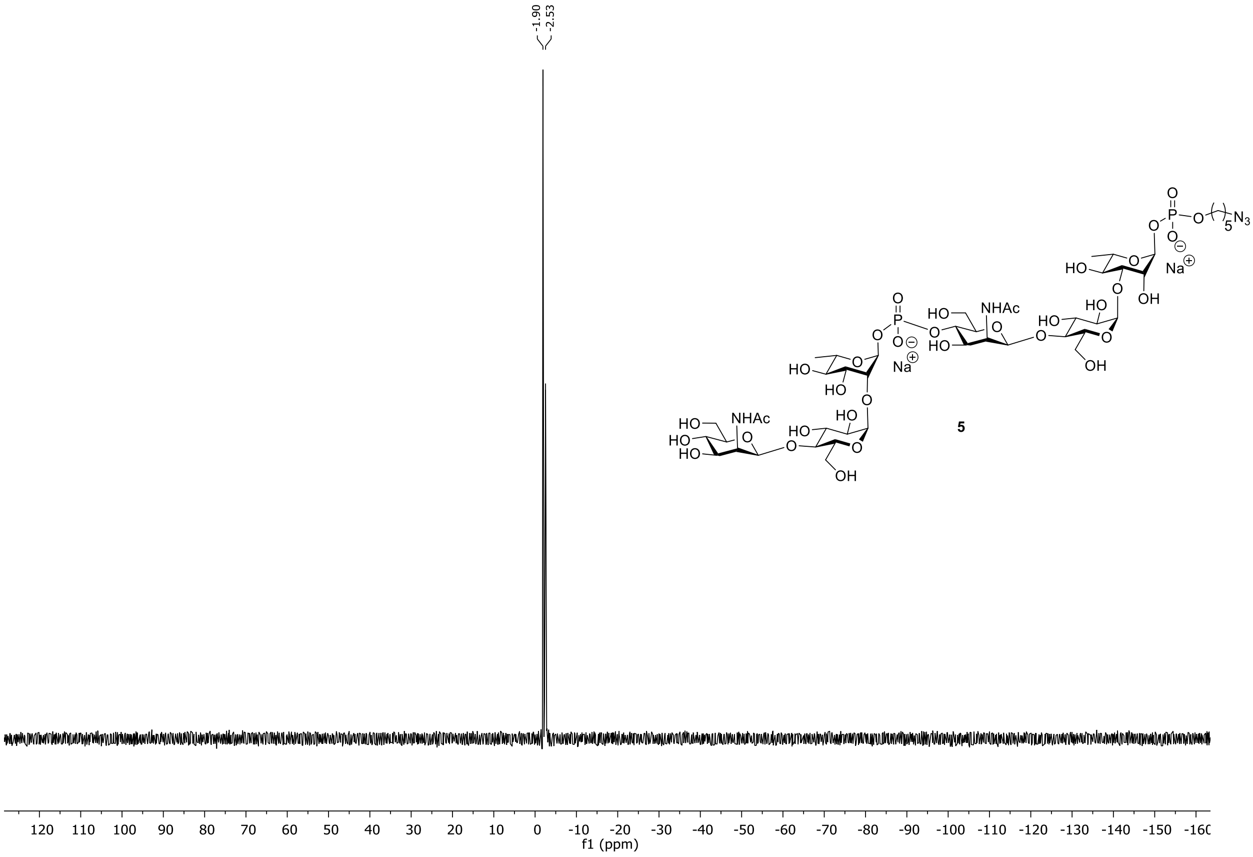

5

13C NMR, 151 MHz, D2O

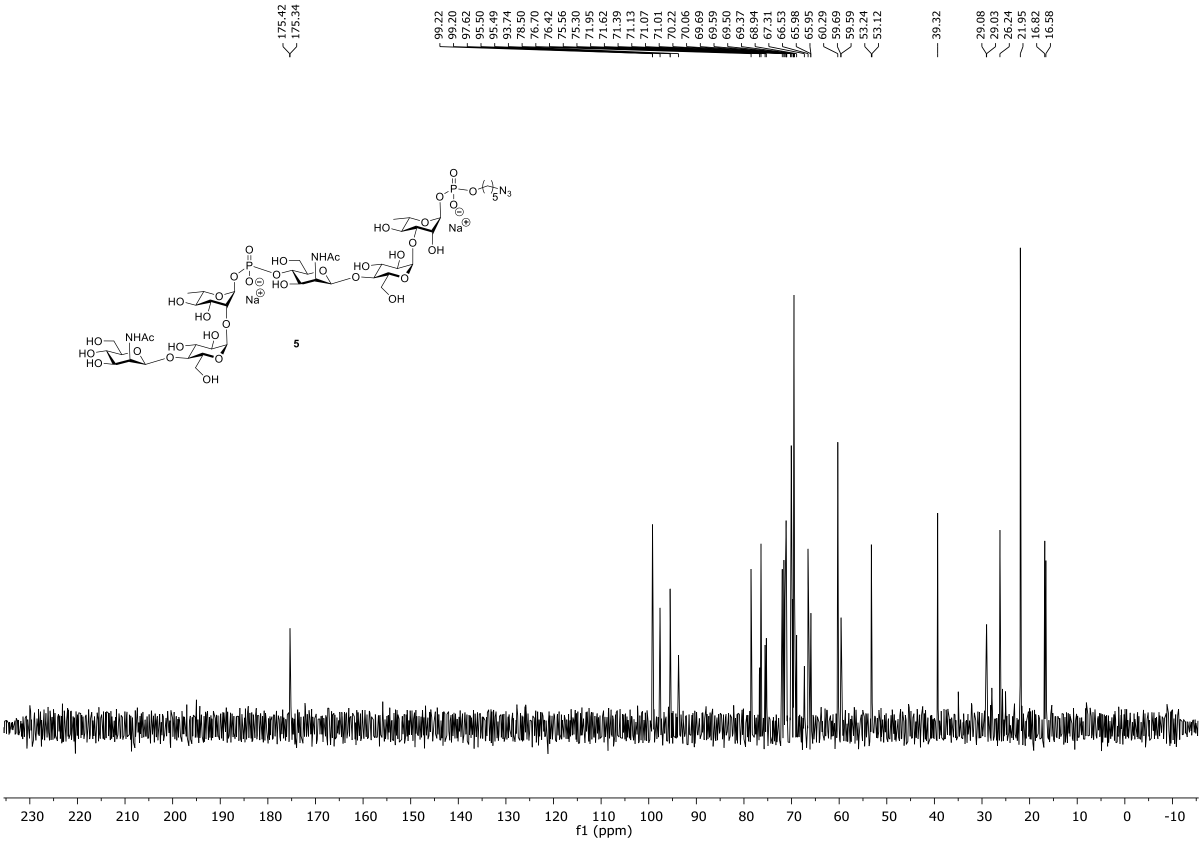

5

CH-HSQC NMR, 600 MHz, D_2O

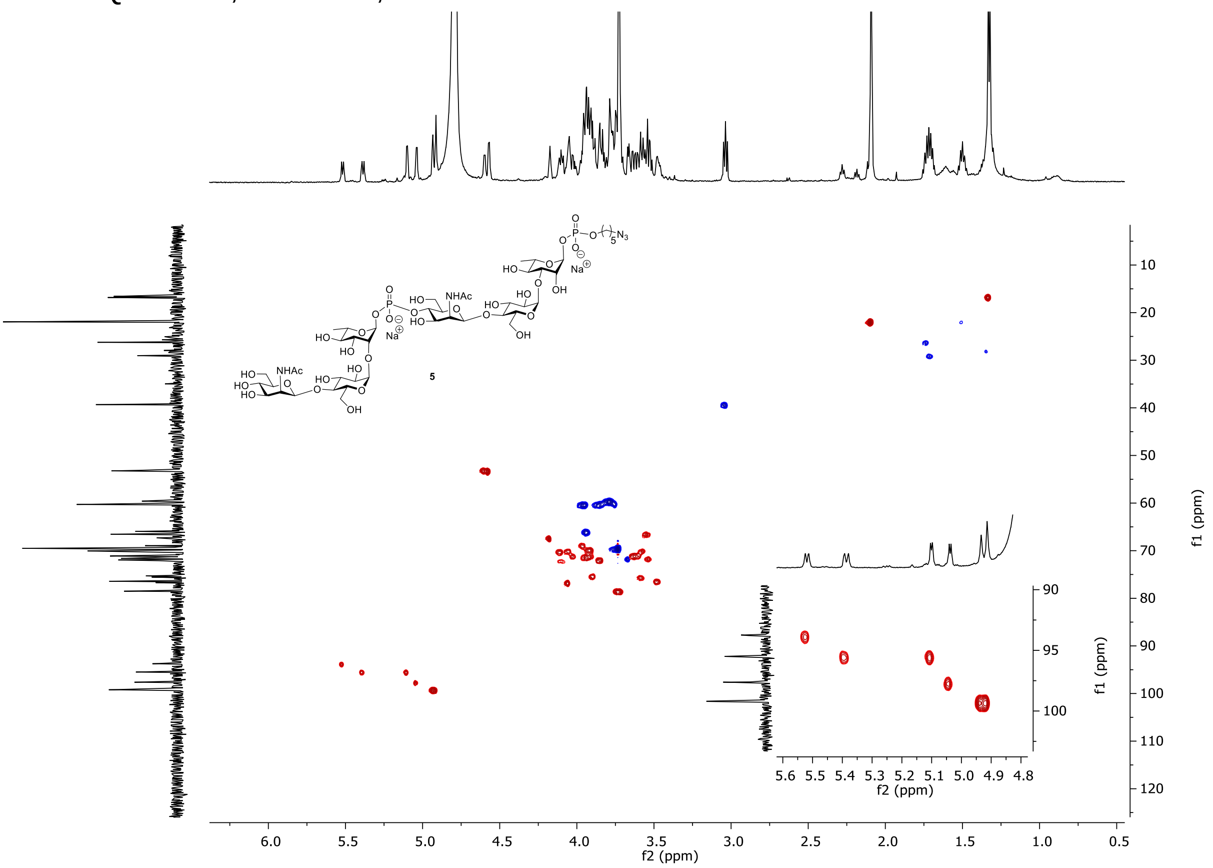

CH-HSQC NMR, 600 MHz, D_2O

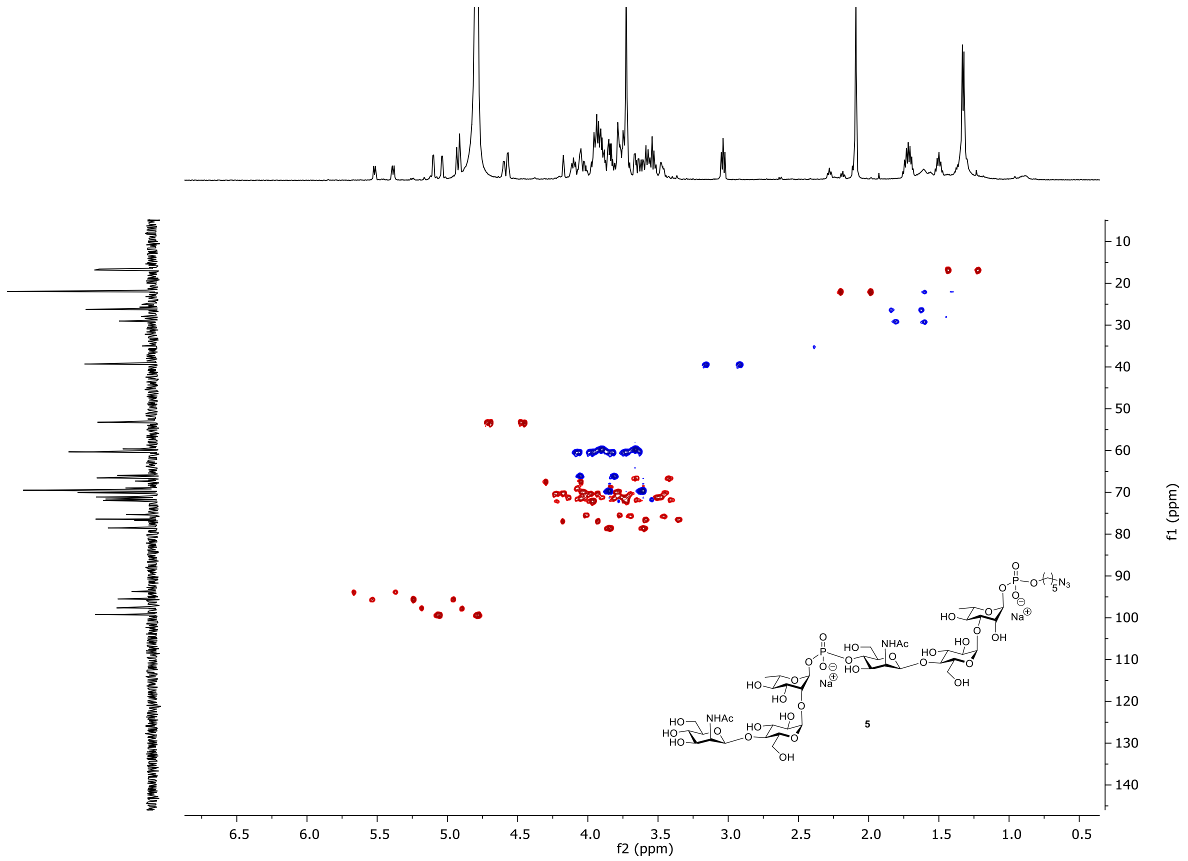

CH-HSQC NMR, 600 MHz, D_2O (Expansion)

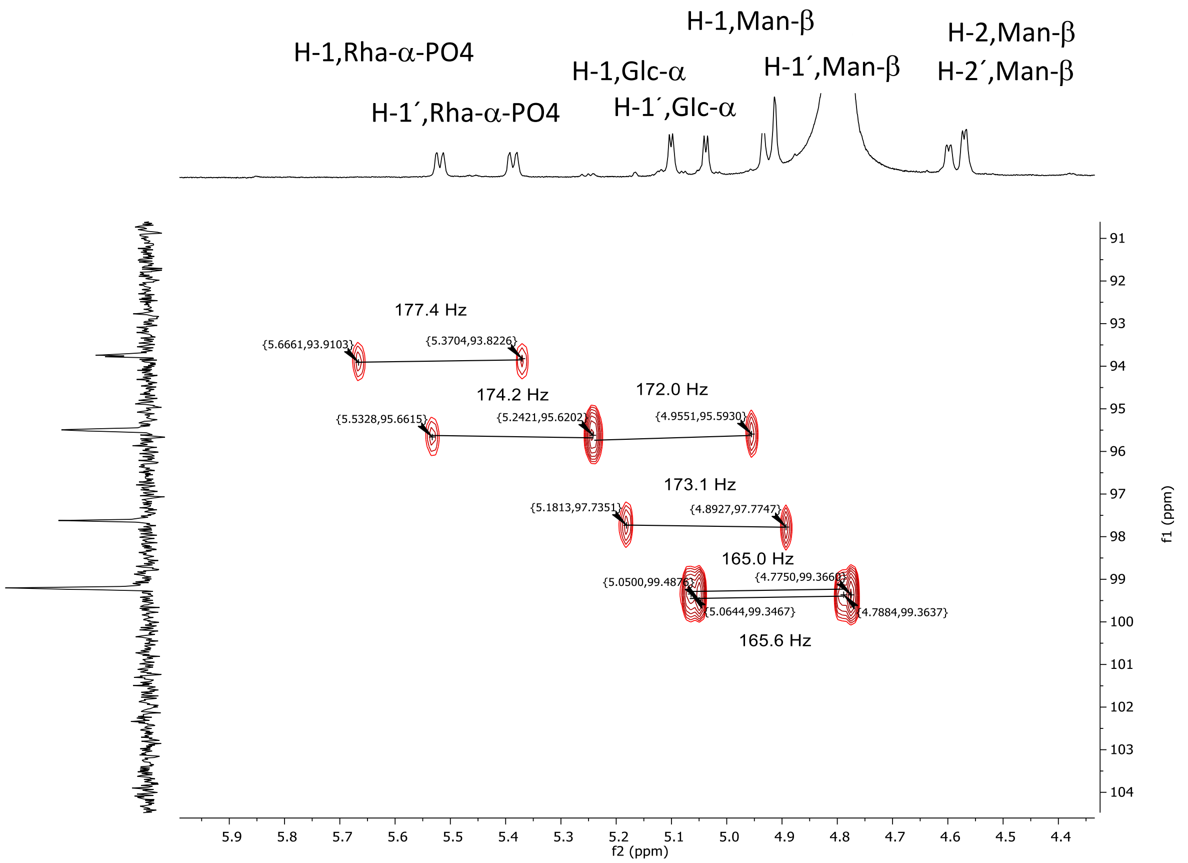

HRMS (Q-Tof)

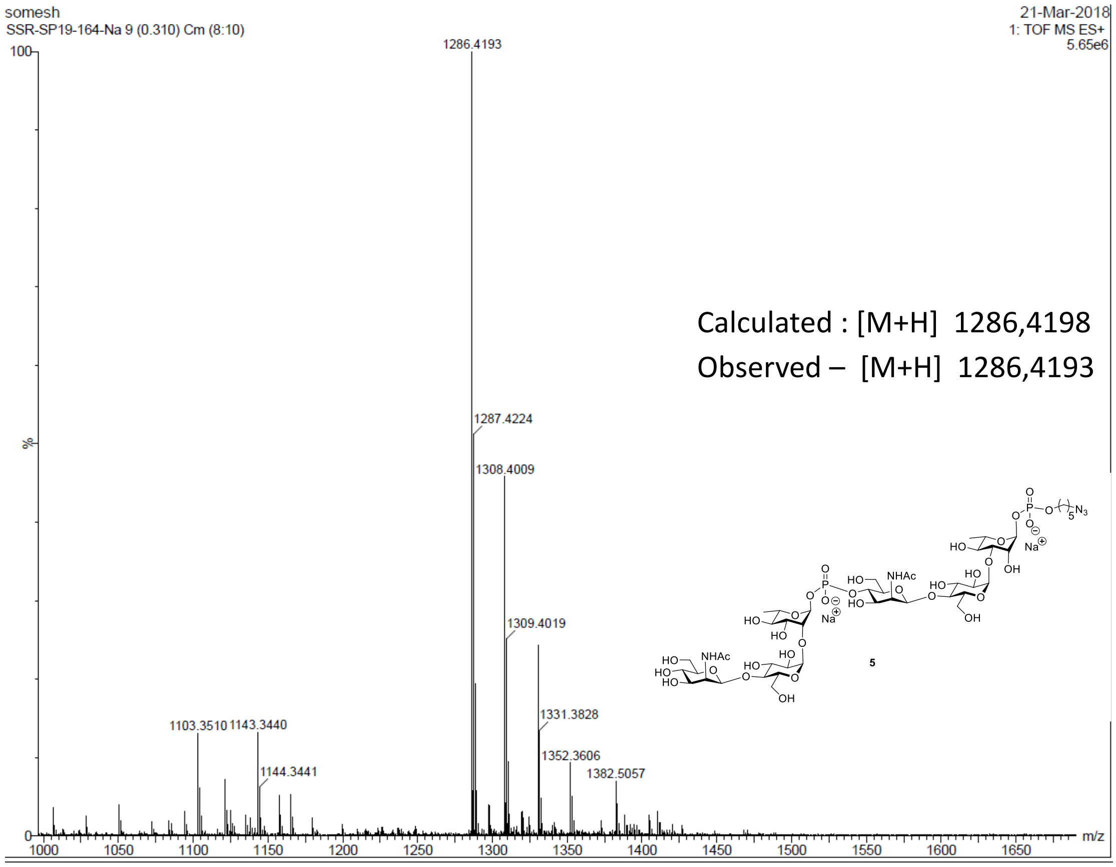

References

1. G. Guchhait, A. K. Misra, *Tetrahedron Asymmetry* 2009, **20**, 1791–1797.
2. C. Uriel, A. M. Gómez, J. C. López, B. Fraser-Reid, *Synlett* 2003, **14**, 2203–2207.
3. L. J. Liotta, R. D. Capotosto, R. A. Garbitt, B. M. Horan, P. J. Kelly, A. P. Koleros, L. M. Brouillette, A. M. Kuhn, S. Targontsidis, *Carbohydr. Res.* 2001, **331**, 247-253.
4. P. Xu, W. Xu, Y. Dai, Y. Yang, B. Yu, *Org. Chem. Front*. 2014, **1**, 405–414.
5. F. Broecker, P. H. Seeberger, *Methods Mol. Biol.* 2017**, 1518**, 227-240.
6. R. L. Burton, M. H. Nahm, *Clin. Vaccine Immunol.* 2006**, 13**, 1004-1009.
7. R. L. Burton, H. W. Kim, S. Lee, H. Kim, J. H. Seok, S. H. Lee, A. Balloch, P. Licciardi, R. Marimla, S. Bae, M. H. Nahm, K. H. Kim, *Medicine (Baltimore)* 2018**, 97**, e0567.
